# Mechanism of Nitrogen Reduction to Ammonia in a Diiron Model of Nitrogenase

**DOI:** 10.1021/acs.inorgchem.3c02089

**Published:** 2023-08-31

**Authors:** Maxim Barchenko, Patrick J. O’Malley, Sam P. de Visser

**Affiliations:** † Manchester Institute of Biotechnology, The University of Manchester, 131 Princess Street, Manchester M1 7DN, U.K.; ‡ Department of Chemistry, The University of Manchester, Oxford Road, Manchester M13 9PL, U.K.; § Department of Chemical Engineering, The University of Manchester, Oxford Road, Manchester M13 9PL, U.K.

## Abstract

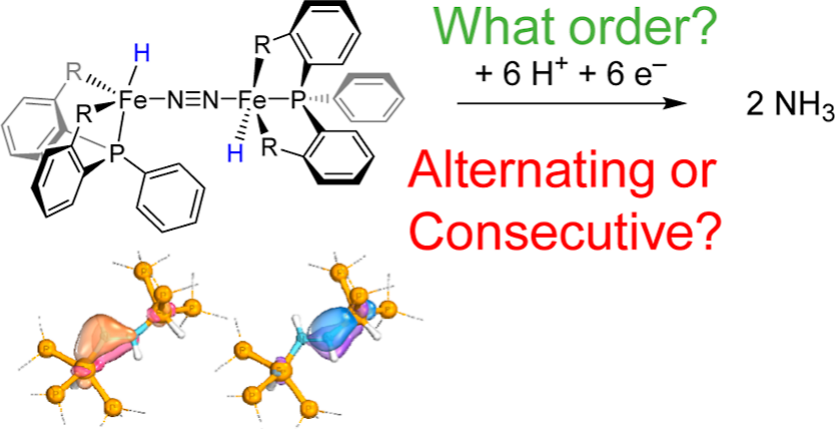

Nitrogenase is a fascinating enzyme in biology that reduces dinitrogen from air to ammonia through stepwise reduction and protonation. Despite it being studied in detail by experimental and computational groups, there are still many unknown factors in the catalytic cycle of nitrogenase, especially related to the addition of protons and electrons and their order. A recent biomimetic study characterized a potential dinitrogen-bridged diiron cluster as a synthetic model of nitrogenase. Using strong acid and reductants, the dinitrogen was converted into ammonia molecules, but details of the mechanism remains unknown. In particular, it was unclear from the experimental studies whether the proton and electron transfer steps are sequential or alternating. Moreover, the work failed to establish what the function of the diiron core is and whether it split into mononuclear iron fragments during the reaction. To understand the structure and reactivity of the biomimetic dinitrogen-bridged diiron complex [(P_2_^P′Ph^FeH)_2_(μ-N_2_)] with triphenylphosphine ligands, we performed a density functional theory study. Our computational methods were validated against experimental crystal structure coordinates, Mössbauer parameters, and vibrational frequencies and show excellent agreement. Subsequently, we investigated the alternating and consecutive addition of electrons and protons to the system. The calculations identify a number of possible reaction channels, namely, same-site protonation, alternating protonation, and complex dissociation into mononuclear iron centers. The calculations show that the overall mechanism is not a pure sequential set of electron and proton transfers but a mixture of alternating and consecutive steps. In particular, the first reaction steps will start with double proton transfer followed by an electron transfer, while thereafter, there is another proton transfer and a second electron transfer to give a complex whereby ammonia can split off with a low energetic barrier. The second channel starts with alternating protonation of the two nitrogen atoms, whereafter the initial double proton transfer, electrons and protons are added sequentially to form a hydrazine-bound complex. The latter split off ammonia spontaneously after further protonation. The various reaction channels are analyzed with valence bond and orbital diagrams. We anticipate the nitrogenase enzyme to operate with mixed alternating and consecutive protonation and electron transfer steps.

## Abstract 2

Density functional theory calculations on a μ-dinitrogen bridged diiron complex investigate the reaction mechanisms and possibilities of dinitrogen reduction to two ammonia molecules through alternating and consecutive pathways. The calculations show that multiple possible pathways through mixed alternating and consecutive mechanisms are feasible.

## Introduction

Nitrogen fixation is an essential process on Earth that has made life possible. It is catalyzed in nature by the nitrogenase enzyme that uses protons and electrons to reduce dinitrogen from the atmosphere into ammonia molecules, which acts as the building blocks of amino acids and other natural products.^1–5^ In nitrogenase, the reaction takes place on the Fe_7_MoS_9_C cofactor called FeMoco, which links seven iron atoms, a molybdenum atom, nine sulfur atoms, and a central carbon atom. The exact details on the catalytic cycle of nitrogenase remain controversial and many groups have investigated steps of the catalytic cycle using combined experimental and computational approaches.^6–11^

As there is still no consensus catalytic cycle for nitrogenase and specific details on the individual steps remain controversial, many groups have created biomimetic complexes of the active site of nitrogenase. In particular, biomimetic complexes have been created of metalloenzymes, which generally take a transition metal with a first-coordination sphere analogous to the enzyme and investigate its structure, electronic properties, and reactivity patterns in solution.^12–23^ Many of those studies investigated mononuclear metal–nitrido and metal–imido complexes and their functional properties.^24–37^ In more recent years also biomimetic nitrogenase complexes with diiron centers have been synthesized and investigated.^38–46^

**Scheme 1:**
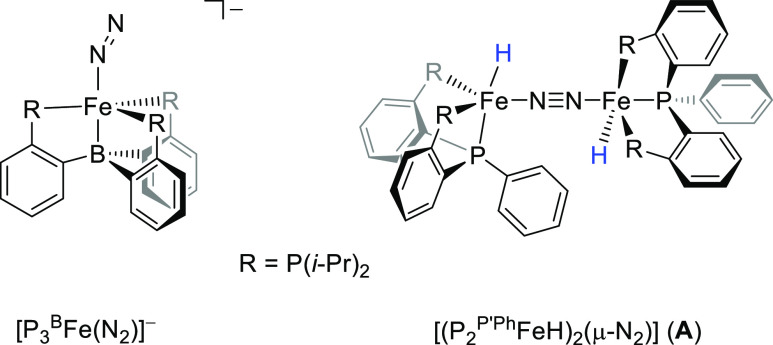
Mononuclear and Dinuclear Nitrogen Reduction Models

One of the first biomimetic complexes with mononuclear iron that was shown to give nitrogenase function was the [P_3_^B^Fe(N_2_)]^−^ system with P_3_^B^ = tris(phosphine)borane (Scheme 1).^27,28^ Using proton and electron donors, the authors were able to trap and characterize various products along the nitrogen reduction pathway. They proposed that an end-on bound iron–dinitrogen complex [P_3_^B^Fe(N_2_)]^−^ would abstract electrons and protons in an alternating fashion. Computational modeling indeed confirmed that the most thermodynamically favorable pathway had alternating electron and proton transfer leading to reduction of the terminal nitrogen atom of iron–dinitrogen to ammonia first.^47,48^ Moreover, proximal nitrogen protonation was found to be unfeasible for the first and second proton abstraction steps. More recently, the group reported a dinitrogen-bridged diiron(I) system with an analogous triphenylphosphine ligand system, namely [(P_2_^P′Ph^FeH)_2_(μ-N_2_)] designated **A** (Scheme 1). The structure was characterized with infrared spectroscopy, crystallography, and ^1^H NMR spectrometry. Addition of protons and electrons to the system leads to the conversion of dinitrogen to ammonia. Details of the mechanism and how it compares with the mononuclear iron system [P_3_^B^Fe(N_2_)]^−^ remain unknown, and it encouraged us to start a comprehensive density functional theory (DFT) study. Our work shows that a variety of reaction channels are feasible including consecutive and alternating protonation and reduction channels of the dinitrogen group.

## Methods

The model of the dinitrogen-bridged dimer complex [(P_2_^P′Ph^FeH)_2_(μ-N_2_)] (designated **A**) was taken from the crystal structure coordinates of ref (45). Hydrogen atoms were added in Gaussview^49^ and subsequently the geometry of the full structure was optimized in the *ORCA* software package.^50^ To test and validate the computational methods, we did a geometry optimization of structure **A** using various DFT methods, namely, with the functionals BP86,^51,52^ B3LYP,^53,54^ PBE0,^55^ TPSS,^56^ and TPSSh^57^ all with the D3BJ dispersion correction included.^58^ Geometry optimizations and analytical frequencies were done for the broken symmetry singlet and triplet spin states with a def2-TZVP basis set on iron and a def2-SVP basis set on the rest of the atoms: basis set BS1.^59^ The def2/J auxiliary basis set was used to enable the RI-J approximation for nonhybrid functionals, and the RIJCOSX approximation was applied for hybrid density functionals. The conductor-type polarized continuum model alongside the universal solvation model together with a dielectric constant mimicking tetrahydrofuran as implicit solvent model in *ORCA* was applied as part of the geometry optimizations and frequencies.^60,61^ Following geometry optimizations and frequency analyses, single point calculations were performed on the optimized structures, utilizing the TPSSh functional and def2-TZVP basis set on all atoms, with free energies calculated using the zero-point energy, thermal, and entropy corrections obtained from the frequency analyses on the optimized geometries. These methods were previously validated for analogous iron complexes and found to reproduce spin-state assignment and experimental free energies of activation to within a few kcal mol^–1^.^62–64^

The ^57^Fe Mössbauer parameters were obtained by calculating the electron densities and electric field gradients around the iron centers in the optimized geometries. In particular, the quadrupole splitting, Δ*E*_Q_, values were obtained directly from the calculation while the calculated ρ value was used to obtain the isomer shift δ according to the equation
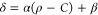
1

In eq 1, α, β, and *C* are constants obtained from a calibration curve against experimental data for the specific combination of density functional method and basis set and taken from the literature.^65^

Thereafter, reaction pathways for various chemical transformations were calculated as follows: Transition states were found by first conducting a constraint geometry scan of the complex with one degree of freedom fixed, i.e., the N–N bond for an N–N cleavage pathway. The highest energy structure from the scans was taken as input for *ORCA*’s “OptTS” transition-state optimization function, augmented by the calculation of a numerical Hessian. The frequency calculation ascertained the identity of the transition state via an accompanying vibrational frequency analysis. All local minima had real frequencies only while the transition states had one imaginary mode for the correct transition. Vibrational frequencies reported here are unscaled values.

Reduction and protonation steps for the free-energy profiles were calculated relative to the redox energy of cobaltocene (Cp_2_Co) and the deprotonation energy of (Et_2_O)_2_H^+^ (formed via protonation of a diethyl ether dimer). We also tested reduction energies against the ferrocene/ferrocenium couple, but the same trends were observed. Redox potentials were calculated as before with respect to SHE by taking the free energy of activation with solvent, entropic and thermal corrections included and subtracting a value of 4.44 eV for the SHE electrode.^66^ Intrinsic bond orbitals (IBOs) were generated using *ORCA*’s “IAOIBO” orbital localization method and imported into the IBOView software for viewing and analysis.^67^

## Results

### Method Validation

**Figure 1:**
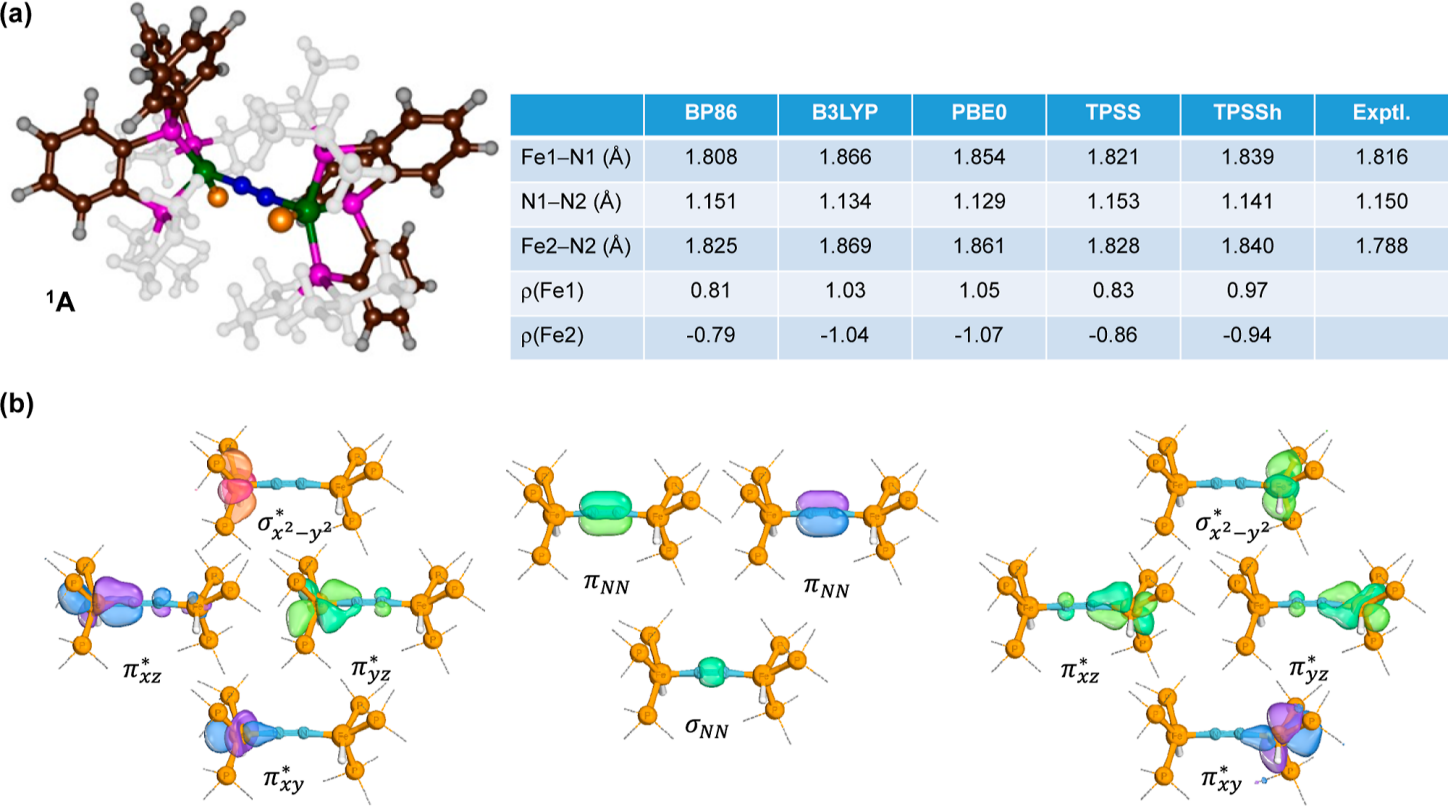
(a) DFT optimized geometries of dinitrogen-bridged diiron complex **A** in the antiferromagnetically coupled singlet spin state. Bond lengths are in Å while iron spin densities (ρ) are in electron units. Experimental data from ref (45). The hydride atoms bound to iron are highlighted in amber. (b) Relevant IBOs of complex **A**.

Before embarking on a mechanistic study of individual proton and electron transfer processes, we validated our methods and models against experimental data. In particular, we compared our optimized geometries with crystal structure coordinates and calculated spectroscopic parameters; see Figure 1. Experimental studies identified **A** as a singlet spin ground state based on the room temperature ^1^H NMR spectrum.^45^ Moreover, Mössbauer spectroscopy characterized the species with an isomer shift δ = 0.15 mm s^–1^ and a quadrupole splitting of Δ*E*_Q_ = 0.80 mm s^–1^. Therefore, a combination of pure and hybrid density functional methods was initially chosen, and a full geometry optimization of structure **A** was performed with focus on its structure and spectroscopic features. In all cases, the metal atoms are in the iron(I) oxidation state with an antiferromagnetically coupled doublet spin state on each iron center with molecular orbital occupation, π_xy_^*^_,Fe1_^2^ π_xz_^*^_,Fe1_^2^ π_yz_^*^_,Fe1_^2^_,Fe1_^1^ π_xy_^*^_,Fe2_^2^ π_xz_^*^_,Fe2_^2^ π_yz_^*^_,Fe2_^2^_,Fe2_^1^. Note that the *z*-axis is chosen along the Fe–N–N–Fe axis of the molecule. The two unpaired electrons in  on either Fe1 or Fe2 are antiferromagnetically coupled in an overall singlet spin state. There are some differences in the optimized geometries; see Figure 1a, as expected from the choice of the density functional method. The dinitrogen bond is found in a narrow window ranging from 1.129 to 1.151 Å and therefore all DFT methods reproduce an experimental distance of 1.150 Å well.^45^ The BP86 and TPSS optimized structures give the best agreement against the crystal structure distances for the Fe1–N1 and N1–N2 bond lengths. All DFT methods overestimate the Fe2–N2 interaction by more than 0.03 Å, although it is not clear why. The various DFT methods, however, give quite different unpaired spin populations for the antiferromagnetically coupled singlet spin state (^1^**A**). Thus, using B3LYP and PBE0 the spin density values are large and well over 1 in magnitude. By contrast, with the BP86 and TPSS methods, a much lesser unpaired spin density of around 0.8 is observed. This will have an effect on the reduction steps in the chemical reaction mechanism. In addition, the spin densities are not exactly the same on each iron center, although they are not different by more than 0.05 units. Nevertheless, this small difference in unpaired spin density may mean that catalysis will be directed to one specific iron atom. Although most of the subsequent studies employed the BP86 functional, tests of selected reaction steps with the PBE0 method were performed and confirmed the general trend and conclusions (Table S13, Supporting Information).

To understand the bond patterns in dinitrogen-bound diiron(I) complex **A**, we analyzed the IBOs. The valence IBOs for key bonding interactions in complex **A** are listed in Figure 1b. As can be seen, three N–N bond orbitals can be identified from the IBOs, namely, one of σ-type (σ_NN_) and two of π-type (π_NN_). As such, the dinitrogen group should be considered as having a dominant triply bonded N–N interaction. This is consistent with the short N–N distance of about 1.15 Å observed in the calculations and only slightly longer than the value of 1.0977 Å for a free N_2_ molecule in the gas phase.^68^ All three N–N bond orbitals have occupation on the nitrogen atoms only, and no density is found on either of the iron atoms.

We also analyzed the iron orbitals, and the sets of IBOs for Fe1 (left-hand-side of Figure 1b) and Fe2 (right-hand-side of Figure 1b) are almost identical and each other’s mirror image. In general, the bond order for both Fe–N interactions has a value below 1. The three π* orbitals on each iron center show a small contribution on the nitrogen atoms, whereby the π_xz_^*^ orbital gives 73% 3d_*xz*_(Fe) and 15% 2p_*x*_(N) character. As such, the π* orbitals in ^1^**A** cannot be considered chemical bonds but are a dative bond between nitrogen and iron where the metal 3d orbitals (3d_*xy*_, 3d_*xz*_, and 3d_*yz*_) interact with the lone pair on nitrogen. The singly occupied orbital on both iron atoms is the  orbital that is perpendicular to the Fe–N axis. Despite the fact that N_2_ does not appear to have significant orbital overlap with the iron centers, we calculate a dissociation energy of Δ*E* = 57.8 kcal mol^–1^ of splitting ^1^**A** into isolated N_2_ and two [P_2_^P′Ph^FeH]^0^ fragments. Consequently, these dative Fe–N bonds have a strength of about 29 kcal mol^–1^ each, which is much lower in energy than a typical covalent Fe–N bond. Therefore, complex ^1^**A** is a stable structure as confirmed by crystallography, even though the dinitrogen–iron interactions from the molecular orbitals appear relatively weak.

To further validate our chemical structures, we calculated the Mössbauer parameters for ^1^**A**. Experimental work^43^ identified an isomer shift δ = 0.15 mm s^–1^ and a quadrupole splitting of Δ*E*_Q_ = 0.80 mm s^–1^. Our BP86 optimized geometry gives calculated values of δ = 0.10 mm s^–1^ and Δ*E*_Q_ = 0.83 mm s^–1^. Consequently, the calculated structures and spectroscopic parameters are in perfect agreement with experimental data and confirm that the computational approach is appropriate for the calculations of these complexes.

An analysis of the UBP86/BS1 calculated infrared frequencies gives Fe–H stretch vibrations at 1798 and 1806 cm^–1^, which compare well with the experimental values of 1734 and 1833 cm^–1^.^43^ Although, the dinitrogen stretch vibration could not be located experimentally, the frequency calculation puts it at 1981 cm^–1^ but with a small IR intensity and some degree of mixing with Fe–H stretch vibrations. The Fe–N bending vibrations are spread out over a large range from 600 to 750 cm^–1^. Subsequently, we re-evaluated the frequency calculations with the iron-bound hydrides replaced by deuterium. H/D replacements of the hydride groups shift the Fe–H vibrations from 1806 (Fe2–H stretch) and 1798 (Fe1–H stretch) cm^–1^ to 1287 and 1282 cm^–1^ for Fe2–D and Fe1–D, respectively. These values are in good quantitative agreement with experimental observation that showed a downshift by 509 and 478 cm^–1^ for the two Fe–H frequencies when hydrogen was replaced by deuterium.^45^

We also calculated the triplet spin state with one unpaired electron on each iron center, as well as the overall septet spin state with a quartet spin on each iron atom. Structurally, the singlet and triplet optimized geometries are very similar (see the Supporting Information) as expected for systems with the same electronic configuration and orbital occupation. The electronic state with a ferromagnetically coupled quartet spin configuration on each iron atom, namely ^7^**A**, was also calculated and is found to be >70 kcal mol^–1^ higher in energy than ^1^**A**. Therefore, the diiron complex with two unpaired electrons is lowest in energy.

### Dinitrogen Reduction Mechanism

Starting from the dinitrogen-bridged diiron complex, i.e., **A**, we first attempted an internal proton transfer from one of the Fe–H groups on the complex. These scans (Supporting Information, Figure S1) gave high-energy pathways with energies Δ*E* > 30 kcal mol^–1^ while a geometry optimization of a structure with the hydride bound to dinitrogen converged back to structure ^1^**A**. Therefore, the hydride groups cannot take part in the dinitrogen reduction reaction, and internal proton transfer was ruled out as a feasible reaction mechanism. Instead, based on previous experimental and computational studies of dinitrogen reduction mechanisms, we explored alternating and consecutive mechanisms, whereby external protons and electrons are added to the system.^42–48^

**Scheme 2:**
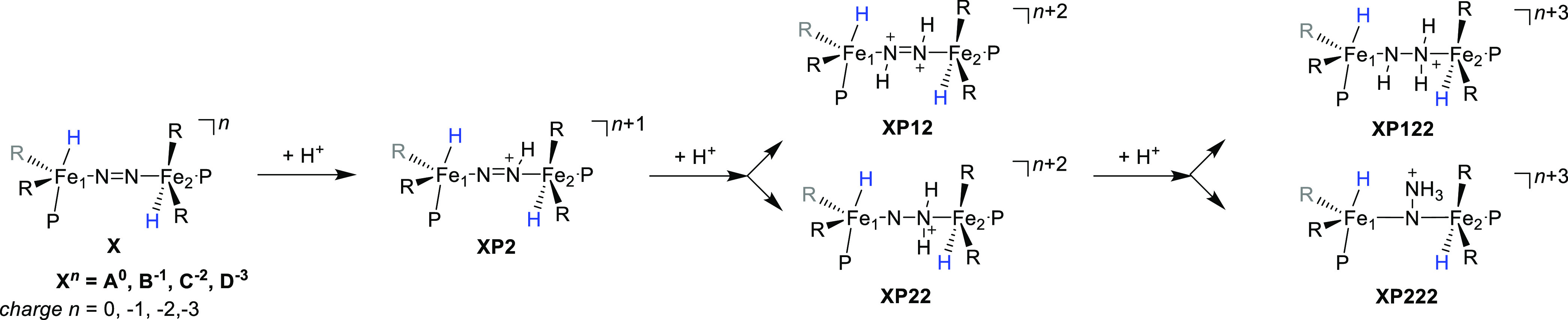
Definition of the Labels of the Structures Investigated Here

Scheme 2 shows the definition of the structures for the possible pathways investigated here for adding the first three electrons and protons to structure **A** in all possible orders. As internal proton transfer appeared to be high in energy, external electrons and protons for dinitrogen reduction at ^1^**A** were considered instead. The electron transfer was calculated with respect to the Cp_2_Co/Cp_2_Co^+^ couple while the proton transfer energy was evaluated with respect to the protonated diethyl ether dimer, (Et_2_O)_2_H^+^, which were the reductant and proton sources used in the experiments of ref (45). In particular, the structures without external electrons are labeled **A**, while addition of one electron gives the **B** structures and a subsequent addition of 1, 2, or 3 electrons gives the **C**, **D**, and **E** systems. The charges are balanced by adding protons to the structures. Thus, we added the first proton to the nitrogen atom adjacent to Fe2 (structure designated **AP2**), which because of symmetry is equal to **AP1** where the proton is located on N1. Thereafter, a second proton was added to either nitrogen 1 or 2 to give the diazene-bridged diiron (**AP12**) or a complex with one of the nitrogen atoms doubly protonated (**AP11** or **AP22**). The third proton transfer then gives protonated diazene (**AP112** or **AP122**) or an N-bridged diiron complex bound to ammonium (**AP222**). The latter structure in all cases is optimized to a geometry that has the NH_3_ group split from the iron atoms, while the other nitrogen atom bridges the two iron atoms. Similar labels for the protonated structures associated with the oxidation states for **B**, **C**, **D**, and **E** structures. We validated our energetics at the UBP86/BS1 level of theory by calculating the redox potential with respect to SHE for the Fc/Fc^+^ and Cp_2_Co/Cp_2_Co^+^ couples first. Values of 0.53 eV for the Fc/Fc^+^ couple and −0.80 eV for the Cp_2_Co/Cp_2_Co^+^ couple were obtained. These values match experimental redox values of 0.4 and −0.9 eV, respectively, excellently.^69^

**Figure 2:**
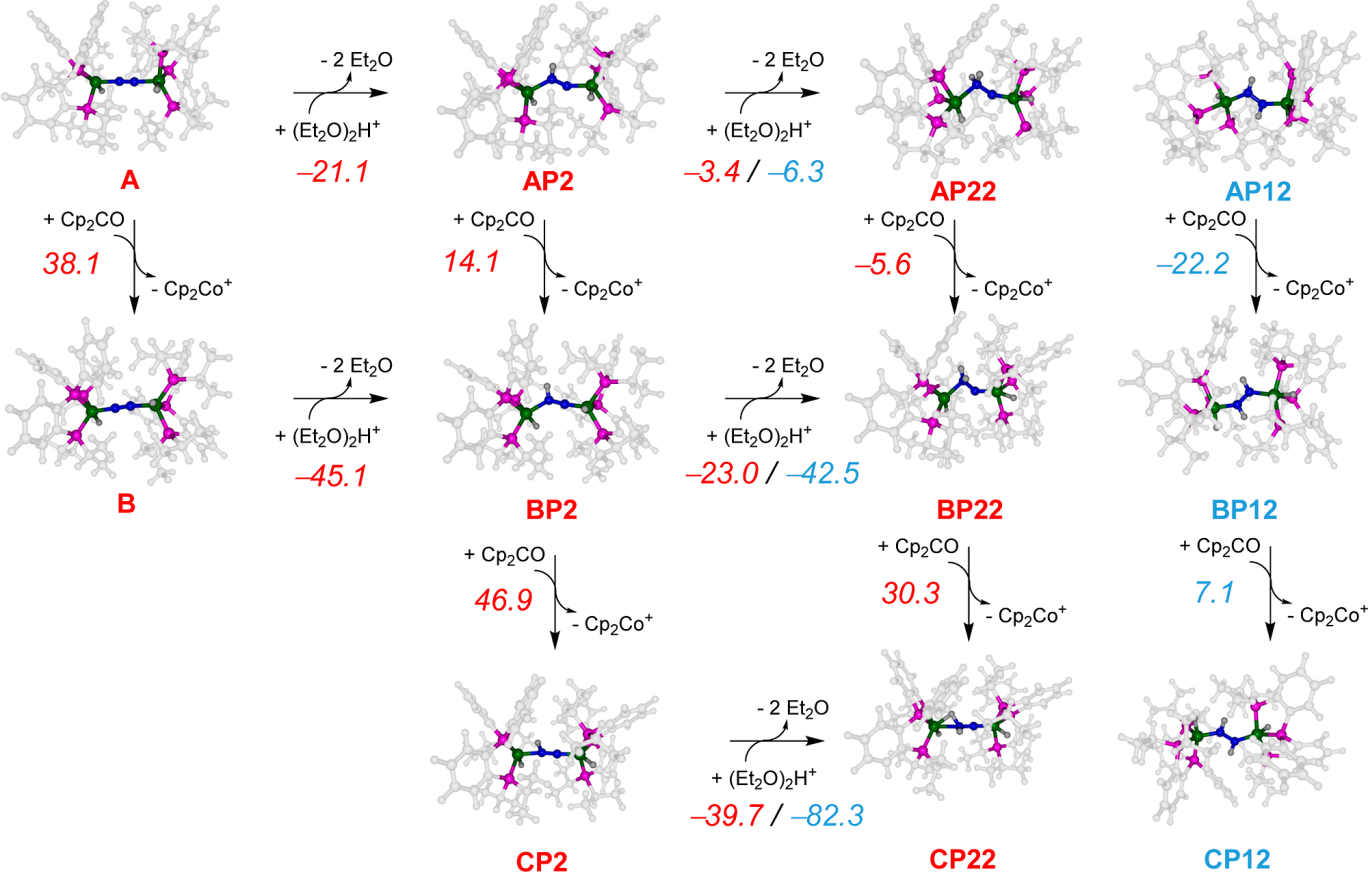
Alternating and consecutive electron and proton transfers to structure **A**. Reaction free energies (Δ*G* at 298 K with zero-point, entropic, thermal, and solvent corrections included in kcal mol^–1^) calculated at the UBP86/BS1//UBP86/BS2 level of theory are shown above each arrow. Proton transfer energies are relative to the protonated diethyl ether dimer and electron transfer energies relative to the cobaltocene/cobaltocenium couple.

Figure 2 shows the thermodynamics of addition of two electrons and two protons consecutively to structure **A**, whereby a horizontal pathway represents a proton transfer while the vertical channels cover single electron transfer steps. These structures are the result of full geometry optimization without constraints. The full matrix of pathways was explored for consecutive and alternating proton and electron transfers. As can be seen from Figure 2, the reduction of **A** by Cp_2_Co is endergonic by Δ*G* = 38.1 kcal mol^–1^, whereas proton transfer from protonated diethyl ether dimer is exergonic by Δ*G* = −21.1 kcal mol^–1^. Therefore, in the absence of heat for the reaction, an initial proton transfer will take place rather than an electron transfer. Of course, in the presence of a heat source or light, as in a photolysis experiment, there may be sufficient heat available to overcome the initial electron transfer barriers and hence will lead to differences in the chemical reaction mechanism. Indeed, experimentally, the reaction was shown to give a higher yield under photolysis conditions in agreement with our thermochemical values. Moreover, under photolysis conditions excited-state structures may be accessible enabling different pathways.^70^

Interestingly, the reduction of the singly protonated species (**AP2**) is still endergonic (Δ*G* = 14.1 kcal mol^–1^), while the second proton transfer from this intermediate is exergonic by −3.4 kcal mol^–1^ to form **AP22** and −6.3 kcal mol^–1^ to form **AP12**. These values imply an equilibrium between **AP2**, **AP22**, and **AP12** along those reaction coordinates. Thermochemically, therefore, the first two proton transfer steps from (Et_2_O)_2_H^+^, i.e., for the steps **A** → **AP2** → **AP22**/**AP12**, will be the preferential pathways over reduction of the complex and are expected to take place in quick succession. Note that the diazene-bridged complex **AP12** is a more stable complex than the one with one nitrogen atom doubly protonated (**AP22**) by Δ*G* = 2.9 kcal mol^–1^. Both doubly protonated species abstract an electron from Cp_2_Co rapidly in an exergonic electron transfer to give **BP22** and **BP12**. The free-energy gap between the singly reduced structures considerably widens after reduction, and **BP12** is more stable than **BP22** by Δ*G* = 19.5 kcal mol^–1^. The thermochemical cycles shown in Figure 2, therefore, indicate that at room temperature, the most likely channel for conversion of **A** is by initial double protonation to form the diazene-bridged diiron complex **AP12**, which is rapidly reduced to **BP12**. The latter can convert to **CP12** after passing a small barrier, while the reduction of **BP22** is strongly endergonic and unlikely to happen without photons. Consequently, the calculations predict a mechanism starting with two successive proton transfers followed by two electron transfers to give doubly protonated species **CP12**.

**Figure 3:**
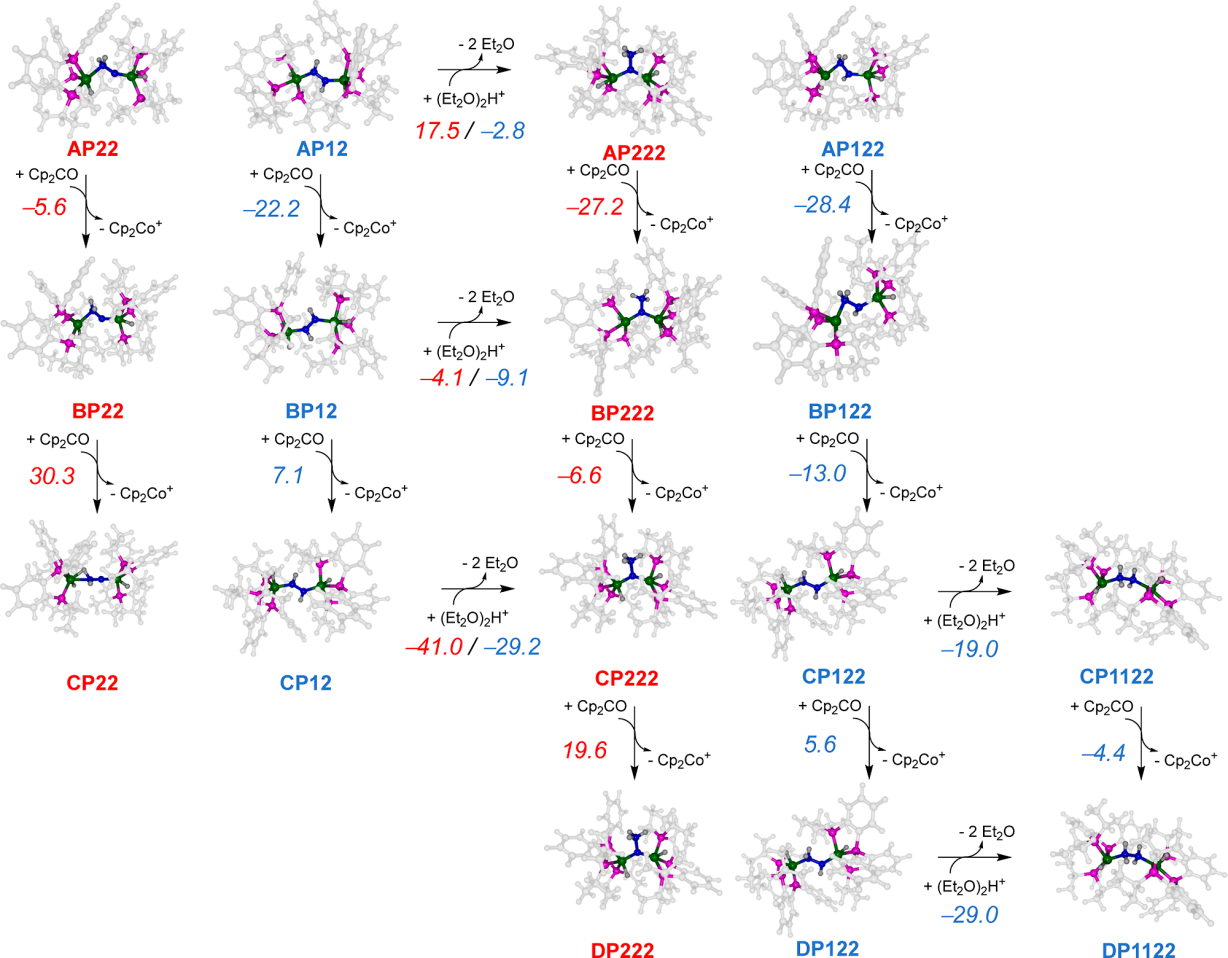
Alternating and consecutive electron and proton transfers starting from the doubly protonated intermediates. Reaction free energies (Δ*G* at 298 K with zero-point, entropic, thermal, and solvent corrections included in kcal mol^–1^) calculated at the UBP86/BS1//UBP86/BS2 level of theory are shown above each arrow. Proton transfer energies are relative to the protonated diethyl ether dimer and electron transfer energies relative to the cobaltocene/cobaltocenium couple.

Next, we explored further reduction and protonation of the diazene- and nitride-based structures **AP12** and **AP22** and the free energies for all possible steps are shown in Figure 3. Protonation of the unreduced species **AP12** and **AP22** to form either **AP122** or **AP222** is Δ*G* = −2.8 and 17.5 kcal mol^–1^, respectively, while electron transfer from Cp_2_Co to **AP12** is more exergonic by at least Δ*G* = 16.6 kcal mol^–1^ over **AP22**. Clearly triple protonation without reduction gives a highly charged complex that requires additional energy to be formed. The most likely pathway from **AP12**, therefore, will be its reduction to **BP12**. Continuing the mechanism from **BP12** will go through protonation to **BP122**, favorable over reduction by Δ*G* = 16.2 kcal mol^–1^. In particular, reduction of **BP12** to form **CP12** is endergonic by Δ*G* = 7.1 kcal mol^–1^, while its protonation releases Δ*G* = −9.1 kcal mol^–1^ to form **BP122**. Both reaction channels via **CP12** and **BP122** come together again due to a strongly exergonic process to form **CP122**. The latter reacts further through an exergonic proton abstraction (by −19.0 kcal mol^–1^) to generate the hydrazine-bridged diiron complex **CP1122**. This species is then reduced to **DP1122** in another exothermic step with a Cp_2_Co of −4.4 kcal mol^–1^. Further protonation of either **CP1122** or **DP1122** leads to the spontaneous release of ammonia from the complexes and their dissociation into two mononuclear iron systems.

Also, the structures **BP22** and **BP222** are in equilibrium with a small free energy along this reaction coordinate. The latter structure has lost the Fe2–N2 interaction and now has one nitrogen atom bridging the two iron atoms, while the ammonium group dangles from the bridging nitrogen atom. The **BP222** structure gives an exergonic electron abstraction from Cp_2_Co of Δ*G* = −6.6 kcal mol^–1^ to form **CP222**. A subsequent electron transfer to form **DP222**, however, is endergonic by 19.6 kcal mol^–1^. Therefore, **CP222** appears to be an end-point in the catalytic mechanism shown in Figures 2 and 3 and either splits off NH_3_ or separates into two mononuclear iron complexes. These possible mechanisms are discussed below. As mentioned above, although the **AP22** formation channel may be competitive with **AP12**, it may produce some **BP22** that reacts further via proton abstraction to give **BP222** and electron transfer to give **CP222**. The latter may be able to split off a NH_3_ molecule prior to further protonation and reduction.

**Figure 4:**
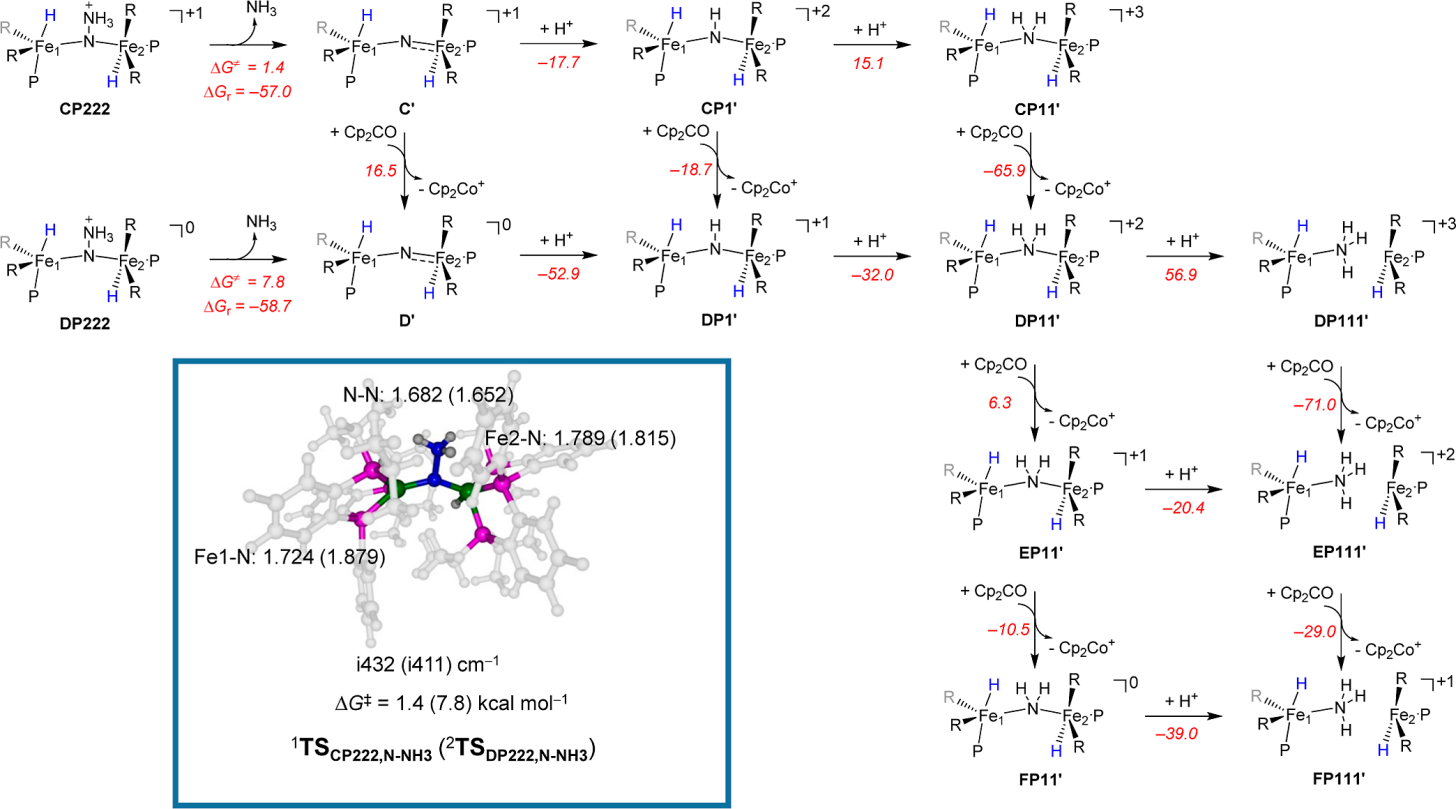
Transition-state structure for the first ammonia release from complexes **CP222** and **DP222** and the subsequent protonation and reduction sequences of the μ-nitrido-bridged diiron complex **C′**. Transition states for NH_3_ release transition states calculated at UBP86/BS1//UBP86/BS2 in Orca are shown with bond lengths in angstroms and the imaginary frequency in cm^–1^. Relative free energies include solvent, entropic, and thermal corrections at 298 K in kcal mol^–1^.

Subsequently, we explored the potential energy landscape for ^1^**CP222** and ^2^**DP222** and located transition-state structures for the N–NH_3_ cleavage pathway for the dissociation of ammonia from the complex, designated as ^1^**TS**_CP222,N-NH_3__ and ^2^**TS**_DP222,N-NH_3__. For structure ^1^**CP222**, we located a small barrier for N–NH_3_ cleavage of Δ*G*^*⧧*^ = 1.4 kcal mol^–1^, namely ^1^**TS**_CP222,N-NH_3__ as displayed in Figure 4. Therefore, upon formation of structure **CP222**, the N–N bond will break spontaneously, and an ammonia molecule will be released. We also characterized a transition state for the further reduced structure, namely **DP222**, and find its Δ*G*^*⧧*^ = 7.8 kcal mol^–1^ above the local minimum. Consequently, both **CP222** and **DP222** rapidly dissociate an ammonia molecule from the complex with small barriers. Regardless of the overall charge of the complex, the N–NH_3_ cleavage transition states look very similar in structure and vibrational frequencies. In particular, both have an imaginary frequency representing the N–N stretch vibration with magnitudes of i432 and i411 cm^–1^. The N–N bond has elongated to 1.682 Å in ^2^**TS**_CP222,N-NH_3__, while it is 1.652 Å in ^1^**TS**_DP222,N-NH_3__. The N–N cleavage in structures **CP222** and **DP222** gives nitrogen-bridged diiron complexes **C′** and **D′**, respectively. The reaction steps for ammonia release are highly exergonic by Δ*G*_r_ = −57 and −59 kcal mol^–1^ as expected from the low barriers for these processes.

We then investigated pathways for reduction and protonation of the μ-nitrido-bridged diiron complexes **C′** and **D′** to form a second molecule of ammonia. The protonation and reduction scheme is shown in Figure 4 as well. Single protonation of **C′** (to form **CP1′**) is exergonic by −17.7 kcal mol^–1^, however, the subsequent protonation to form **CP11′** is endergonic by 15.1 kcal mol^–1^. By contrast, the reduction of **CP1′** is exergonic by −18.7 kcal mol^–1^. Therefore, upon release of ammonia from structure **CP222**, the μ-nitrido-bridged diiron system will be protonated and reduced to form **DP1′** rapidly. From the latter complex, we located an exergonic proton abstraction to form **DP11′** with −32.0 kcal mol^–1^. However, abstraction of a third proton by **DP11′** was found to be highly endergonic by 56.9 kcal mol^–1^ and splits the diiron complex into two mononuclear iron complexes, although its reduction is close to thermoneutral at +6.3 kcal mol^–1^ and forms **EP11′**. Despite the fact that both the protonation and reduction steps from **EP11′** are exergonic, the protonation to form **EP111′** is more favorable by −9.9 kcal mol^–1^. This species will be reduced easily by Cp_2_Co to form complex **FP111′** that splits into two mononuclear iron centers upon release of −29.0 kcal mol^–1^ in free energy. The second formation of a molecule of ammonia is, therefore, done through an alternating pathway of consecutive proton and electron transfer steps. The **FP111′** product complex can react through exchange with N_2_ in an exergonic reaction (Δ*G* = −6.8 kcal mol^–1^) and subsequently for the oxidized reactant complex **A**^+^, eq 2.
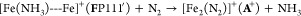
2

The alternative pathway to ligand exchange with N_2_ is further reduction of **FP111′** to **GP111′**, which is endergonic by 13.2 kcal mol^–1^. **GP111′** can dissociate with a further small barrier of Δ*G*^*⧧*^ = 1.6 kcal mol^–1^ to release the second molecule of ammonia.

## Discussion

**Scheme 3:**
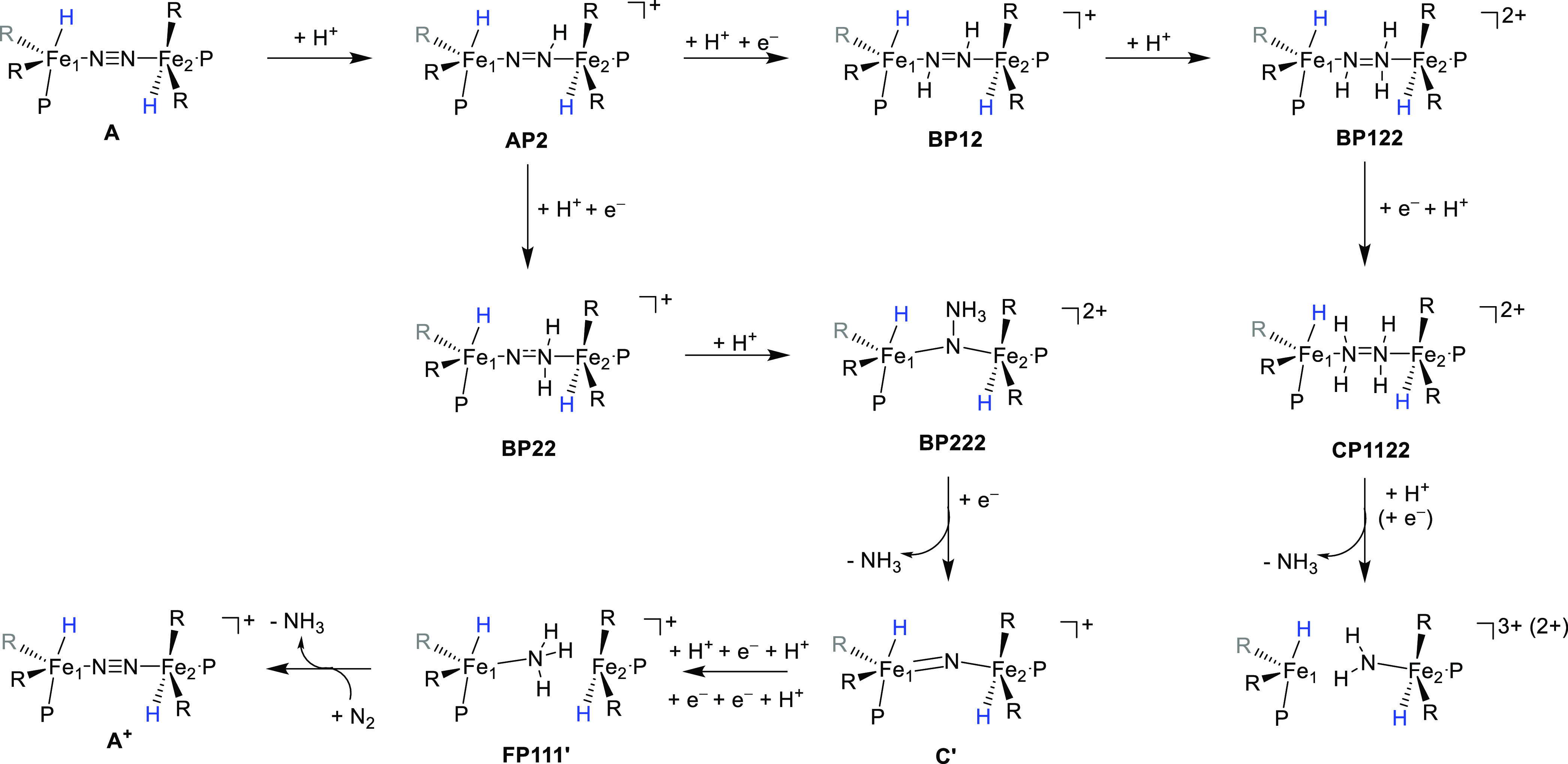
Reaction Mechanism Calculated for the Lowest-Energy Pathways for Conversion of N_2_ into NH_3_ from A by the Addition of Protons and Electrons

In this work, the conversion of a biomimetic μ-dinitrogen-bridged diiron system into two ammonia molecules is studied using external protons and electrons. Scheme 3 shows a summarized scheme of the lowest-energy pathways elucidated for nitrogen fixation from compound **A** by using protons from (Et_2_O)_2_H^+^ and electrons from the cobaltocene reductant. A mixture between consecutive and alternating steps is found, where in the absence of heat the reaction is most likely to start with two protonation steps; however, these two protons can move to the same site and form **AP22** or to the two different nitrogen atoms of N_2_ to form **AP12**. Subsequently, **AP22** is protonated and reduced to form **BP222**, which is a μ-nitrido-bridged diiron system with a dangling NH_3_ group bound to the nitrido atom. Single or double reduction of **BP222** leads to spontaneous release of an ammonia molecule and the formation of the μ-nitrido-bridged diiron complex **C′** or **D′**. This species can be activated further through consecutive proton, electron, proton, electron, electron, and proton transfers to form two mononuclear iron centers, one bearing an NH_3_ group: **FP111’**. Interestingly, the release of ammonia from **FP111′** is high in energy, but instead, a favorable sequential ligand exchange pathway was found for the exchange of NH_3_ by N_2_ to form the oxidized structure **A**^+^. The alternative pathway starts with the alternating protonation of the two nitrogen atoms of N_2_ in **A** to form **AP12**. The latter is reduced and protonated to give **BP122**, which is further reduced and protonated to form **CP1122**, which is the μ-hydrazine-bridged diiron structure. Upon protonation it can dissociate an ammonia molecule with a small reaction barrier. Alternatively, **CP1122** is protonated and reduced and spontaneously releases a molecule of ammonia. The release of an ammonia molecule from the hydrazine-bridged structure, however, splits the diiron complex into two mononuclear iron sites, one of which will hold an NH_2_ ligand. The latter upon protonation will release the second ammonia molecule in the process.

**Scheme 4:**
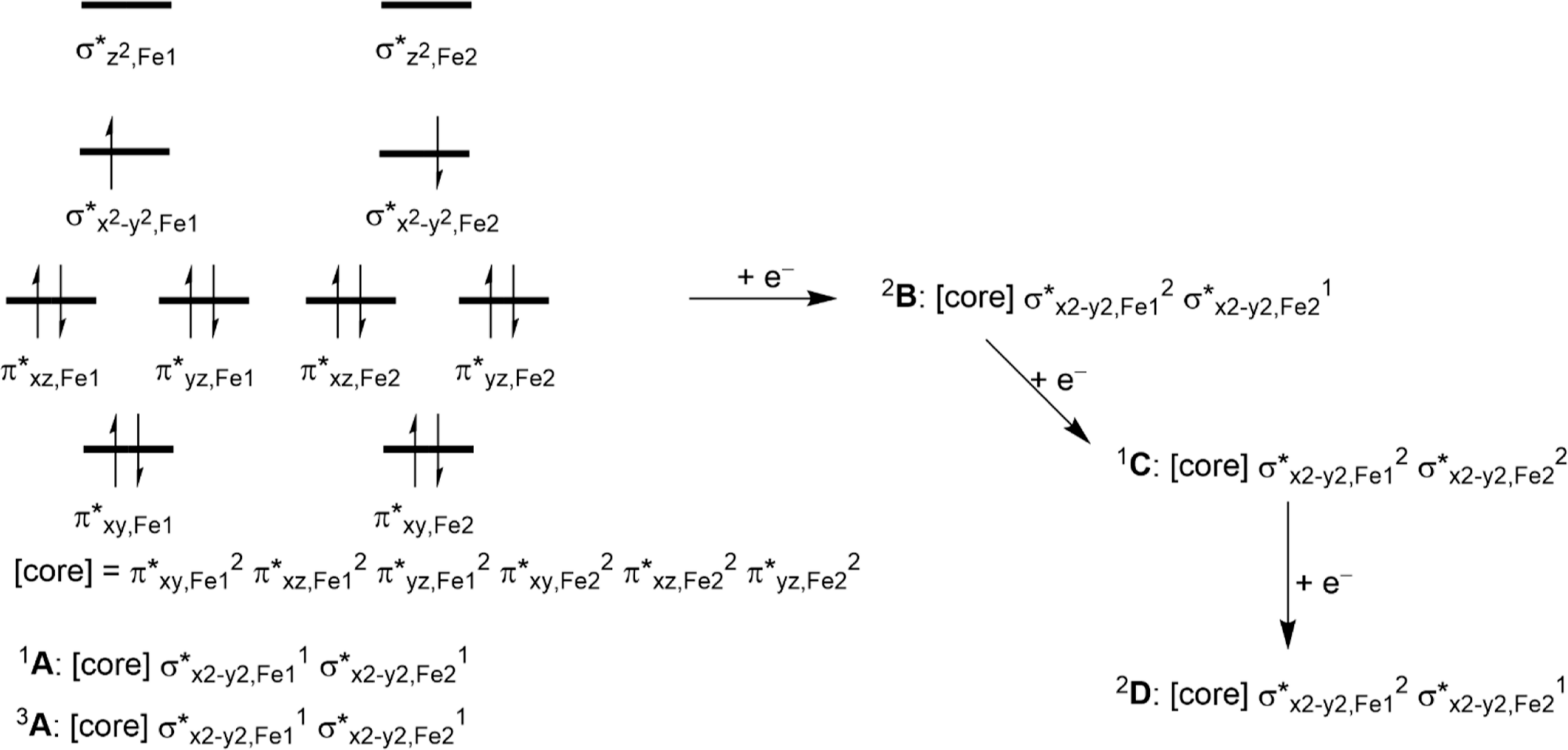
Orbital Occupation of Structure A and Reduced Species B, C, and D

To further understand the reaction pathways and the chemical reaction obtained, we performed a detailed analysis of the reactant structure and its electronic configuration. In particular, in Scheme 4 we show the electron and proton transfer processes of converting the **A**-type structures into **D**-type structures. Thus, in **A** as well as in **AP2**, **AP12**, **AP22**, **AP122**, and **AP222**, both metal atoms are in a formal iron(I) oxidation state with orbitals labeled based on the 3d contribution of the iron atom, where the charge and spin on each metal are approximately the same. Lowest in energy are the antibonding interactions of the metal 3d_*xy*_, 3d_*xz*_, and 3d_*yz*_ atom orbitals on Fe1/Fe2 with first-coordination sphere ligands: π_xy_^*^, π_xz_^*^, and π_yz_^*^. These core orbitals are doubly occupied in all complexes discussed here. The valence orbitals of complex **A** are the two  orbitals for the antibonding interactions of the metal with its ligands, and both of these are singly occupied and ferromagnetically coupled into an overall triplet spin state or antiferromagnetically coupled into an open-shell singlet spin state.

Upon reduction of the **A**-type structures, one electron is added to the metal d-system, which pairs up with one of the  electrons in an overall doublet spin state for the **B**-type structures. For structure **B** also, a quartet spin state was tested with _,Fe1_^1^_,Fe1_^1^_,Fe2_^1^ configuration but found to be higher in energy by at least 10.7 kcal mol^–1^. Reduction of the **B**-structures gives the **C**-structures a closed-shell singlet spin state with _,Fe1_^2^_,Fe2_^2^ occupation. Further reduction of structure **C** starts filling the virtual  orbital along the Fe–N interaction. This is generally a high-energy pathway and, in several cases, leads to dissociation of the complex into fragments. Note as well that in many structures, there is partial electron transfer from iron to nitrogen.

**Figure 5:**
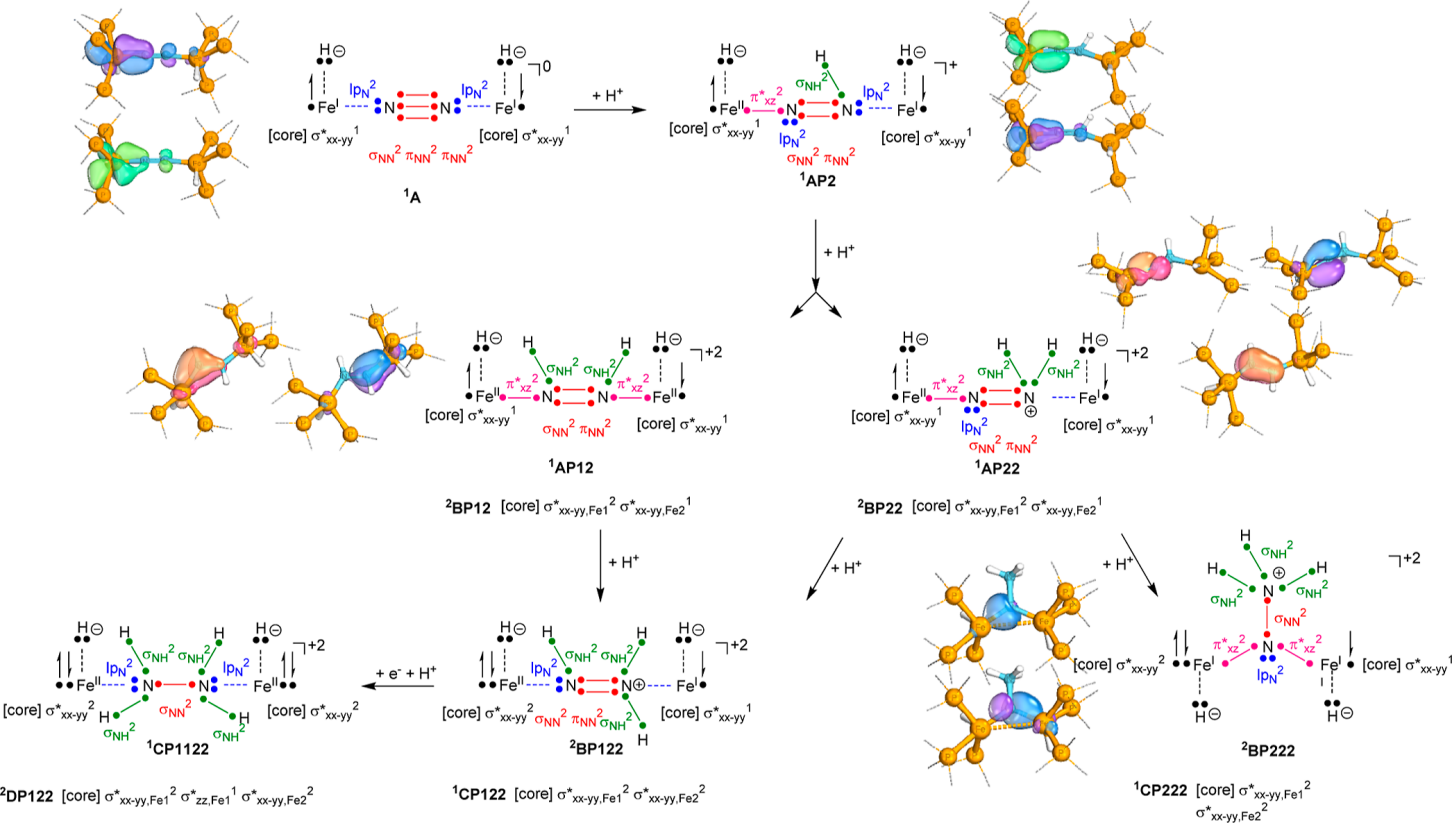
VB description of bonding patterns in various intermediates. An electron is represented with a dot, and a line separating two dots is a doubly occupied bonding orbital. Key IBO orbitals for several structures are shown as well.

To explain the electron transfer processes further, we display the electron occupation and orbital interactions of the lowest-energy structures for the mechanism from ^1^**A** via electron and proton transfer to give ^1^**CP1122** in Figure 5. In particular, to understand the bonding patterns in the various local minima after electron transfer and/or proton transfer, we devised a valence bond diagram that highlights the valence molecular orbitals in each structure. These diagrams have been used before to explain electron transfer pathways during reaction mechanisms.^71–73^ Thus, the reactant species ^1^**A** has a triple bonded N_2_ group with orbital occupation of σ_NN_^2^ π_NN_^2^ π_NN_^2^, where the atomic lone-pair orbitals (lp_N_) interact with the two iron atoms. Both iron atoms are in the iron(I) oxidation state with orbital occupation π_xy_^*2^ π_xz_^*2^ π_yz_^*2^ in an antiferromagnetically coupled overall singlet spin state. Two IBOs for the π_xz_^*^ and π_yz_^*^ orbitals on Fe1 show that they are dominantly located on the iron atom with little involvement from the dinitrogen group. Consequently, they are shown as a dative bond in VB with the nitrogen lone pair interacting weakly with iron. Upon protonation of ^1^**A** to ^1^**AP2**, the electronic configuration does not change, and the proton binds to nitrogen and replaces one of the π-bonds (π_NN_ orbital) with an N–H orbital (σ_NH_). The loss of the triple bond, however, triggers an electron transfer from Fe1 to N1 and makes the π_xz_^*^ orbital a Fe–N bonding orbital; hence, we draw the π_xz_^*2^ orbital as a bond between Fe and N in pink in Figure 5 for ^1^**AP2**. As a consequence of this Fe–N bond formation, the iron is oxidized to iron(II) in ^1^**AP2**. Indeed, in the optimized geometry of ^1^**AP2** the Fe1–N1 distance has shortened to 1.732 Å, while the N–N bond has elongated to 1.224 Å, in agreement with the VB bond assignment. The IBOs for the Fe1–N1 interaction also support the bond formation. The subsequent protonation on the other nitrogen atom to form ^1^**AP12** displaces one of the lone-pair orbitals on nitrogen and gives another N–H orbital (σ_NH_). In this structure, however, the dinitrogen bond is not a pure double bond but is mixed with the lone-pair orbital and some contribution on iron. In particular, the IBOs of ^1^**AP12** give significant Fe–N bond configuration with π-type overlap. Nevertheless, the orbital occupation remains the same for **A**, **AP2**, and **AP12** with unpaired electrons in the  orbitals. Geometrically, the N–N bond in ^1^**AP12** is short (1.340 Å) and implicates significant double-bond character. Interestingly, both Fe–N bonds in ^1^**AP12** have elongated considerably as compared to those in ^1^**AP2** to 1.769 and 1.854 Å.

Protonation on the same nitrogen atom to form ^1^**AP22**, however, retains a clear double bond between two nitrogen atoms as evidenced by the IBO shown in Figure 5 (right-hand-side). At the same time, electron donation from Fe1 to N1 happens where the π_xz_^*^ orbital becomes a bonding orbital. Indeed, structure ^1^**AP22** shows considerable bond shortage for the Fe1–N1 bond to 1.644 Å and an almost symmetric Fe–N bond orbital for the interaction is seen in the IBO analysis. As there is considerable charge built up on the HNNH group in ^1^**AP12** not surprisingly, addition of a third proton is endothermic. Therefore, an electron transfer is needed to lower the overall charge on the HNNH group and to make further protonation steps possible. Reduction of the complex adds a second electron into the  molecular orbital of Fe1 and enables proton abstraction to form ^2^**BP122**. This complex is also formed from the transfer of a proton to N1 in ^2^**BP22**. The ^2^**BP122** structure has donated two electrons from iron into the lone-pair orbital of nitrogen and retains the N=N double bond. Geometrically, the N–N bond is long (1.427 Å) and so are the Fe–N interactions (1.909 and 1.971 Å). The formation of a hydrazine-bound complex is achieved by the reduction of ^2^**BP122** to form ^1^**CP122** and its subsequent protonation to give ^1^**CP1122**. The proton transfer to ^1^**CP122** breaks the dinitrogen double bond, and at the same time, an electron pair is donated from Fe2 to N2. This results in a hydrazine-bound diiron(II) complex, where no significant bonding orbitals between metal and nitrogen atoms are seen. Hence, both structures ^1^**A** and ^1^**CP1122** have a diiron with a neutral molecule wedged between the metal atoms and show no significant bonding interactions. The chemical structures confirm the assignment and give Fe1–N1, N1–N2, and N2–Fe2 distances of 1.981, 1.485, and 1.998 Å, respectively.

**Figure 6:**
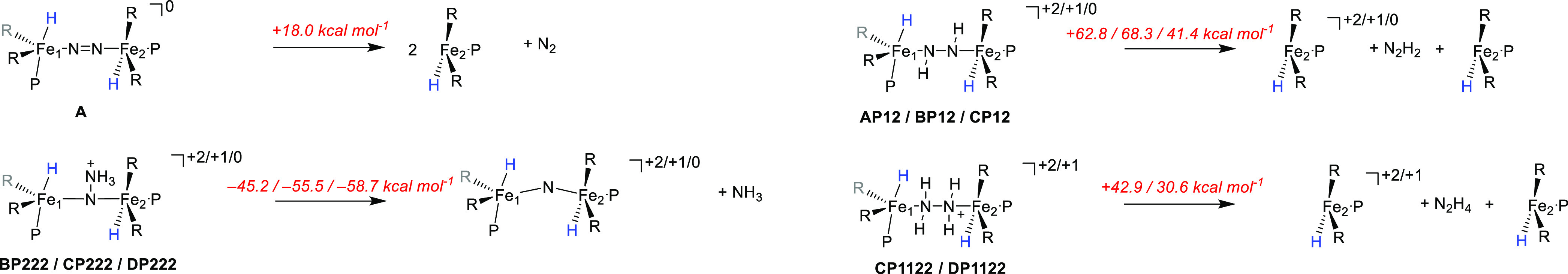
Fragmentation energies of selected intermediates along the dinitrogen activation pathway on ^1^**A**. Energies represent Δ*G* values (in kcal mol^–1^) calculated at the UBP86/BS1//UBP86//BS2 level of theory at 298 K with thermal, zero-point, and entropic corrections included.

To find out whether these weakly bound diiron complexes with N_2_H_*x*_ (*x* = 0, 2, 4) bound can release N_2_, N_2_H_2_, or N_2_H_4_, we calculated the fragmentation energies of several complexes, see Figure 6. As can be seen from Figure 6, the dinitrogen release from ^1^**A** is endergonic by Δ*G* = +18.0 kcal mol^–1^, hence dinitrogen is strongly bound, and we do not expect it to dissociate in a significant amount. Similarly, the N_2_H_2_ dissociation from ^1^**AP12**, ^2^**BP12**, and ^1^**CP12** is endergonic by Δ*G* = 62.8, 68.3, and 41.4 kcal mol^–1^, respectively, which implies that these complexes are highly stable and unlikely to fragment hydrazine at room temperature. Also, release of N_2_H_4_ from either **CP1122** or **DP1122** is endergonic by a large amount; namely, these dissociation steps were calculated at Δ*G* = 42.9 and 30.6 kcal mol^–1^, respectively. Consequently, we do not expect the reaction to give significant amounts of N_2_H_2_ or N_2_H_4_ byproducts during the reduction and protonation processes from **A**. Not surprisingly, the terminal ammonia structures ^2^**BP222**, ^1^**CP222**, and ^2^**DP222** give highly exothermic reaction free energies for ammonia release from these complexes at Δ*G* = −45.2, −55.5, and −58.7 kcal mol^–1^, respectively. As such, these complexes are likely to expel ammonia prior to further reduction and/or protonation processes. Indeed, experimental studies of N_2_ activation by diiron complexes observed the formation of μ-nitrido-bridged diiron complexes.^74^

## Conclusions

In this work, a computational study is presented on a novel biomimetic diiron complex that has been proposed to convert dinitrogen into two ammonia molecules with the aid of external protons and electrons. The work was validated against spectroscopic and crystallographic data from the literature, and excellent agreement between theory and experiment was found. Subsequently, energetic pathways were calculated for reaction energies for proton transfer from the protonated diethyl ether dimer as well as electron transfer from cobaltocene. Pathways for possible mixed consecutive and alternating proton and electron transfer were calculated and analyzed. Specifically, two possible reaction channels are found that both start from structure **A** and lead to either one site protonated sequentially or the alternating of proton transfer pathways. Nevertheless, both pathways give low-energy and exergonic reaction channels, leading to ammonia that splits the diiron system into mononuclear iron complexes. The calculations and their results are analyzed with valence bond and molecular orbital approaches. The work has relevance to the nitrogenase enzyme catalytic cycle, and we anticipate similar mechanisms as proposed here with mixed alternating and sequential addition of protons and electrons, where multiple channels lead to ammonia expulsion from the reaction complex.

## Supporting Information Available

Raw data including absolute energies, relative energies, group charges, group spin densities, and Cartesian coordinates of optimized geometries (PDF)
Diiron coordinates (ZIP)

## Abbreviations

BDE: bond dissociation energy
CPCM: conductor-type polarized continuum model
DFT: density functional theory
IBO: intrinsic bond orbital
SCRF: self-consistent reaction field
ZPE: zero-point energy
