# Supplementary material for: Mechanism of Nitrogen Reduction to Ammonia in a Diiron Model of Nitrogenase
Source: Inorg Chem. 2023 Aug 31;62(36):14715–26. doi: 10.1021/acs.inorgchem.3c02089 (PMC10498488; doi:10.1021/acs.inorgchem.3c02089)
Supplement: Supplementary file 1 — ic3c02089_si_001.pdf [file ic3c02089_si_001.pdf]

Supporting Information

Mechanism of Nitrogen Reduction to Ammonia in a Diiron Model of Nitrogenase

Maxim Barchenko,[§#] Patrick J. O'Malley,[#] and Sam P. de Visser*[§⊥]

[§] Manchester Institute of Biotechnology, The University of Manchester, 131 Princess Street, Manchester M1 7DN, United Kingdom

[#] Department of Chemistry, The University of Manchester, Oxford Road, Manchester M13 9PL, United Kingdom

[⊥] Department of Chemical Engineering, The University of Manchester, Oxford Road, Manchester M13 9PL, United Kingdom

Email: sam.devisser@manchester.ac.uk

Table S1 – Comparison of key bond lengths (in Å) and Mulliken spin populations of the reactant structure A as optimized with the following DFT approaches: BP86, TPSS, TPSSh, B3LYP and PBE0. Also shown are the structural coordinates obtained from the available experimentally obtained crystallographic data.[a] Geometry optimizations were performed for the broken symmetry singlet spin state.[b]

Method	Fe1 Spin	Fe2 Spin	N-N Bond	N-Fe1 Bond	N-Fe2 Bond	Fe1-P Bond Avg	Fe2-P Bond Avg
**Expt. [a]**	-	-	1.150	1.816	1.788	2.152	2.162
**BP86**	0.814	-0.785	1.151	1.808	1.825	2.172	2.154
**TPSS**	-0.862	0.826	1.153	1.821	1.828	2.186	2.176
**TPSSh**	0.966	-0.939	1.141	1.839	1.840	2.182	2.190
**B3LYP**	-1.043	1.035	1.134	1.866	1.869	2.205	2.226
**PBE0**	-1.071	1.051	1.129	1.854	1.861	2.185	2.198

[a] Data taken from: Buscagan, T. M.; Oyala, P. H.; Peters, J. C. N_2-to-NH_3 Conversion by a Triphos–Iron Catalyst and Enhanced Turnover under Photolysis. *Angew. Chem. Int. Ed*. **2017**, *56*, 6921-6926. [b] Bond lengths in Å.

Table S2 – Absolute, Zero Point, and Gibbs Free Energies (in au) of BP86/BS1 optimized geometries calculated along the alternating reaction mechanism pathway.

Structure	E [au]	ZPE [au]	G [au]
^{1}A	-7021.009232	1.260665	-7019.843802
^{1}AP2	-7021.464959	1.273789	-7020.289557
^{1}AP12	-7021.891650	1.288513	-7020.700726
^{3}AP122	-7022.296040	1.297424	-7021.096197
^{2}B	-7021.098885	1.257735	-7019.939120
^{2}BP2	-7021.575183	1.271498	-7020.402562
^{2}BP12	-7022.056740	1.286289	-7020.866825
^{2}BP122	-7022.489234	1.302726	-7021.282014
^{1}CP2	-7021.652111	1.274140	-7020.474439
^{1}CP12	-7022.180656	1.284217	-7020.993845
^{1}CP122	-7022.638664	1.300932	-7021.433484
^{1}CP1122	-7023.084118	1.315799	-7021.864692
^{2}DP122	-7022.755936	1.295994	-7021.557851
^{2}DP1122	-7023.222426	1.311453	-7022.008495
^{1}EP1122	-7023.341892	1.306838	-7022.131262
^{2}EP11222(H)	-3568.006587	0.691923	-3567.378809
^{2}EP11222(H)^{TS}	-3568.005478	0.686963	-3567.383774
^{1}EP11’	-6967.340307	1.278834	-6966.157911
^{2}EP11’(H)	-3511.539028	0.649424	-3510.955873
^{2}EP111’(H)	-3511.982822	0.664870	-3511.382209
^{2}FP11’	-6967.479275	1.282956	-6966.291157
^{1}FP11’(H)	-3511.678371	0.649658	-3511.092115
^{1}FP111’(H)	-3512.162027	0.661635	-3511.563970
^{2}GP11’(H)	-3511.735749	0.643536	-3511.156311
^{2}GP111’(H)	-3512.272727	0.659833	-3511.677445

Table S3 – Mulliken Spin Populations present on the Fe, N and P atoms of the BP86/BS1 optimized geometries calculated for the alternating pathway. All atoms are numbered according to their appearance in the coordinates, with the protons in structures P1-P3 being ligated to Fe1, and the protons for P4-P6 attached to Fe2.

Structure	Fe1	Fe2	N1	N2	P1	P2	P3	P4	P5	P6
^{1}A	0.81	-0.77	-0.04	0.04	-0.01	0.02	0.01	0.02	-0.06	-0.03
^{1}AP2	0	0	0	0	0	0	0	0	0	0
^{1}AP12	-0.70	0.74	-0.06	0.01	0.02	0	0	-0.02	0.01	-0.01
^{3}AP122	0.98	0.95	0	0.04	-0.05	0.02	0.03	-0.01	0	0
^{2}B	0.55	0.58	-0.03	-0.07	0.02	-0.02	-0.02	-0.02	0.03	0.03
^{2}BP2	0.47	0.54	0.12	0.04	0.06	0	0	0.02	0.05	0.01
^{2}BP12	-0.03	1.10	-0.07	-0.01	0	-0.01	0	-0.03	0.02	0.02
^{2}BP122	-0.06	1.08	-0.05	-0.03	0	0	0	0	0.01	0.03
^{1}CP2	0	0	0	0	0	0	0	0	0	0
^{1}CP12	0	0	0	0	0	0	0	0	0	0
^{1}CP122	0	0	0	0	0	0	0	0	0	0
^{1}CP1122	0	0	0	0	0	0	0	0	0	0
^{2}DP122	0	0.99	0	-0.02	0	0	0	0.03	0.06	0.04
^{2}DP1122	0.03	0.90	0	-0.01	0.01	0	0	-0.02	0.07	0.02
^{1}EP1122	0.83	-0.91	-0.02	0.02	0.08	0	0.01	0.02	-0.07	-0.03
^{2}EP11222(H)	0.96	-	-0.02	0	-0.02	0.07	0.03	-	-	-
^{2}EP11222(H)^{TS}	0.97	-	-0.03	-0.02	-0.02	0.07	0.03	-	-	-
^{1}EP11’	0	0	0	-	0	0	0	0	0	0
^{2}EP11’(H)	1.00	-	-0.08	-	-0.04	0.04	0.04	-	-	-
^{2}EP111’(H)	0.98	-	-0.02	-	-0.04	0.04	0.05	-	-	-
^{2}FP11’	0	0.95	-0.02	-	0	0	0	0.06	0.03	-0.03
^{1}FP11’(H)	0	-	0	-	0	0	0	-	-	-
^{1}FP111’(H)	0	-	0	-	0	0	0	-	-	-
^{2}GP11’(H)	0.57	-	0.08	-	0.08	0	0	-	-	-
^{2}GP111’(H)	0.86	-	-0.01	-	0.1	0	0	-	-	-

Table S4 – Mulliken Atomic Charges present on the Fe, N and P atoms of the BP86/BS1 optimized geometries calculated for the alternating pathway. All atoms are numbered according to their appearance in the coordinates, with the protons in structures P1-P3 being ligated to Fe1, and the protons for P4-P6 attached to Fe2.

Structure	Fe1	Fe2	N1	N2	P1	P2	P3	P4	P5	P6
^{1}A	-0.50	-0.53	-0.10	-0.14	0.36	0.26	0.24	0.40	0.26	0.25
^{1}AP2	-0.43	-0.64	-0.22	-0.10	0.37	0.23	0.24	0.32	0.23	0.43
^{1}AP12	-0.39	-0.52	-0.18	-0.14	0.37	0.16	0.20	0.39	0.19	0.26
^{3}AP122	-0.41	-0.47	-0.35	-0.02	0.41	0.19	0.16	0.36	0.22	0.25
^{2}B	-0.41	-0.55	-0.15	-0.17	0.32	0.25	0.24	0.39	0.24	0.25
^{2}BP2	-0.44	-0.65	-0.20	-0.12	0.37	0.26	0.25	0.41	0.25	0.25
^{2}BP12	-0.44	-0.51	-0.18	-0.15	0.16	0.36	0.20	0.39	0.21	0.22
^{2}BP122	-0.47	-0.43	-0.30	-0.03	0.38	0.23	0.17	0.37	0.21	0.23
^{1}CP2	-0.55	-0.60	-0.15	-0.13	0.35	0.23	0.23	0.40	0.23	0.23
^{1}CP12	-0.44	-0.60	-0.22	-0.20	0.36	0.18	0.21	0.38	0.26	0.24
^{1}CP122	-0.47	-0.80	-0.30	-0.05	0.36	0.18	0.22	0.36	0.25	0.36
^{1}CP1122	-0.48	-0.83	-0.10	-0.04	0.38	0.14	0.20	0.33	0.31	0.22
^{2}DP122	-0.45	-0.63	-0.29	-0.07	0.35	0.20	0.21	0.35	0.22	0.20
^{2}DP1122	-0.45	-0.67	-0.10	-0.08	0.38	0.15	0.20	0.35	0.23	0.18
^{1}EP1122	-0.66	-0.66	-0.06	-0.09	0.32	0.24	0.22	0.39	0.22	0.20
^{2}EP11222(H)	-0.63	-	-0.15	0.15	0.31	0.23	0.21	-	-	-
^{2}EP11222(H)^{TS}	-0.61	-	-0.16	0.08	0.31	0.24	0.21	-	-	-
^{1}EP11’	-0.65	-0.72	-0.24	-	0.42	0.20	0.24	0.37	0.22	0.34
^{2}EP11’(H)	-0.41	-	-0.27	-	0.35	0.20	0.20	-	-	-
^{2}EP111’(H)	-0.50	-	-0.08	-	0.41	0.19	0.21	-	-	-
^{2}FP11’	-0.61	-0.44	-0.15	-	0.42	0.16	0.19	0.30	0.25	0.27
^{1}FP11’(H)	-0.47	-	-0.26	-	0.34	0.25	0.22	-	-	-
^{1}FP111’(H)	-0.46	-	-0.10	-	0.38	0.20	0.20	-	-	-
^{2}GP11’(H)	-0.50	-	-0.32	-	0.33	0.26	0.26	-	-	-
^{2}GP111’(H)	-0.53	-	-0.11	-	0.32	0.25	0.24	-	-	-

Table S5 – Absolute, Zero Point, and Gibbs Free Energies (in au) of BP86/BS1 optimized geometries calculated along the non-alternating reaction mechanism pathway.

Structure	E [au]	ZPE [au]	G [au]
^{1}A	-7021.009232	1.260665	-7019.843802
^{1}AP2	-7021.464959	1.273789	-7020.289557
^{1}AP22	-7021.897055	1.293975	-7020.697169
^{1}AP222	-7022.292848	1.301474	-7021.086980
^{2}B	-7021.098885	1.257735	-7019.939120
^{2}BP2	-7021.575183	1.271498	-7020.402562
^{2}BP22	-7022.033518	1.289311	-7020.839662
^{1}BP22(H)	-3566.260632	0.655091	-3565.670523
^{2}BP222	-7022.456391	1.297013	-7021.257315
^{2}B’	-6965.997166	1.262945	-6964.827584
^{1}BP222(H)	-3566.655627	0.670460	-3566.048511
^{1}CP2	-7021.652111	1.274140	-7020.474439
^{1}CP22	-7022.129072	1.289373	-7020.935665
^{2}CP22(H)	-3566.357312	0.651496	-3565.770439
^{1}CP222	-7022.606558	1.296150	-7021.407565
^{1}CP222^{TS}	-7022.617603	1.300666	-7021.410218
^{1}C’	-6966.162005	1.265720	-6964.988832
^{1}CP1’	-6966.592543	1.271764	-6965.415674
^{1}CP11’	-6966.993151	1.293933	-6965.789693
^{2}CP222(H)	-3566.805143	0.665821	-3566.204448
^{2}DP222	-7022.695353	1.290500	-7021.503866
^{2}DP222^{TS}	-7022.694254	1.295712	-7021.493820
^{2}D’	-6966.261343	1.259810	-6965.094778
^{1}DP222(H)	-3566.914073	0.665159	-3566.312932
^{1}DP222(H)^{TS}	-3566.913735	0.661738	-3566.316937
^{2}DP1’	-6966.739127	1.266150	-6965.569912
^{2}DP11’	-6967.194956	1.281438	-6966.010891
^{1}D’(H)	-3510.429217	0.627479	-3509.865217
^{1}DP1’(H)	-3510.922004	0.640540	-3510.344575
^{1}DP11’(H)	-3511.359052	0.653505	-3510.767754
^{1}DP111’(H)	-3511.746349	0.666214	-3511.140784
^{1}EP1’	-6966.851931	1.270941	-6965.673753
^{1}EP11’	-6967.337800	1.283632	-6966.148768
^{2}E’(H)	-3510.488565	0.621130	-3509.932282
^{2}EP1’(H)	-3511.036689	0.635665	-3510.466010
^{2}EP11’(H)	-3511.539028	0.649424	-3510.955873
^{2}EP111’(H)	-3511.982822	0.664870	-3511.382209
^{1}FP1’(H)	-3511.105417	0.634143	-3510.534689
^{2}FP11’	-6967.479275	1.282956	-6966.291157
^{1}FP11’(H)	-3511.678371	0.649658	-3511.092115
^{1}FP111’(H)	-3512.162027	0.661635	-3511.563970
^{2}GP11’(H)	-3511.735749	0.643536	-3511.156311
^{2}GP111’(H)	-3512.272727	0.659833	-3511.677445

Table S6 - Mulliken Spin Populations present on the Fe, N and P atoms of the BP86/BS1 optimized geometries calculated for the non-alternating pathway. All atoms are numbered according to their appearance in the coordinates, with the protons in structures P1-P3 being ligated to Fe1, and the protons for P4-P6 attached to Fe2.

Structure	Fe1	Fe2	N1	N2	P1	P2	P3	P4	P5	P6
^{1}A	0.81	-0.77	-0.04	0.04	-0.01	0.02	0.01	0.02	-0.06	-0.03
^{1}AP2	0	0	0	0	0	0	0	0	0	0
^{1}AP22	0	0	0	0	0	0	0	0	0	0
^{1}AP222	0	0	0	0	0	0	0	0	0	0
^{2}B	0.55	0.58	-0.03	-0.07	0.02	-0.02	-0.02	-0.02	0.03	0.03
^{2}BP2	0.47	0.54	0.12	0.04	0.06	0	0	0.02	0.05	0.01
^{2}BP22	-0.09	1.12	-0.11	-0.01	0	-0.01	0	-0.03	0.04	0.05
^{1}BP22(H)	0	-	0	0	0	0	0	-	-	-
^{2}BP222	0.10	0.94	-0.10	0	0.03	0.02	-0.02	0.03	0	0
^{2}B’	0.03	0.82	0.04	-	0.03	0	0	0	-0.01	0.05
^{1}BP222(H)	0	-	0	0	0	0	0	-	-	-
^{1}CP2	0	0	0	0	0	0	0	0	0	0
^{1}CP22	0	0	0	0	0	0	0	0	0	0
^{2}CP22(H)	0.69	-	0.26	-0.05	0.04	0.01	-0.02	-	-	-
^{1}CP222	0	0	0	0	0	0	0	0	0	0
^{1}CP222^{TS}	0	0	0	0	0	0	0	0	0	0
^{1}C’	0	0	-	0	0	0	0	0	0	0
^{1}CP1’	0	0	-	0	0	0	0	0	0	0
^{1}CP11’	0	0	-	0	0	0	0	0	0	0
^{2}CP222(H)	0.58	-	0.34	0	0	0.06	0	-	-	-
^{2}DP222	0.43	0.48	-0.08	0	0.06	0	0	-0.02	0.04	0.02
^{2}DP222^{TS}	0.49	0.38	-0.07	0	0.07	0	0	0.01	0.04	0.02
^{2}D’	0.35	0.40	-0.03	-	0.07	0	0	0	0.04	0.02
^{1}DP222(H)	0	-	0	0	0	0	0	-	-	-
^{1}DP222(H)^{TS}	0	-	0	0	0	0	0	-	-	-
^{2}DP1’	-0.17	1.20	-0.07	-	0.01	-0.01	0	0	0.02	0.06
^{2}DP11’	0.23	0.76	0.02	-	-0.01	0	0	-0.04	0.04	0.02
^{1}D’(H)	0	-	0	-	0	0	0	-	-	-
^{1}DP1’(H)	0	-	0	-	0	0	0	-	-	-
^{1}DP11’(H)	0	-	0	-	0	0	0	-	-	-
^{1}DP111’(H)	0	-	0	-	0	0	0	-	-	-
^{1}EP1’	0	0	0	-	0	0	0	0	0	0
^{1}EP11’	0	0	0	-	0	0	0	0	0	0
^{2}E’(H)	0	-	0.04	-	0.04	-0.01	0.01	-	-	-
^{2}EP1’(H)	0.50	-	0.46	-	-0.03	0	0.07	-	-	-
^{2}EP11’(H)	1.00	-	-0.08	-	-0.04	0.04	0.04	-	-	-
^{2}EP111’(H)	0.98	-	-0.02	-	-0.04	0.04	0.05	-	-	-
^{1}FP1’(H)	0	-	0	-	0	0	0	-	-	-
^{2}FP11’	0	0.95	-0.02	-	0	0	0	0.06	0.03	-0.03
^{1}FP11’(H)	0	-	0	-	0	0	0	-	-	-
^{1}FP111’(H)	0	-	0	-	0	0	0	-	-	-
^{2}GP11’(H)	0.57	-	0.08	-	0.08	0	0	-	-	-
^{2}GP111’(H)	0.86	-	-0.01	-	0.1	0	0	-	-	-

Table S7 – Mulliken Atomic Charges present on the Fe, N and P atoms of the BP86/BS1 optimized geometries calculated for the non-alternating pathway. All atoms are numbered according to their appearance in the coordinates, with the protons in structures P1-P3 being ligated to Fe1, and the protons for P4-P6 attached to Fe2.

Structure	Fe1	Fe2	N1	N2	P1	P2	P3	P4	P5	P6
^{1}A	-0.50	-0.53	-0.10	-0.14	0.36	0.26	0.24	0.40	0.26	0.25
^{1}AP2	-0.43	-0.64	-0.22	-0.10	0.37	0.23	0.24	0.32	0.23	0.43
^{1}AP22	-0.35	-0.86	-0.30	-0.02	0.40	0.20	0.27	0.38	0.28	0.27
^{1}AP222	-0.36	-0.63	-0.40	0.18	0.39	0.17	0.18	0.45	0.21	0.17
^{2}B	-0.41	-0.55	-0.15	-0.17	0.32	0.25	0.24	0.39	0.24	0.25
^{2}BP2	-0.44	-0.65	-0.20	-0.12	0.37	0.26	0.25	0.41	0.25	0.25
^{2}BP22	-0.33	-0.42	-0.30	-0.03	0.38	0.18	0.21	0.36	0.21	0.22
^{1}BP22(H)	-0.35	-	-0.29	-0.01	0.39	0.25	0.24	-	-	-
^{2}BP222	-0.26	-0.39	-0.43	0.19	0.37	0.12	0.18	0.35	0.29	0.20
^{2}B’	-0.53	-0.31	-0.24	-	0.39	0.21	0.19	0.33	0.22	0.27
^{1}BP222(H)	-0.34	-	-0.41	0.17	0.40	0.22	0.32	-	-	-
^{1}CP2	-0.55	-0.60	-0.15	-0.13	0.35	0.23	0.23	0.40	0.23	0.23
^{1}CP22	-0.37	-0.67	-0.22	-0.06	0.35	0.24	0.24	0.38	0.22	0.20
^{2}CP22(H)	-0.43	-	-0.29	-0.07	0.33	0.24	0.23	-	-	-
^{1}CP222	-0.30	-0.62	-0.41	0.17	0.39	0.13	0.17	0.40	0.27	0.22
^{1}CP222^{TS}	-0.31	-0.52	-0.40	0.08	0.38	0.16	0.18	0.42	0.27	0.22
^{1}C’	-0.38	-0.40	-0.26	-	0.37	0.17	0.23	0.33	0.28	0.25
^{1}CP1’	-0.66	-0.49	-0.23	-	0.42	0.20	0.19	0.35	0.23	0.35
^{1}CP11’	-0.61	-0.69	-0.24	-	0.40	0.12	0.26	0.32	0.28	0.30
^{2}CP222(H)	-0.38	-	-0.42	0.17	0.37	0.23	0.24	-	-	-
^{2}DP222	-0.27	-0.68	-0.39	0.18	0.33	0.13	0.14	0.36	0.25	0.21
^{2}DP222^{TS}	-0.26	-0.66	-0.35	0.10	0.33	0.13	0.16	0.38	0.25	0.21
^{2}D’	-0.35	-0.44	-0.30	-	0.35	0.17	0.23	0.34	0.26	0.25
^{1}DP222(H)	-0.49	-	-0.41	0.11	0.33	0.27	0.19	-	-	-
^{1}DP222(H)^{TS}	-0.48	-	-0.37	0.04	0.33	0.28	0.18	-	-	-
^{2}DP1’	-0.53	-0.38	-0.25	-	0.41	0.17	0.20	0.33	0.25	0.23
^{2}DP11’	-0.66	-0.75	-0.21	-	0.43	0.18	0.15	0.50	0.19	0.18
^{1}D’(H)	-0.68	-	-0.25	-	0.34	0.23	0.25	-	-	-
^{1}DP1’(H)	-0.40	-	-0.33	-	0.40	0.25	0.28	-	-	-
^{1}DP11’(H)	-0.50	-	-0.20	-	0.50	0.25	0.26	-	-	-
^{1}DP111’(H)	-0.79	-	-0.05	-	0.39	0.29	0.26	-	-	-
^{1}EP1’	-0.39	-0.58	-0.29	-	0.38	0.15	0.22	0.42	0.27	0.22
^{1}EP11’	-0.48	-0.72	-0.25	-	0.42	0.13	0.16	0.40	0.30	0.25
^{2}E’(H)	-0.73	-	-0.27	-	0.34	0.23	0.25	-	-	-
^{2}EP1’(H)	-0.47	-	-0.33	-	0.35	0.26	0.24	-	-	-
^{2}EP11’(H)	-0.41	-	-0.27	-	0.35	0.20	0.20	-	-	-
^{2}EP111’(H)	-0.50	-	-0.08	-	0.41	0.19	0.21	-	-	-
^{1}FP1’(H)	-0.62	-	-0.38	-	0.30	0.29	0.27	-	-	-
^{2}FP11’	-0.61	-0.44	-0.15	-	0.42	0.16	0.19	0.30	0.25	0.27
^{1}FP11’(H)	-0.47	-	-0.26	-	0.34	0.25	0.22	-	-	-
^{1}FP111’(H)	-0.46	-	-0.10	-	0.38	0.20	0.20	-	-	-
^{2}GP11’(H)	-0.50	-	-0.32	-	0.33	0.26	0.26	-	-	-
^{2}GP111’(H)	-0.53	-	-0.11	-	0.32	0.25	0.24	-	-	-

Table S8 – Absolute, Zero Point, and Gibbs Free Energies (in au) of the BP86/BS1 calculated supplementary structures, including products, redox/protonation agents, and alternate electron configurations. [a] Structures calculated with a def2-TZVP basis set on all atoms. [b] Structures calculated in the gas phase, i.e., without implicit solvation model.

Structure	E [au]	ZPE [au]	G [au]
^{3}A	-7021.009980	1.256521	-7019.854432
^{2}A (+)	-7020.888791	1.263908	-7019.722739
^{1}A (+2)	-7020.740556	1.267268	-7019.568311
^{7}A	-7020.913991	1.253554	-7019.761405
^{4}BP2	-7021.547221	1.264740	-7020.380607
^{5}CP12	-7022.127197	1.275827	-7020.953781
^{3}CP12	-7022.164898	1.279054	-7020.985281
^{2}Cobaltocene[a]	-1770.314054	0.159988	-1770.190053
^{1}Cobaltocene(+)[a]	-1770.188797	0.165131	-1770.056210
^{2}Cobaltocene[ab]	-1770.301243	0.160589	-1770.175660
^{1}Cobaltocene (+)[ab]	-1770.111053	0.166051	-1769.978509
$^{1}Et_2O$	-233.501030	0.131512	-233.399508
$^{1}(Et_2O)_2H^+$	-467.447735	0.276390	-467.210047
^{1}Ferrocene[a]	-1651.257629	0.163444	-1651.126705
^{2}Ferrocene(+)[a]	-1651.072841	0.163523	-1650.943975
^{1}Ferrocene[ab]	-1651.245153	0.171692	-1651.106279
^{2}Ferrocene(+)[ab]	-1650.995300	0.181192	-1650.848445
$^{2}Fe\text{-}(PR_3)_3$ half-complex	-3455.717353	0.620878	-3455.159918
$^{1}Fe\text{-}(PR_3)_3^+$ half-complex	-3455.555633	0.622606	-3454.993502
$^{2}Fe\text{-}(PR_3)_3^{2+}$ half-complex	-3455.397779	0.624601	-3454.834624
$^{2}N_2\text{-}Fe\text{-}(PR_3)_3$ half-complex	-3565.226142	0.633650	-3564.656983
$^{1}N_2\text{-}Fe\text{-}(PR_3)_3^{+1}$ half-complex	-3565.090211	0.637261	-3564.516039
$^{2}N_2\text{-}Fe\text{-}(PR_3)_3^{+2}$ half-complex	-3564.870759	0.634845	-3564.300864
$^{1}N_2$	-109.440252	0.005442	-109.453270
$^{1}N_2H_2$	-110.565730	0.027279	-110.560095
$^{1}N_2H_4$	-111.791443	0.051228	-111.762888
$^{1}NH_3$	-56.515046	0.033119	-56.501044

Table S9 – Absolute and Gibbs free energies (in au) of TPSSh/def2-tzvp single point calculations for the alternating pathway, utilizing geometries optimized with the standard method (BP86). The Gibbs free energy is calculated using ZPE, thermal and entropy corrections as determined with frequency analysis in the standard method optimization run (Table S2).

Structure	E [au]	G [au]
^{1}A	-7023.902243	-7022.736813
^{1}AP2	-7024.352216	-7023.176814
^{1}AP12	-7024.784123	-7023.593199
^{3}AP122	-7025.203771	-7024.003928
^{2}B	-7023.980180	-7022.807559
^{2}BP2	-7024.458516	-7023.285895
^{2}BP12	-7024.949933	-7023.760017
^{2}BP122	-7025.388139	-7024.180920
^{1}CP2	-7024.520308	-7023.342637
^{1}CP12	-7025.066989	-7023.880178
^{1}CP122	-7025.538360	-7024.333180
^{1}CP1122	-7025.989308	-7024.769882
^{2}DP122	-7025.653799	-7024.455714
^{2}DP1122	-7026.122297	-7024.908366
^{1}EP1122	-7026.244387	-7025.033757
^{2}EP11222(H)	-3569.524702	-3568.896924
^{2}EP11222(H)^{TS}	-3569.521546	-3568.899842
^{1}EP11’	-6970.164112	-6968.981716
^{2}EP11’(H)	-3513.002580	-3512.419425
^{2}EP111’(H)	-3513.450924	-3512.850312
^{2}FP11’	-6970.311444	-6969.123326
^{1}FP11’(H)	-3513.002580	-3512.416324
^{1}FP111’(H)	-3513.626150	-3513.028094
^{2}GP11’(H)	-3513.190744	-3512.611306
^{2}GP111’(H)	-3513.733795	-3513.138513

Table S10 – Absolute and Gibbs free energies (in au) of TPSSh/def2-tzvp single point calculations for the non-alternating pathway, utilizing geometries optimized with the standard method (BP86). The Gibbs free energy is calculated using ZPE, thermal and entropy corrections as determined with frequency analysis in the standard method optimization run (Table S5).

Structure	E [au]	G [au]
^{1}A	-7023.902243	-7022.736813
^{1}AP2	-7024.352216	-7023.176814
^{1}AP22	-7024.788514	-7023.588627
^{1}AP222	-7025.173020	-7023.967151
^{2}B	-7023.980180	-7022.807559
^{2}BP2	-7024.458516	-7023.285895
^{2}BP22	-7024.922889	-7023.729032
^{1}BP22(H)	-3567.769021	-3567.178912
^{2}BP222	-7025.341063	-7024.141986
^{2}B’	-6968.811380	-6967.641797
^{1}BP222(H)	-3568.167242	-3567.560126
^{1}CP2	-7024.520308	-7023.342637
^{1}CP22	-7025.005714	-7023.812306
^{2}CP22(H)	-3567.861688	-3567.274814
^{1}CP222	-7025.483045	-7024.284053
^{1}CP222^{TS}	-7025.489148	-7024.281763
^{1}C’	-6968.973538	-6967.800365
^{1}CP1’	-6969.411774	-6968.234905
^{1}CP11’	-6969.820667	-6968.617209
^{2}CP222(H)	-3568.319183	-3567.718488
^{2}DP222	-7025.575865	-7024.384377
^{2}DP222^{TS}	-7025.572322	-7024.371888
^{2}D’	-6969.072215	-6967.905650
^{1}DP222(H)	-3568.427351	-3567.824426
^{1}DP222(H)^{TS}	-3568.426147	-3567.829349
^{2}DP1’	-6969.565495	-6968.396280
^{2}DP11’	-6970.037751	-6968.853686
^{1}D’(H)	-3511.877014	-3511.313014
^{1}DP1’(H)	-3512.372934	-3511.795505
^{1}DP11’(H)	-3512.817973	-3512.226675
^{1}DP111’(H)	-3513.211183	-3512.605617
^{1}EP1’	-6969.668340	-6968.490162
^{1}EP11’	-6970.164112	-6968.975080
^{2}E’(H)	-3511.929043	-3511.372760
^{2}EP1’(H)	-3512.492204	-3511.921525
^{2}EP11’(H)	-3513.002580	-3512.419425
^{2}EP111’(H)	-3513.450924	-3512.850312
^{1}FP1’(H)	-3512.558627	-3511.987899
^{2}FP11’	-6970.311444	-6969.123326
^{1}FP11’(H)	-3513.002580	-3512.416324

^{1}FP111'(H)	-3513.62615	-3513.028094
^{2}GP11'(H)	-3513.190744	-3512.611306
^{2}GP111'(H)	-3513.733795	-3513.138513

Table S11 – Absolute and Gibbs free energies (in au) of TPSSh/def2-tzvp single point calculations for supplementary structures, utilizing geometries optimized with the standard method (BP86). The Gibbs free energy is calculated using ZPE, thermal and entropy corrections as determined with frequency analysis in the standard method optimization run (Table S8).[a] – Geometries for these structures have been reoptimized and frequency analysis performed at the TPSSh/def2-tzvp level.

Structure	E [au]	G [au]
^{3}A	-7023.904528	-7022.739098
^{2}A (+)	-7023.783364	-7022.611120
^{1}A (+2)	-7023.636145	-7022.470092
^{7}A	-7023.766262	-7022.613676
^{4}BP2	-7024.435380	-7023.268766
^{5}CP12	-7025.020549	-7023.847132
^{3}CP12	-7025.054317	-7023.874700
^{2}Cobaltocene[a]	-1770.130092	-1770.000939
^{1}Cobaltocene(+)[a]	-1770.007394	-1769.869432
$^{1}Et_2O$[a]	-233.782211	-233.676839
$^{1}(Et_2O)_2H^{+}$[a]	-468.004650	-467.760084
^{1}Ferrocene[a]	-1651.096461	-1650.960511
^{2}Ferrocene(+)[a]	-1650.901115	-1650.766022
$^{2}Fe-(PR_3)_3$ half-complex	-3457.121183	-3456.563748
$^{1}Fe-(PR_3)_3^{+}$ half-complex	-3456.962874	-3456.400742
$^{2}Fe-(PR_3)_3^{2+}$ half-complex	-3456.805770	-3456.242616
$^{2}N_2-Fe-(PR_3)_3$ half-complex	-3566.739867	-3566.170707
$^{1}N_2-Fe-(PR_3)_3^{+1}$ half-complex	-3566.608204	-3566.034032
$^{2}N_2-Fe-(PR_3)_3^{+2}$ half-complex	-3566.395238	-3565.825343
$^{1}N_2$	-109.567695	-109.580713
$^{1}N_2H_2$	-110.692352	-110.686717
$^{1}N_2H_4$	-111.923667	-111.895112
$^{1}NH_3$	-56.586215	-56.572213

Table S12 – Comparison of key bond lengths (in Å) and Mulliken spin populations between the High Spin (triplet) and Broken Symmetry (singlet) optimized geometries at BP86 level of theory for A. [a] Structure utilizes the def2-TZVP basis set on all atoms.

Structure	Fe1 Spin	Fe2 Spin	N-N Bond	N-Fe1 Bond	N-Fe2 Bond	Fe1-P Bond Avg	Fe2-P Bond Avg
^{3}A[a]	1.214	0.750	1.154	1.814	1.807	2.178	2.151
^{1}A[a]	0.814	-0.785	1.151	1.808	1.825	2.172	2.154
^{1}A	0.805	-0.766	1.161	1.802	1.817	2.185	2.168

Table S13 - Absolute, Zero Point, and Gibbs Free Energies (in au) of optimized geometries calculated for select structures with the PBE0 functional.
[a] Structures calculated with def2-TZVP basis set on all atoms.

Structure	E [au]	ZPE [au]	G [au]
^{1}A	-7015.986292	1.302785	-7014.776988
^{1}AP2	-7016.439962	1.315378	-7015.221194
^{2}B	-7016.052971	1.301073	-7014.847711
^{2}BP2	-7016.531223	1.313767	-7015.311543
^{1}Ferrocene[a]	-1650.309770	0.169770	-1650.172337
^{2}Ferrocene(+)[a]	-1650.117078	0.169958	-1649.980521
^{1}Et$_2$O	-233.224508	0.136317	-233.118055
^{1}(Et$_2$O)$_2$H$^+$	-466.894800	0.285671	-466.648498

Table S14 – Mulliken Spin Populations present on the Fe, N and P atoms of the optimized geometries calculated with the PBE0 functional.

Structure	Fe1	Fe2	N1	N2	P1	P2	P3	P4	P5	P6
^{1}A	-1.07	1.05	0.00	0.03	0.02	0.00	0.00	0.03	0.03	0.00
^{1}AP2	-1.96	2.16	-0.13	0.07	0.05	0.00	-0.03	-0.07	0.01	-0.02
^{2}B	0.80	0.88	-0.17	-0.22	-0.06	-0.01	0.00	-0.06	0.01	0.00
^{2}BP2	0.66	0.88	-0.33	-0.01	-0.02	0.00	0.01	0.03	0.02	0.00

Table S15 – Mulliken Atomic Charges present on the Fe, N and P atoms of the optimized geometries calculated with the PBE0 functional.

Structure	Fe1	Fe2	N1	N2	P1	P2	P3	P4	P5	P6
^{1}A	-0.39	-0.33	-0.09	-0.09	0.42	0.23	0.22	0.41	0.25	0.20
^{1}AP2	-0.29	-0.16	-0.21	-0.21	0.45	0.19	0.20	0.45	0.20	0.20
^{2}B	-0.44	-0.40	-0.19	-0.19	0.41	0.24	0.25	0.40	0.23	0.25
^{2}BP2	-0.47	-0.48	-0.22	-0.16	0.41	0.25	0.29	0.44	0.23	0.23

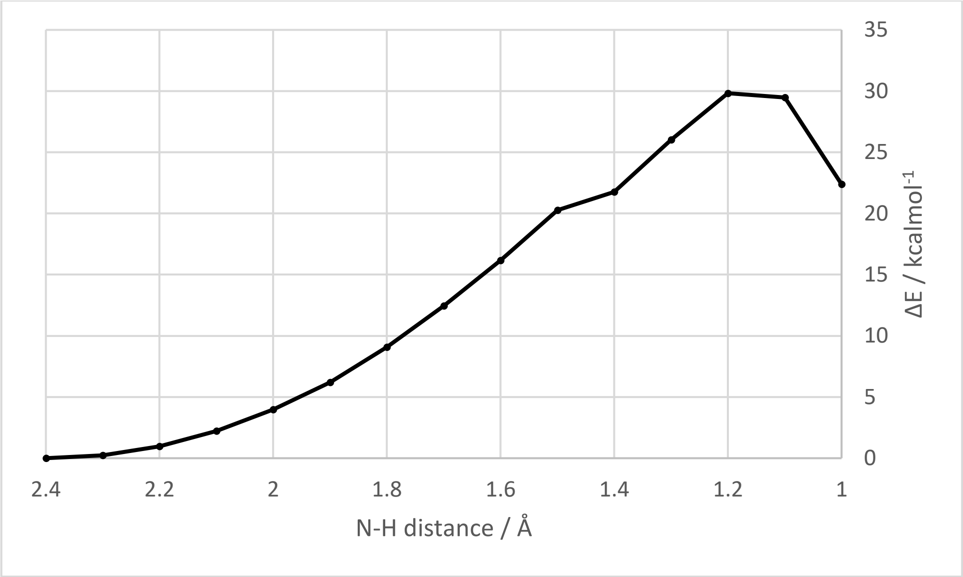

Figure S1 – Relaxed geometry scan for internal H-transfer from the bound H-Fe group to N, furthest distance (left-hand-side) represents H as a hydride on Fe, while the closest distance (right-hand-side) has the proton on N. Each data point represents a full geometry optimization with fixed N-H distance. As can be seen the internal proton transfer has a high barrier of over 30 kcal/mol and is overall endothermic.

Cartesian coordinates of optimized geometries.

Starting structure from crystallographic data

N	16.682709000	18.634802000	20.534650000
N	17.394427000	18.912662000	19.674687000
Fe	15.555778000	18.152491000	21.874202000
P	15.129219000	19.279567000	23.607740000
C	15.678785000	18.386855000	25.122687000
C	15.485512000	18.815450000	26.441297000
H	15.048122000	19.641390000	26.610410000
C	15.929616000	18.039323000	27.504142000
H	15.777388000	18.325827000	28.395781000
C	16.596848000	16.841020000	27.263301000
H	16.874722000	16.295547000	27.990015000
C	16.854485000	16.444412000	25.954116000
H	17.352954000	15.650651000	25.791024000
C	16.383051000	17.202551000	24.870851000
P	16.714161000	16.788725000	23.108778000
C	18.581307000	16.687715000	23.155114000
H	18.845237000	16.103533000	23.921881000
C	19.173637000	18.072858000	23.382343000
H	18.798085000	18.457036000	24.201991000
H	20.145977000	18.001427000	23.468994000
H	18.955248000	18.650726000	22.620812000
C	19.166214000	16.093834000	21.879961000
H	18.786868000	15.203121000	21.730744000
H	18.948309000	16.672735000	21.120786000
H	20.139038000	16.023435000	21.968968000
C	16.263645000	14.975414000	22.962964000
H	17.021235000	14.429898000	23.319777000
C	16.061586000	14.621085000	21.477860000
H	16.873150000	14.845184000	20.974186000
H	15.878015000	13.661666000	21.393042000
H	15.304661000	15.130337000	21.120786000
C	15.004245000	14.628252000	23.744653000
H	15.133457000	14.853767000	24.688910000
H	14.246930000	15.138524000	23.387841000
H	14.820284000	13.669852000	23.660097000
C	15.423512000	21.025623000	24.335501000
C	15.134554000	21.441802000	25.649660000
H	14.638813000	20.874880000	26.228204000
C	15.576327000	22.687444000	26.102547000
H	15.478117000	22.949232000	27.010940000
C	16.164741000	23.530655000	25.167976000
H	16.364634000	24.422378000	25.424525000
C	16.465529000	23.143736000	23.911410000
H	16.943647000	23.725090000	23.330248000
C	16.068714000	21.863106000	23.468994000
H	16.245844000	21.586732000	22.576308000
C	13.280427000	19.573974000	23.734443000
C	12.553731000	19.946001000	24.868495000
H	13.000608000	20.044691000	25.702017000
C	11.197664000	20.172380000	24.792577000
H	10.717764000	20.433945000	25.568507000
C	10.524793000	20.019534000	23.579205000
H	9.591570000	20.188907000	23.526586000
C	11.229632000	19.615676000	22.441751000
H	10.770810000	19.490917000	21.620794000
C	12.603921000	19.395642000	22.509815000
P	13.658418000	18.863489000	21.096701000
C	13.666364000	20.343633000	19.968671000
H	14.146854000	20.068518000	19.136458000
C	12.307075000	20.874286000	19.535156000
H	11.802418000	21.164149000	20.324960000
H	11.812835000	20.166652000	19.073630000
H	12.430650000	21.634951000	18.929648000
C	14.500578000	21.457043000	20.609258000
H	14.578303000	22.208420000	19.984640000
H	15.394092000	21.114715000	20.822351000
H	14.063208000	21.761635000	21.432309000
C	12.578926000	17.645951000	20.200613000
H	11.743906000	18.114159000	19.911340000
C	12.190836000	16.475998000	21.112408000
H	11.566730000	15.885224000	20.639102000
H	11.762608000	16.821338000	21.921847000
H	12.995343000	15.972662000	21.356392000
C	13.311914000	17.145715000	18.954256000
H	14.179734000	16.771744000	19.212375000
H	13.451162000	17.892439000	18.335397000
H	12.775037000	16.453269000	18.516028000
Fe	18.574581000	19.209012000	18.364193000
P	17.590000000	19.617167000	16.486150000
C	18.313637000	18.549858000	15.158117000
C	18.116124000	18.728753000	13.784271000
H	17.577707000	19.446947000	13.474056000
C	18.704066000	17.861305000	12.872213000
H	18.575830000	17.995742000	11.939998000
C	19.479170000	16.796254000	13.319865000
H	19.876829000	16.201144000	12.696556000
C	19.670651000	16.605399000	14.682716000
H	20.196172000	15.874805000	14.987171000
C	19.096839000	17.481945000	15.613884000
C	17.892538000	21.349657000	15.915983000
C	17.321532000	21.949592000	14.786382000
H	16.738762000	21.448877000	14.227996000
C	17.603234000	23.274220000	14.478524000
H	17.243371000	23.666468000	13.691337000
C	18.414288000	24.024112000	15.326182000
H	18.587641000	24.937287000	15.128535000
C	18.971831000	23.444261000	16.459186000
H	19.509525000	23.968106000	17.042181000
C	18.744263000	22.091078000	16.746102000
C	15.791884000	19.489742000	16.056038000
C	14.927194000	20.484565000	16.533010000
H	15.274596000	21.197700000	17.055270000
C	13.568452000	20.439662000	16.249235000
H	12.997536000	21.131108000	16.563115000
C	13.038710000	19.391292000	15.511264000
H	12.109064000	19.363867000	15.317020000
C	13.876594000	18.384137000	15.058115000
H	13.516589000	17.656364000	14.565698000
C	15.246170000	18.432988000	15.321994000
H	15.812141000	17.741066000	15.000260000
P	19.308843000	17.401775000	17.441402000
C	18.287502000	15.896261000	17.913924000
H	18.370037000	15.801781000	18.906087000
C	16.808930000	16.136941000	17.627531000
H	16.663517000	16.163220000	16.659975000
H	16.534795000	16.991131000	18.023873000
H	16.277737000	15.410549000	18.016020000
C	18.725598000	14.552195000	17.313390000
H	18.139917000	13.838798000	17.644285000
H	19.650628000	14.363711000	17.576221000
H	18.665050000	14.595083000	16.335362000
C	21.025059000	16.705603000	17.616013000
H	21.076761000	15.874660000	17.060506000
C	21.311094000	16.320561000	19.062896000
H	22.202653000	15.918978000	19.123369000
H	20.640853000	15.673952000	19.366828000
H	21.273170000	17.120423000	19.628613000
C	22.091293000	17.670524000	17.114171000
H	22.081436000	18.482450000	17.662610000
H	21.905722000	17.906045000	16.180909000
H	22.972279000	17.244409000	17.175691000
P	19.571280000	21.114644000	18.074921000
C	19.513460000	22.264165000	19.533847000

H	19.878933000	23.151201000	19.251643000
C	20.370814000	21.731850000	20.681773000
H	20.382298000	22.386799000	21.411367000
H	21.285758000	21.580551000	20.364228000
H	19.994668000	20.887073000	21.005600000
C	18.063961000	22.458250000	19.980190000
H	17.699494000	21.603046000	20.288310000
H	17.531696000	22.787700000	19.225465000
H	18.032706000	23.109757000	20.712401000
C	21.355055000	21.265751000	17.497424000
H	21.880762000	20.593658000	18.018637000
C	21.439296000	20.842353000	16.026980000
H	21.094737000	21.563247000	15.458384000
H	20.903391000	20.032092000	15.887710000
H	22.373113000	20.659635000	15.793468000
C	22.060660000	22.610939000	17.683815000
H	22.990957000	22.534953000	17.387736000
H	22.040223000	22.863286000	18.631214000
H	21.603271000	23.296388000	17.154748000
H	15.335187000	16.724481000	21.036928000
H	19.261631000	19.069936000	19.879951000

Benchmark optimized starting structure – BP86

N	16.699274000	18.561115000	20.658361000
N	17.438261000	18.956101000	19.855351000
Fe	15.499595000	17.779260000	21.752827000
P	15.063127000	19.141920000	23.383403000
C	15.752669000	18.524824000	24.993044000
C	15.642887000	19.186899000	26.232615000
H	15.125400000	20.156396000	26.300682000
C	16.222846000	18.623893000	27.381650000
H	16.131869000	19.138343000	28.350885000
C	16.929440000	17.410147000	27.290141000
H	17.387846000	16.970834000	28.189859000
C	17.072667000	16.769030000	26.047669000
H	17.653308000	15.834941000	25.984553000
C	16.490967000	17.325489000	24.890635000
P	16.705371000	16.634914000	23.167997000
C	18.590669000	16.629994000	23.074517000
H	18.943069000	15.960259000	23.891049000
C	19.120174000	18.050881000	23.311741000
H	18.776882000	18.482810000	24.271996000
H	20.230688000	18.046675000	23.320905000
H	18.800421000	18.720367000	22.486321000
C	19.095076000	16.115274000	21.724743000
H	18.750929000	15.090849000	21.491761000
H	18.763805000	16.788323000	20.909974000
H	20.204141000	16.106559000	21.721090000
C	16.197935000	14.842741000	23.481395000
H	16.837366000	14.467608000	24.310696000
C	16.387257000	13.951424000	22.249271000
H	17.451647000	13.715121000	22.056618000
H	15.855983000	12.985542000	22.387233000
H	15.976491000	14.449419000	21.344496000
C	14.731673000	14.860512000	23.936926000
H	14.587983000	15.456865000	24.860176000
H	14.087312000	15.305366000	23.150909000
H	14.371570000	13.828872000	24.136562000
C	15.563817000	20.921015000	23.504255000
C	14.682073000	21.976837000	23.811618000
H	13.614743000	21.773020000	23.983878000
C	15.161230000	23.296044000	23.898976000
H	14.463866000	24.114270000	24.138136000
C	16.524061000	23.569099000	23.691490000
H	16.900002000	24.601022000	23.768563000
C	17.405526000	22.518319000	23.377185000
H	18.472541000	22.724650000	23.208704000
C	16.926070000	21.205322000	23.268680000
H	17.606293000	20.384423000	22.996831000
C	13.219291000	19.192427000	23.586531000
C	12.524908000	19.503362000	24.771461000
H	13.074421000	19.760666000	25.688444000
C	11.119340000	19.467929000	24.797494000
H	10.582541000	19.695357000	25.731423000
C	10.405026000	19.135512000	23.634491000
H	9.304551000	19.103783000	23.652196000
C	11.093592000	18.829626000	22.446613000
H	10.518361000	18.556605000	21.549606000
C	12.502630000	18.843924000	22.411149000
P	13.538747000	18.352399000	20.934373000
C	13.416815000	19.850486000	19.791174000
H	13.898161000	19.491589000	18.855044000
C	11.982252000	20.294073000	19.494624000
H	11.464205000	20.626976000	20.417818000
H	11.370148000	19.500121000	19.022148000
H	11.990038000	21.155606000	18.797119000
C	14.259435000	21.002492000	20.341663000
H	14.270756000	21.844552000	19.619446000
H	15.305827000	20.694730000	20.522813000
H	13.848314000	21.387915000	21.296515000
C	12.436614000	17.089586000	20.083078000
H	11.441722000	17.563043000	19.938034000
C	12.283771000	15.849569000	20.967836000
H	11.597514000	15.114960000	20.495377000
H	11.878507000	16.094643000	21.970551000
H	13.269032000	15.360710000	21.109237000
C	13.027874000	16.735490000	18.715751000
H	14.061189000	16.351099000	18.834805000
H	13.072795000	17.603799000	18.029018000
H	12.421055000	15.947991000	18.220375000
Fe	18.639804000	19.386512000	18.562096000
P	17.557519000	19.916481000	16.759823000
C	18.043904000	18.787760000	15.348633000
C	17.671110000	18.972931000	14.003180000
H	16.991240000	19.790745000	13.721159000
C	18.166509000	18.112662000	13.007524000
H	17.884499000	18.271541000	11.954928000
C	19.027157000	17.056880000	13.356570000
H	19.423891000	16.387557000	12.577125000
C	19.378883000	16.850418000	14.702737000
H	20.047925000	16.015898000	14.963134000
C	18.890481000	17.711601000	15.706748000
C	18.068156000	21.602110000	16.130242000
C	17.474042000	22.278804000	15.045485000
H	16.646242000	21.814788000	14.488249000
C	17.908392000	23.567924000	14.695251000
H	17.440727000	24.093561000	13.848004000
C	18.925598000	24.193160000	15.439698000
H	19.254644000	25.211303000	15.179061000
C	19.517425000	23.521621000	16.523337000
H	20.302386000	24.027763000	17.105299000
C	19.101363000	22.218433000	16.866710000
C	15.723760000	20.062219000	16.496352000
C	15.065778000	21.248812000	16.892683000
H	15.648119000	22.074211000	17.329784000
C	13.684353000	21.397652000	16.705576000
H	13.194783000	22.341516000	16.991570000
C	12.926332000	20.347551000	16.155165000
H	11.841005000	20.462511000	16.012556000
C	13.564318000	19.148826000	15.795993000
H	12.981456000	18.319024000	15.366703000
C	14.953954000	19.010457000	15.958728000
H	15.446046000	18.079567000	15.644560000
P	19.232208000	17.567113000	17.531418000
C	18.100061000	16.101459000	18.003328000
H	18.227630000	16.056558000	19.105350000
C	16.637596000	16.449965000	17.720806000
H	16.438174000	16.465053000	16.628869000
H	16.350466000	17.428607000	18.147998000
H	15.975165000	15.681479000	18.169615000
C	18.455623000	14.739838000	17.399052000

H	17.740383000	13.968701000	17.760220000
H	19.470296000	14.386792000	17.669367000
H	18.380421000	14.753592000	16.291141000
C	20.920743000	16.725310000	17.563711000
H	20.819028000	15.807431000	16.946070000
C	21.265330000	16.317137000	18.997643000
H	22.279306000	15.864955000	19.043139000
H	20.551809000	15.576211000	19.406943000
H	21.249567000	17.204682000	19.664200000
C	22.013980000	17.603135000	16.959211000
H	22.167278000	18.509653000	17.575309000
H	21.773568000	17.924903000	15.927425000
H	22.977429000	17.050481000	16.925240000
P	19.808085000	21.179330000	18.244057000
C	19.671664000	22.375918000	19.699148000
H	20.243241000	23.289834000	19.426989000
C	20.276711000	21.750216000	20.960670000
H	20.309675000	22.490732000	21.787903000
H	21.312493000	21.385163000	20.802822000
H	19.670522000	20.880823000	21.287689000
C	18.196623000	22.743117000	19.900381000
H	17.592859000	21.833990000	20.103701000
H	17.770125000	23.235621000	19.003165000
H	18.072831000	23.435641000	20.758009000
C	21.648494000	21.162633000	17.791614000
H	22.009061000	20.277280000	18.358724000
C	21.767755000	20.868389000	16.289287000
H	21.575178000	21.782490000	15.691604000
H	21.035436000	20.100807000	15.966476000
H	22.784620000	20.504137000	16.033065000
C	22.491215000	22.375471000	18.198357000
H	23.552469000	22.210578000	17.910797000
H	22.475592000	22.563382000	19.289659000
H	22.161343000	23.303058000	17.688034000
H	15.403748000	16.464173000	20.936987000
H	19.807142000	19.102041000	19.526033000

Benchmark optimized starting structure – TPSS

N	16.677479000	18.460151000	20.608541000
N	17.404653000	18.855593000	19.805524000
Fe	15.481098000	17.708328000	21.756511000
P	15.094727000	19.151041000	23.332237000
C	15.791161000	18.576078000	24.958008000
C	15.705445000	19.284884000	26.171994000
H	15.196555000	20.253997000	26.211370000
C	16.300170000	18.766939000	27.330537000
H	16.226949000	19.317310000	28.274838000
C	17.000477000	17.550080000	27.276569000
H	17.471565000	17.148524000	28.180446000
C	17.117487000	16.859656000	26.061924000
H	17.692182000	15.927067000	26.026614000
C	16.518152000	17.370442000	24.894315000
P	16.673580000	16.589613000	23.208006000
C	18.552299000	16.472740000	23.080983000
H	18.883509000	15.785635000	23.884071000
C	19.173445000	17.861532000	23.299459000
H	18.930289000	18.282811000	24.289101000
H	20.274525000	17.798497000	23.218013000
H	18.820881000	18.559641000	22.519608000
C	18.993764000	15.929050000	21.717399000
H	18.528072000	14.964619000	21.462785000
H	18.744240000	16.653811000	20.925629000
H	20.088913000	15.787006000	21.716766000
C	16.096192000	14.842689000	23.640726000
H	16.736528000	14.491328000	24.472401000
C	16.213618000	13.864990000	22.464786000
H	17.261741000	13.604299000	22.243023000
H	15.681508000	12.924343000	22.700539000
H	15.764519000	14.303487000	21.554873000
C	14.643106000	14.947949000	24.131135000
H	14.556623000	15.583130000	25.029423000
H	14.004011000	15.389088000	23.346190000
H	14.246939000	13.945906000	24.380252000
C	15.622056000	20.928008000	23.449439000
C	14.741220000	22.011612000	23.622206000
H	13.661533000	21.840164000	23.675861000
C	15.239448000	23.319402000	23.734080000
H	14.542785000	24.154501000	23.868121000
C	16.620566000	23.556144000	23.684191000
H	17.008553000	24.576127000	23.778961000
C	17.504127000	22.479082000	23.506416000
H	18.583476000	22.655569000	23.461724000
C	17.007743000	21.177177000	23.377177000
H	17.696586000	20.339639000	23.227181000
C	13.251296000	19.237418000	23.549728000
C	12.577374000	19.624232000	24.722166000
H	13.140505000	19.938397000	25.605841000
C	11.175417000	19.595745000	24.774974000
H	10.658517000	19.881933000	25.697247000
C	10.440728000	19.194528000	23.650093000
H	9.346384000	19.166439000	23.689749000
C	11.105756000	18.819586000	22.472178000
H	10.517110000	18.500550000	21.606342000
C	12.512424000	18.828818000	22.409930000
P	13.519850000	18.278600000	20.938165000
C	13.381776000	19.740826000	19.750320000
H	13.883489000	19.378564000	18.833265000
C	11.940251000	20.147298000	19.422191000
H	11.396128000	20.454814000	20.333049000
H	11.370237000	19.339427000	18.933021000
H	11.942812000	21.010384000	18.734096000
C	14.184301000	20.923498000	20.300697000
H	14.184206000	21.752699000	19.571194000
H	15.229478000	20.643797000	20.505450000
H	13.737082000	21.302324000	21.235622000
C	12.411922000	16.989443000	20.136955000
H	11.422742000	17.452071000	19.964991000
C	12.250231000	15.782917000	21.069108000
H	11.577785000	15.032682000	20.612917000
H	11.825189000	16.070203000	22.046731000
H	13.231823000	15.309730000	21.245864000
C	13.016759000	16.573343000	18.789890000
H	14.041456000	16.193502000	18.944379000
H	13.069074000	17.411001000	18.073542000
H	12.411382000	15.771205000	18.328359000
Fe	18.615976000	19.422914000	18.559763000
P	17.552274000	19.968195000	16.746816000
C	18.092912000	18.872232000	15.330048000
C	17.760956000	19.089826000	13.980508000
H	17.101753000	19.917328000	13.700208000
C	18.274301000	18.249459000	12.981531000
H	18.024607000	18.433769000	11.930851000
C	19.111137000	17.178130000	13.330047000
H	19.518222000	16.523384000	12.551553000
C	19.424617000	16.940509000	14.677105000
H	20.073499000	16.096611000	14.933474000
C	18.921338000	17.783870000	15.686788000
C	18.050787000	21.673735000	16.160697000
C	17.454483000	22.371414000	15.092345000
H	16.640132000	21.915377000	14.520182000
C	17.872813000	23.671627000	14.777191000
H	17.402848000	24.209673000	13.946556000
C	18.875953000	24.290361000	15.540447000
H	19.189235000	25.314453000	15.309406000
C	19.471743000	23.600346000	16.606045000
H	20.242642000	24.099797000	17.201394000
C	19.073652000	22.284528000	16.913933000
C	15.722801000	20.090994000	16.420896000
C	15.032569000	21.252206000	16.829274000
H	15.583189000	22.070613000	17.305256000
C	13.656376000	21.382147000	16.609291000

H	13.144388000	22.303278000	16.908298000
C	12.933705000	20.336280000	16.012628000
H	11.855269000	20.434339000	15.848946000
C	13.602535000	19.164109000	15.633532000
H	13.049135000	18.340004000	15.169634000
C	14.987617000	19.046951000	15.826620000
H	15.500180000	18.140057000	15.494910000
P	19.226878000	17.604838000	17.513163000
C	18.080589000	16.137120000	17.938976000
H	18.130591000	16.106404000	19.042046000
C	16.637080000	16.471633000	17.551193000
H	16.517886000	16.468983000	16.452788000
H	16.323053000	17.452675000	17.939804000
H	15.954134000	15.708473000	17.963733000
C	18.476946000	14.765574000	17.377571000
H	17.731607000	14.008721000	17.689440000
H	19.460573000	14.418410000	17.735347000
H	18.493008000	14.770569000	16.272245000
C	20.910347000	16.750051000	17.560355000
H	20.809067000	15.837176000	16.945501000
C	21.238257000	16.335721000	18.999385000
H	22.249148000	15.889170000	19.050402000
H	20.524026000	15.590431000	19.384505000
H	21.208648000	17.213036000	19.669843000
C	22.028553000	17.605431000	16.961633000
H	22.205483000	18.503671000	17.574247000
H	21.799127000	17.926699000	15.932383000
H	22.970452000	17.025737000	16.932809000
P	19.794543000	21.221281000	18.260033000
C	19.709863000	22.390742000	19.736690000
H	20.272233000	23.304292000	19.466465000
C	20.351505000	21.742385000	20.970463000
H	20.376098000	22.457857000	21.813034000
H	21.388739000	21.414463000	20.779486000
H	19.773519000	20.853006000	21.275366000
C	18.241463000	22.760129000	19.992237000
H	17.650392000	21.858358000	20.232833000
H	17.784702000	23.239152000	19.108466000
H	18.159045000	23.461919000	20.841171000
C	21.623019000	21.181508000	17.771553000
H	21.990677000	20.302714000	18.332444000
C	21.705467000	20.877542000	16.266468000
H	21.497900000	21.786206000	15.674586000
H	20.968914000	20.109410000	15.971962000
H	22.713437000	20.516341000	15.993231000
C	22.488465000	22.391613000	18.145843000
H	23.536445000	22.202477000	17.844220000
H	22.490743000	22.592516000	19.230021000
H	22.162485000	23.308737000	17.625205000
H	15.371384000	16.366282000	20.973537000
H	19.762870000	19.141669000	19.561603000

Benchmark optimized starting structure – TPSSh

N	16.685975000	18.454031000	20.621906000
N	17.342414000	18.995199000	19.861736000
Fe	15.435058000	17.619935000	21.680134000
P	15.334307000	18.939100000	23.382554000
C	15.970732000	18.137386000	24.925841000
C	16.077279000	18.762355000	26.179019000
H	15.758631000	19.799795000	26.306779000
C	16.623496000	18.068869000	27.262527000
H	16.703826000	18.555967000	28.237456000
C	17.080614000	16.754999000	27.093801000
H	17.513324000	16.214328000	27.939250000
C	17.002096000	16.140374000	25.840423000
H	17.378768000	15.121130000	25.719686000
C	16.454042000	16.829276000	24.745986000
P	16.322684000	16.133545000	23.024600000
C	18.070397000	15.475602000	22.820737000
H	18.310647000	14.893404000	23.726771000
C	19.005049000	16.688360000	22.747155000
H	18.911538000	17.323453000	23.642225000
H	20.054721000	16.358786000	22.678796000
H	18.775541000	17.307216000	21.866227000
C	18.212424000	14.570348000	21.593973000
H	17.807929000	13.564772000	21.776889000
H	17.687950000	14.997487000	20.726434000
H	19.276305000	14.451626000	21.329292000
C	15.246260000	14.631725000	23.404438000
H	15.806809000	14.009222000	24.124677000
C	14.925085000	13.803621000	22.157788000
H	15.807760000	13.287078000	21.757316000
H	14.170001000	13.036532000	22.400105000
H	14.527508000	14.452085000	21.361524000
C	13.955814000	15.132071000	24.067800000
H	14.149836000	15.622948000	25.033184000
H	13.445688000	15.862242000	23.418461000
H	13.266186000	14.289884000	24.245903000
C	16.218464000	20.553836000	23.543297000
C	15.623869000	21.762905000	23.933376000
H	14.551947000	21.807272000	24.137696000
C	16.399590000	22.921434000	24.062303000
H	15.925378000	23.858656000	24.365202000
C	17.774839000	22.879367000	23.812476000
H	18.380401000	23.782699000	23.919709000
C	18.371722000	21.676674000	23.414624000
H	19.443127000	21.638044000	23.210703000
C	17.597037000	20.525475000	23.266610000
H	18.058106000	19.592356000	22.933394000
C	13.555311000	19.360150000	23.682090000
C	12.993320000	19.765081000	24.901619000
H	13.620837000	19.884947000	25.786632000
C	11.617036000	19.999660000	25.000950000
H	11.183758000	20.298468000	25.958493000
C	10.798560000	19.842523000	23.877790000
H	9.722758000	20.018483000	23.954817000
C	11.353106000	19.447528000	22.654599000
H	10.698394000	19.316323000	21.790256000
C	12.729607000	19.194731000	22.544172000
P	13.584083000	18.591166000	21.005316000
C	13.695650000	20.135177000	19.940968000
H	14.202709000	19.766176000	19.033758000
C	12.348688000	20.750074000	19.558805000
H	11.787874000	21.065677000	20.453791000
H	11.714685000	20.063543000	18.978645000
H	12.509905000	21.651694000	18.944437000
C	14.603494000	21.171425000	20.602110000
H	14.778866000	22.013463000	19.912218000
H	15.578182000	20.748075000	20.872701000
H	14.139924000	21.579452000	21.514672000
C	12.266825000	17.587698000	20.136062000
H	11.372058000	18.221971000	20.020604000
C	11.901781000	16.360707000	20.971905000
H	11.122556000	15.768394000	20.462873000
H	11.517723000	16.639938000	21.965702000
H	12.785122000	15.719910000	21.113717000
C	12.784070000	17.193902000	18.749153000
H	13.738066000	16.649333000	18.841374000
H	12.957075000	18.067744000	18.104012000
H	12.055269000	16.541388000	18.239188000
Fe	18.489391000	19.775944000	18.653714000
P	17.497171000	19.854697000	16.712363000
C	18.427046000	18.776320000	15.504882000
C	18.286246000	18.843944000	14.111329000
H	17.556933000	19.522183000	13.663441000
C	19.087739000	18.052539000	13.281263000
H	18.984832000	18.123537000	12.195483000
C	20.025040000	17.177668000	13.842147000
H	20.656448000	16.562916000	13.195537000
C	20.157059000	17.090005000	15.232679000
H	20.892529000	16.402844000	15.657659000
C	19.367498000	17.890289000	16.073591000

C	17.650503000	21.556205000	15.967586000
C	16.951801000	22.005861000	14.835708000
H	16.289190000	21.327999000	14.292610000
C	17.067033000	23.335863000	14.420030000
H	16.514904000	23.681894000	13.542630000
C	17.867826000	24.227639000	15.143692000
H	17.937475000	25.274380000	14.837153000
C	18.576072000	23.781618000	16.264697000
H	19.187475000	24.491729000	16.825451000
C	18.485485000	22.441109000	16.675118000
C	15.734316000	19.524735000	16.221742000
C	14.761921000	20.468293000	16.602755000
H	15.064822000	21.357241000	17.163247000
C	13.422294000	20.298729000	16.245729000
H	12.685300000	21.053316000	16.527686000
C	13.023824000	19.164107000	15.527939000
H	11.974930000	19.028476000	15.253273000
C	13.976267000	18.206123000	15.168897000
H	13.675811000	17.315129000	14.611486000
C	15.322187000	18.388945000	15.506910000
H	16.056436000	17.645311000	15.194055000
P	19.446670000	17.934355000	17.923027000
C	18.452078000	16.386361000	18.375519000
H	18.375582000	16.472235000	19.471432000
C	17.035166000	16.468948000	17.806568000
H	17.045739000	16.314998000	16.715691000
H	16.557640000	17.433606000	18.021512000
H	16.405508000	15.683035000	18.253695000
C	19.088177000	15.043081000	18.012406000
H	18.413767000	14.219196000	18.303595000
H	20.049863000	14.868833000	18.516891000
H	19.251030000	14.960174000	16.924742000
C	21.188054000	17.354722000	18.322656000
H	21.341173000	16.389712000	17.811721000
C	21.293306000	17.135163000	19.834577000
H	22.323314000	16.853724000	20.112224000
H	20.623412000	16.336402000	20.183121000
H	21.025858000	18.057930000	20.374298000
C	22.254106000	18.332482000	17.835931000
H	22.135703000	19.310007000	18.325479000
H	22.210833000	18.483774000	16.747838000
H	23.259106000	17.949609000	18.082966000
P	19.429888000	21.664892000	18.070038000
C	19.350881000	22.985927000	19.396605000
H	19.600840000	23.950118000	18.921879000
C	20.358854000	22.698031000	20.510800000
H	20.264894000	23.447094000	21.314870000
H	21.398962000	22.725218000	20.149620000
H	20.175143000	21.700081000	20.936680000
C	17.915352000	23.041332000	19.926285000
H	17.636385000	22.077597000	20.380252000
H	17.198672000	23.254875000	19.117164000
H	17.811510000	23.826644000	20.693583000
C	21.186990000	21.793345000	17.398429000
H	21.769538000	21.207927000	18.128804000
C	21.244683000	21.088604000	16.039908000
H	20.753996000	21.699365000	15.264650000
H	20.736811000	20.114719000	16.066463000
H	22.290651000	20.927691000	15.730654000
C	21.792328000	23.195840000	17.303854000
H	22.847026000	23.118309000	16.987543000
H	21.777100000	23.738149000	18.260426000
H	21.273642000	23.808957000	16.550918000
H	15.190977000	16.453271000	20.678099000
H	19.537509000	19.883097000	19.788354000

Benchmark optimized starting structure – B3LYP

N	16.676956000	18.467902000	20.645646000
N	17.370393000	18.769557000	19.800843000
Fe	15.400769000	17.763183000	21.810609000
P	15.178885000	19.162402000	23.479466000
C	15.848816000	18.445107000	25.053920000
C	15.866844000	19.105509000	26.292742000
H	15.462546000	20.116023000	26.383861000
C	16.432296000	18.486833000	27.410103000
H	16.440708000	19.002991000	28.373771000
C	16.999863000	17.211646000	27.290962000
H	17.449991000	16.729466000	28.162733000
C	17.010516000	16.564188000	26.052870000
H	17.481625000	15.581376000	25.967899000
C	16.441446000	17.176888000	24.925628000
P	16.452678000	16.434241000	23.218107000
C	18.278102000	16.050770000	23.003109000
H	18.544021000	15.275213000	23.743247000
C	19.109203000	17.313116000	23.260229000
H	18.995967000	17.695464000	24.284830000
H	20.179482000	17.102505000	23.095422000
H	18.818761000	18.110808000	22.557160000
C	18.559383000	15.537494000	21.589865000
H	17.950717000	14.667670000	21.311884000
H	18.359060000	16.332487000	20.858790000
H	19.618878000	15.249795000	21.500416000
C	15.638407000	14.785030000	23.620668000
H	16.203364000	14.353616000	24.464670000
C	15.660160000	13.795315000	22.454854000
H	16.669746000	13.406352000	22.257838000
H	15.015609000	12.928888000	22.681728000
H	15.286795000	14.271028000	21.533524000
C	14.202423000	15.065306000	24.076979000
H	14.164474000	15.752930000	24.935915000
H	13.618282000	15.520680000	23.263271000
H	13.701988000	14.127402000	24.372288000
C	15.870940000	20.870550000	23.640280000
C	15.121055000	22.011653000	23.956945000
H	14.043380000	21.935588000	24.112272000
C	15.745964000	23.258707000	24.077379000
H	15.149013000	24.141051000	24.323485000
C	17.126181000	23.374897000	23.892096000
H	17.614558000	24.347691000	23.992594000
C	17.879798000	22.239355000	23.570904000
H	18.957060000	22.323353000	23.415859000
C	17.254754000	20.999891000	23.432917000
H	17.841071000	20.118046000	23.163108000
C	13.355609000	19.402546000	23.732969000
C	12.735276000	19.755326000	24.939415000
H	13.331042000	19.930386000	25.836605000
C	11.343138000	19.872378000	25.011950000
H	10.867559000	20.133728000	25.960794000
C	10.563931000	19.649814000	23.872826000
H	9.475467000	19.737102000	23.926654000
C	11.175536000	19.302865000	22.663341000
H	10.552593000	19.118734000	21.785792000
C	12.569118000	19.165362000	22.580673000
P	13.488351000	18.613873000	21.058451000
C	13.505482000	20.139767000	19.962325000
H	13.923096000	19.739946000	19.025492000
C	12.133277000	20.750066000	19.676551000
H	11.662502000	21.127241000	20.598711000
H	11.438639000	20.039621000	19.204208000
H	12.236242000	21.611203000	18.994502000
C	14.478375000	21.189665000	20.494783000
H	14.573926000	22.019746000	19.774136000
H	15.476914000	20.769679000	20.664244000
H	14.126254000	21.620847000	21.444033000
C	12.264668000	17.504218000	20.177021000
H	11.322842000	18.061826000	20.046816000
C	11.988722000	16.253483000	21.012514000
H	11.244319000	15.613016000	20.509613000
H	11.598198000	16.499382000	22.012277000
H	12.913766000	15.671401000	21.141546000
C	12.822704000	17.139966000	18.797057000
H	13.832643000	16.708017000	18.893742000

H	12.894024000	18.006633000	18.123351000
H	12.174980000	16.393375000	18.306755000
Fe	18.602808000	19.412557000	18.551479000
P	17.556469000	19.757646000	16.629986000
C	18.440599000	18.800471000	15.295720000
C	18.320441000	19.076297000	13.926724000
H	17.642567000	19.858570000	13.580357000
C	19.075974000	18.362727000	12.991483000
H	18.987615000	18.594265000	11.926677000
C	19.949198000	17.359020000	13.423004000
H	20.547166000	16.801671000	12.696933000
C	20.061188000	17.067856000	14.786450000
H	20.748180000	16.281189000	15.105274000
C	19.317016000	17.786293000	15.735307000
C	17.774669000	21.524842000	16.076278000
C	17.060492000	22.126183000	15.028062000
H	16.327839000	21.549163000	14.460005000
C	17.257022000	23.475036000	14.722108000
H	16.694290000	23.936261000	13.906025000
C	18.158560000	24.236791000	15.474142000
H	18.301213000	25.297604000	15.251184000
C	18.871785000	23.642841000	16.519184000
H	19.556495000	24.256005000	17.107724000
C	18.696111000	22.282963000	16.821617000
C	15.776720000	19.527890000	16.154871000
C	14.834618000	20.410238000	16.711475000
H	15.170655000	21.200815000	17.387706000
C	13.479032000	20.301796000	16.396333000
H	12.764132000	21.006585000	16.825365000
C	13.034136000	19.286885000	15.541494000
H	11.971235000	19.195656000	15.303468000
C	13.959179000	18.392456000	14.996837000
H	13.624473000	17.595635000	14.326940000
C	15.320693000	18.517966000	15.294443000
H	16.029410000	17.827808000	14.835099000
P	19.411518000	17.560200000	17.578360000
C	18.367602000	15.995375000	17.826585000
H	18.469833000	15.815784000	18.907774000
C	16.890755000	16.272760000	17.544111000
H	16.720663000	16.448196000	16.471311000
H	16.516905000	17.142235000	18.099082000
H	16.279850000	15.401799000	17.835769000
C	18.835881000	14.750009000	17.071461000
H	18.188535000	13.891508000	17.322840000
H	19.868046000	14.456837000	17.314809000
H	18.774250000	14.894034000	15.980453000
C	21.142108000	16.869229000	17.833615000
H	21.276891000	16.033356000	17.128848000
C	21.282879000	16.328608000	19.258968000
H	22.327845000	16.032532000	19.454203000
H	20.652751000	15.445137000	19.432584000
H	21.001658000	17.094606000	19.999701000
C	22.206146000	17.923193000	17.539331000
H	22.092391000	18.784725000	18.212688000
H	22.150603000	18.289172000	16.504902000
H	23.214938000	17.502221000	17.691080000
P	19.602804000	21.355556000	18.153888000
C	19.469941000	22.550927000	19.592954000
H	19.830661000	23.529833000	19.236300000
C	20.337488000	22.099135000	20.769660000
H	20.330393000	22.864278000	21.563831000
H	21.387722000	21.932453000	20.483461000
H	19.955780000	21.155374000	21.187374000
C	17.999592000	22.683739000	19.998745000
H	17.601586000	21.718716000	20.347370000
H	17.373510000	23.021705000	19.158375000
H	17.884344000	23.408866000	20.820413000
C	21.388628000	21.499309000	17.571505000
H	21.908993000	20.777081000	18.220323000
C	21.474107000	21.003679000	16.124035000
H	21.077983000	21.760254000	15.427814000
H	20.897099000	20.081484000	15.970414000
H	22.521561000	20.805917000	15.842597000
C	22.071170000	22.859022000	17.726388000
H	23.128574000	22.777224000	17.419516000
H	22.062770000	23.229555000	18.761629000
H	21.610660000	23.625499000	17.084672000
H	15.192697000	16.495152000	20.931940000
H	19.676771000	19.297864000	19.664198000

Benchmark optimized starting structure – PBE0

N	16.675314000	18.503137000	20.616961000
N	17.332201000	18.989245000	19.838575000
Fe	15.416173000	17.670623000	21.692639000
P	15.301534000	18.953558000	23.421490000
C	15.950613000	18.136160000	24.943684000
C	16.060295000	18.740657000	26.202065000
H	15.739472000	19.775500000	26.347523000
C	16.609895000	18.035112000	27.270372000
H	16.693297000	18.508647000	28.251950000
C	17.064822000	16.727605000	27.082343000
H	17.500581000	16.174905000	27.918598000
C	16.981612000	16.132984000	25.824612000
H	17.357163000	15.114959000	25.689372000
C	16.433024000	16.834931000	24.744441000
P	16.304686000	16.162594000	23.018428000
C	18.049245000	15.527315000	22.798916000
H	18.297707000	14.940297000	23.699571000
C	18.975743000	16.736019000	22.728627000
H	18.872691000	17.383488000	23.613374000
H	20.027488000	16.411574000	22.674593000
H	18.760308000	17.347894000	21.839343000
C	18.190606000	14.634335000	21.572659000
H	17.806065000	13.620587000	21.748835000
H	17.651346000	15.055178000	20.710724000
H	19.252083000	14.531348000	21.294520000
C	15.257310000	14.648495000	23.387300000
H	15.831557000	14.039628000	24.108747000
C	14.956855000	13.813948000	22.149677000
H	15.841007000	13.290488000	21.764193000
H	14.198155000	13.049442000	22.385748000
H	14.570107000	14.452414000	21.340387000
C	13.959548000	15.106643000	24.046808000
H	14.133600000	15.660781000	24.980733000
H	13.392126000	15.765214000	23.371033000
H	13.324504000	14.237371000	24.283816000
C	16.142397000	20.583346000	23.599451000
C	15.538800000	21.745247000	24.090778000
H	14.482753000	21.741465000	24.370245000
C	16.278763000	22.921554000	24.223960000
H	15.795584000	23.823767000	24.608356000
C	17.627939000	22.945059000	23.873407000
H	18.208074000	23.864894000	23.984321000
C	18.232968000	21.790655000	23.373260000
H	19.286042000	21.803877000	23.087129000
C	17.493016000	20.620961000	23.225289000
H	17.961301000	19.725343000	22.806817000
C	13.520350000	19.332115000	23.730312000
C	12.950191000	19.671097000	24.961165000
H	13.570371000	19.741849000	25.857382000
C	11.577608000	19.900927000	25.061104000
H	11.136530000	20.150919000	26.029254000
C	10.770640000	19.803158000	23.927980000
H	9.694317000	19.977741000	24.004519000
C	11.332653000	19.465457000	22.696251000
H	10.684123000	19.372457000	21.821840000
C	12.705301000	19.216649000	22.584523000
P	13.558144000	18.654488000	21.035734000
C	13.656606000	20.204454000	19.994126000
H	14.151686000	19.839922000	19.077625000
C	12.317133000	20.825735000	19.627932000
H	11.783754000	21.182814000	20.523698000

H	11.653580000	20.135495000	19.087433000
H	12.474928000	21.703653000	18.979372000
C	14.573879000	21.229861000	20.641787000
H	14.732286000	22.079577000	19.957586000
H	15.556108000	20.809353000	20.888479000
H	14.136825000	21.630927000	21.570176000
C	12.250999000	17.655139000	20.162749000
H	11.346893000	18.280046000	20.071969000
C	11.912007000	16.410520000	20.968590000
H	11.137320000	15.818682000	20.453508000
H	11.532541000	16.654667000	21.972879000
H	12.805119000	15.778041000	21.085115000
C	12.750693000	17.299961000	18.767965000
H	13.712901000	16.765521000	18.831391000
H	12.905846000	18.185677000	18.135279000
H	12.025359000	16.648620000	18.252894000
Fe	18.514925000	19.752121000	18.620529000
P	17.547208000	19.831960000	16.666506000
C	18.481692000	18.748183000	15.476780000
C	18.367202000	18.825089000	14.085303000
H	17.655243000	19.515502000	13.627335000
C	19.169778000	18.030869000	13.266490000
H	19.085751000	18.108913000	12.179312000
C	20.084931000	17.145081000	13.836062000
H	20.720380000	16.526411000	13.196841000
C	20.192569000	17.050312000	15.223681000
H	20.914885000	16.353985000	15.657242000
C	19.400146000	17.851433000	16.054058000
C	17.715681000	21.523700000	15.918805000
C	17.031020000	21.966646000	14.780640000
H	16.365140000	21.288791000	14.240604000
C	17.162545000	23.285678000	14.351060000
H	16.622104000	23.626350000	13.464142000
C	17.964004000	24.175263000	15.068915000
H	18.048791000	25.217244000	14.749521000
C	18.651705000	23.738004000	16.200089000
H	19.261712000	24.451061000	16.759249000
C	18.545874000	22.407135000	16.624493000
C	15.788378000	19.510744000	16.186232000
C	14.822164000	20.438425000	16.603974000
H	15.128984000	21.315828000	17.181442000
C	13.481091000	20.270579000	16.268713000
H	12.745110000	21.012962000	16.585458000
C	13.076641000	19.154134000	15.533413000
H	12.023346000	19.019223000	15.274793000
C	14.023905000	18.212382000	15.136795000
H	13.717822000	17.333519000	14.563043000
C	15.370923000	18.393257000	15.455419000
H	16.104184000	17.660265000	15.115379000
P	19.461658000	17.884298000	17.899710000
C	18.460615000	16.350080000	18.339630000
H	18.391338000	16.441068000	19.435877000
C	17.045720000	16.436399000	17.786445000
H	17.039779000	16.270072000	16.697663000
H	16.570563000	17.403357000	17.993952000
H	16.414068000	15.660416000	18.247580000
C	19.080819000	15.006884000	17.983814000
H	18.406388000	14.189435000	18.290872000
H	20.047324000	14.824980000	18.474998000
H	19.228920000	14.907644000	16.895755000
C	21.191263000	17.303916000	18.309123000
H	21.347752000	16.340693000	17.795910000
C	21.297115000	17.075804000	19.811799000
H	22.322077000	16.771782000	20.082424000
H	20.615900000	16.291016000	20.170560000
H	21.058601000	18.000452000	20.360913000
C	22.255514000	18.277819000	17.834167000
H	22.141261000	19.252705000	18.329375000
H	22.219959000	18.440234000	16.748178000
H	23.259073000	17.892520000	18.080831000
P	19.459769000	21.649446000	18.043520000
C	19.328419000	22.973803000	19.349947000
H	19.590493000	23.933841000	18.873865000
C	20.295742000	22.705614000	20.493612000
H	20.178446000	23.467748000	21.281535000
H	21.348121000	22.724640000	20.171627000
H	20.099027000	21.715822000	20.933119000
C	17.885433000	23.034939000	19.833681000
H	17.584070000	22.074597000	20.281187000
H	17.187406000	23.253144000	19.010310000
H	17.761920000	23.817235000	20.600683000
C	21.226703000	21.782065000	17.428633000
H	21.782357000	21.201709000	18.184296000
C	21.342958000	21.073816000	16.084466000
H	20.887308000	21.677931000	15.283584000
H	20.840129000	20.097587000	16.086629000
H	22.401130000	20.915572000	15.820344000
C	21.831015000	23.177509000	17.350061000
H	22.896915000	23.101426000	17.076030000
H	21.779232000	23.728618000	18.299611000
H	21.345245000	23.787103000	16.573292000
H	15.175913000	16.528726000	20.665665000
H	19.528634000	19.845013000	19.784585000

Hydrazine (N_2H_4)

N	11.312011000	13.026651000	8.297263000
H	11.083223000	13.970345000	8.654150000
H	10.560271000	12.421565000	8.657065000
N	11.232589000	12.980629000	6.860691000
H	12.132574000	13.329360000	6.501485000
H	10.529868000	13.650724000	6.503748000

Nitrogen (N_2)

N	16.714882000	18.569457000	20.641401000
N	17.422653000	18.947759000	19.872311000

Diazene (N_2H_2)

N	16.343918000	18.802865000	20.238352000
N	17.552156000	18.633411000	19.963340000
H	15.889587000	19.179376000	19.367658000
H	18.006492000	18.256900000	20.834032000

Diethyl Ether (Et_2O)

H	-1.240136000	0.000924000	-3.709824000
O	-1.437944000	-0.214271000	-1.054007000
C	-0.942568000	-0.302274000	0.272521000
C	-1.522754000	-1.537362000	0.939868000
H	-1.212638000	0.618002000	0.851471000
H	0.176674000	-0.349538000	0.268244000
H	-1.236349000	-2.456075000	0.387499000
H	-1.149920000	-1.627607000	1.980338000
H	-2.630693000	-1.485638000	0.971339000
C	-0.941561000	0.913951000	-1.756461000
C	-1.523264000	0.922021000	-3.159720000
H	0.177656000	0.886259000	-1.797199000
H	-1.209644000	1.858375000	-1.216790000
H	-2.631004000	0.977280000	-3.127623000
H	-1.148571000	1.795953000	-3.730284000

Protonated Diethyl Ether $(Et_2O)_2H^+$

H	-4.779901000	6.561837000	-2.549063000
O	-5.532910000	3.987682000	-2.477255000
C	-6.144566000	2.780421000	-1.900447000
C	-5.068935000	1.759616000	-1.609287000
H	-6.912275000	2.404123000	-2.609313000
H	-6.656116000	3.113765000	-0.976552000
H	-4.321271000	2.153398000	-0.893153000
H	-5.545291000	0.867634000	-1.155642000
H	-4.548732000	1.425971000	-2.528370000
C	-6.432500000	5.148971000	-2.576942000
C	-5.674994000	6.321671000	-3.156868000
H	-6.778489000	5.349475000	-1.544100000

H	-7.308613000	4.859839000	-3.195039000
H	-5.366701000	6.140613000	-4.205313000
H	-6.340470000	7.207730000	-3.151189000
H	-4.993499000	3.780390000	-3.490080000
O	-4.435266000	3.635162000	-4.600256000
C	-5.106209000	2.885016000	-5.655538000
C	-3.004435000	3.912636000	-4.720712000
C	-4.718281000	1.420636000	-5.699523000
C	-2.178653000	3.105052000	-3.739684000
H	-2.891234000	5.001727000	-4.546431000
H	-2.726142000	3.709407000	-5.773386000
H	-1.110288000	3.375485000	-3.865565000
H	-2.277883000	2.014860000	-3.908875000
H	-2.455648000	3.334549000	-2.691335000
H	-6.185641000	3.017054000	-5.444951000
H	-4.884600000	3.402498000	-6.611266000
H	-3.642714000	1.280350000	-5.927149000
H	-5.294157000	0.925311000	-6.507841000
H	-4.956025000	0.902889000	-4.748820000

Ammonia (NH_3)

N	22.353074000	16.614551000	10.318633000
H	22.353187000	17.550334000	10.754872000
H	23.163438000	16.146609000	10.754902000
H	21.542762000	16.146509000	10.754882000

Cobaltocene

Co	6.344589000	-1.561245000	5.953905000
C	6.026650000	-0.546454000	7.802200000
C	5.793926000	-1.965899000	7.908725000
C	7.027263000	-2.657895000	7.660778000
C	7.975582000	-1.681640000	7.254858000
C	7.360107000	-0.371799000	7.360382000
H	5.287494000	0.235028000	7.954483000
H	4.853560000	-2.430128000	8.196330000
H	7.174797000	-3.733919000	7.679528000
H	7.844394000	0.573501000	7.129679000
C	7.003128000	-1.909393000	4.001631000
C	6.025526000	-2.892531000	4.309638000
C	4.831408000	-2.191442000	4.688986000
C	5.029766000	-0.779986000	4.467660000
C	6.380215000	-0.601285000	4.082295000
H	8.038709000	-2.097096000	3.729431000
H	6.179777000	-3.966969000	4.351726000
H	3.906468000	-2.652872000	5.026706000
H	6.874870000	0.346378000	3.885900000
H	4.298768000	0.003285000	4.647461000
H	8.998738000	-1.872179000	6.940651000

Cobaltocene (+)

Co	6.345202000	-1.560226000	5.953714000
C	5.994265000	-0.566119000	7.703061000
C	5.768666000	-1.971586000	7.869810000
C	6.987334000	-2.656431000	7.553340000
C	7.966053000	-1.673896000	7.191144000
C	7.352249000	-0.381962000	7.283836000
H	5.253309000	0.217897000	7.827422000
H	4.826981000	-2.438039000	8.143575000
H	7.131029000	-3.732740000	7.544450000
H	7.819501000	0.566015000	7.034320000
C	7.029762000	-1.893298000	4.058594000
C	6.050775000	-2.875838000	4.419755000
C	4.831837000	-2.191352000	4.735807000
C	5.057599000	-0.785692000	4.570274000
C	6.415941000	-0.601361000	4.151512000
H	8.067419000	-2.089790000	3.805964000
H	6.217109000	-3.946843000	4.487023000
H	3.913577000	-2.653244000	5.086087000
H	6.906618000	0.352079000	3.980115000
H	4.340139000	0.003711000	4.773116000
H	8.980370000	-1.875826000	6.860033000

Cobaltocene (Gas Phase)

Co	6.344574000	-1.561357000	5.953947000
C	6.027310000	-0.546767000	7.802627000
C	5.795111000	-1.965528000	7.908327000
C	7.027723000	-2.657159000	7.661433000
C	7.974794000	-1.681403000	7.255519000
C	7.359588000	-0.372218000	7.361164000
H	5.289351000	0.234519000	7.955700000
H	4.855653000	-2.429635000	8.196768000
H	7.175830000	-3.732183000	7.681585000
H	7.843332000	0.572900000	7.132083000
C	7.001989000	-1.909536000	4.001587000
C	6.025260000	-2.892041000	4.308919000
C	4.832433000	-2.190982000	4.688744000
C	5.030099000	-0.780341000	4.466936000
C	6.379441000	-0.601984000	4.082054000
H	8.036368000	-2.098180000	3.728186000
H	6.179011000	-3.965705000	4.349582000
H	3.907429000	-2.652356000	5.024405000
H	6.873090000	0.345270000	3.885261000
H	4.299801000	0.002715000	4.645401000
H	8.997544000	-1.872571000	6.942723000

Cobaltocene (+, Gas Phase)

Co	6.345264000	-1.560123000	5.953747000
C	5.996269000	-0.565634000	7.709187000
C	5.770728000	-1.970932000	7.875979000
C	6.989148000	-2.655855000	7.559492000
C	7.967801000	-1.673428000	7.197424000
C	7.354152000	-0.381596000	7.290043000
H	5.259344000	0.219984000	7.848016000
H	4.832573000	-2.436514000	8.163596000
H	7.136785000	-3.731828000	7.564797000
H	7.826446000	0.567758000	7.054914000
C	7.027738000	-1.893518000	4.052429000
C	6.049143000	-2.876186000	4.413707000
C	4.830245000	-2.191964000	4.729682000
C	5.055626000	-0.786402000	4.564299000
C	6.413752000	-0.601848000	4.145367000
H	8.061673000	-2.091109000	3.785094000
H	6.211212000	-3.948797000	4.466863000
H	3.906968000	-2.655293000	5.065092000
H	6.900547000	0.351092000	3.959635000
H	4.333333000	0.002363000	4.752993000
H	8.986990000	-1.874710000	6.880596000

Ferrocene

Fe	18.470240000	0.033734000	7.198375000
C	17.706257000	-1.707019000	6.434298000
C	19.869398000	-0.351248000	8.644535000
C	18.734181000	-1.191011000	5.576844000
C	16.678624000	-0.711848000	6.542483000
C	18.598838000	-0.055426000	9.241432000
C	20.261709000	0.779786000	7.854053000
C	18.341890000	0.123007000	5.155398000
C	17.071331000	0.419163000	5.752167000
C	18.206158000	1.258416000	8.819848000
C	19.233813000	1.774699000	7.962238000
H	17.718352000	-2.670300000	6.937785000
H	20.423231000	-1.280788000	8.748591000
H	19.661563000	-1.694678000	5.316416000
H	15.775574000	-0.789396000	7.142544000
H	18.020777000	-0.721636000	9.876690000
H	21.164701000	0.857495000	7.253924000
H	18.920087000	0.789052000	4.520074000
H	16.517984000	1.349004000	5.648361000
H	17.278810000	1.762011000	9.080594000
H	19.221181000	2.737799000	7.458428000

Ferrocene (+)

Fe	18.470161000	0.033857000	7.198403000

C	17.666608000	-1.757740000	6.359482000
C	19.878597000	-0.320735000	8.660559000
C	18.695502000	-1.240817000	5.521037000
C	16.658566000	-0.757728000	6.498350000
C	18.611126000	-0.018477000	9.265984000
C	20.281852000	0.825319000	7.898214000
C	18.329400000	0.086278000	5.130832000
C	17.061830000	0.388405000	5.736107000
C	18.245049000	1.308619000	8.875761000
C	19.273896000	1.825412000	8.037164000
H	17.682004000	-2.720121000	6.863976000
H	20.426669000	-1.254032000	8.751595000
H	19.623673000	-1.746452000	5.269085000
H	15.762960000	-0.838432000	7.107981000
H	18.023094000	-0.684694000	9.890662000
H	21.177350000	0.905938000	7.288437000
H	18.917522000	0.752635000	4.506391000
H	16.513397000	1.321458000	5.644705000
H	17.316849000	1.814275000	9.127604000
H	19.258592000	2.787847000	7.532749000

Ferrocene (Gas Phase)

Fe	18.470224000	0.033848000	7.198337000
C	17.706472000	-1.706245000	6.434108000
C	19.868814000	-0.350735000	8.644329000
C	18.733584000	-1.190443000	5.577103000
C	16.679379000	-0.711845000	6.542208000
C	18.599089000	-0.054959000	9.240802000
C	20.261068000	0.779541000	7.854466000
C	18.341358000	0.122656000	5.155872000
C	17.071633000	0.418432000	5.752345000
C	18.206864000	1.258140000	8.819571000
C	19.233975000	1.773942000	7.962566000
H	17.718400000	-2.669021000	6.937089000
H	20.422376000	-1.279649000	8.748452000
H	19.660406000	-1.693729000	5.316784000
H	15.776593000	-0.789594000	7.141454000
H	18.021687000	-0.720522000	9.876187000
H	21.163855000	0.857291000	7.255220000
H	18.918761000	0.788218000	4.520487000
H	16.518071000	1.347345000	5.648222000
H	17.280041000	1.761426000	9.079890000
H	19.222047000	2.736718000	7.459585000

Ferrocene (+, Gas Phase)

Fe	18.470224000	0.033848000	7.198337000
C	17.692597000	-1.739705000	6.365474000
C	19.869420000	-0.327022000	8.687421000
C	18.696424000	-1.235858000	5.486944000
C	16.679811000	-0.734991000	6.505935000
C	18.621465000	-0.009698000	9.312996000
C	20.260637000	0.802688000	7.890739000
C	18.318983000	0.077394000	5.083678000
C	17.071028000	0.394719000	5.709253000
C	18.244023000	1.303555000	8.909730000
C	19.247850000	1.807402000	8.031200000
H	17.708123000	-2.706820000	6.860606000
H	20.420410000	-1.256818000	8.797039000
H	19.618080000	-1.743913000	5.215254000
H	15.777043000	-0.816795000	7.104551000
H	18.043070000	-0.667824000	9.955856000
H	21.163405000	0.884492000	7.292123000
H	18.897377000	0.735520000	4.440818000
H	16.520037000	1.324515000	5.599634000
H	17.322368000	1.811610000	9.181421000
H	19.232325000	2.774517000	7.536068000

Fe-$(PR_3)_3$ Half-Complex

Fe	16.400704000	18.063145000	22.038499000
P	16.292932000	18.929458000	23.995562000
C	17.615556000	18.276778000	25.124256000
C	17.769804000	18.599276000	26.487138000
H	17.058219000	19.277952000	26.982762000
C	18.854606000	18.077996000	27.212768000
H	18.970215000	18.323137000	28.279953000
C	19.799902000	17.256288000	26.571405000
H	20.654644000	16.854977000	27.137916000
C	19.665919000	16.959741000	25.203526000
H	20.425541000	16.333313000	24.709197000
C	18.575505000	17.469532000	24.470792000
P	18.278268000	17.144561000	22.651914000
C	19.968257000	17.529808000	21.899126000
H	20.684604000	16.767168000	22.278406000
C	20.421054000	18.926602000	22.344855000
H	20.509752000	19.018768000	23.444917000
H	21.410162000	19.170557000	21.902640000
H	19.703522000	19.698190000	21.992750000
C	19.903107000	17.457523000	20.367559000
H	19.622167000	16.455275000	19.993683000
H	19.155941000	18.177987000	19.973460000
H	20.891224000	17.715634000	19.930213000
C	18.164305000	15.256339000	22.733487000
H	19.073231000	14.910886000	23.274316000
C	18.114714000	14.591050000	21.355329000
H	19.071525000	14.688983000	20.806383000
H	17.908916000	13.504462000	21.464040000
H	17.310308000	15.033106000	20.731554000
C	16.920938000	14.915128000	23.567406000
H	16.967771000	15.344471000	24.588149000
H	16.002597000	15.309878000	23.083838000
H	16.805067000	13.814753000	23.665903000
C	16.473141000	20.746345000	24.359768000
C	15.513470000	21.511939000	25.052364000
H	14.607001000	21.031558000	25.449585000
C	15.701863000	22.894645000	25.229636000
H	14.944364000	23.482229000	25.771839000
C	16.847702000	23.525243000	24.716296000
H	16.991913000	24.608104000	24.853630000
C	17.809784000	22.766002000	24.023597000
H	18.710164000	23.252712000	23.616985000
C	17.620883000	21.387736000	23.844948000
H	18.369358000	20.795968000	23.294673000
C	14.614412000	18.564711000	24.689842000
C	14.267834000	18.425406000	26.046613000
H	15.033753000	18.522744000	26.829966000
C	12.936582000	18.150156000	26.409583000
H	12.672973000	18.024693000	27.471222000
C	11.947324000	18.039092000	25.418358000
H	10.903812000	17.829627000	25.700411000
C	12.289015000	18.179886000	24.060274000
H	11.505600000	18.071360000	23.295766000
C	13.622968000	18.425442000	23.679967000
P	14.250766000	18.449437000	21.911960000
C	13.599164000	20.056039000	21.162107000
H	13.727834000	19.870117000	20.072724000
C	12.126311000	20.358104000	21.449382000
H	11.961281000	20.564406000	22.527051000
H	11.451157000	19.529837000	21.152508000
H	11.802355000	21.260470000	20.887223000
C	14.516018000	21.217634000	21.556201000
H	14.239431000	22.139398000	21.001395000
H	15.579567000	20.988941000	21.337722000
H	14.444914000	21.443212000	22.639008000
C	13.151388000	17.140362000	21.115296000
H	12.097419000	17.391564000	21.364647000
C	13.496125000	15.771963000	21.713065000
H	12.820649000	14.987128000	21.311113000
H	13.402825000	15.761928000	22.818035000
H	14.536967000	15.486448000	21.454460000
C	13.317501000	17.148875000	19.592305000
H	14.387707000	17.038557000	19.315220000

H	12.936586000	18.080377000	19.127022000
H	12.758617000	16.302672000	19.138998000
H	16.452060000	17.317828000	20.642187000

Fe-$(PR_3)_3^+$ Half-Complex

Fe	16.229054000	17.869260000	22.159658000
P	16.107874000	18.690390000	24.199385000
C	17.519917000	18.065419000	25.212501000
C	17.623294000	18.111553000	26.616537000
H	16.781522000	18.457789000	27.232330000
C	18.820911000	17.732578000	27.242191000
H	18.896370000	17.764741000	28.339552000
C	19.925235000	17.326805000	26.470457000
H	20.865647000	17.038179000	26.963974000
C	19.831840000	17.286754000	25.070440000
H	20.699840000	16.957988000	24.477977000
C	18.630543000	17.654543000	24.431051000
P	18.367114000	17.498597000	22.596358000
C	19.966214000	18.025748000	21.787823000
H	20.729093000	17.267463000	22.071734000
C	20.392341000	19.407753000	22.301589000
H	20.566000000	19.424163000	23.395525000
H	21.338324000	19.714886000	21.809460000
H	19.629718000	20.176383000	22.056198000
C	19.789740000	18.035334000	20.262504000
H	19.497254000	17.049535000	19.856319000
H	19.013944000	18.768740000	19.959786000
H	20.743346000	18.330468000	19.777682000
C	18.126457000	15.637003000	22.527409000
H	18.727307000	15.144407000	23.320778000
C	18.428409000	14.978821000	21.182334000
H	19.508367000	15.022855000	20.939301000
H	18.143123000	13.906469000	21.224029000
H	17.857692000	15.447554000	20.355210000
C	16.629760000	15.599628000	22.890924000
H	16.415133000	15.804364000	23.953250000
H	15.977360000	16.205509000	22.118564000
H	16.167167000	14.616441000	22.652839000
C	16.298607000	20.512413000	24.468799000
C	15.206689000	21.350059000	24.775612000
H	14.209065000	20.922270000	24.948511000
C	15.388009000	22.740973000	24.870474000
H	14.528158000	23.383969000	25.112867000
C	16.656169000	23.306123000	24.659401000
H	16.794979000	24.395508000	24.732456000
C	17.750449000	22.473789000	24.360711000
H	18.749716000	22.907077000	24.201963000
C	17.573593000	21.085794000	24.267024000
H	18.436270000	20.443234000	24.039040000
C	14.422158000	18.394372000	24.889590000
C	14.038252000	18.491087000	26.240077000
H	14.788739000	18.643911000	27.027893000
C	12.680355000	18.410931000	26.590362000
H	12.385741000	18.471268000	27.648849000
C	11.699802000	18.272792000	25.592536000
H	10.635327000	18.229016000	25.868232000
C	12.072748000	18.188038000	24.241045000
H	11.294055000	18.076098000	23.471813000
C	13.433842000	18.232381000	23.879201000
P	14.056547000	18.019018000	22.146218000
C	14.064830000	19.643896000	21.219663000
H	13.777959000	19.356397000	20.186913000
C	13.145031000	20.753258000	21.718196000
H	13.376509000	21.031258000	22.765255000
H	12.079925000	20.449930000	21.669971000
H	13.264036000	21.664743000	21.095138000
C	15.564861000	20.014199000	21.220161000
H	15.838435000	20.599699000	20.315774000
H	16.283128000	19.112804000	20.987780000
H	15.872422000	20.608501000	22.094742000
C	12.825031000	16.867190000	21.341639000
H	11.827811000	17.347932000	21.452230000
C	12.830868000	15.520903000	22.078678000
H	12.045512000	14.856935000	21.662311000
H	12.637741000	15.627332000	23.164780000
H	13.803142000	15.001517000	21.954016000
C	13.135797000	16.702107000	19.849538000
H	14.164949000	16.318331000	19.690514000
H	13.033534000	17.650075000	19.284782000
H	12.428113000	15.975503000	19.399598000
H	16.366722000	17.322867000	20.728631000

Fe-$(PR_3)_3^{2+}$ Half-Complex

Fe	16.537159000	18.645814000	21.892300000
P	16.315667000	19.271674000	24.055115000
C	17.597301000	18.473912000	25.082570000
C	17.879447000	18.827667000	26.415368000
H	17.358935000	19.668800000	26.896848000
C	18.848874000	18.101067000	27.123786000
H	19.077169000	18.370323000	28.165628000
C	19.528844000	17.033135000	26.508573000
H	20.287170000	16.468758000	27.071162000
C	19.259448000	16.688164000	25.174716000
H	19.811616000	15.861454000	24.704302000
C	18.293671000	17.418600000	24.454381000
P	17.923687000	17.145557000	22.679762000
C	19.349088000	17.868179000	21.697083000
H	19.517334000	17.157105000	20.861077000
C	20.645894000	18.090008000	22.467405000
H	21.061917000	17.141317000	22.858602000
H	21.402858000	18.530777000	21.785761000
H	20.509313000	18.787555000	23.317903000
C	18.738061000	19.157921000	21.122234000
H	17.889808000	18.922794000	20.412312000
H	18.435704000	19.890186000	21.913286000
H	19.432792000	19.727251000	20.471866000
C	17.877901000	15.308522000	22.456730000
H	18.927334000	15.011896000	22.686007000
C	17.578296000	14.924777000	21.006021000
H	18.255558000	15.425717000	20.285784000
H	17.708819000	13.831163000	20.880529000
H	16.537216000	15.170888000	20.720029000
C	16.936029000	14.668877000	23.484403000
H	17.302538000	14.794979000	24.520841000
H	15.916476000	15.097949000	23.435856000
H	16.850089000	13.581706000	23.285344000
C	16.445270000	21.056897000	24.378964000
C	15.296081000	21.856800000	24.556417000
H	14.298182000	21.397294000	24.610775000
C	15.433548000	23.247714000	24.685389000
H	14.538953000	23.870511000	24.834229000
C	16.707299000	23.840266000	24.630315000
H	16.809637000	24.931311000	24.729886000
C	17.852396000	23.042344000	24.452512000
H	18.850017000	23.504691000	24.416810000
C	17.726306000	21.652195000	24.322326000
H	18.626352000	21.030827000	24.194511000
C	14.688198000	18.724349000	24.685694000
C	14.286968000	18.873753000	26.025897000
H	14.957911000	19.331716000	26.767164000
C	13.012768000	18.432644000	26.415748000
H	12.696127000	18.542340000	27.463394000
C	12.143086000	17.853516000	25.474079000
H	11.144231000	17.513365000	25.784844000
C	12.537658000	17.705253000	24.134800000
H	11.841498000	17.256148000	23.411955000
C	13.818426000	18.137425000	23.737255000
P	14.431124000	18.079714000	22.010767000
C	13.850571000	19.638712000	21.146997000
H	13.590842000	19.294659000	20.124798000
C	12.641431000	20.314110000	21.786042000
H	12.854979000	20.649714000	22.820548000

H	11.758880000	19.645247000	21.814449000
H	12.363608000	21.209611000	21.191834000
C	15.080732000	20.557233000	21.045620000
H	14.940887000	21.337039000	20.268330000
H	15.992148000	20.026747000	20.624196000
H	15.320312000	21.087840000	21.985416000
C	13.553149000	16.679151000	21.160352000
H	12.494217000	17.022732000	21.215904000
C	13.685898000	15.337050000	21.884446000
H	12.952226000	14.621415000	21.462005000
H	13.493172000	15.407427000	22.972385000
H	14.691166000	14.899387000	21.736317000
C	13.959328000	16.587072000	19.686549000
H	15.032654000	16.326983000	19.575233000
H	13.777556000	17.524679000	19.125758000
H	13.368221000	15.788649000	19.194659000
H	16.275508000	17.492597000	20.929504000

N_2-Fe-$(PR_3)_3$ half-complex

Fe	16.505794000	18.103175000	21.867101000
P	16.389447000	19.015970000	23.822361000
C	17.623729000	18.228496000	24.962153000
C	17.817948000	18.582403000	26.312738000
H	17.206312000	19.375031000	26.771027000
C	18.815890000	17.948021000	27.070414000
H	18.964096000	18.225507000	28.125485000
C	19.633288000	16.968253000	26.476832000
H	20.418148000	16.472695000	27.068987000
C	19.464415000	16.635688000	25.122414000
H	20.126634000	15.885389000	24.662511000
C	18.464554000	17.266085000	24.354062000
P	18.263836000	16.994704000	22.519282000
C	20.008681000	17.433823000	21.939961000
H	20.699398000	16.773092000	22.510120000
C	20.316129000	18.894826000	22.285034000
H	20.192404000	19.105006000	23.366234000
H	21.365004000	19.140816000	22.014723000
H	19.656823000	19.582670000	21.717634000
C	20.185545000	17.167188000	20.440500000
H	20.124101000	16.089668000	20.191368000
H	19.420189000	17.704956000	19.842622000
H	21.182653000	17.524049000	20.104342000
C	18.212178000	15.111192000	22.355090000
H	19.261273000	14.754338000	22.455560000
C	17.673203000	14.743502000	20.965421000
H	18.263838000	15.186232000	20.140598000
H	17.670692000	13.641326000	20.827310000
H	16.630090000	15.107738000	20.854388000
C	17.348037000	14.477223000	23.450599000
H	17.772457000	14.614890000	24.463711000
H	16.328188000	14.917307000	23.446445000
H	17.243677000	13.385957000	23.272133000
C	16.638971000	20.808643000	24.179861000
C	15.722012000	21.617351000	24.883335000
H	14.809973000	21.175715000	25.311347000
C	15.961373000	22.994707000	25.032457000
H	15.237048000	23.615771000	25.582535000
C	17.116147000	23.578056000	24.483648000
H	17.300367000	24.657147000	24.601269000
C	18.033208000	22.776855000	23.777494000
H	18.937129000	23.226739000	23.337939000
C	17.792109000	21.404399000	23.620239000
H	18.496108000	20.781253000	23.049916000
C	14.697403000	18.674719000	24.502248000
C	14.332615000	18.658231000	25.861660000
H	15.085163000	18.839857000	26.642920000
C	13.001484000	18.399026000	26.233002000
H	12.724264000	18.379395000	27.298275000
C	12.029867000	18.163053000	25.245577000
H	10.987154000	17.958957000	25.534059000
C	12.389085000	18.170651000	23.885976000
H	11.623224000	17.956846000	23.125420000
C	13.722270000	18.416435000	23.502358000
P	14.342648000	18.352305000	21.745221000
C	13.660546000	19.930078000	20.959259000
H	14.065517000	19.873929000	19.924045000
C	12.133887000	20.032494000	20.898450000
H	11.688498000	20.077661000	21.914147000
H	11.664177000	19.188665000	20.355073000
H	11.837419000	20.964838000	20.371530000
C	14.278874000	21.153032000	21.645054000
H	13.983235000	22.082646000	21.113835000
H	15.384156000	21.107176000	21.659700000
H	13.929253000	21.246282000	22.693984000
C	13.294122000	16.993278000	20.975679000
H	12.230520000	17.285855000	21.110112000
C	13.528085000	15.650843000	21.674154000
H	12.840208000	14.880075000	21.266277000
H	13.357370000	15.712767000	22.767866000
H	14.567996000	15.301322000	21.517575000
C	13.610447000	16.915761000	19.476492000
H	14.691853000	16.715758000	19.315060000
H	13.357748000	17.851407000	18.937626000
H	13.038388000	16.092529000	18.998834000
H	15.931641000	16.847002000	22.543949000
N	17.122254000	19.334684000	20.749092000
N	17.489790000	20.115208000	19.989241000

A (+)

N	16.715699000	18.509391000	20.646848000
N	17.441384000	18.922144000	19.832650000
Fe	15.523972000	17.766742000	21.729954000
P	15.047968000	19.107000000	23.419215000
C	15.721064000	18.461592000	25.011871000
C	15.605955000	19.111661000	26.257324000
H	15.106160000	20.089628000	26.332121000
C	16.157979000	18.520294000	27.404376000
H	16.066525000	19.024053000	28.378718000
C	16.835799000	17.290291000	27.306313000
H	17.269139000	16.828138000	28.206561000
C	16.984808000	16.662022000	26.059235000
H	17.544379000	15.715789000	25.993033000
C	16.436785000	17.249831000	24.901143000
P	16.703511000	16.587384000	23.180456000
C	18.585465000	16.608660000	23.143470000
H	18.911717000	15.931761000	23.963974000
C	19.091127000	18.031054000	23.417514000
H	18.722211000	18.447895000	24.374712000
H	20.200499000	18.036848000	23.451369000
H	18.785178000	18.710358000	22.594590000
C	19.144316000	16.115435000	21.809223000
H	18.817433000	15.092762000	21.545453000
H	18.842280000	16.803085000	20.995329000
H	20.252144000	16.113413000	21.851531000
C	16.177910000	14.794839000	23.420779000
H	16.814013000	14.388636000	24.237704000
C	16.369731000	13.947523000	22.158711000
H	17.436032000	13.744446000	21.942104000
H	15.865692000	12.965540000	22.279410000
H	15.929677000	14.455988000	21.274221000
C	14.710770000	14.803879000	23.874858000
H	14.568958000	15.351491000	24.827635000
H	14.065814000	15.285886000	23.111681000
H	14.345709000	13.765819000	24.021399000
C	15.560935000	20.873207000	23.488205000
C	14.689560000	21.924379000	23.836131000
H	13.631670000	21.718683000	24.056686000
C	15.170398000	23.244159000	23.893572000
H	14.484224000	24.061652000	24.163512000
C	16.519541000	23.518938000	23.612740000
H	16.894894000	24.552404000	23.664158000
C	17.389113000	22.470446000	23.261386000

H	18.445950000	22.678959000	23.040463000
C	16.910291000	21.154947000	23.186627000
H	17.581402000	20.334796000	22.892459000
C	13.208983000	19.124004000	23.585603000
C	12.505221000	19.422375000	24.768346000
H	13.046103000	19.668455000	25.693260000
C	11.101124000	19.388177000	24.779268000
H	10.555463000	19.611395000	25.708573000
C	10.398471000	19.060047000	23.607495000
H	9.298278000	19.027563000	23.614603000
C	11.096116000	18.758577000	22.425632000
H	10.527650000	18.485670000	21.524790000
C	12.505655000	18.780570000	22.400758000
P	13.532364000	18.328094000	20.910744000
C	13.412767000	19.845816000	19.799746000
H	13.872760000	19.498842000	18.848310000
C	11.973821000	20.299961000	19.540739000
H	11.478929000	20.624632000	20.478925000
H	11.347575000	19.516093000	19.070760000
H	11.973806000	21.170665000	18.855557000
C	14.269272000	20.983234000	20.358911000
H	14.266754000	21.839964000	19.655356000
H	15.319608000	20.674103000	20.514769000
H	13.875495000	21.350358000	21.327430000
C	12.455362000	17.061866000	20.044130000
H	11.462476000	17.537788000	19.895569000
C	12.293537000	15.815288000	20.918329000
H	11.602747000	15.093330000	20.434695000
H	11.885772000	16.051200000	21.921821000
H	13.271799000	15.311638000	21.055289000
C	13.052280000	16.718903000	18.676871000
H	14.067558000	16.291424000	18.796252000
H	13.134981000	17.599069000	18.009339000
H	12.422074000	15.965767000	18.159411000
Fe	18.629255000	19.486194000	18.616153000
P	17.485443000	19.988719000	16.793935000
C	17.984971000	18.826700000	15.430306000
C	17.577724000	18.962865000	14.089365000
H	16.873397000	19.756160000	13.798444000
C	18.068730000	18.080198000	13.113681000
H	17.753663000	18.193645000	12.065165000
C	18.965964000	17.058883000	13.474517000
H	19.356496000	16.371954000	12.708210000
C	19.364046000	16.908789000	14.813566000
H	20.063426000	16.102910000	15.081754000
C	18.874666000	17.790997000	15.798738000
C	18.036302000	21.631754000	16.117198000
C	17.400422000	22.313152000	15.059675000
H	16.509716000	21.885156000	14.576429000
C	17.885469000	23.560194000	14.636970000
H	17.386598000	24.089960000	13.811063000
C	18.995874000	24.138660000	15.278896000
H	19.365473000	25.125448000	14.960738000
C	19.630889000	23.463047000	16.333296000
H	20.489038000	23.935711000	16.832972000
C	19.162236000	22.199402000	16.749471000
C	15.658663000	20.138806000	16.567771000
C	15.023796000	21.338927000	16.960053000
H	15.616473000	22.158491000	17.393848000
C	13.645799000	21.509020000	16.766423000
H	13.170693000	22.460885000	17.048650000
C	12.875545000	20.471103000	16.211371000
H	11.793881000	20.605121000	16.059987000
C	13.493020000	19.261096000	15.855786000
H	12.898422000	18.442672000	15.422142000
C	14.878656000	19.096744000	16.026569000
H	15.353619000	18.156409000	15.715006000
P	19.233776000	17.671370000	17.609850000
C	18.076484000	16.255516000	18.141863000
H	18.129581000	16.295335000	19.249152000
C	16.632936000	16.552084000	17.730328000
H	16.510786000	16.499794000	16.629147000
H	16.282040000	17.539986000	18.081864000
H	15.965106000	15.788382000	18.176407000
C	18.493379000	14.858643000	17.669227000
H	17.741666000	14.117497000	18.014961000
H	19.472859000	14.532257000	18.068787000
H	18.526307000	14.789597000	16.561912000
C	20.921000000	16.847278000	17.680429000
H	20.763851000	15.893336000	17.134185000
C	21.294538000	16.521028000	19.127778000
H	22.301241000	16.054824000	19.165512000
H	20.585156000	15.811789000	19.592750000
H	21.314799000	17.439560000	19.750288000
C	22.033596000	17.613167000	16.967827000
H	22.319430000	18.522490000	17.527986000
H	21.759069000	17.905006000	15.936715000
H	22.936984000	16.971194000	16.902241000
P	19.904945000	21.190071000	18.117767000
C	19.743708000	22.374070000	19.574679000
H	20.299736000	23.287757000	19.271720000
C	20.369413000	21.791600000	20.846939000
H	20.416341000	22.567631000	21.639136000
H	21.400616000	21.417186000	20.686781000
H	19.768512000	20.939385000	21.222965000
C	18.264575000	22.733865000	19.773377000
H	17.665182000	21.841141000	20.053203000
H	17.817603000	23.170765000	18.858469000
H	18.153389000	23.477394000	20.588057000
C	21.727408000	21.119668000	17.665106000
H	22.074072000	20.237614000	18.244165000
C	21.836802000	20.812322000	16.163646000
H	21.649202000	21.722832000	15.560349000
H	21.104867000	20.045963000	15.838787000
H	22.852317000	20.443362000	15.912775000
C	22.586065000	22.324797000	18.063314000
H	23.643769000	22.125447000	17.789095000
H	22.565094000	22.529525000	19.150939000
H	22.285676000	23.250198000	17.532660000
H	15.371133000	16.458541000	20.936598000
H	19.777038000	19.205533000	19.587875000

A (+2)

N	16.738889000	18.596198000	20.505350000
N	17.378620000	19.189635000	19.745960000
Fe	15.889765000	17.628630000	21.712706000
P	15.725312000	18.946977000	23.493246000
C	16.140032000	18.016889000	25.024239000
C	16.494819000	18.605730000	26.251629000
H	16.606314000	19.697802000	26.334795000
C	16.713016000	17.785420000	27.370990000
H	16.991782000	18.238179000	28.334435000
C	16.579694000	16.388992000	27.262161000
H	16.753857000	15.750187000	28.141103000
C	16.243993000	15.800233000	26.030884000
H	16.168817000	14.704184000	25.957349000
C	16.028201000	16.614188000	24.902622000
P	15.731007000	15.956562000	23.195427000
C	17.416476000	15.202791000	22.847510000
H	17.728288000	14.641642000	23.753733000
C	18.318775000	16.437590000	22.671934000
H	18.413462000	17.037654000	23.595726000
H	19.339012000	16.147327000	22.350065000
H	17.941365000	17.110096000	21.851732000
C	17.470504000	14.280949000	21.626752000
H	16.966084000	13.314665000	21.809620000
H	17.006043000	14.755982000	20.738845000
H	18.530535000	14.059709000	21.383035000
C	14.436137000	14.655274000	23.473963000
H	14.842743000	14.020912000	24.290803000
C	14.211468000	13.767029000	22.240016000
H	14.944804000	12.940267000	22.200523000

H	13.202069000	13.309732000	22.277156000
H	14.298238000	14.325988000	21.287445000
C	13.184999000	15.375639000	24.004852000
H	13.370482000	15.825702000	25.000234000
H	12.856436000	16.191525000	23.333592000
H	12.344533000	14.659378000	24.107421000
C	16.663179000	20.512257000	23.626864000
C	16.118077000	21.727742000	24.084837000
H	15.072303000	21.778376000	24.421315000
C	16.910911000	22.887956000	24.094571000
H	16.480534000	23.837450000	24.447585000
C	18.245708000	22.838622000	23.656682000
H	18.862358000	23.750149000	23.668086000
C	18.791805000	21.624855000	23.201603000
H	19.835125000	21.579707000	22.857689000
C	18.001449000	20.467474000	23.175502000
H	18.421856000	19.524564000	22.793207000
C	13.930720000	19.301056000	23.743553000
C	13.377975000	19.758027000	24.953638000
H	14.025205000	19.972771000	25.817033000
C	11.986470000	19.914009000	25.070002000
H	11.554472000	20.266182000	26.018661000
C	11.147789000	19.608372000	23.984055000
H	10.057692000	19.722533000	24.081128000
C	11.692370000	19.144197000	22.774214000
H	11.021689000	18.900278000	21.937607000
C	13.085782000	18.981476000	22.650340000
P	13.952746000	18.421250000	21.124641000
C	14.096100000	20.034830000	20.134516000
H	14.687707000	19.739944000	19.241753000
C	12.736065000	20.581156000	19.682565000
H	12.076352000	20.796107000	20.548485000
H	12.199375000	19.909535000	18.988334000
H	12.891593000	21.543793000	19.152825000
C	14.856778000	21.120246000	20.898855000
H	14.982229000	22.006819000	20.244800000
H	15.860486000	20.806607000	21.230852000
H	14.287461000	21.454961000	21.789078000
C	12.732095000	17.457533000	20.085853000
H	11.939604000	18.204704000	19.873862000
C	12.079134000	16.265728000	20.778730000
H	11.319817000	15.823163000	20.101284000
H	11.560639000	16.546317000	21.715302000
H	12.815036000	15.477507000	21.009492000
C	13.406124000	17.067403000	18.764282000
H	14.220327000	16.336397000	18.937036000
H	13.836831000	17.934914000	18.228025000
H	12.661249000	16.600758000	18.088737000
Fe	18.435087000	20.044883000	18.610507000
P	17.389484000	19.956500000	16.633490000
C	18.479480000	18.913148000	15.556443000
C	18.426885000	18.939607000	14.151458000
H	17.671576000	19.549976000	13.634034000
C	19.359020000	18.201556000	13.402812000
H	19.323551000	18.233410000	12.303525000
C	20.341365000	17.434484000	14.053366000
H	21.075461000	16.864957000	13.463918000
C	20.397618000	17.397727000	15.457232000
H	21.176554000	16.800380000	15.953429000
C	19.471118000	18.141480000	16.213242000
C	17.469698000	21.608270000	15.810503000
C	16.626641000	22.026496000	14.763760000
H	15.861217000	21.348253000	14.358764000
C	16.750127000	23.327639000	14.249953000
H	16.090858000	23.656515000	13.432514000
C	17.697078000	24.214285000	14.791825000
H	17.772758000	25.242999000	14.408540000
C	18.544320000	23.796950000	15.832153000
H	19.262845000	24.510910000	16.257513000
C	18.446121000	22.484346000	16.335765000
C	15.670327000	19.439305000	16.233108000
C	14.620347000	20.279170000	16.668795000
H	14.846103000	21.195690000	17.236180000
C	13.291046000	19.975564000	16.338276000
H	12.482363000	20.651905000	16.650439000
C	12.993940000	18.815399000	15.601173000
H	11.950048000	18.575938000	15.348612000
C	14.032699000	17.966526000	15.185915000
H	13.807776000	17.057913000	14.607198000
C	15.367783000	18.281068000	15.489978000
H	16.173602000	17.626944000	15.128992000
P	19.421644000	18.203261000	18.049723000
C	18.475410000	16.633345000	18.512272000
H	18.367398000	16.711950000	19.614602000
C	17.082616000	16.602357000	17.882173000
H	17.150224000	16.452240000	16.786798000
H	16.492722000	17.515566000	18.075770000
H	16.511053000	15.748417000	18.297794000
C	19.242998000	15.348807000	18.173703000
H	18.619253000	14.472404000	18.446103000
H	20.201478000	15.248368000	18.716997000
H	19.448732000	15.270702000	17.086285000
C	21.133871000	17.837049000	18.697773000
H	21.424564000	16.891006000	18.193577000
C	21.058366000	17.599161000	20.211880000
H	22.079843000	17.461878000	20.621229000
H	20.477123000	16.694478000	20.473220000
H	20.600809000	18.470225000	20.726979000
C	22.155634000	18.915362000	18.348957000
H	21.933708000	19.853755000	18.893832000
H	22.192723000	19.128289000	17.263885000
H	23.167351000	18.582354000	18.658643000
P	19.521798000	21.794828000	17.672251000
C	19.270711000	23.003005000	19.071898000
H	19.256822000	24.033681000	18.659607000
C	20.356119000	22.889698000	20.143682000
H	20.124416000	23.572817000	20.986352000
H	21.356293000	23.167304000	19.756983000
H	20.415666000	21.856613000	20.541014000
C	17.870234000	22.627509000	19.587094000
H	17.839345000	21.541000000	19.893985000
H	17.079342000	22.827768000	18.840429000
H	17.610759000	23.164171000	20.522439000
C	21.255168000	21.952900000	16.997856000
H	21.868769000	21.449726000	17.771318000
C	21.363349000	21.179123000	15.677193000
H	20.770350000	21.669452000	14.878487000
H	21.007662000	20.134954000	15.761426000
H	22.420223000	21.151549000	15.341549000
C	21.754340000	23.396298000	16.848909000
H	22.847488000	23.376600000	16.657869000
H	21.591377000	24.012617000	17.754240000
H	21.286373000	23.908757000	15.986261000
H	15.665859000	16.469296000	20.743152000
H	19.568327000	20.147075000	19.623532000

A (Septet)

N	16.784887000	18.696684000	20.520339000
N	17.536849000	18.986750000	19.667209000
Fe	15.641373000	17.970816000	21.660086000
P	15.193277000	19.391852000	23.388770000
C	15.874620000	18.696397000	24.929542000
C	15.737104000	19.324897000	26.205924000
H	15.328825000	20.344883000	26.275740000
C	16.162185000	18.667010000	27.364769000
H	16.050026000	19.164997000	28.341699000
C	16.753650000	17.384700000	27.296464000
H	17.096680000	16.878310000	28.211187000
C	16.924384000	16.767714000	26.033573000
H	17.416971000	15.783593000	25.974115000
C	16.490569000	17.411481000	24.865584000
P	16.711932000	16.703519000	23.179123000

C	18.578060000	16.646752000	22.987439000
H	18.925966000	15.923060000	23.757812000
C	19.164047000	18.032057000	23.283584000
H	18.917341000	18.384953000	24.303851000
H	20.269403000	18.005570000	23.187942000
H	18.782515000	18.775310000	22.553691000
C	19.007153000	16.171076000	21.596796000
H	18.583958000	15.186074000	21.327653000
H	18.708840000	16.902652000	20.820522000
H	20.111372000	16.081028000	21.566662000
C	16.110394000	14.942045000	23.421998000
H	16.757212000	14.510472000	24.217290000
C	16.227159000	14.081908000	22.159097000
H	17.275503000	13.814217000	21.924608000
H	15.668129000	13.132428000	22.294682000
H	15.804530000	14.608761000	21.277576000
C	14.664611000	15.019073000	23.935962000
H	14.596633000	15.555727000	24.902160000
H	14.014610000	15.549374000	23.212404000
H	14.251015000	13.998739000	24.077337000
C	15.844927000	21.127012000	23.486049000
C	15.001186000	22.251364000	23.404590000
H	13.921337000	22.110071000	23.248873000
C	15.528789000	23.546949000	23.547225000
H	14.858313000	24.418708000	23.488789000
C	16.902301000	23.727789000	23.782463000
H	17.314163000	24.741635000	23.903951000
C	17.748377000	22.606321000	23.870070000
H	18.823557000	22.741773000	24.063829000
C	17.223745000	21.314451000	23.716874000
H	17.882714000	20.438023000	23.801320000
C	13.392124000	19.522998000	23.516183000
C	12.706913000	20.032820000	24.666454000
H	13.272931000	20.556411000	25.451556000
C	11.329158000	19.873255000	24.805440000
H	10.824750000	20.273583000	25.699857000
C	10.567590000	19.209323000	23.803928000
H	9.480608000	19.083857000	23.919058000
C	11.219137000	18.734634000	22.644852000
H	10.622515000	18.249798000	21.855921000
C	12.605218000	18.890722000	22.478450000
P	13.532269000	18.377411000	20.985640000
C	13.405902000	19.816665000	19.777859000
H	13.971152000	19.468052000	18.887458000
C	11.952203000	20.114442000	19.398515000
H	11.332673000	20.288904000	20.302975000
H	11.486312000	19.300313000	18.810150000
H	11.901582000	21.033322000	18.780599000
C	14.108383000	21.052089000	20.339743000
H	14.118449000	21.860928000	19.580902000
H	15.157281000	20.842750000	20.623414000
H	13.578072000	21.435204000	21.232635000
C	12.505135000	17.042869000	20.171387000
H	11.491970000	17.480594000	20.043104000
C	12.403028000	15.801775000	21.060926000
H	11.710727000	15.062206000	20.606463000
H	12.022304000	16.040960000	22.073510000
H	13.392890000	15.314103000	21.166399000
C	13.083768000	16.699859000	18.795104000
H	14.125206000	16.333870000	18.895102000
H	13.098102000	17.561647000	18.099566000
H	12.484865000	15.896717000	18.316972000
Fe	18.753648000	19.219837000	18.407221000
P	17.634606000	19.850265000	16.536118000
C	18.136459000	18.737559000	15.201026000
C	17.857034000	18.974775000	13.817209000
H	17.191241000	19.802433000	13.528556000
C	18.412147000	18.167640000	12.827124000
H	18.181681000	18.370815000	11.768808000
C	19.264564000	17.077647000	13.168759000
H	19.703812000	16.443758000	12.384071000
C	19.523530000	16.807692000	14.527943000
H	20.166764000	15.952860000	14.790898000
C	18.969656000	17.604362000	15.545704000
C	18.019291000	21.588905000	16.156024000
C	17.474231000	22.292058000	15.036142000
H	16.762016000	21.791486000	14.363470000
C	17.795870000	23.637144000	14.823934000
H	17.362895000	24.165296000	13.958775000
C	18.635646000	24.334177000	15.720561000
H	18.861921000	25.399219000	15.561753000
C	19.175221000	23.649377000	16.838061000
H	19.806456000	24.197155000	17.554586000
C	18.885631000	22.292943000	17.046413000
C	15.776653000	19.899479000	16.436772000
C	15.094760000	21.056127000	16.872642000
H	15.661620000	21.880036000	17.332015000
C	13.709384000	21.172205000	16.687552000
H	13.191838000	22.091429000	17.002454000
C	12.983551000	20.120942000	16.098591000
H	11.896170000	20.212791000	15.955052000
C	13.653304000	18.954623000	15.689509000
H	13.092157000	18.128005000	15.226571000
C	15.046264000	18.851254000	15.845498000
H	15.579913000	17.963347000	15.476762000
P	19.231155000	17.301081000	17.331658000
C	18.085883000	15.896805000	17.850941000
H	18.261378000	15.819340000	18.945422000
C	16.626283000	16.293567000	17.624771000
H	16.396078000	16.340548000	16.541548000
H	16.375907000	17.271626000	18.077173000
H	15.957715000	15.531122000	18.074145000
C	18.412101000	14.562136000	17.174210000
H	17.699375000	13.782589000	17.517902000
H	19.432094000	14.195478000	17.401732000
H	18.310764000	14.640601000	16.071627000
C	20.947231000	16.558687000	17.446718000
H	20.939305000	15.679073000	16.766949000
C	21.248503000	16.087746000	18.871091000
H	22.263504000	15.640177000	18.916746000
H	20.533498000	15.324876000	19.235647000
H	21.223644000	16.947602000	19.571832000
C	22.004217000	17.552146000	16.960354000
H	22.061925000	18.422810000	17.644436000
H	21.793881000	17.924965000	15.939489000
H	23.003328000	17.068066000	16.947258000
P	19.629674000	21.280610000	18.387861000
C	19.389858000	22.247225000	19.963758000
H	19.868271000	23.237075000	19.796750000
C	20.082365000	21.532719000	21.129267000
H	20.006763000	22.143604000	22.050897000
H	21.158819000	21.349429000	20.936090000
H	19.606075000	20.551514000	21.329687000
C	17.888238000	22.451523000	20.204045000
H	17.374274000	21.482997000	20.360533000
H	17.405342000	22.963217000	19.347332000
H	17.722937000	23.067998000	21.110107000
C	21.460841000	21.288271000	17.952514000
H	21.859874000	20.438762000	18.547077000
C	21.595132000	20.958856000	16.457959000
H	21.295261000	21.826435000	15.835899000
H	20.957810000	20.103653000	16.154499000
H	22.647156000	20.707458000	16.211012000
C	22.225488000	22.561553000	18.325595000
H	23.299111000	22.437309000	18.068687000
H	22.173036000	22.792123000	19.408141000
H	21.857190000	23.443050000	17.763344000
H	15.494600000	16.649691000	20.894818000
H	20.010768000	18.920475000	19.237024000

AP12

N	16.275698000	18.789920000	20.028066000

N	17.592647000	18.677214000	19.806956000
Fe	15.610951000	18.619846000	21.750687000
P	14.952944000	19.523187000	23.629565000
C	15.643307000	18.623210000	25.074541000
C	15.422559000	18.991853000	26.416769000
H	14.857952000	19.903860000	26.661659000
C	15.950283000	18.201968000	27.448932000
H	15.776067000	18.485211000	28.497777000
C	16.706623000	17.054890000	27.143928000
H	17.114706000	16.433184000	27.954955000
C	16.970372000	16.714618000	25.808003000
H	17.599173000	15.837667000	25.589284000
C	16.447261000	17.503494000	24.763277000
P	16.927370000	17.265190000	22.991654000
C	18.803894000	17.402594000	23.212678000
H	18.997322000	16.725319000	24.072482000
C	19.151095000	18.835035000	23.621038000
H	18.655503000	19.124584000	24.568484000
H	20.244392000	18.944583000	23.770633000
H	18.847807000	19.558463000	22.837037000
C	19.637622000	16.899440000	22.031713000
H	19.409771000	15.849009000	21.767234000
H	19.544070000	17.515864000	21.111462000
H	20.711068000	16.936855000	22.310699000
C	16.650483000	15.450946000	22.602452000
H	17.527873000	14.911786000	23.021338000
C	16.630501000	15.279937000	21.076781000
H	17.536826000	15.694975000	20.593860000
H	16.571186000	14.205342000	20.809074000
H	15.754446000	15.795779000	20.636007000
C	15.372581000	14.920190000	23.261833000
H	15.451206000	14.890286000	24.365362000
H	14.489823000	15.543579000	23.007730000
H	15.166719000	13.889095000	22.907126000
C	15.357191000	21.285460000	23.931435000
C	14.358351000	22.280661000	23.979279000
H	13.296807000	22.008249000	23.888654000
C	14.720100000	23.626969000	24.150265000
H	13.936315000	24.398412000	24.190971000
C	16.073177000	23.987128000	24.271168000
H	16.352205000	25.043352000	24.404301000
C	17.070227000	22.995770000	24.228609000
H	18.130623000	23.271256000	24.332648000
C	16.715251000	21.650096000	24.056500000
H	17.497459000	20.879584000	24.024940000
C	13.118914000	19.446639000	23.759340000
C	12.371894000	19.578559000	24.944390000
H	12.869447000	19.712702000	25.914582000
C	10.969477000	19.534344000	24.887253000
H	10.384008000	19.628092000	25.814104000
C	10.314561000	19.366136000	23.654144000
H	9.215605000	19.327648000	23.615585000
C	11.056662000	19.235692000	22.469249000
H	10.531843000	19.085583000	21.514981000
C	12.464333000	19.273136000	22.518504000
P	13.562641000	19.053265000	21.058636000
C	13.367747000	20.582210000	19.991494000
H	13.727278000	20.206200000	19.010955000
C	11.923336000	21.058414000	19.820546000
H	11.506965000	21.441183000	20.774141000
H	11.250532000	20.265545000	19.438152000
H	11.890759000	21.894219000	19.090642000
C	14.300432000	21.702685000	20.456204000
H	14.303010000	22.531803000	19.718842000
H	15.342361000	21.346976000	20.578751000
H	13.975857000	22.124655000	21.427356000
C	12.725222000	17.705420000	20.059952000
H	11.749321000	18.144528000	19.757481000
C	12.473183000	16.448533000	20.897084000
H	11.860428000	15.729886000	20.315013000
H	11.937645000	16.656601000	21.844102000

H	13.426621000	15.941034000	21.145767000
C	13.540156000	17.381920000	18.803945000
H	14.516681000	16.935278000	19.073952000
H	13.724821000	18.268608000	18.169508000
H	12.997136000	16.645293000	18.178616000
Fe	18.744481000	18.774723000	18.467992000
P	17.538256000	19.409006000	16.696721000
C	18.228608000	18.513862000	15.221475000
C	17.966764000	18.889693000	13.890302000
H	17.312469000	19.744835000	13.669788000
C	18.542195000	18.173057000	12.829190000
H	18.342530000	18.480590000	11.791733000
C	19.371703000	17.068365000	13.090569000
H	19.821528000	16.506291000	12.258387000
C	19.628585000	16.676943000	14.414269000
H	20.279168000	15.810981000	14.604851000
C	19.061215000	17.396135000	15.485998000
C	17.813463000	21.225036000	16.400495000
C	17.169499000	21.966865000	15.389621000
H	16.492246000	21.473304000	14.677882000
C	17.349725000	23.356458000	15.313486000
H	16.843470000	23.928685000	14.521577000
C	18.148637000	24.019200000	16.262518000
H	18.265573000	25.112502000	16.219265000
C	18.796875000	23.288574000	17.270694000
H	19.413216000	23.819305000	18.011077000
C	18.645033000	21.888021000	17.331703000
C	15.715728000	19.346664000	16.347306000
C	14.887453000	20.383008000	16.835052000
H	15.320838000	21.207708000	17.422251000
C	13.516327000	20.400803000	16.529548000
H	12.888227000	21.230491000	16.886442000
C	12.949514000	19.370092000	15.760713000
H	11.875339000	19.385881000	15.523194000
C	13.763301000	18.325434000	15.290772000
H	13.330328000	17.516207000	14.683531000
C	15.138579000	18.317194000	15.574498000
H	15.768169000	17.517041000	15.160043000
P	19.357571000	16.992325000	17.258929000
C	18.252491000	15.517682000	17.656829000
H	18.514708000	15.301253000	18.714852000
C	16.775429000	15.906294000	17.592213000
H	16.464817000	16.137433000	16.554988000
H	16.551268000	16.784272000	18.224802000
H	16.146782000	15.061573000	17.939001000
C	18.560347000	14.286206000	16.800148000
H	17.908385000	13.446680000	17.120129000
H	19.607962000	13.941379000	16.899175000
H	18.352638000	14.474325000	15.726906000
C	21.084977000	16.285468000	17.349931000
H	21.092533000	15.426827000	16.644344000
C	21.356885000	15.769970000	18.767459000
H	22.386405000	15.360865000	18.825478000
H	20.663676000	14.961582000	19.073038000
H	21.275903000	16.595640000	19.504280000
C	22.139892000	17.298035000	16.900419000
H	22.203194000	18.148936000	17.607922000
H	21.941082000	17.695994000	15.886742000
H	23.136347000	16.810812000	16.878677000
P	19.595212000	20.797611000	18.468810000
C	19.646150000	21.684201000	20.105553000
H	20.030118000	22.700420000	19.868916000
C	20.623035000	20.995510000	21.062741000
H	20.616993000	21.507726000	22.046586000
H	21.664650000	21.010563000	20.686135000
H	20.346583000	19.935398000	21.223026000
C	18.231113000	21.820042000	20.678341000
H	17.823690000	20.841384000	20.998948000
H	17.526177000	22.254608000	19.941609000
H	18.240487000	22.487536000	21.562340000
C	21.325539000	20.867708000	17.735289000

H	21.847399000	20.037173000	18.256775000
C	21.251369000	20.559627000	16.234087000
H	20.805515000	21.407123000	15.675338000
H	20.646241000	19.659366000	16.011054000
H	22.270492000	20.390524000	15.831971000
C	22.068015000	22.181138000	18.000816000
H	23.086230000	22.113033000	17.563643000
H	22.190232000	22.403912000	19.078329000
H	21.565125000	23.043089000	17.518051000
H	14.740370000	17.473388000	22.131825000
H	19.936189000	18.475102000	19.387214000
H	15.810584000	19.042915000	19.140692000
H	18.030869000	18.395518000	20.709144000

AP22

N	16.499728000	19.012489000	20.448899000
N	16.611629000	19.544073000	19.195977000
Fe	15.713213000	18.469139000	21.786851000
P	15.120497000	19.457686000	23.645656000
C	15.728740000	18.589779000	25.148541000
C	15.624584000	19.083932000	26.463396000
H	15.184381000	20.073643000	26.657615000
C	16.115103000	18.313337000	27.528883000
H	16.034994000	18.691840000	28.558990000
C	16.717178000	17.064356000	27.282692000
H	17.101076000	16.465328000	28.122187000
C	16.853844000	16.587661000	25.969225000
H	17.353561000	15.623196000	25.790010000
C	16.364485000	17.357438000	24.895261000
P	16.623151000	16.927339000	23.119782000
C	18.500239000	16.863322000	23.056318000
H	18.763127000	16.106506000	23.828852000
C	19.059581000	18.229270000	23.466132000
H	18.733627000	18.537453000	24.478932000
H	20.168198000	18.200068000	23.464944000
H	18.743565000	19.005568000	22.741009000
C	19.045560000	16.425379000	21.696665000
H	18.728071000	15.404623000	21.412458000
H	18.750416000	17.139239000	20.899519000
H	20.153978000	16.422011000	21.739329000
C	16.004294000	15.163946000	22.987881000
H	16.748702000	14.543944000	23.533523000
C	15.942998000	14.707964000	21.524684000
H	16.939356000	14.690709000	21.045541000
H	15.522596000	13.683595000	21.463401000
H	15.293039000	15.378192000	20.926705000
C	14.640188000	15.056832000	23.684528000
H	14.720437000	15.174080000	24.781910000
H	13.931906000	15.830364000	23.318869000
H	14.186032000	14.065629000	23.480401000
C	15.647182000	21.192933000	23.924648000
C	14.731797000	22.244015000	24.133428000
H	13.650026000	22.046551000	24.147788000
C	15.203035000	23.553355000	24.328635000
H	14.484000000	24.370689000	24.490586000
C	16.582954000	23.817429000	24.322177000
H	16.948251000	24.843788000	24.478438000
C	17.497905000	22.768075000	24.119603000
H	18.579743000	22.969638000	24.120885000
C	17.033419000	21.461061000	23.914914000
H	17.749307000	20.643576000	23.747335000
C	13.280832000	19.479715000	23.774434000
C	12.544300000	19.697973000	24.953295000
H	13.053855000	19.871036000	25.911850000
C	11.140314000	19.685768000	24.906417000
H	10.563577000	19.845072000	25.829790000
C	10.472152000	19.465097000	23.688907000
H	9.372342000	19.452752000	23.659219000
C	11.201778000	19.248974000	22.507982000
H	10.667023000	19.066068000	21.564886000
C	12.609710000	19.248255000	22.550771000
P	13.697244000	18.961920000	21.097692000
C	13.550509000	20.525803000	20.059625000
H	14.046641000	20.233304000	19.108913000
C	12.110183000	20.931541000	19.731459000
H	11.572590000	21.273112000	20.638667000
H	11.521141000	20.120306000	19.261527000
H	12.123188000	21.783673000	19.020677000
C	14.321408000	21.681646000	20.700108000
H	14.421084000	22.522333000	19.983572000
H	15.332476000	21.384657000	21.038398000
H	13.781519000	22.068371000	21.586634000
C	12.871594000	17.665659000	20.030604000
H	11.994748000	18.172890000	19.575291000
C	12.384268000	16.456296000	20.831027000
H	11.837136000	15.764903000	20.158039000
H	11.702017000	16.732902000	21.657459000
H	13.232513000	15.890187000	21.261613000
C	13.843446000	17.250225000	18.919121000
H	14.754037000	16.786625000	19.346213000
H	14.145144000	18.100567000	18.276987000
H	13.364119000	16.507377000	18.251581000
Fe	18.336313000	18.848463000	18.296271000
P	17.532343000	19.443477000	16.348056000
C	18.375858000	18.557795000	14.971386000
C	18.091223000	18.748351000	13.606872000
H	17.320053000	19.467628000	13.292225000
C	18.792840000	18.008409000	12.641235000
H	18.577981000	18.159995000	11.572736000
C	19.764748000	17.072615000	13.038874000
H	20.307539000	16.488138000	12.280731000
C	20.045867000	16.873718000	14.401098000
H	20.802506000	16.130853000	14.693001000
C	19.359117000	17.621179000	15.378196000
C	17.781627000	21.262772000	16.153951000
C	17.206133000	22.042339000	15.132689000
H	16.596393000	21.571959000	14.346561000
C	17.383333000	23.435371000	15.136249000
H	16.930222000	24.046051000	14.340815000
C	18.121346000	24.050430000	16.163605000
H	18.241598000	25.144313000	16.175302000
C	18.708145000	23.275455000	17.178435000
H	19.282589000	23.773501000	17.973352000
C	18.551867000	21.875091000	17.174554000
C	15.737421000	19.231559000	16.006926000
C	14.810396000	20.193947000	16.476812000
H	15.164994000	21.147727000	16.898794000
C	13.428385000	19.962200000	16.363017000
H	12.717882000	20.725289000	16.713964000
C	12.956463000	18.767960000	15.793152000
H	11.874522000	18.588909000	15.705246000
C	13.871839000	17.804547000	15.332506000
H	13.508223000	16.869179000	14.881006000
C	15.252887000	18.029667000	15.441294000
H	15.958723000	17.274801000	15.062973000
P	19.594780000	17.448185000	17.208983000
C	18.598886000	15.912504000	17.675750000
H	19.184733000	15.487585000	18.517483000
C	17.257507000	16.403489000	18.236155000
H	16.562517000	16.753139000	17.450566000
H	17.403236000	17.186999000	19.036028000
H	16.736737000	15.596974000	18.790828000
C	18.440829000	14.856194000	16.586535000
H	17.892768000	13.978517000	16.990418000
H	19.417447000	14.490237000	16.210854000
H	17.865309000	15.243613000	15.721805000
C	21.351581000	16.822870000	17.458517000
H	21.299238000	15.787449000	17.052747000
C	21.641944000	16.749380000	18.962731000
H	22.653404000	16.326047000	19.131616000
H	20.921012000	16.107648000	19.504874000
H	21.605902000	17.756762000	19.425132000

C	22.455923000	17.577473000	16.712430000
H	22.680986000	18.547733000	17.193989000
H	22.217341000	17.766204000	15.649237000
H	23.388518000	16.977126000	16.741800000
P	19.402889000	20.705171000	18.340667000
C	19.462282000	21.632968000	19.969865000
H	19.979402000	22.584383000	19.724622000
C	20.295216000	20.855459000	20.993150000
H	20.327475000	21.401015000	21.958652000
H	21.340837000	20.694941000	20.663402000
H	19.848344000	19.857964000	21.171954000
C	18.067921000	21.966580000	20.511216000
H	17.591491000	21.076610000	20.970897000
H	17.398959000	22.395124000	19.734551000
H	18.140068000	22.731664000	21.309316000
C	21.176443000	20.781948000	17.699873000
H	21.645374000	19.891930000	18.171661000
C	21.193566000	20.620904000	16.174854000
H	20.775704000	21.518272000	15.676017000
H	20.618459000	19.744889000	15.823306000
H	22.235481000	20.497953000	15.817531000
C	21.958757000	22.034895000	18.110503000
H	22.983952000	21.971769000	17.689239000
H	22.066581000	22.148591000	19.205621000
H	21.500595000	22.959007000	17.702312000
H	14.800512000	17.284377000	21.892317000
H	19.158103000	18.589824000	19.575576000
H	16.623062000	20.580895000	19.228175000
H	15.831856000	19.243764000	18.576881000

AP122

N	17.017774000	19.105301000	20.509032000
N	16.789630000	19.881517000	19.337998000
Fe	15.840370000	18.575492000	21.898035000
P	15.042591000	19.414492000	23.729128000
C	15.695969000	18.529948000	25.193798000
C	15.460577000	18.940969000	26.520204000
H	14.907717000	19.868338000	26.731925000
C	15.957453000	18.164624000	27.578323000
H	15.772058000	18.476860000	28.616743000
C	16.697756000	16.996864000	27.315507000
H	17.084261000	16.392442000	28.149528000
C	16.972986000	16.611074000	25.993857000
H	17.587415000	15.717246000	25.806571000
C	16.473483000	17.381272000	24.925551000
P	16.956967000	17.105453000	23.175365000
C	18.816122000	17.328945000	23.287716000
H	19.114566000	16.619519000	24.090642000
C	19.132889000	18.753898000	23.745404000
H	18.663113000	18.998761000	24.717589000
H	20.227723000	18.882327000	23.861137000
H	18.793882000	19.502905000	23.000637000
C	19.534802000	16.943008000	21.994825000
H	19.450212000	15.861253000	21.781709000
H	19.151689000	17.498462000	21.114444000
H	20.615479000	17.170311000	22.091647000
C	16.642680000	15.315200000	22.761459000
H	17.494028000	14.750143000	23.198882000
C	16.652781000	15.146924000	21.235691000
H	17.593290000	15.511733000	20.782450000
H	16.538687000	14.077894000	20.966240000
H	15.818694000	15.707000000	20.767784000
C	15.330484000	14.840288000	23.396608000
H	15.390490000	14.800564000	24.500692000
H	14.481221000	15.504378000	23.132181000
H	15.085258000	13.822709000	23.030328000
C	15.381232000	21.185828000	24.021363000
C	14.347125000	22.135685000	24.155337000
H	13.293193000	21.823120000	24.137187000
C	14.666773000	23.493745000	24.316287000
H	13.857770000	24.231398000	24.425657000
C	16.009100000	23.908850000	24.335527000
H	16.253266000	24.974962000	24.456548000
C	17.041687000	22.961676000	24.206780000
H	18.094198000	23.281013000	24.234346000
C	16.730521000	21.604427000	24.049264000
H	17.539239000	20.866556000	23.957647000
C	13.213817000	19.252184000	23.753236000
C	12.414439000	19.340317000	24.906890000
H	12.865508000	19.492198000	25.897410000
C	11.019643000	19.221900000	24.790339000
H	10.392714000	19.280172000	25.692354000
C	10.425873000	19.022149000	23.531838000
H	9.333404000	18.923929000	23.448398000
C	11.220604000	18.934608000	22.377091000
H	10.744673000	18.759225000	21.401865000
C	12.620162000	19.046910000	22.485808000
P	13.773262000	18.906634000	21.066264000
C	13.581682000	20.502004000	20.082895000
H	14.074644000	20.250806000	19.116896000
C	12.123896000	20.853568000	19.774755000
H	11.559518000	21.076056000	20.701849000
H	11.593835000	20.055491000	19.220037000
H	12.092791000	21.769696000	19.149625000
C	14.315437000	21.669062000	20.748678000
H	14.428232000	22.510469000	20.035485000
H	15.319217000	21.397655000	21.134291000
H	13.740745000	22.050278000	21.614422000
C	13.074760000	17.592428000	19.947706000
H	12.114187000	18.002797000	19.568391000
C	12.798201000	16.285515000	20.695521000
H	12.273020000	15.579644000	20.020803000
H	12.161210000	16.433427000	21.588983000
H	13.736022000	15.793237000	21.018594000
C	14.038614000	17.399288000	18.773966000
H	15.031560000	17.062984000	19.134611000
H	14.172053000	18.320643000	18.172722000
H	13.652477000	16.625039000	18.083106000
Fe	18.238482000	18.955776000	18.390845000
P	17.488415000	19.556863000	16.387621000
C	18.216105000	18.537279000	15.055909000
C	18.064294000	18.809935000	13.684494000
H	17.501610000	19.691291000	13.343530000
C	18.645007000	17.943968000	12.744419000
H	18.539035000	18.155670000	11.670210000
C	19.361479000	16.811860000	13.171948000
H	19.813119000	16.136036000	12.430684000
C	19.511554000	16.536379000	14.541496000
H	20.085356000	15.652521000	14.854793000
C	18.939390000	17.401830000	15.494288000
C	17.873558000	21.341481000	16.170246000
C	17.329244000	22.124314000	15.134232000
H	16.681959000	21.671331000	14.368890000
C	17.587360000	23.503476000	15.100784000
H	17.164946000	24.116639000	14.291046000
C	18.361271000	24.104188000	16.109710000
H	18.542105000	25.189203000	16.091927000
C	18.906568000	23.328110000	17.145780000
H	19.508618000	23.816374000	17.925479000
C	18.672881000	21.938698000	17.174615000
C	15.677624000	19.533861000	16.094068000
C	14.884225000	20.557360000	16.664263000
H	15.354956000	21.403871000	17.188694000
C	13.489480000	20.534323000	16.507358000
H	12.880770000	21.345294000	16.931739000
C	12.876299000	19.487478000	15.797122000
H	11.782603000	19.469431000	15.680565000
C	13.664063000	18.477868000	15.218032000
H	13.190860000	17.668196000	14.643016000
C	15.060344000	18.499462000	15.359709000
H	15.667667000	17.717089000	14.884743000
P	19.071355000	17.160521000	17.317470000

C	17.894140000	15.727064000	17.709898000
H	17.746218000	15.813380000	18.808062000
C	16.553461000	15.960744000	17.011268000
H	16.650299000	15.840445000	15.913980000
H	16.141372000	16.966280000	17.211692000
H	15.809085000	15.219175000	17.363151000
C	18.446622000	14.334950000	17.389547000
H	17.667460000	13.582098000	17.630740000
H	19.345395000	14.070396000	17.977570000
H	18.681017000	14.223877000	16.311130000
C	20.768576000	16.419239000	17.575259000
H	20.684453000	15.440817000	17.057836000
C	21.006064000	16.166725000	19.064778000
H	21.948866000	15.599851000	19.203451000
H	20.195136000	15.577194000	19.534734000
H	21.109291000	17.123677000	19.615593000
C	21.922718000	17.190056000	16.935890000
H	22.127141000	18.136187000	17.469962000
H	21.753985000	17.414622000	15.866259000
H	22.842919000	16.574789000	17.004088000
P	19.497125000	20.775308000	18.338904000
C	19.713196000	21.704439000	19.946987000
H	20.334829000	22.577783000	19.659654000
C	20.508938000	20.839410000	20.929994000
H	20.711179000	21.402314000	21.863169000
H	21.483329000	20.510268000	20.519411000
H	19.952398000	19.919973000	21.207748000
C	18.414524000	22.249611000	20.556669000
H	17.830458000	21.470265000	21.089346000
H	17.765779000	22.759584000	19.814934000
H	18.660781000	23.007896000	21.326044000
C	21.231173000	20.678557000	17.615721000
H	21.688874000	19.822275000	18.154911000
C	21.150784000	20.357337000	16.119352000
H	20.756676000	21.219816000	15.545893000
H	20.509826000	19.484338000	15.899761000
H	22.163940000	20.132441000	15.731534000
C	22.084188000	21.927133000	17.864323000
H	23.074409000	21.774687000	17.387684000
H	22.268863000	22.125679000	18.937346000
H	21.640888000	22.833511000	17.404363000
H	14.900746000	17.490945000	22.357485000
H	19.633742000	18.719157000	18.986225000
H	16.826537000	20.903304000	19.512186000
H	15.850891000	19.662499000	18.964866000
H	17.964409000	19.371241000	20.839872000

AP222

N	16.359217000	18.372204000	19.476412000
N	15.427017000	17.598618000	18.623240000
Fe	15.945402000	18.742687000	21.183780000
P	15.614771000	19.744172000	23.245703000
C	15.918202000	18.478402000	24.544145000
C	15.426404000	18.539203000	25.861543000
H	14.705910000	19.313991000	26.157802000
C	15.868172000	17.601687000	26.807000000
H	15.478061000	17.640746000	27.834900000
C	16.813543000	16.621549000	26.449691000
H	17.167137000	15.898414000	27.199503000
C	17.296411000	16.550762000	25.134721000
H	18.016633000	15.765904000	24.859231000
C	16.836555000	17.474888000	24.173370000
P	17.263228000	17.375844000	22.392784000
C	19.141032000	17.557009000	22.253660000
H	19.515902000	16.512190000	22.234023000
C	19.778912000	18.297334000	23.431994000
H	19.619363000	17.773954000	24.392924000
H	20.873727000	18.370576000	23.268354000
H	19.393662000	19.330317000	23.531658000
C	19.499760000	18.237269000	20.934421000
H	19.054820000	17.683992000	20.065815000
H	19.150487000	19.281056000	20.950719000
H	20.592232000	18.244609000	20.765394000
C	17.056490000	15.528971000	22.078876000
H	17.929965000	15.091957000	22.609434000
C	17.186573000	15.246444000	20.587248000
H	18.059021000	15.752702000	20.139217000
H	17.288040000	14.159071000	20.400497000
H	16.275500000	15.590169000	20.061572000
C	15.778509000	14.912347000	22.645951000
H	15.643847000	15.103499000	23.726694000
H	14.876469000	15.273583000	22.116704000
H	15.816177000	13.812853000	22.502603000
C	16.559993000	21.168665000	23.936126000
C	16.167349000	22.481784000	23.598494000
H	15.297587000	22.651729000	22.947201000
C	16.867059000	23.581302000	24.118300000
H	16.550856000	24.601393000	23.853459000
C	17.962386000	23.378563000	24.976730000
H	18.510993000	24.241556000	25.383037000
C	18.343721000	22.072389000	25.329064000
H	19.186042000	21.907729000	26.017620000
C	17.642491000	20.969076000	24.816813000
H	17.931440000	19.954876000	25.124024000
C	13.904104000	20.359781000	23.471899000
C	13.459281000	21.064460000	24.610333000
H	14.157524000	21.318452000	25.420773000
C	12.119182000	21.467363000	24.691739000
H	11.767535000	22.017302000	25.577235000
C	11.228526000	21.176525000	23.641365000
H	10.177459000	21.494901000	23.706475000
C	11.672959000	20.487072000	22.503566000
H	10.960193000	20.265904000	21.697637000
C	13.022604000	20.087196000	22.412174000
P	13.709217000	19.090188000	21.025759000
C	13.068786000	19.943125000	19.470376000
H	13.779240000	19.605115000	18.688522000
C	11.654732000	19.560947000	19.022621000
H	10.886000000	19.837058000	19.769822000
H	11.544616000	18.483037000	18.797307000
H	11.405580000	20.112220000	18.093205000
C	13.244043000	21.459849000	19.621702000
H	13.120633000	21.961274000	18.640267000
H	14.241807000	21.729644000	20.015563000
H	12.487273000	21.888310000	20.306721000
C	12.651674000	17.545647000	21.263997000
H	11.614755000	17.901729000	21.090768000
C	12.764246000	17.057102000	22.714635000
H	12.233102000	16.090154000	22.825885000
H	12.313256000	17.771185000	23.428483000
H	13.817406000	16.895129000	23.022505000
C	12.960730000	16.440518000	20.253573000
H	13.987040000	16.036021000	20.387962000
H	12.815176000	16.758829000	19.199260000
H	12.277968000	15.579837000	20.402552000
Fe	17.978994000	18.810610000	18.648712000
P	17.345888000	19.512921000	16.686887000
C	18.351230000	18.680825000	15.393149000
C	18.453259000	19.152131000	14.070685000
H	17.910496000	20.051243000	13.748917000
C	19.268425000	18.471754000	13.153602000
H	19.354935000	18.843355000	12.121943000
C	19.979455000	17.328004000	13.555526000
H	20.626960000	16.800941000	12.839234000
C	19.873540000	16.852065000	14.872288000
H	20.445573000	15.960424000	15.164834000
C	19.056722000	17.527095000	15.800488000
C	17.647003000	21.311529000	16.465584000
C	17.177170000	22.046158000	15.357714000
H	16.572646000	21.564441000	14.575467000
C	17.457650000	23.416911000	15.271447000
H	17.094771000	23.994069000	14.408156000

C	18.181469000	24.055557000	16.295085000
H	18.389995000	25.133847000	16.231682000
C	18.625981000	23.329494000	17.410201000
H	19.166847000	23.856237000	18.209001000
C	18.361552000	21.946337000	17.497354000
C	15.608916000	19.422108000	16.083596000
C	14.690230000	20.368847000	16.599012000
H	15.013100000	21.081664000	17.373000000
C	13.391190000	20.442544000	16.076380000
H	12.694050000	21.206403000	16.449687000
C	12.988535000	19.561041000	15.058059000
H	11.967561000	19.621462000	14.652957000
C	13.896670000	18.622219000	14.540733000
H	13.593886000	17.947686000	13.726412000
C	15.207975000	18.560388000	15.037512000
H	15.924456000	17.863882000	14.582563000
P	18.770677000	16.956584000	17.531135000
C	17.694708000	15.406529000	17.219958000
H	17.204438000	15.213739000	18.195752000
C	16.619430000	15.681516000	16.163181000
H	17.060093000	15.733884000	15.148214000
H	16.065573000	16.625174000	16.313382000
H	15.872174000	14.862977000	16.169702000
C	18.476621000	14.144785000	16.831443000
H	17.752367000	13.319973000	16.668387000
H	19.185130000	13.805604000	17.607288000
H	19.028466000	14.282138000	15.880261000
C	20.463657000	16.249713000	17.986127000
H	20.628528000	15.543206000	17.145889000
C	20.538424000	15.428673000	19.278892000
H	21.517731000	14.908720000	19.305164000
H	19.760772000	14.646928000	19.356050000
H	20.491704000	16.055106000	20.187328000
C	21.557685000	17.316133000	17.920833000
H	21.459637000	18.067151000	18.728471000
H	21.568378000	17.847919000	16.951224000
H	22.547453000	16.830395000	18.042833000
P	18.873400000	20.890506000	18.912522000
C	18.303306000	21.942375000	20.348868000
H	18.708547000	22.947132000	20.103686000
C	18.851026000	21.507123000	21.703736000
H	18.676976000	22.304139000	22.450096000
H	19.935463000	21.292041000	21.691765000
H	18.315300000	20.606963000	22.061585000
C	16.778823000	22.000354000	20.320535000
H	16.337719000	21.025194000	20.648741000
H	16.380760000	22.222024000	19.311180000
H	16.403068000	22.778618000	21.008925000
C	20.752812000	20.979420000	18.846522000
H	21.078437000	20.114420000	19.462780000
C	21.211703000	20.766699000	17.395863000
H	21.037304000	21.677801000	16.789955000
H	20.692168000	19.928147000	16.889993000
H	22.297839000	20.547020000	17.374980000
C	21.363162000	22.260766000	19.426820000
H	22.468190000	22.192207000	19.354142000
H	21.116707000	22.423924000	20.492124000
H	21.058671000	23.158605000	18.853933000
H	15.252029000	17.484624000	21.477208000
H	19.394988000	18.906506000	18.063292000
H	15.144426000	18.162600000	17.785302000
H	14.574235000	17.271091000	19.123313000
H	15.894216000	16.746951000	18.259459000

B

N	16.698512000	18.624059000	20.520151000
N	17.477721000	18.950590000	19.711374000
Fe	15.559272000	18.024511000	21.753813000
P	15.043173000	19.226339000	23.436451000
C	15.777063000	18.615002000	25.034096000
C	15.656968000	19.227686000	26.297301000
H	15.093341000	20.167859000	26.406121000
C	16.287195000	18.654474000	27.418802000
H	16.185360000	19.126897000	28.408902000
C	17.057362000	17.486979000	27.270754000
H	17.557203000	17.042617000	28.146403000
C	17.205467000	16.894338000	26.000852000
H	17.831588000	15.993816000	25.892549000
C	16.564152000	17.454625000	24.878738000
P	16.672353000	16.807956000	23.125842000
C	18.565080000	16.700878000	22.962025000
H	18.946064000	16.029578000	23.765367000
C	19.136895000	18.112966000	23.143887000
H	18.910568000	18.532886000	24.144478000
H	20.241036000	18.111419000	23.015476000
H	18.706479000	18.786875000	22.373869000
C	18.977447000	16.157244000	21.590619000
H	18.600776000	15.134905000	21.398055000
H	18.609724000	16.823985000	20.784508000
H	20.084427000	16.123422000	21.514121000
C	16.141021000	15.009164000	23.457185000
H	16.851917000	14.586884000	24.202078000
C	16.165172000	14.160244000	22.181520000
H	17.190820000	13.868735000	21.882057000
H	15.580199000	13.224591000	22.316384000
H	15.718511000	14.737995000	21.341589000
C	14.730926000	15.054536000	24.062117000
H	14.718383000	15.561334000	25.048144000
H	14.052685000	15.623446000	23.392643000
H	14.322338000	14.029351000	24.197634000
C	15.500264000	21.030796000	23.627765000
C	14.580994000	22.084861000	23.813145000
H	13.507018000	21.863397000	23.907473000
C	15.024329000	23.418749000	23.880019000
H	14.292800000	24.230779000	24.021821000
C	16.394139000	23.716019000	23.776392000
H	16.742532000	24.759162000	23.835425000
C	17.317866000	22.668899000	23.587630000
H	18.392680000	22.891944000	23.500987000
C	16.872167000	21.343111000	23.497281000
H	17.586998000	20.525981000	23.312144000
C	13.194532000	19.281268000	23.672179000
C	12.496503000	19.578747000	24.858691000
H	13.040051000	19.888954000	25.763440000
C	11.091381000	19.458776000	24.901723000
H	10.552490000	19.651955000	25.843083000
C	10.383645000	19.093411000	23.746411000
H	9.286346000	18.998930000	23.777092000
C	11.077319000	18.840533000	22.543057000
H	10.507753000	18.564045000	21.642600000
C	12.483772000	18.904190000	22.504582000
P	13.570774000	18.432765000	21.050571000
C	13.325578000	19.906387000	19.873068000
H	13.791339000	19.534377000	18.935016000
C	11.877756000	20.312942000	19.591936000
H	11.378708000	20.687546000	20.510313000
H	11.265342000	19.481618000	19.187196000
H	11.843095000	21.132360000	18.842864000
C	14.160805000	21.090705000	20.361029000
H	14.146367000	21.912377000	19.613548000
H	15.213152000	20.792612000	20.531021000
H	13.771619000	21.498536000	21.316858000
C	12.528186000	17.117597000	20.184185000
H	11.538274000	17.571628000	19.960027000
C	12.327238000	15.896243000	21.084502000
H	11.669784000	15.147698000	20.590648000
H	11.867005000	16.162213000	22.057797000
H	13.302304000	15.413953000	21.297539000
C	13.219950000	16.731874000	18.871894000
H	14.256812000	16.398247000	19.084060000
H	13.289756000	17.577147000	18.158409000
H	12.679927000	15.904261000	18.362286000

Fe	18.695992000	19.234634000	18.429035000
P	17.651464000	19.828582000	16.650756000
C	18.142870000	18.743917000	15.201973000
C	17.808856000	18.979845000	13.854226000
H	17.150579000	19.819133000	13.583613000
C	18.315616000	18.144646000	12.840224000
H	18.068820000	18.347889000	11.785999000
C	19.140485000	17.057304000	13.175196000
H	19.544754000	16.406147000	12.383773000
C	19.446309000	16.794746000	14.525032000
H	20.083782000	15.932062000	14.773966000
C	18.955623000	17.634967000	15.545146000
C	18.130475000	21.555923000	16.106963000
C	17.564047000	22.263468000	15.027159000
H	16.782382000	21.796913000	14.408631000
C	17.960669000	23.586374000	14.761673000
H	17.514495000	24.135405000	13.917145000
C	18.910514000	24.212697000	15.588521000
H	19.209628000	25.255142000	15.394744000
C	19.472715000	23.510079000	16.670781000
H	20.202632000	24.017556000	17.319964000
C	19.097334000	22.175683000	16.928266000
C	15.813294000	19.961859000	16.382092000
C	15.132524000	21.103928000	16.863557000
H	15.707858000	21.919378000	17.328120000
C	13.740487000	21.214775000	16.742781000
H	13.232516000	22.123097000	17.102942000
C	12.991023000	20.167133000	16.174606000
H	11.896297000	20.248778000	16.091858000
C	13.653020000	19.012128000	15.723517000
H	13.078296000	18.182977000	15.280823000
C	15.051926000	18.915115000	15.820066000
H	15.562105000	18.017359000	15.442280000
P	19.273417000	17.449525000	17.376783000
C	18.159979000	15.953807000	17.808192000
H	18.328629000	15.862018000	18.902814000
C	16.689780000	16.320202000	17.599624000
H	16.445506000	16.376583000	16.517829000
H	16.435476000	17.289638000	18.068804000
H	16.033040000	15.543953000	18.046329000
C	18.493855000	14.624828000	17.124484000
H	17.817490000	13.821140000	17.491633000
H	19.531391000	14.282346000	17.309923000
H	18.348642000	14.691206000	16.025328000
C	20.974572000	16.624178000	17.407881000
H	20.918101000	15.728769000	16.751541000
C	21.302545000	16.175753000	18.834629000
H	22.331821000	15.759281000	18.891324000
H	20.607203000	15.398561000	19.209064000
H	21.235713000	17.042483000	19.525472000
C	22.048005000	17.568462000	16.869729000
H	22.141395000	18.454105000	17.527886000
H	21.815004000	17.928623000	15.848288000
H	23.036530000	17.060594000	16.834547000
P	19.774435000	21.085411000	18.288026000
C	19.570567000	22.220591000	19.791888000
H	20.115961000	23.168295000	19.587198000
C	20.159701000	21.542925000	21.033420000
H	20.057456000	22.196099000	21.926522000
H	21.237381000	21.301992000	20.918745000
H	19.628615000	20.590154000	21.235714000
C	18.079106000	22.520404000	19.980084000
H	17.511632000	21.577459000	20.119053000
H	17.651792000	23.042958000	19.099692000
H	17.911297000	23.158914000	20.872295000
C	21.638259000	21.158076000	17.898804000
H	22.006112000	20.262589000	18.446293000
C	21.823322000	20.917288000	16.394005000
H	21.628344000	21.845229000	15.817708000
H	21.121336000	20.141404000	16.025048000
H	22.858226000	20.586454000	16.162342000
C	22.430057000	22.380721000	18.372938000
H	23.506711000	22.264618000	18.117143000
H	22.371761000	22.531824000	19.468830000
H	22.083826000	23.314109000	17.883675000
H	15.187044000	16.638828000	21.205380000
H	19.834024000	18.867465000	19.402518000

BP2

N	16.569752000	18.441886000	20.554848000
N	17.352635000	18.961990000	19.791178000
Fe	15.456595000	17.888517000	21.782871000
P	14.941058000	19.089628000	23.460756000
C	15.606945000	18.396211000	25.051487000
C	15.451828000	18.959856000	26.333517000
H	14.906062000	19.907554000	26.462372000
C	16.021201000	18.327056000	27.451824000
H	15.894042000	18.764598000	28.454263000
C	16.763052000	17.142343000	27.288274000
H	17.210458000	16.647383000	28.164322000
C	16.956289000	16.602710000	26.004782000
H	17.567304000	15.693930000	25.884396000
C	16.382772000	17.228009000	24.879608000
P	16.705662000	16.720370000	23.115001000
C	18.591606000	16.889151000	23.145277000
H	18.928583000	16.319864000	24.040155000
C	18.977126000	18.362385000	23.305895000
H	18.486614000	18.841170000	24.176904000
H	20.075451000	18.460846000	23.439999000
H	18.703504000	18.929448000	22.392696000
C	19.249099000	16.306291000	21.895377000
H	19.081701000	15.217041000	21.788030000
H	18.875402000	16.820172000	20.986663000
H	20.346244000	16.466995000	21.937750000
C	16.455569000	14.845489000	23.123720000
H	17.348941000	14.398988000	23.614838000
C	16.356741000	14.350652000	21.673221000
H	17.249910000	14.599937000	21.068841000
H	16.221549000	13.248262000	21.644390000
H	15.488172000	14.828646000	21.174861000
C	15.194303000	14.464318000	23.906924000
H	15.279397000	14.687055000	24.988213000
H	14.315018000	15.018150000	23.513440000
H	14.985739000	13.378380000	23.799872000
C	15.428151000	20.873107000	23.630404000
C	14.607736000	21.884060000	24.172539000
H	13.597940000	21.642480000	24.536064000
C	15.067846000	23.211708000	24.239040000
H	14.415597000	23.993262000	24.659735000
C	16.352810000	23.541299000	23.774418000
H	16.713221000	24.580049000	23.831931000
C	17.172829000	22.537424000	23.226584000
H	18.177429000	22.786265000	22.854102000
C	16.705992000	21.218103000	23.140929000
H	17.324189000	20.432313000	22.682726000
C	13.093099000	19.140304000	23.661485000
C	12.365780000	19.385645000	24.841830000
H	12.887111000	19.590597000	25.788144000
C	10.959447000	19.350113000	24.825089000
H	10.395920000	19.528438000	25.754104000
C	10.277832000	19.082166000	23.626147000
H	9.177227000	19.051985000	23.611514000
C	10.999877000	18.833692000	22.444305000
H	10.451005000	18.604035000	21.518890000
C	12.408650000	18.844938000	22.455278000
P	13.498025000	18.415676000	21.001633000
C	13.357944000	19.935572000	19.887082000
H	13.839326000	19.594091000	18.945100000
C	11.927999000	20.392114000	19.589735000
H	11.419172000	20.747161000	20.509766000
H	11.304065000	19.595430000	19.137120000
H	11.939712000	21.240176000	18.875045000

C	14.206282000	21.075161000	20.454737000
H	14.239118000	21.925370000	19.742012000
H	15.244645000	20.746273000	20.647174000
H	13.796360000	21.454098000	21.413694000
C	12.452107000	17.150267000	20.079010000
H	11.477280000	17.639135000	19.863202000
C	12.204790000	15.902865000	20.931500000
H	11.501043000	15.216560000	20.413843000
H	11.772642000	16.147363000	21.922938000
H	13.152792000	15.355840000	21.107842000
C	13.142700000	16.800321000	18.757455000
H	14.158676000	16.403298000	18.953318000
H	13.255081000	17.675601000	18.087520000
H	12.568366000	16.027170000	18.204141000
Fe	18.649931000	19.429072000	18.547154000
P	17.597151000	19.922365000	16.719368000
C	18.080784000	18.742340000	15.352783000
C	17.723300000	18.888411000	13.998581000
H	17.077975000	19.720146000	13.679000000
C	18.188683000	17.970154000	13.041580000
H	17.916742000	18.096693000	11.982041000
C	19.004472000	16.895661000	13.438067000
H	19.373972000	16.177837000	12.689080000
C	19.347376000	16.733646000	14.792182000
H	19.983060000	15.885075000	15.087941000
C	18.891257000	17.654032000	15.757897000
C	18.115775000	21.590956000	16.060900000
C	17.520775000	22.255063000	14.969445000
H	16.690724000	21.786510000	14.419327000
C	17.956651000	23.539627000	14.604479000
H	17.486932000	24.057649000	13.753767000
C	18.977105000	24.170909000	15.339457000
H	19.306322000	25.185834000	15.066923000
C	19.570242000	23.511479000	16.429730000
H	20.355185000	24.022604000	17.007404000
C	19.150032000	22.214608000	16.790492000
C	15.763858000	20.070340000	16.474065000
C	15.119470000	21.245293000	16.924788000
H	15.713905000	22.053301000	17.378614000
C	13.734782000	21.402846000	16.775012000
H	13.253709000	22.336988000	17.103838000
C	12.961644000	20.371718000	16.209300000
H	11.873133000	20.491959000	16.099324000
C	13.588129000	19.186099000	15.791312000
H	12.992443000	18.371927000	15.349967000
C	14.981193000	19.039367000	15.915087000
H	15.463625000	18.117889000	15.560034000
P	19.229107000	17.568052000	17.588254000
C	18.064358000	16.151805000	18.119361000
H	18.089828000	16.236370000	19.227218000
C	16.635035000	16.458601000	17.670396000
H	16.531914000	16.367929000	16.568841000
H	16.312149000	17.471345000	17.975125000
H	15.934650000	15.731910000	18.130317000
C	18.471201000	14.734543000	17.706192000
H	17.708211000	14.006077000	18.057295000
H	19.441847000	14.414452000	18.133030000
H	18.528912000	14.631440000	16.602012000
C	20.905930000	16.702002000	17.655412000
H	20.764955000	15.740288000	17.118398000
C	21.273196000	16.400379000	19.110144000
H	22.278366000	15.930677000	19.168698000
H	20.554697000	15.709131000	19.590212000
H	21.287753000	17.335736000	19.707845000
C	22.018895000	17.477638000	16.952992000
H	22.250214000	18.416585000	17.490879000
H	21.762610000	17.732530000	15.906894000
H	22.948228000	16.868868000	16.931409000
P	19.860232000	21.191837000	18.178023000
C	19.895365000	22.456512000	19.581051000
H	20.552301000	23.286068000	19.242755000
C	20.484690000	21.827495000	20.847618000
H	20.584754000	22.584795000	21.654421000
H	21.488280000	21.387204000	20.674877000
H	19.833785000	21.006476000	21.214279000
C	18.490840000	23.015045000	19.822470000
H	17.785795000	22.207269000	20.115502000
H	18.077614000	23.504732000	18.918125000
H	18.503440000	23.767974000	20.637726000
C	21.669649000	21.048012000	17.623125000
H	22.014028000	20.171239000	18.213196000
C	21.685255000	20.678295000	16.133189000
H	21.475599000	21.567338000	15.504322000
H	20.920773000	19.912325000	15.892587000
H	22.676913000	20.279637000	15.833964000
C	22.598367000	22.230269000	17.913688000
H	23.627192000	21.992469000	17.565841000
H	22.668424000	22.469973000	18.992983000
H	22.282516000	23.148151000	17.376821000
H	14.530999000	16.745811000	22.239426000
H	19.810333000	19.126617000	19.553892000
H	17.543444000	20.072949000	19.960656000

BP22

N	16.636209000	18.832460000	20.228965000
N	16.290940000	19.256943000	18.991430000
Fe	15.870800000	18.487304000	21.768715000
P	15.219927000	19.493667000	23.557658000
C	15.883887000	18.683896000	25.082840000
C	15.665033000	19.126365000	26.402529000
H	15.117789000	20.061982000	26.594609000
C	16.170523000	18.381765000	27.480266000
H	15.996484000	18.723152000	28.512189000
C	16.904436000	17.204696000	27.240606000
H	17.295852000	16.618714000	28.086346000
C	17.169102000	16.792158000	25.924288000
H	17.788194000	15.897866000	25.752366000
C	16.667634000	17.534776000	24.836133000
P	17.183325000	17.268329000	23.072749000
C	19.049761000	17.447986000	23.339448000
H	19.314732000	16.647343000	24.065749000
C	19.393406000	18.800494000	23.962625000
H	18.926753000	18.942058000	24.956849000
H	20.492454000	18.893838000	24.090338000
H	19.070372000	19.627847000	23.305723000
C	19.790253000	17.197546000	22.024201000
H	19.739198000	16.131703000	21.732541000
H	19.373059000	17.797356000	21.187519000
H	20.865989000	17.454739000	22.128673000
C	17.026629000	15.413011000	22.760362000
H	17.933688000	14.941926000	23.197827000
C	17.019593000	15.174971000	21.242663000
H	17.878993000	15.658895000	20.740677000
H	17.052502000	14.088517000	21.016018000
H	16.103990000	15.600969000	20.785972000
C	15.780892000	14.825345000	23.432336000
H	15.864667000	14.808731000	24.535994000
H	14.874028000	15.411445000	23.175204000
H	15.614658000	13.782156000	23.089333000
C	15.561476000	21.283910000	23.832439000
C	14.546129000	22.255348000	23.946904000
H	13.488356000	21.954141000	23.943116000
C	14.880593000	23.615403000	24.067528000
H	14.080042000	24.366299000	24.153762000
C	16.227485000	24.015455000	24.080657000
H	16.486338000	25.081234000	24.175333000
C	17.244455000	23.048443000	23.975787000
H	18.301873000	23.354395000	23.991018000
C	16.912233000	21.692508000	23.846747000
H	17.704173000	20.936911000	23.752498000
C	13.376986000	19.369059000	23.743884000
C	12.643038000	19.567110000	24.928911000

H	13.148111000	19.858578000	25.860296000
C	11.250416000	19.381096000	24.930183000
H	10.680884000	19.525102000	25.861013000
C	10.587792000	19.001448000	23.749734000
H	9.498476000	18.843589000	23.754974000
C	11.313703000	18.814980000	22.561162000
H	10.783289000	18.503483000	21.649769000
C	12.710976000	18.999742000	22.553016000
P	13.805310000	18.766941000	21.080653000
C	13.359271000	20.239131000	19.975794000
H	13.860699000	19.993905000	19.016570000
C	11.870533000	20.425507000	19.673961000
H	11.293208000	20.658170000	20.591480000
H	11.414812000	19.539258000	19.189376000
H	11.732641000	21.280060000	18.978149000
C	13.989703000	21.513000000	20.544905000
H	13.981020000	22.328256000	19.791710000
H	15.035579000	21.351226000	20.875174000
H	13.423449000	21.873563000	21.426968000
C	12.999806000	17.336512000	20.167868000
H	11.973704000	17.675008000	19.910985000
C	12.903947000	16.079969000	21.036786000
H	12.300387000	15.306009000	20.518057000
H	12.428778000	16.277728000	22.018006000
H	13.908442000	15.653534000	21.224414000
C	13.774979000	17.055431000	18.877653000
H	14.828823000	16.805323000	19.119948000
H	13.751888000	17.909107000	18.168639000
H	13.342679000	16.189105000	18.336934000
Fe	18.157154000	18.939125000	18.337929000
P	17.601031000	19.671698000	16.350318000
C	18.241047000	18.600883000	14.993704000
C	18.146221000	18.891045000	13.619321000
H	17.643204000	19.806194000	13.274046000
C	18.699592000	18.004860000	12.680552000
H	18.634527000	18.234107000	11.606042000
C	19.340810000	16.830561000	13.113876000
H	19.780274000	16.140218000	12.377694000
C	19.423255000	16.531457000	14.484814000
H	19.932426000	15.610423000	14.804844000
C	18.869638000	17.411636000	15.436377000
C	18.154209000	21.413749000	16.055556000
C	17.789855000	22.201743000	14.946567000
H	17.183157000	21.778682000	14.131856000
C	18.166812000	23.553605000	14.901333000
H	17.881907000	24.172946000	14.037167000
C	18.882061000	24.122694000	15.971613000
H	19.155725000	25.188652000	15.944262000
C	19.240805000	23.338880000	17.081223000
H	19.787119000	23.802164000	17.916508000
C	18.887245000	21.974827000	17.124218000
C	15.796207000	19.852761000	16.010547000
C	15.115328000	20.926523000	16.635367000
H	15.687854000	21.714640000	17.151515000
C	13.715845000	21.013419000	16.576416000
H	13.201636000	21.860167000	17.055355000
C	12.974551000	20.026820000	15.901893000
H	11.876708000	20.090549000	15.863862000
C	13.644340000	18.966943000	15.265554000
H	13.072701000	18.201140000	14.719243000
C	15.046030000	18.880287000	15.315150000
H	15.558038000	18.054589000	14.801044000
P	18.866675000	17.131573000	17.276927000
C	17.621684000	15.703022000	17.480880000
H	17.435828000	15.710465000	18.576831000
C	16.318806000	16.062264000	16.764694000
H	16.441501000	16.013246000	15.663476000
H	15.974599000	17.083266000	17.015220000
H	15.511144000	15.355963000	17.043853000
C	18.107274000	14.311655000	17.063879000
H	17.294757000	13.572668000	17.231439000
H	18.984724000	13.963396000	17.642193000
H	18.361774000	14.273044000	15.984361000
C	20.510010000	16.263400000	17.547909000
H	20.461569000	15.357747000	16.907111000
C	20.638174000	15.836327000	19.009729000
H	21.549670000	15.219952000	19.156273000
H	19.772271000	15.242788000	19.363455000
H	20.719285000	16.731029000	19.655738000
C	21.710854000	17.103697000	17.115393000
H	21.817097000	17.996470000	17.760729000
H	21.641595000	17.439016000	16.062594000
H	22.641925000	16.507175000	17.216148000
P	19.364176000	20.771643000	18.456913000
C	19.158033000	21.784774000	20.017363000
H	19.750189000	22.714899000	19.879412000
C	19.686101000	20.992509000	21.214927000
H	19.598238000	21.593581000	22.143144000
H	20.748500000	20.693769000	21.110163000
H	19.080748000	20.072957000	21.332793000
C	17.676826000	22.135110000	20.214564000
H	17.127219000	21.233041000	20.554392000
H	17.203074000	22.536334000	19.294952000
H	17.558976000	22.900867000	21.007800000
C	21.225875000	20.655517000	18.152466000
H	21.501027000	19.733207000	18.708394000
C	21.463837000	20.424738000	16.652960000
H	21.317342000	21.363572000	16.081141000
H	20.775344000	19.665260000	16.230703000
H	22.502445000	20.079418000	16.472141000
C	22.066693000	21.824440000	18.672896000
H	23.140531000	21.634138000	18.460860000
H	21.972474000	21.970927000	19.766342000
H	21.804332000	22.778225000	18.171155000
H	15.069794000	17.289492000	22.212719000
H	19.312488000	18.518865000	19.272082000
H	16.017304000	20.254653000	18.914415000
H	15.605563000	18.672754000	18.475483000

BP222

N	16.362151000	18.295415000	19.452506000
N	15.390319000	17.586017000	18.589517000
Fe	16.014213000	18.650358000	21.213047000
P	15.636459000	19.796078000	23.174927000
C	15.784673000	18.575756000	24.552574000
C	15.235344000	18.746780000	25.838056000
H	14.638108000	19.637490000	26.077801000
C	15.452921000	17.772582000	26.823870000
H	15.014931000	17.901232000	27.825114000
C	16.237181000	16.640212000	26.536096000
H	16.418633000	15.882780000	27.313541000
C	16.793081000	16.472740000	25.258449000
H	17.419578000	15.591459000	25.054684000
C	16.548693000	17.429933000	24.252459000
P	17.302600000	17.356664000	22.568567000
C	19.123939000	17.487778000	23.132881000
H	19.231871000	16.557929000	23.734105000
C	19.402159000	18.666364000	24.061764000
H	18.850992000	18.573178000	25.016814000
H	20.484000000	18.688454000	24.309855000
H	19.150151000	19.641078000	23.609351000
C	20.077377000	17.403053000	21.948768000
H	20.056846000	16.394042000	21.501295000
H	19.818123000	18.132208000	21.152185000
H	21.121859000	17.599773000	22.268684000
C	17.267920000	15.517889000	22.120204000
H	18.139829000	15.090206000	22.659264000
C	17.485642000	15.363480000	20.615500000
H	18.269947000	16.033729000	20.221816000
H	17.747391000	14.316162000	20.363508000
H	16.545254000	15.611152000	20.086302000
C	16.005391000	14.759833000	22.536832000

H	15.784652000	14.816835000	23.618323000
H	15.117491000	15.126370000	21.985604000
H	16.124078000	13.687114000	22.276802000
C	16.492691000	21.290038000	23.841207000
C	16.141971000	22.551195000	23.311719000
H	15.354794000	22.627628000	22.547826000
C	16.769611000	23.716106000	23.776097000
H	16.487898000	24.692175000	23.352612000
C	17.748622000	23.635227000	24.782766000
H	18.243833000	24.548767000	25.145558000
C	18.070264000	22.387837000	25.343308000
H	18.810535000	22.319153000	26.154856000
C	17.439942000	21.220429000	24.880582000
H	17.677547000	20.256865000	25.346910000
C	13.896863000	20.378052000	23.349452000
C	13.426389000	21.198341000	24.396648000
H	14.125489000	21.629587000	25.127929000
C	12.056213000	21.478137000	24.494413000
H	11.685952000	22.117245000	25.310084000
C	11.156943000	20.939116000	23.555017000
H	10.080138000	21.149872000	23.638176000
C	11.627735000	20.141935000	22.501525000
H	10.908948000	19.738886000	21.774887000
C	13.009199000	19.876348000	22.386417000
P	13.776093000	18.898878000	21.022135000
C	13.151980000	19.844461000	19.500225000
H	13.857151000	19.559394000	18.693879000
C	11.732467000	19.509496000	19.037438000
H	10.970898000	19.778503000	19.794830000
H	11.602117000	18.440776000	18.778133000
H	11.494519000	20.094221000	18.125837000
C	13.349654000	21.346650000	19.747819000
H	13.169190000	21.916504000	18.814698000
H	14.377113000	21.578976000	20.091601000
H	12.642202000	21.731744000	20.507430000
C	12.709107000	17.344429000	21.188918000
H	11.672146000	17.701674000	21.019173000
C	12.817045000	16.810869000	22.624157000
H	12.218134000	15.882694000	22.725870000
H	12.445122000	17.538044000	23.370081000
H	13.863479000	16.564036000	22.892080000
C	13.018009000	16.254958000	20.160229000
H	14.052958000	15.866527000	20.271580000
H	12.849018000	16.580638000	19.111122000
H	12.347057000	15.384945000	20.311775000
Fe	17.919431000	18.809129000	18.664928000
P	17.220433000	19.629711000	16.705634000
C	18.087113000	18.817445000	15.294298000
C	18.013308000	19.262880000	13.959572000
H	17.414594000	20.145353000	13.693021000
C	18.710594000	18.572031000	12.957531000
H	18.660808000	18.922415000	11.915622000
C	19.470920000	17.434113000	13.284807000
H	20.015168000	16.890183000	12.498058000
C	19.539854000	16.986374000	14.613243000
H	20.136907000	16.092593000	14.846590000
C	18.851962000	17.683097000	15.628772000
C	17.626274000	21.417011000	16.487736000
C	17.121270000	22.244713000	15.464009000
H	16.391417000	21.861626000	14.735846000
C	17.533710000	23.583348000	15.391213000
H	17.141100000	24.232966000	14.594435000
C	18.435072000	24.097805000	16.342150000
H	18.748006000	25.151603000	16.289994000
C	18.926928000	23.276042000	17.368071000
H	19.612167000	23.701422000	18.115192000
C	18.524991000	21.926182000	17.441475000
C	15.456388000	19.632953000	16.137983000
C	14.596864000	20.643821000	16.629909000
H	14.980387000	21.391073000	17.340722000
C	13.273896000	20.733010000	16.172682000
H	12.628534000	21.550399000	16.527025000
C	12.775814000	19.790387000	15.256057000
H	11.735082000	19.859159000	14.905549000
C	13.616429000	18.773476000	14.774288000
H	13.240778000	18.042765000	14.042413000
C	14.954446000	18.705361000	15.195989000
H	15.618835000	17.949823000	14.755141000
P	18.812800000	17.168039000	17.408988000
C	17.738275000	15.580486000	17.273846000
H	17.257871000	15.532088000	18.271704000
C	16.650961000	15.728914000	16.202328000
H	17.075603000	15.638355000	15.183586000
H	16.130461000	16.702971000	16.232388000
H	15.883713000	14.936706000	16.323977000
C	18.485954000	14.259140000	17.067409000
H	17.750201000	13.427555000	17.058541000
H	19.218166000	14.037985000	17.865038000
H	19.012284000	14.234819000	16.091299000
C	20.558867000	16.469404000	17.589800000
H	20.566583000	15.624987000	16.870819000
C	20.848753000	15.928081000	18.986602000
H	21.814592000	15.381904000	18.984530000
H	20.076461000	15.233081000	19.365639000
H	20.940915000	16.765296000	19.700819000
C	21.653714000	17.454364000	17.181060000
H	21.741831000	18.274044000	17.917714000
H	21.495275000	17.895516000	16.180131000
H	22.629078000	16.924938000	17.162528000
P	19.055455000	20.732433000	18.754855000
C	18.617703000	21.735263000	20.269403000
H	19.190825000	22.681305000	20.166971000
C	19.020658000	21.020129000	21.553139000
H	18.826683000	21.661820000	22.434951000
H	20.089644000	20.731514000	21.568051000
H	18.413816000	20.096593000	21.664322000
C	17.121754000	22.048093000	20.224380000
H	16.526982000	21.120401000	20.392590000
H	16.805555000	22.479924000	19.255157000
H	16.860958000	22.771436000	21.016314000
C	20.930840000	20.759659000	18.572934000
H	21.232717000	19.794534000	19.031922000
C	21.270879000	20.730454000	17.074898000
H	21.128354000	21.729390000	16.616715000
H	20.636322000	20.017072000	16.511243000
H	22.329983000	20.438714000	16.923528000
C	21.665744000	21.891933000	19.296895000
H	22.758106000	21.780820000	19.130672000
H	21.500865000	21.883485000	20.391348000
H	21.384066000	22.891906000	18.910848000
H	15.384468000	17.361040000	21.578521000
H	19.251287000	18.523938000	19.357490000
H	15.158722000	18.154821000	17.741398000
H	14.512950000	17.308020000	19.072660000
H	15.817756000	16.705072000	18.245877000

CP2

N	16.691319000	18.555225000	20.544313000
N	17.414708000	19.183626000	19.831723000
Fe	15.585166000	17.722197000	21.607239000
P	15.214862000	19.009301000	23.269065000
C	15.909490000	18.358519000	24.868562000
C	15.949942000	19.045355000	26.098183000
H	15.574144000	20.078566000	26.167712000
C	16.500736000	18.420416000	27.233340000
H	16.530744000	18.954571000	28.196483000
C	17.018120000	17.116176000	27.133489000
H	17.450131000	16.625746000	28.020636000
C	17.001966000	16.442536000	25.896136000
H	17.428213000	15.428159000	25.829744000
C	16.454884000	17.062828000	24.755544000
P	16.408263000	16.338554000	23.025775000

C	18.254720000	15.862873000	22.913198000
H	18.523725000	15.363843000	23.870789000
C	19.055428000	17.164623000	22.780466000
H	18.854774000	17.851091000	23.628999000
H	20.147570000	16.956951000	22.764482000
H	18.783112000	17.702036000	21.848715000
C	18.544455000	14.904915000	21.755764000
H	18.157783000	13.884566000	21.945779000
H	18.087453000	15.275911000	20.816081000
H	19.639152000	14.809757000	21.588506000
C	15.564109000	14.673056000	23.428898000
H	16.238621000	14.104298000	24.108329000
C	15.314058000	13.862163000	22.152979000
H	16.245999000	13.456332000	21.715534000
H	14.632145000	13.006117000	22.350021000
H	14.853729000	14.515751000	21.382154000
C	14.246950000	14.990294000	24.151595000
H	14.417475000	15.348197000	25.186167000
H	13.704993000	15.794516000	23.608495000
H	13.588554000	14.096308000	24.202743000
C	15.829511000	20.762245000	23.386959000
C	15.035408000	21.896583000	23.654186000
H	13.960131000	21.779117000	23.855927000
C	15.605826000	23.182910000	23.664373000
H	14.971800000	24.059477000	23.874730000
C	16.978795000	23.350396000	23.415971000
H	17.426688000	24.356382000	23.429217000
C	17.776157000	22.223453000	23.137878000
H	18.848097000	22.347540000	22.925551000
C	17.204074000	20.944241000	23.108954000
H	17.816547000	20.064423000	22.852141000
C	13.382842000	19.176017000	23.596162000
C	12.780577000	19.602845000	24.795239000
H	13.397325000	19.957263000	25.634937000
C	11.378274000	19.552209000	24.941596000
H	10.914074000	19.853124000	25.894516000
C	10.579977000	19.112340000	23.874666000
H	9.484795000	19.066245000	23.986563000
C	11.178478000	18.724034000	22.657151000
H	10.536846000	18.389417000	21.827370000
C	12.580483000	18.730710000	22.514557000
P	13.553779000	18.125152000	21.028747000
C	13.325472000	19.608902000	19.838985000
H	13.847875000	19.270076000	18.917418000
C	11.879734000	19.981098000	19.499673000
H	11.304000000	20.241558000	20.412966000
H	11.335454000	19.172614000	18.971326000
H	11.855000000	20.870415000	18.835212000
C	14.093308000	20.815481000	20.381453000
H	14.066392000	21.652626000	19.652680000
H	15.152222000	20.561335000	20.573613000
H	13.658439000	21.184585000	21.333850000
C	12.345122000	16.897702000	20.247053000
H	11.388517000	17.441358000	20.090788000
C	12.086404000	15.701942000	21.165187000
H	11.318260000	15.029056000	20.724424000
H	11.726807000	16.012285000	22.166726000
H	13.010107000	15.110956000	21.312830000
C	12.893955000	16.454202000	18.887396000
H	13.903899000	16.011982000	19.017808000
H	12.994953000	17.298025000	18.175068000
H	12.233585000	15.692293000	18.418561000
Fe	18.691048000	19.808131000	18.590320000
P	17.656381000	20.110588000	16.732542000
C	18.281295000	18.905245000	15.438898000
C	18.033490000	18.990115000	14.055838000
H	17.379928000	19.780495000	13.656923000
C	18.627050000	18.070273000	13.170724000
H	18.445239000	18.154001000	12.087402000
C	19.455855000	17.050988000	13.670442000
H	19.925659000	16.333006000	12.979460000
C	19.685449000	16.945141000	15.056031000
H	20.330469000	16.136926000	15.434827000
C	19.108972000	17.871804000	15.947192000
C	18.040519000	21.790355000	15.998905000
C	17.411284000	22.353171000	14.870548000
H	16.639221000	21.786788000	14.326477000
C	17.733537000	23.659671000	14.459906000
H	17.233276000	24.099481000	13.582465000
C	18.677585000	24.409276000	15.183973000
H	18.917624000	25.439626000	14.876678000
C	19.311214000	23.847093000	16.308615000
H	20.037463000	24.451947000	16.873225000
C	19.003590000	22.533287000	16.717539000
C	15.822858000	20.101498000	16.413614000
C	15.073820000	21.192548000	16.911955000
H	15.601255000	22.020474000	17.412807000
C	13.680813000	21.232061000	16.767436000
H	13.117258000	22.102415000	17.138521000
C	13.000928000	20.158550000	16.160220000
H	11.904869000	20.181602000	16.060553000
C	13.731907000	19.054133000	15.691299000
H	13.209894000	18.206999000	15.218380000
C	15.133201000	19.031151000	15.807143000
H	15.697724000	18.174318000	15.413062000
P	19.309055000	17.894474000	17.802292000
C	18.129046000	16.483193000	18.313272000
H	18.064823000	16.627544000	19.413329000
C	16.726250000	16.734498000	17.760464000
H	16.679325000	16.535854000	16.668884000
H	16.389710000	17.769479000	17.949150000
H	15.998406000	16.070885000	18.269666000
C	18.588092000	15.056415000	18.002128000
H	17.808300000	14.327273000	18.313816000
H	19.521896000	14.770888000	18.525511000
H	18.749000000	14.907625000	16.912720000
C	20.983610000	17.060897000	18.073749000
H	20.929618000	16.062469000	17.589586000
C	21.187205000	16.879646000	19.581169000
H	22.182579000	16.436178000	19.801015000
H	20.417852000	16.225289000	20.034248000
H	21.113697000	17.865400000	20.086991000
C	22.135966000	17.844900000	17.448104000
H	22.244112000	18.834614000	17.933173000
H	21.990439000	18.013729000	16.363420000
H	23.094815000	17.297872000	17.580543000
P	19.777411000	21.606626000	18.144141000
C	19.876027000	22.959796000	19.462922000
H	20.443804000	23.807776000	19.023466000
C	20.628723000	22.425734000	20.685517000
H	20.708422000	23.201606000	21.477702000
H	21.658869000	22.096374000	20.435931000
H	20.098166000	21.542273000	21.097000000
C	18.465501000	23.435397000	19.817608000
H	17.848991000	22.583929000	20.177607000
H	17.949585000	23.871478000	18.937505000
H	18.492781000	24.207838000	20.615543000
C	21.576016000	21.496787000	17.518886000
H	22.002588000	20.734363000	18.206141000
C	21.561868000	20.918724000	16.098353000
H	21.243323000	21.686919000	15.363109000
H	20.861813000	20.063889000	16.012539000
H	22.573369000	20.568300000	15.801759000
C	22.440555000	22.759320000	17.590010000
H	23.467724000	22.536928000	17.224991000
H	22.543101000	23.158036000	18.618558000
H	22.040986000	23.569811000	16.946642000
H	15.476959000	16.446679000	20.742420000
H	19.872625000	19.676099000	19.676012000
H	17.415959000	20.393110000	19.836529000

CP12

N	16.290121000	18.915925000	20.262682000
N	17.623658000	18.755745000	20.004494000
Fe	15.392601000	18.474157000	21.813647000
P	14.694729000	19.398271000	23.578640000
C	15.434820000	18.658013000	25.111393000
C	15.137663000	19.005895000	26.444000000
H	14.446638000	19.832952000	26.665742000
C	15.744412000	18.310845000	27.503921000
H	15.502301000	18.576788000	28.544507000
C	16.663537000	17.280645000	27.233508000
H	17.132393000	16.728264000	28.062642000
C	17.005698000	16.972672000	25.905510000
H	17.756094000	16.190657000	25.707515000
C	16.400842000	17.664045000	24.836506000
P	16.902912000	17.466525000	23.046376000
C	18.753266000	17.900127000	23.334643000
H	18.979579000	17.429981000	24.315566000
C	18.863026000	19.418458000	23.494382000
H	18.178206000	19.797326000	24.280520000
H	19.896001000	19.713993000	23.775219000
H	18.608913000	19.945402000	22.553631000
C	19.750396000	17.325492000	22.321530000
H	19.732375000	16.217887000	22.304855000
H	19.606217000	17.679432000	21.276823000
H	20.781630000	17.623537000	22.610075000
C	17.005243000	15.596394000	22.796126000
H	17.922751000	15.250753000	23.321723000
C	17.140641000	15.339872000	21.287807000
H	17.996585000	15.879100000	20.839072000
H	17.267576000	14.256481000	21.078086000
H	16.232327000	15.699799000	20.763032000
C	15.791227000	14.864170000	23.373610000
H	15.712306000	14.974233000	24.473295000
H	14.854617000	15.254269000	22.925199000
H	15.852086000	13.778304000	23.145538000
C	14.981820000	21.213847000	23.827651000
C	14.196442000	22.045654000	24.650870000
H	13.335481000	21.635830000	25.199736000
C	14.493105000	23.416522000	24.756651000
H	13.871824000	24.061195000	25.398005000
C	15.574187000	23.963597000	24.043394000
H	15.802125000	25.037732000	24.125640000
C	16.356882000	23.137200000	23.215781000
H	17.196035000	23.563549000	22.644372000
C	16.057845000	21.771083000	23.105437000
H	16.630969000	21.110939000	22.433348000
C	12.852701000	19.193537000	23.754231000
C	12.082373000	19.285622000	24.929293000
H	12.550330000	19.535559000	25.891839000
C	10.699571000	19.028831000	24.890553000
H	10.106374000	19.089908000	25.816108000
C	10.081544000	18.684141000	23.677488000
H	9.001172000	18.473927000	23.647451000
C	10.844731000	18.594238000	22.498889000
H	10.348698000	18.308815000	21.559634000
C	12.230842000	18.841052000	22.528560000
P	13.374944000	18.712426000	21.065026000
C	12.927838000	20.228741000	20.038111000
H	13.484377000	20.039256000	19.097143000
C	11.446497000	20.390973000	19.692379000
H	10.835358000	20.589273000	20.596992000
H	11.024854000	19.501569000	19.181563000
H	11.304491000	21.254802000	19.007653000
C	13.521244000	21.481037000	20.685042000
H	13.357206000	22.371691000	20.041451000
H	14.608971000	21.362168000	20.851183000
H	13.058923000	21.694492000	21.671956000
C	12.634363000	17.322143000	20.037526000
H	11.542979000	17.516669000	19.959197000
C	12.845710000	15.970384000	20.723475000
H	12.323876000	15.166511000	20.161617000
H	12.457851000	15.968104000	21.762484000
H	13.923857000	15.717668000	20.768736000
C	13.250654000	17.367526000	18.633813000
H	14.357052000	17.406444000	18.689590000
H	12.914101000	18.248201000	18.054646000
H	12.972086000	16.465342000	18.049136000
Fe	18.696648000	18.884003000	18.564738000
P	17.584619000	19.610675000	16.848039000
C	18.113055000	18.662191000	15.319164000
C	17.819395000	19.047401000	13.997632000
H	17.178427000	19.920548000	13.803776000
C	18.351045000	18.323898000	12.914792000
H	18.136946000	18.643897000	11.883044000
C	19.155846000	17.195793000	13.151241000
H	19.571467000	16.626894000	12.304802000
C	19.429204000	16.789040000	14.470169000
H	20.046264000	15.894192000	14.641115000
C	18.925829000	17.527975000	15.559507000
C	18.110529000	21.356919000	16.419839000
C	17.470791000	22.200220000	15.489473000
H	16.594116000	21.842503000	14.928462000
C	17.926746000	23.515985000	15.299586000
H	17.420370000	24.173237000	14.575349000
C	19.014169000	23.997858000	16.050506000
H	19.357273000	25.036477000	15.921891000
C	19.662193000	23.154516000	16.970397000
H	20.504144000	23.548072000	17.559303000
C	19.225318000	21.825678000	17.147740000
C	15.756990000	19.804690000	16.531237000
C	15.086774000	20.884905000	17.150165000
H	15.649525000	21.577261000	17.797629000
C	13.720024000	21.105109000	16.922673000
H	13.222601000	21.972327000	17.383288000
C	12.985579000	20.222030000	16.109653000
H	11.911727000	20.391531000	15.937280000
C	13.633072000	19.120128000	15.525520000
H	13.066039000	18.416597000	14.895905000
C	15.009188000	18.917350000	15.728459000
H	15.509161000	18.071163000	15.233676000
P	19.228319000	17.182906000	17.360664000
C	18.017910000	15.729504000	17.667181000
H	18.363176000	15.346463000	18.651808000
C	16.590021000	16.248962000	17.835464000
H	16.202594000	16.673223000	16.886646000
H	16.541105000	17.039920000	18.604674000
H	15.906335000	15.425228000	18.130692000
C	18.072112000	14.592803000	16.641061000
H	17.401075000	13.765296000	16.958406000
H	19.083240000	14.155550000	16.518258000
H	17.723660000	14.931781000	15.643200000
C	20.853412000	16.217338000	17.425694000
H	20.652612000	15.267904000	16.885550000
C	21.174980000	15.888912000	18.889661000
H	22.093330000	15.267270000	18.956476000
H	20.356562000	15.331753000	19.388448000
H	21.335308000	16.822262000	19.467496000
C	22.035840000	16.910629000	16.747178000
H	22.340694000	17.816287000	17.305645000
H	21.817081000	17.208230000	15.703930000
H	22.913033000	16.228928000	16.723886000
P	20.018357000	20.551944000	18.252946000
C	20.314040000	21.549419000	19.826821000
H	20.893595000	22.449157000	19.526313000
C	21.134746000	20.727258000	20.824171000
H	21.326787000	21.308297000	21.751232000
H	22.116977000	20.418911000	20.411124000
H	20.587611000	19.803261000	21.094934000
C	18.971781000	21.992044000	20.418912000
H	18.331793000	21.109095000	20.619380000
H	18.412097000	22.652424000	19.725487000
H	19.131298000	22.546477000	21.368049000

C	21.744134000	20.439209000	17.472448000
H	22.146622000	19.523704000	17.957111000
C	21.583784000	20.173781000	15.970356000
H	21.263731000	21.095437000	15.441929000
H	20.823041000	19.395736000	15.765448000
H	22.543932000	19.844132000	15.520595000
C	22.722422000	21.590571000	17.727306000
H	23.708200000	21.351150000	17.271776000
H	22.901295000	21.780542000	18.803872000
H	22.376425000	22.536333000	17.263009000
H	14.694910000	17.269504000	22.414458000
H	19.829213000	18.434137000	19.490414000
H	15.865568000	19.270350000	19.389383000
H	18.020920000	18.437367000	20.899874000

BP22(H)

N	16.839363000	18.912099000	20.492999000
N	17.009464000	19.350869000	19.305276000
Fe	16.453784000	18.293136000	21.984013000
P	16.305534000	19.038824000	24.000285000
C	17.564998000	18.289044000	25.120687000
C	17.754891000	18.624519000	26.475513000
H	17.130152000	19.393145000	26.955310000
C	18.770180000	17.992613000	27.211140000
H	18.917458000	18.248786000	28.271269000
C	19.605871000	17.043288000	26.592959000
H	20.401871000	16.551631000	27.172568000
C	19.442017000	16.735013000	25.232715000
H	20.115891000	16.007603000	24.753916000
C	18.421872000	17.361171000	24.489261000
P	18.178320000	17.123039000	22.669484000
C	19.880749000	17.599206000	22.024401000
H	20.594982000	17.006947000	22.638328000
C	20.116557000	19.091245000	22.277359000
H	19.967835000	19.368220000	23.340460000
H	21.156319000	19.364938000	22.001924000
H	19.429626000	19.705103000	21.658878000
C	20.073279000	17.240658000	20.547459000
H	20.064674000	16.147676000	20.372313000
H	19.291315000	17.705287000	19.912649000
H	21.055866000	17.620648000	20.196683000
C	18.074261000	15.258237000	22.459055000
H	19.116069000	14.885636000	22.577272000
C	17.558252000	14.899379000	21.058412000
H	18.154144000	15.354758000	20.245840000
H	17.578550000	13.798624000	20.918359000
H	16.511513000	15.240203000	20.926994000
C	17.185327000	14.643012000	23.547045000
H	17.612430000	14.756319000	24.561716000
H	16.179168000	15.112739000	23.549458000
H	17.047555000	13.558547000	23.355600000
C	16.524225000	20.846572000	24.285427000
C	15.771607000	21.576410000	25.227383000
H	14.999756000	21.079132000	25.832772000
C	15.992608000	22.955753000	25.385075000
H	15.398368000	23.520872000	26.119498000
C	16.960299000	23.611670000	24.605520000
H	17.127683000	24.692645000	24.728863000
C	17.709522000	22.885480000	23.661811000
H	18.463944000	23.395255000	23.043004000
C	17.489302000	21.509960000	23.499098000
H	18.053464000	20.941468000	22.744783000
C	14.633077000	18.668282000	24.694373000
C	14.281540000	18.594218000	26.054538000
H	15.038911000	18.728945000	26.840007000
C	12.949609000	18.327260000	26.417080000
H	12.678037000	18.259133000	27.481364000
C	11.969825000	18.142076000	25.426618000
H	10.928593000	17.931365000	25.713921000
C	12.317381000	18.205530000	24.065954000
H	11.546211000	18.030096000	23.301676000
C	13.651623000	18.459056000	23.693843000
P	14.277118000	18.466431000	21.955140000
C	13.588842000	20.028731000	21.167363000
H	13.882885000	19.908093000	20.100898000
C	12.065990000	20.165242000	21.259162000
H	11.738573000	20.336511000	22.304988000
H	11.526735000	19.279784000	20.867716000
H	11.732464000	21.040369000	20.662949000
C	14.311253000	21.257475000	21.727268000
H	13.956366000	22.175369000	21.214293000
H	15.406658000	21.183709000	21.587813000
H	14.118572000	21.384367000	22.812303000
C	13.357528000	17.078073000	21.099372000
H	12.284704000	17.366577000	21.127389000
C	13.531374000	15.745147000	21.832497000
H	12.903605000	14.965698000	21.353067000
H	13.238689000	15.806124000	22.899357000
H	14.584721000	15.403348000	21.790266000
C	13.814772000	16.993371000	19.637152000
H	14.900147000	16.770617000	19.575581000
H	13.627490000	17.930780000	19.075653000
H	13.268776000	16.180979000	19.114466000
H	15.894643000	16.972957000	22.451742000
H	17.312855000	20.323956000	19.141330000
H	16.893425000	18.733736000	18.486046000

BP222(H)

N	17.023869000	19.468725000	20.632658000
N	16.732091000	20.370294000	19.548804000
Fe	16.670216000	18.475854000	21.872729000
P	16.447212000	19.151155000	23.954946000
C	17.748281000	18.506200000	25.070195000
C	18.012247000	18.986851000	26.366359000
H	17.438621000	19.829775000	26.780843000
C	19.034231000	18.386158000	27.118610000
H	19.251190000	18.751918000	28.133269000
C	19.788165000	17.326493000	26.578241000
H	20.591345000	16.867289000	27.173739000
C	19.535738000	16.859550000	25.277592000
H	20.144307000	16.042653000	24.861183000
C	18.514837000	17.461779000	24.517927000
P	18.074659000	16.992019000	22.794160000
C	19.682956000	17.151569000	21.837514000
H	20.309551000	16.335605000	22.262920000
C	20.334124000	18.510330000	22.126867000
H	20.477191000	18.700937000	23.208144000
H	21.331664000	18.547249000	21.643937000
H	19.727711000	19.336718000	21.701832000
C	19.504128000	16.932266000	20.330802000
H	19.033784000	15.963377000	20.082756000
H	18.885393000	17.739637000	19.889426000
H	20.498190000	16.958837000	19.839615000
C	17.648136000	15.189728000	23.004858000
H	18.527747000	14.769620000	23.540092000
C	17.475002000	14.439523000	21.681566000
H	18.437956000	14.298931000	21.154775000
H	17.053631000	13.432844000	21.879309000
H	16.780401000	14.957683000	20.989148000
C	16.425256000	15.106062000	23.931018000
H	16.634705000	15.532531000	24.931787000
H	15.551300000	15.643055000	23.512668000
H	16.132522000	14.046034000	24.073308000
C	16.515461000	20.954853000	24.245470000
C	15.589277000	21.618185000	25.074300000
H	14.765048000	21.065229000	25.548082000
C	15.713569000	23.002139000	25.284055000
H	14.988617000	23.520719000	25.929348000
C	16.752797000	23.719844000	24.669015000
H	16.843215000	24.804386000	24.832534000
C	17.677407000	23.054880000	23.842186000
H	18.491613000	23.616166000	23.359467000

C	17.561299000	21.674339000	23.628141000
H	18.277715000	21.155616000	22.972078000
C	14.833311000	18.554784000	24.591617000
C	14.504871000	18.414431000	25.951073000
H	15.236169000	18.660987000	26.734834000
C	13.232539000	17.934593000	26.306037000
H	12.974828000	17.811679000	27.368431000
C	12.295040000	17.604664000	25.311922000
H	11.302009000	17.226687000	25.596985000
C	12.620729000	17.740308000	23.951172000
H	11.882093000	17.465113000	23.184763000
C	13.897731000	18.206793000	23.586803000
P	14.485521000	18.397386000	21.856712000
C	13.683486000	19.976061000	21.208051000
H	13.922836000	19.937861000	20.122088000
C	12.159085000	19.979552000	21.372635000
H	11.869730000	19.997294000	22.442680000
H	11.662597000	19.118624000	20.886082000
H	11.748268000	20.897371000	20.903744000
C	14.298423000	21.236558000	21.818012000
H	13.929789000	22.133611000	21.280497000
H	15.405583000	21.242841000	21.792781000
H	14.008490000	21.348971000	22.881197000
C	13.667076000	17.076424000	20.818328000
H	12.591717000	17.354880000	20.822163000
C	13.803034000	15.665555000	21.391174000
H	13.213089000	14.962021000	20.769221000
H	13.431063000	15.583413000	22.430428000
H	14.852651000	15.316569000	21.371205000
C	14.200242000	17.169319000	19.382095000
H	15.286259000	16.944151000	19.342188000
H	14.032471000	18.166437000	18.927895000
H	13.679179000	16.429271000	18.741664000
H	16.254613000	17.057897000	21.618310000
H	17.611922000	20.773393000	19.164259000
H	16.247878000	19.890006000	18.761506000
H	16.138412000	21.170006000	19.859528000

CP222(H)

N	17.043068000	18.885247000	20.469212000
N	16.497427000	19.614316000	19.343722000
Fe	16.515628000	18.258601000	21.971959000
P	16.329244000	19.025140000	23.978305000
C	17.575840000	18.294451000	25.131416000
C	17.739734000	18.653115000	26.484011000
H	17.100688000	19.424910000	26.939680000
C	18.746015000	18.042865000	27.249844000
H	18.871291000	18.318337000	28.308057000
C	19.600827000	17.090840000	26.663156000
H	20.391193000	16.616648000	27.264800000
C	19.461939000	16.757225000	25.306051000
H	20.150345000	16.026948000	24.852710000
C	18.451038000	17.360863000	24.531379000
P	18.242382000	17.075414000	22.712138000
C	19.956226000	17.534505000	22.078632000
H	20.669245000	16.936196000	22.688890000
C	20.196744000	19.027402000	22.329039000
H	20.041923000	19.310234000	23.389934000
H	21.239610000	19.296142000	22.059118000
H	19.512722000	19.635495000	21.702244000
C	20.129496000	17.182091000	20.597160000
H	20.126829000	16.089112000	20.419741000
H	19.322367000	17.645437000	19.991340000
H	21.102469000	17.571651000	20.229342000
C	18.139944000	15.202566000	22.562074000
H	19.177033000	14.825662000	22.705481000
C	17.637085000	14.806813000	21.166804000
H	18.251114000	15.226653000	20.348464000
H	17.638185000	13.701804000	21.060863000
H	16.598595000	15.164878000	21.015231000
C	17.228702000	14.623437000	23.651774000
H	17.629644000	14.779334000	24.671605000
H	16.220066000	15.086435000	23.610267000
H	17.102804000	13.530889000	23.500554000
C	16.541971000	20.834725000	24.263314000
C	15.778159000	21.568677000	25.192942000
H	15.001051000	21.072486000	25.792787000
C	15.994624000	22.949706000	25.345367000
H	15.391194000	23.518046000	26.069867000
C	16.970416000	23.602807000	24.573632000
H	17.135089000	24.684669000	24.693219000
C	17.731546000	22.872320000	23.642330000
H	18.492294000	23.380278000	23.029607000
C	17.514589000	21.495753000	23.484016000
H	18.083979000	20.922219000	22.736529000
C	14.648344000	18.649434000	24.647722000
C	14.276644000	18.561438000	26.001758000
H	15.018807000	18.710036000	26.799297000
C	12.945882000	18.260226000	26.341957000
H	12.658717000	18.179445000	27.401350000
C	11.987699000	18.056658000	25.334331000
H	10.947446000	17.817881000	25.602999000
C	12.355754000	18.139243000	23.979558000
H	11.599565000	17.953125000	23.203009000
C	13.689087000	18.425244000	23.629046000
P	14.348986000	18.462482000	21.896766000
C	13.611863000	20.038183000	21.151834000
H	13.779646000	19.896487000	20.059850000
C	12.107050000	20.219872000	21.374046000
H	11.878255000	20.418094000	22.440771000
H	11.516155000	19.339637000	21.050122000
H	11.741998000	21.090975000	20.790052000
C	14.408152000	21.258435000	21.624369000
H	14.082832000	22.172828000	21.086617000
H	15.501418000	21.131558000	21.483567000
H	14.259341000	21.435833000	22.709010000
C	13.408853000	17.110776000	20.995718000
H	12.340670000	17.417081000	21.006762000
C	13.547347000	15.756757000	21.696650000
H	12.936006000	14.993283000	21.171966000
H	13.213811000	15.791010000	22.752489000
H	14.601257000	15.412961000	21.688725000
C	13.896751000	17.047773000	19.541546000
H	14.984418000	16.824679000	19.502057000
H	13.706433000	17.990718000	18.988182000
H	13.367196000	16.242380000	18.991617000
H	15.886596000	16.936854000	22.381788000
H	17.250487000	20.031536000	18.761012000
H	15.943763000	19.002538000	18.709869000
H	15.877509000	20.402314000	19.631338000

^{TS}DP222(H)

N	17.298177000	18.691837000	20.511183000
N	16.397990000	19.509137000	19.358420000
Fe	16.511506000	18.224401000	21.992524000
P	16.316055000	19.009951000	23.952427000
C	17.542737000	18.284081000	25.143286000
C	17.670998000	18.596460000	26.511284000
H	17.018029000	19.351457000	26.976240000
C	18.658327000	17.963505000	27.288074000
H	18.748150000	18.198886000	28.360108000
C	19.537021000	17.041284000	26.692116000
H	20.312997000	16.548262000	27.298404000
C	19.437896000	16.759957000	25.317114000
H	20.146551000	16.053235000	24.856617000
C	18.441944000	17.377575000	24.534690000
P	18.256219000	17.139062000	22.691246000
C	20.022082000	17.565242000	22.154715000
H	20.714686000	16.988284000	22.807930000
C	20.234210000	19.066272000	22.383104000
H	20.017433000	19.367497000	23.428602000
H	21.284422000	19.353823000	22.159211000

H	19.561434000	19.642141000	21.715508000
C	20.272090000	17.191943000	20.687257000
H	20.265162000	16.095910000	20.523691000
H	19.490386000	17.656409000	20.049585000
H	21.267575000	17.564692000	20.359407000
C	18.204770000	15.253053000	22.522918000
H	19.249238000	14.893534000	22.661525000
C	17.711352000	14.881775000	21.117916000
H	18.320758000	15.337156000	20.314913000
H	17.723296000	13.779223000	20.977450000
H	16.673427000	15.247009000	20.977831000
C	17.303158000	14.620907000	23.589595000
H	17.695163000	14.759379000	24.615948000
H	16.287820000	15.069606000	23.549881000
H	17.199571000	13.528981000	23.411175000
C	16.580832000	20.827530000	24.243942000
C	15.961916000	21.572657000	25.267753000
H	15.259233000	21.087813000	25.961891000
C	16.222065000	22.949485000	25.396608000
H	15.728957000	23.526175000	26.195109000
C	17.103792000	23.588987000	24.507934000
H	17.304571000	24.666974000	24.610024000
C	17.721309000	22.848817000	23.481809000
H	18.405466000	23.347546000	22.776957000
C	17.453794000	21.477938000	23.347593000
H	17.890646000	20.880621000	22.529264000
C	14.612337000	18.710545000	24.630986000
C	14.215116000	18.629740000	25.979809000
H	14.938651000	18.811167000	26.787980000
C	12.889034000	18.287357000	26.306938000
H	12.589935000	18.205045000	27.363614000
C	11.953502000	18.046793000	25.287963000
H	10.917342000	17.773826000	25.541718000
C	12.342216000	18.138688000	23.936615000
H	11.601395000	17.929679000	23.150723000
C	13.671902000	18.453488000	23.598700000
P	14.361625000	18.479535000	21.858741000
C	13.541761000	20.055176000	21.152102000
H	13.726216000	19.966763000	20.057629000
C	12.031766000	20.198746000	21.363138000
H	11.785933000	20.358134000	22.432963000
H	11.464061000	19.313647000	21.010001000
H	11.645961000	21.077838000	20.802486000
C	14.303182000	21.275396000	21.678247000
H	13.958290000	22.209361000	21.185825000
H	15.397929000	21.177394000	21.529374000
H	14.155915000	21.395949000	22.771943000
C	13.369222000	17.131233000	20.985424000
H	12.297459000	17.419956000	21.041771000
C	13.557994000	15.769672000	21.660268000
H	12.899882000	15.009000000	21.188142000
H	13.320981000	15.800882000	22.742521000
H	14.608320000	15.430932000	21.561800000
C	13.797071000	17.079563000	19.512784000
H	14.891996000	16.897262000	19.441352000
H	13.552038000	18.012743000	18.963119000
H	13.283441000	16.251169000	18.980056000
H	15.874209000	16.957060000	22.592201000
H	16.991645000	19.768629000	18.555228000
H	15.624296000	18.920422000	19.012909000
H	15.999267000	20.371857000	19.760505000

EP1’(H)

N	17.072566000	18.889552000	20.497777000
Fe	16.504697000	18.267158000	21.979443000
P	16.315162000	19.029738000	23.962044000
C	17.537919000	18.276296000	25.135825000
C	17.684678000	18.585238000	26.502590000
H	17.041461000	19.341228000	26.978680000
C	18.679612000	17.947268000	27.262427000
H	18.788228000	18.183508000	28.332224000
C	19.542921000	17.017385000	26.654025000
H	20.323371000	16.518841000	27.249493000
C	19.423672000	16.737210000	25.282153000
H	20.119244000	16.024108000	24.812013000
C	18.423242000	17.366819000	24.514894000
P	18.225791000	17.148445000	22.672198000
C	19.978790000	17.603014000	22.129781000
H	20.659142000	17.035865000	22.803064000
C	20.184081000	19.104737000	22.350374000
H	19.970404000	19.407442000	23.395728000
H	21.232172000	19.392957000	22.121410000
H	19.510248000	19.682323000	21.685566000
C	20.269856000	17.201590000	20.679922000
H	20.273693000	16.103270000	20.536887000
H	19.519026000	17.639282000	19.990478000
H	21.269976000	17.576638000	20.373587000
C	18.179126000	15.270944000	22.469665000
H	19.226643000	14.919367000	22.603034000
C	17.689082000	14.910808000	21.060363000
H	18.287159000	15.386712000	20.260743000
H	17.725364000	13.811078000	20.907989000
H	16.641949000	15.249355000	20.922908000
C	17.285738000	14.622692000	23.533695000
H	17.688338000	14.739641000	24.558307000
H	16.270428000	15.072365000	23.514333000
H	17.177490000	13.535570000	23.333707000
C	16.576055000	20.839381000	24.268766000
C	15.968331000	21.559475000	25.316926000
H	15.268074000	21.062228000	26.004273000
C	16.237213000	22.930349000	25.479097000
H	15.754230000	23.487653000	26.296981000
C	17.113055000	23.588720000	24.598453000
H	17.319797000	24.662645000	24.726600000
C	17.715438000	22.874135000	23.546560000
H	18.392456000	23.387640000	22.845941000
C	17.442150000	21.508076000	23.379544000
H	17.870907000	20.936136000	22.540592000
C	14.614949000	18.709482000	24.637203000
C	14.230451000	18.650251000	25.990434000
H	14.969150000	18.804194000	26.790225000
C	12.895533000	18.368647000	26.333252000
H	12.604489000	18.312012000	27.393544000
C	11.939517000	18.154373000	25.326393000
H	10.895153000	17.931857000	25.594316000
C	12.318344000	18.205144000	23.972335000
H	11.566306000	18.007813000	23.193975000
C	13.655220000	18.470993000	23.618576000
P	14.312368000	18.454829000	21.880214000
C	13.604136000	20.016425000	21.092744000
H	13.904327000	19.894609000	20.028123000
C	12.083117000	20.166958000	21.180083000
H	11.755047000	20.342493000	22.225570000
H	11.537526000	19.282163000	20.794426000
H	11.750886000	21.042281000	20.581353000
C	14.340358000	21.236075000	21.654462000
H	13.995563000	22.164769000	21.151935000
H	15.432665000	21.138474000	21.506258000
H	14.158424000	21.356470000	22.743073000
C	13.344192000	17.075622000	21.048363000
H	12.268880000	17.349878000	21.116044000
C	13.557840000	15.735496000	21.758723000
H	12.897894000	14.958773000	21.317506000
H	13.335190000	15.793681000	22.843192000
H	14.607323000	15.397059000	21.649533000
C	13.757595000	17.005743000	19.572343000
H	14.857679000	16.878565000	19.486050000
H	13.480552000	17.920131000	19.009588000
H	13.265186000	16.145694000	19.071358000
H	15.943709000	16.936054000	22.495315000
H	17.892397000	19.013101000	19.877129000

FP111'(H)

N	16.848972000	18.136490000	20.041417000
Fe	16.507363000	18.258475000	22.047537000
P	16.305611000	18.913304000	24.057600000
C	17.582093000	18.209825000	25.183521000
C	17.701285000	18.480497000	26.561058000
H	16.985186000	19.147106000	27.065197000
C	18.761762000	17.919124000	27.289819000
H	18.855123000	18.124060000	28.367163000
C	19.711561000	17.107151000	26.641596000
H	20.543752000	16.670327000	27.214486000
C	19.613441000	16.866135000	25.261381000
H	20.374147000	16.243531000	24.764880000
C	18.550049000	17.421624000	24.521998000
P	18.345830000	17.228005000	22.683264000
C	20.094509000	17.666848000	22.111160000
H	20.781075000	17.067400000	22.748061000
C	20.343124000	19.155481000	22.373821000
H	20.209977000	19.417703000	23.442753000
H	21.380195000	19.432492000	22.090323000
H	19.655327000	19.788563000	21.773050000
C	20.352759000	17.300459000	20.645769000
H	20.272307000	16.212257000	20.457627000
H	19.661070000	17.826228000	19.954848000
H	21.381495000	17.604630000	20.358642000
C	18.280367000	15.346543000	22.477988000
H	19.330339000	14.995746000	22.588741000
C	17.759935000	14.969554000	21.083966000
H	18.331389000	15.443741000	20.261210000
H	17.827858000	13.872053000	20.932948000
H	16.693105000	15.256032000	20.976709000
C	17.414977000	14.699311000	23.566087000
H	17.827761000	14.846049000	24.582365000
H	16.385405000	15.115383000	23.554048000
H	17.336703000	13.606284000	23.387662000
C	16.469903000	20.728006000	24.360598000
C	15.478242000	21.495719000	25.004855000
H	14.571091000	21.013741000	25.397399000
C	15.640189000	22.885823000	25.137287000
H	14.861664000	23.477947000	25.642548000
C	16.785127000	23.518217000	24.623702000
H	16.906001000	24.607659000	24.724673000
C	17.776833000	22.755327000	23.979926000
H	18.676592000	23.244606000	23.576156000
C	17.619824000	21.367891000	23.849068000
H	18.394036000	20.772333000	23.343405000
C	14.614323000	18.556911000	24.691775000
C	14.226403000	18.390317000	26.032969000
H	14.973483000	18.408590000	26.839350000
C	12.870509000	18.191679000	26.345794000
H	12.566447000	18.049733000	27.393974000
C	11.906782000	18.175890000	25.322849000
H	10.844728000	18.024201000	25.568716000
C	12.293854000	18.334920000	23.980752000
H	11.527357000	18.290815000	23.194156000
C	13.651710000	18.515670000	23.652080000
P	14.335419000	18.568872000	21.920422000
C	13.692608000	20.156029000	21.125823000
H	13.764067000	19.920768000	20.040090000
C	12.242604000	20.519844000	21.452267000
H	12.119522000	20.766761000	22.526526000
H	11.529811000	19.708816000	21.200500000
H	11.935442000	21.414116000	20.869238000
C	14.662307000	21.305416000	21.424347000
H	14.389719000	22.205522000	20.834661000
H	15.709025000	21.037450000	21.169157000
H	14.646131000	21.587451000	22.495675000
C	13.299977000	17.230877000	21.085835000
H	12.245962000	17.564445000	21.207525000
C	13.477630000	15.878602000	21.784770000
H	12.764664000	15.136063000	21.369274000
H	13.303404000	15.939066000	22.877134000
H	14.502780000	15.486433000	21.627353000
C	13.602942000	17.122581000	19.586717000
H	14.608934000	16.690698000	19.408590000
H	13.536119000	18.094330000	19.056591000
H	12.873449000	16.441228000	19.101406000
H	15.978353000	16.969386000	22.521788000
H	16.056412000	18.303799000	19.407037000
H	17.260385000	17.241763000	19.739909000
H	17.550583000	18.854519000	19.793773000

FP11'(H)

N	16.961593000	18.704747000	20.357960000
Fe	16.477433000	18.182184000	22.040723000
P	16.302556000	18.958857000	23.986296000
C	17.541961000	18.236684000	25.163777000
C	17.668473000	18.536726000	26.534456000
H	16.987096000	19.254931000	27.016133000
C	18.691178000	17.940800000	27.292200000
H	18.783457000	18.169295000	28.365326000
C	19.603049000	17.064259000	26.676055000
H	20.406297000	16.599091000	27.268432000
C	19.502089000	16.794596000	25.300080000
H	20.235050000	16.122886000	24.825445000
C	18.474058000	17.379672000	24.534188000
P	18.274498000	17.150483000	22.689414000
C	20.043370000	17.577537000	22.145205000
H	20.727219000	17.057004000	22.851683000
C	20.232213000	19.091388000	22.285561000
H	20.011555000	19.443084000	23.313998000
H	21.276831000	19.384202000	22.045226000
H	19.554546000	19.631807000	21.592398000
C	20.362840000	17.097484000	20.724077000
H	20.350112000	15.993080000	20.638227000
H	19.648823000	17.504697000	19.978269000
H	21.378396000	17.436745000	20.425719000
C	18.239167000	15.264224000	22.508077000
H	19.288850000	14.915348000	22.632410000
C	17.735673000	14.905135000	21.103271000
H	18.328025000	15.379734000	20.297804000
H	17.764263000	13.805350000	20.946589000
H	16.689254000	15.251626000	20.977975000
C	17.359082000	14.608320000	23.578041000
H	17.755793000	14.749466000	24.602078000
H	16.332679000	15.031536000	23.549045000
H	17.279459000	13.515554000	23.393660000
C	16.557834000	20.775174000	24.281353000
C	15.917580000	21.509010000	25.300330000
H	15.207710000	21.015211000	25.980340000
C	16.166939000	22.885982000	25.443155000
H	15.658217000	23.452756000	26.238778000
C	17.056174000	23.538279000	24.571580000
H	17.247731000	24.616826000	24.683889000
C	17.693063000	22.810049000	23.549459000
H	18.382803000	23.317574000	22.856756000
C	17.438851000	21.438027000	23.402767000
H	17.898888000	20.857496000	22.587244000
C	14.594505000	18.670685000	24.657641000
C	14.196398000	18.583518000	26.004488000
H	14.931556000	18.692585000	26.815027000
C	12.849776000	18.331043000	26.326370000
H	12.545764000	18.249983000	27.381512000
C	11.899236000	18.176205000	25.303862000
H	10.846265000	17.974395000	25.554557000
C	12.293255000	18.258931000	23.955158000
H	11.542272000	18.106728000	23.166066000
C	13.640528000	18.495124000	23.620962000
P	14.338670000	18.491049000	21.889185000
C	13.636195000	20.070690000	21.113480000
H	13.908725000	19.953418000	20.040386000
C	12.119506000	20.255366000	21.215958000

H	11.805871000	20.434987000	22.265072000
H	11.551851000	19.382210000	20.835142000
H	11.797285000	21.138173000	20.622303000
C	14.401043000	21.277039000	21.667038000
H	14.077488000	22.213054000	21.163467000
H	15.491894000	21.157191000	21.521953000
H	14.225660000	21.403895000	22.755836000
C	13.332186000	17.143784000	21.037122000
H	12.261075000	17.428019000	21.126841000
C	13.543152000	15.788226000	21.718516000
H	12.871001000	15.023180000	21.274453000
H	13.337705000	15.830576000	22.807240000
H	14.589552000	15.446451000	21.591366000
C	13.716035000	17.092707000	19.552412000
H	14.809772000	16.934557000	19.440651000
H	13.453124000	18.024759000	19.011546000
H	13.191449000	16.256962000	19.042364000
H	15.926158000	16.939577000	22.713731000
H	16.347006000	19.104162000	19.634843000
H	17.895511000	18.624097000	19.934751000

GP111’(H)

N	17.153149000	19.606272000	20.653707000
Fe	16.480185000	18.167892000	21.972739000
P	16.363528000	19.047950000	23.902638000
C	17.594980000	18.268116000	25.064259000
C	17.766591000	18.592525000	26.425298000
H	17.132741000	19.360475000	26.895847000
C	18.769842000	17.962088000	27.182382000
H	18.898083000	18.215751000	28.246464000
C	19.616081000	17.016410000	26.575252000
H	20.404678000	16.522819000	27.164855000
C	19.467726000	16.713361000	25.209899000
H	20.150539000	15.986834000	24.740982000
C	18.461851000	17.338072000	24.444605000
P	18.243061000	17.089515000	22.598328000
C	20.042095000	17.450212000	22.067019000
H	20.701145000	16.787183000	22.670040000
C	20.387462000	18.907457000	22.387747000
H	20.256152000	19.136923000	23.464564000
H	21.443550000	19.130575000	22.122846000
H	19.748421000	19.608408000	21.811691000
C	20.261267000	17.138260000	20.582613000
H	20.188871000	16.054596000	20.364603000
H	19.516935000	17.658706000	19.941647000
H	21.270022000	17.472046000	20.255396000
C	18.204266000	15.189105000	22.468954000
H	19.251062000	14.833953000	22.597610000
C	17.690594000	14.783003000	21.081234000
H	18.299730000	15.199104000	20.255346000
H	17.682477000	13.676719000	20.971290000
H	16.653370000	15.154906000	20.941704000
C	17.321020000	14.574217000	23.560484000
H	17.734006000	14.722356000	24.576993000
H	16.308358000	15.030254000	23.536169000
H	17.204680000	13.481004000	23.397720000
C	16.633360000	20.839161000	24.310096000
C	15.640635000	21.714103000	24.806411000
H	14.662659000	21.313080000	25.112003000
C	15.888785000	23.093370000	24.913198000
H	15.102655000	23.758388000	25.305563000
C	17.132654000	23.626431000	24.529575000
H	17.324409000	24.707317000	24.614803000
C	18.131099000	22.764191000	24.034234000
H	19.110695000	23.168763000	23.733092000
C	17.880091000	21.389163000	23.920570000
H	18.658007000	20.718190000	23.522865000
C	14.663028000	18.744003000	24.602976000
C	14.291127000	18.760960000	25.960606000
H	15.036236000	18.988109000	26.737260000
C	12.963401000	18.485550000	26.337447000
H	12.682934000	18.488504000	27.402497000
C	11.999536000	18.205655000	25.354294000
H	10.960234000	17.986583000	25.645442000
C	12.363196000	18.193860000	23.994312000
H	11.601777000	17.955387000	23.236217000
C	13.693170000	18.452624000	23.607524000
P	14.333836000	18.382869000	21.849924000
C	13.553721000	19.938268000	21.065529000
H	13.959848000	19.908805000	20.027929000
C	12.025170000	19.986876000	20.988986000
H	11.570731000	20.003864000	22.001726000
H	11.590934000	19.130552000	20.435307000
H	11.692929000	20.912184000	20.469018000
C	14.108914000	21.183832000	21.764132000
H	13.833536000	22.106346000	21.209088000
H	15.211383000	21.156211000	21.862928000
H	13.697606000	21.278564000	22.789961000
C	13.273008000	17.007836000	21.101248000
H	12.208370000	17.291446000	21.246155000
C	13.521182000	15.668824000	21.800794000
H	12.835429000	14.889638000	21.403479000
H	13.360365000	15.735581000	22.895893000
H	14.563652000	15.329004000	21.638762000
C	13.568033000	16.919458000	19.598905000
H	14.649534000	16.724346000	19.428694000
H	13.306659000	17.851276000	19.056512000
H	12.994967000	16.091081000	19.129810000
H	15.921086000	16.865957000	22.528918000
H	16.427706000	20.017203000	20.049782000
H	17.899170000	19.293735000	20.017102000
H	17.553218000	20.395029000	21.182641000

CP222

N	16.437493000	18.168777000	19.552956000
N	15.383533000	17.524909000	18.677220000
Fe	15.992413000	18.575885000	21.288729000
P	15.675137000	19.723366000	23.205531000
C	15.787489000	18.494768000	24.592369000
C	15.224114000	18.654670000	25.873277000
H	14.645960000	19.556058000	26.120209000
C	15.398340000	17.658042000	26.846788000
H	14.943470000	17.779280000	27.841878000
C	16.161181000	16.513202000	26.553198000
H	16.309724000	15.736880000	27.319476000
C	16.735265000	16.357772000	25.280910000
H	17.344048000	15.465071000	25.070315000
C	16.529836000	17.334215000	24.285234000
P	17.268071000	17.246841000	22.583164000
C	19.092792000	17.311233000	23.165240000
H	19.176088000	16.420114000	23.827338000
C	19.386835000	18.552629000	24.002610000
H	18.840781000	18.535824000	24.964297000
H	20.470769000	18.600140000	24.240582000
H	19.122575000	19.483806000	23.469355000
C	20.061231000	17.152373000	22.000613000
H	20.013887000	16.135995000	21.569414000
H	19.833283000	17.875106000	21.188443000
H	21.107383000	17.317973000	22.336167000
C	17.170703000	15.403572000	22.159881000
H	17.988880000	14.929420000	22.743959000
C	17.454787000	15.226524000	20.666039000
H	18.273398000	15.874710000	20.306083000
H	17.701734000	14.170886000	20.429270000
H	16.552734000	15.501585000	20.087011000
C	15.843794000	14.731964000	22.521192000
H	15.611518000	14.767203000	23.602063000
H	15.002409000	15.207747000	21.978654000
H	15.870046000	13.663927000	22.217290000
C	16.561982000	21.191256000	23.918914000
C	16.257214000	22.467095000	23.396483000
H	15.492302000	22.565492000	22.612925000

C	16.899685000	23.612946000	23.886322000
H	16.633727000	24.605623000	23.489063000
C	17.860881000	23.500165000	24.907923000
H	18.370952000	24.398534000	25.288811000
C	18.144844000	22.238579000	25.456900000
H	18.871254000	22.142464000	26.278669000
C	17.489134000	21.092637000	24.973820000
H	17.688992000	20.120477000	25.441675000
C	13.956931000	20.385771000	23.385991000
C	13.505971000	21.181364000	24.460896000
H	14.208399000	21.516746000	25.238109000
C	12.159939000	21.568679000	24.526352000
H	11.807677000	22.188969000	25.364687000
C	11.263356000	21.163264000	23.519884000
H	10.204359000	21.459078000	23.571475000
C	11.716780000	20.389716000	22.440769000
H	10.999448000	20.085539000	21.666245000
C	13.075256000	20.013523000	22.359864000
P	13.814918000	18.989276000	20.999241000
C	13.181989000	19.915894000	19.457949000
H	13.872750000	19.586475000	18.654757000
C	11.753113000	19.614193000	18.999452000
H	10.996572000	19.891848000	19.758813000
H	11.600570000	18.548882000	18.738630000
H	11.520794000	20.205149000	18.090456000
C	13.430421000	21.417436000	19.650652000
H	13.283286000	21.961716000	18.695287000
H	14.463790000	21.615258000	19.997031000
H	12.732350000	21.856947000	20.389794000
C	12.631783000	17.507038000	21.203109000
H	11.619117000	17.926576000	21.030057000
C	12.705345000	16.986469000	22.644871000
H	12.068804000	16.084147000	22.757799000
H	12.360617000	17.739452000	23.378413000
H	13.742934000	16.707184000	22.918541000
C	12.855630000	16.374524000	20.198896000
H	13.846658000	15.890065000	20.338767000
H	12.744403000	16.693151000	19.140901000
H	12.104924000	15.571589000	20.352719000
Fe	17.849524000	18.819482000	18.652896000
P	17.095942000	19.561859000	16.706023000
C	18.033989000	18.796929000	15.291880000
C	17.875442000	19.169412000	13.942737000
H	17.143248000	19.939825000	13.659893000
C	18.648824000	18.550164000	12.947798000
H	18.529245000	18.848211000	11.894837000
C	19.572045000	17.548679000	13.298670000
H	20.176577000	17.058023000	12.520329000
C	19.721927000	17.166544000	14.642256000
H	20.436922000	16.369953000	14.895912000
C	18.961269000	17.796129000	15.649686000
C	17.496951000	21.364498000	16.449949000
C	17.006016000	22.169152000	15.401022000
H	16.284922000	21.765535000	14.675030000
C	17.417611000	23.506721000	15.293429000
H	17.029907000	24.134491000	14.476333000
C	18.315388000	24.045603000	16.233992000
H	18.631793000	25.097168000	16.155120000
C	18.803855000	23.244676000	17.278624000
H	19.497552000	23.682793000	18.011787000
C	18.400589000	21.897288000	17.387976000
C	15.356619000	19.547550000	16.038078000
C	14.467951000	20.564276000	16.460894000
H	14.832445000	21.355123000	17.133493000
C	13.144186000	20.599796000	15.997405000
H	12.481081000	21.423160000	16.303069000
C	12.669192000	19.595740000	15.135243000
H	11.630554000	19.621801000	14.771792000
C	13.534879000	18.567368000	14.725765000
H	13.177841000	17.783337000	14.040299000
C	14.870221000	18.549613000	15.161503000

H	15.543767000	17.769176000	14.781112000
P	18.977123000	17.337065000	17.452444000
C	17.991439000	15.677184000	17.329407000
H	17.563387000	15.595768000	18.346065000
C	16.841605000	15.805510000	16.323405000
H	17.202880000	15.705459000	15.280326000
H	16.337052000	16.787810000	16.372721000
H	16.077106000	15.017725000	16.492200000
C	18.773719000	14.391909000	17.044273000
H	18.069077000	13.532605000	17.025716000
H	19.539472000	14.163513000	17.808568000
H	19.267464000	14.422210000	16.050494000
C	20.742387000	16.656486000	17.616134000
H	20.739771000	15.800639000	16.911431000
C	21.005102000	16.122680000	19.022173000
H	21.927657000	15.504828000	19.036125000
H	20.178125000	15.498307000	19.410944000
H	21.150030000	16.961724000	19.726944000
C	21.870236000	17.594138000	17.185161000
H	22.022589000	18.408664000	17.916962000
H	21.702725000	18.044907000	16.189837000
H	22.820176000	17.020270000	17.135973000
P	18.956786000	20.712179000	18.708299000
C	18.604267000	21.768039000	20.221980000
H	19.226443000	22.679056000	20.093690000
C	19.004015000	21.044657000	21.504586000
H	18.847573000	21.692206000	22.390467000
H	20.063507000	20.719950000	21.497036000
H	18.372758000	20.139062000	21.624822000
C	17.128760000	22.167222000	20.225571000
H	16.493916000	21.271221000	20.400973000
H	16.815045000	22.629779000	19.269357000
H	16.932133000	22.896531000	21.032357000
C	20.837071000	20.770395000	18.490657000
H	21.144236000	19.798606000	18.931781000
C	21.155048000	20.763072000	16.988716000
H	20.985852000	21.764556000	16.545315000
H	20.520623000	20.045350000	16.430548000
H	22.216363000	20.493699000	16.812440000
C	21.594070000	21.890924000	19.210845000
H	22.683179000	21.785672000	19.015933000
H	21.457426000	21.869267000	20.309150000
H	21.300281000	22.896116000	18.846037000
H	15.266656000	17.359666000	21.743090000
H	18.811734000	18.559108000	19.805025000
H	15.065961000	18.192984000	17.944403000
H	14.566583000	17.168016000	19.208175000
H	15.796529000	16.721737000	18.171778000

DP122

N	16.269707000	18.653900000	20.280402000
N	17.633135000	18.250016000	19.983906000
Fe	15.427473000	18.436922000	21.871712000
P	14.842420000	19.352442000	23.691617000
C	15.488439000	18.446347000	25.175047000
C	15.229593000	18.755353000	26.525126000
H	14.621385000	19.633054000	26.792192000
C	15.770632000	17.955191000	27.545572000
H	15.558120000	18.191748000	28.599647000
C	16.589525000	16.858977000	27.218764000
H	17.009671000	16.227300000	28.016756000
C	16.892770000	16.581719000	25.874622000
H	17.562102000	15.739892000	25.635082000
C	16.350743000	17.375799000	24.843891000
P	16.783131000	17.174399000	23.034260000
C	18.685113000	17.316633000	23.258782000
H	18.875410000	16.815493000	24.231658000
C	19.029309000	18.800657000	23.406007000
H	18.454061000	19.277750000	24.224714000
H	20.109289000	18.941428000	23.622634000
H	18.804213000	19.366675000	22.476528000

C	19.562086000	16.615902000	22.214398000
H	19.355502000	15.529661000	22.147523000
H	19.480493000	17.047901000	21.196738000
H	20.629374000	16.720941000	22.504798000
C	16.571873000	15.315866000	22.737453000
H	17.429003000	14.808676000	23.233558000
C	16.627804000	15.060009000	21.223833000
H	17.606430000	15.345009000	20.789399000
H	16.472162000	13.984610000	20.994905000
H	15.840079000	15.647040000	20.705767000
C	15.264546000	14.787067000	23.335594000
H	15.233412000	14.887175000	24.438323000
H	14.393700000	15.338784000	22.924133000
H	15.135674000	13.711603000	23.088878000
C	15.328559000	21.103926000	24.073770000
C	14.560371000	21.983440000	24.864159000
H	13.597062000	21.657761000	25.282967000
C	15.009167000	23.293713000	25.107341000
H	14.398449000	23.973827000	25.721603000
C	16.228679000	23.735626000	24.566062000
H	16.579524000	24.761435000	24.758011000
C	16.993865000	22.864964000	23.768835000
H	17.945035000	23.209994000	23.334494000
C	16.540848000	21.561059000	23.516390000
H	17.110756000	20.883696000	22.861921000
C	12.987264000	19.397862000	23.832651000
C	12.208910000	19.497112000	25.001924000
H	12.684135000	19.555871000	25.991361000
C	10.804587000	19.499460000	24.917227000
H	10.202458000	19.564283000	25.836697000
C	10.172891000	19.413672000	23.665187000
H	9.073853000	19.413550000	23.599884000
C	10.943940000	19.308655000	22.493527000
H	10.438866000	19.216105000	21.520539000
C	12.350048000	19.287689000	22.570267000
P	13.488710000	19.024064000	21.125624000
C	13.357733000	20.608911000	20.119516000
H	13.836988000	20.307512000	19.163710000
C	11.941471000	21.104414000	19.822272000
H	11.430492000	21.443557000	20.747013000
H	11.306096000	20.333414000	19.341677000
H	11.975430000	21.975725000	19.133278000
C	14.227811000	21.690412000	20.767308000
H	14.256061000	22.601263000	20.131757000
H	15.265000000	21.330914000	20.911229000
H	13.836044000	21.987005000	21.763068000
C	12.576271000	17.768141000	20.060756000
H	11.636951000	18.257275000	19.721069000
C	12.226573000	16.505519000	20.853793000
H	11.652046000	15.800618000	20.215950000
H	11.616138000	16.718732000	21.754061000
H	13.147517000	15.985996000	21.188125000
C	13.445381000	17.430764000	18.843227000
H	14.400258000	16.974907000	19.173060000
H	13.691262000	18.319346000	18.232298000
H	12.922650000	16.710694000	18.179895000
Fe	18.728761000	18.977426000	18.457166000
P	17.577081000	19.472236000	16.702101000
C	18.232603000	18.496691000	15.232519000
C	17.950195000	18.784308000	13.883926000
H	17.234560000	19.578697000	13.624206000
C	18.589347000	18.066020000	12.855492000
H	18.382591000	18.314375000	11.802547000
C	19.494255000	17.039046000	13.173589000
H	19.999580000	16.479421000	12.370681000
C	19.754425000	16.722832000	14.520772000
H	20.458608000	15.910740000	14.759226000
C	19.136043000	17.454133000	15.554666000
C	17.876694000	21.247267000	16.170527000
C	17.178446000	21.928427000	15.152988000
H	16.390420000	21.415946000	14.580547000
C	17.452119000	23.282324000	14.890732000
H	16.900636000	23.809228000	14.096095000
C	18.411590000	23.966992000	15.657672000
H	18.610964000	25.033820000	15.469967000
C	19.112160000	23.289541000	16.672106000
H	19.846692000	23.839915000	17.279172000
C	18.860421000	21.925149000	16.922156000
C	15.739453000	19.430190000	16.336083000
C	14.927406000	20.456747000	16.871538000
H	15.390680000	21.256156000	17.472976000
C	13.547445000	20.491580000	16.616016000
H	12.936843000	21.316158000	17.014213000
C	12.942168000	19.474997000	15.854088000
H	11.858790000	19.498497000	15.660197000
C	13.732545000	18.429276000	15.348295000
H	13.270200000	17.624564000	14.754932000
C	15.118840000	18.412267000	15.581224000
H	15.729039000	17.608291000	15.144185000
P	19.415420000	17.228866000	17.378804000
C	18.336031000	15.680805000	17.731673000
H	18.496098000	15.521997000	18.822888000
C	16.856353000	16.003180000	17.503460000
H	16.651668000	16.141120000	16.422695000
H	16.535749000	16.931040000	18.014141000
H	16.213708000	15.169268000	17.856706000
C	18.728930000	14.397928000	16.994455000
H	18.046702000	13.566308000	17.276657000
H	19.759144000	14.061991000	17.224108000
H	18.648425000	14.527364000	15.894623000
C	21.132492000	16.452672000	17.489105000
H	21.126931000	15.543393000	16.850348000
C	21.401523000	16.038860000	18.940965000
H	22.412876000	15.589322000	19.039131000
H	20.670966000	15.295159000	19.319121000
H	21.350757000	16.928045000	19.603669000
C	22.215218000	17.403936000	16.980634000
H	22.240110000	18.326999000	17.592420000
H	22.051963000	17.698285000	15.925812000
H	23.215772000	16.924753000	17.046689000
P	19.735952000	20.865233000	18.179771000
C	19.626418000	21.967923000	19.714078000
H	19.984747000	22.983698000	19.436923000
C	20.492612000	21.411931000	20.847400000
H	20.357879000	22.003644000	21.778312000
H	21.573624000	21.422475000	20.599270000
H	20.217589000	20.359191000	21.062830000
C	18.146736000	22.044368000	20.113740000
H	17.738080000	21.033247000	20.327384000
H	17.525131000	22.474736000	19.302672000
H	18.006913000	22.675167000	21.015990000
C	21.530949000	20.999245000	17.588556000
H	22.015009000	20.179575000	18.163654000
C	21.561572000	20.637709000	16.097372000
H	21.185962000	21.480908000	15.481412000
H	20.922415000	19.758000000	15.880730000
H	22.595230000	20.408551000	15.762407000
C	22.282214000	22.303838000	17.869190000
H	23.344978000	22.203651000	17.557205000
H	22.284897000	22.579159000	18.942429000
H	21.861889000	23.155709000	17.297290000
H	14.546390000	17.327593000	22.386981000
H	19.934003000	18.756354000	19.409567000
H	15.902642000	19.000516000	19.381709000
H	17.652358000	17.220095000	19.913341000
H	18.168241000	18.452894000	20.835754000

DP222

N	16.526596000	18.405752000	19.622291000
N	15.466432000	17.836209000	18.739972000
Fe	15.877169000	18.557461000	21.418939000
P	15.566670000	19.663620000	23.282667000

C	15.873310000	18.530058000	24.731521000
C	15.445378000	18.732313000	26.058517000
H	14.921169000	19.656194000	26.341352000
C	15.689252000	17.750573000	27.036307000
H	15.333134000	17.902973000	28.067297000
C	16.394302000	16.582367000	26.698573000
H	16.592676000	15.815688000	27.463999000
C	16.857314000	16.395510000	25.382699000
H	17.442326000	15.494333000	25.141105000
C	16.575893000	17.353590000	24.389071000
P	17.210071000	17.286832000	22.639493000
C	19.058310000	17.416592000	23.165082000
H	19.202035000	16.547608000	23.846771000
C	19.316859000	18.696853000	23.956526000
H	18.828632000	18.669211000	24.949717000
H	20.406578000	18.837413000	24.124924000
H	18.943693000	19.587163000	23.417509000
C	19.997132000	17.263678000	21.973838000
H	19.951559000	16.240353000	21.556430000
H	19.734823000	17.968504000	21.153129000
H	21.051803000	17.448011000	22.275427000
C	17.196838000	15.443160000	22.194112000
H	18.079807000	15.001851000	22.706074000
C	17.380392000	15.324228000	20.676996000
H	18.179979000	15.980487000	20.287421000
H	17.591351000	14.274402000	20.382636000
H	16.444510000	15.643557000	20.175444000
C	15.939402000	14.685834000	22.627091000
H	15.749574000	14.733134000	23.716431000
H	15.046543000	15.090730000	22.110194000
H	16.026512000	13.613979000	22.346060000
C	16.365506000	21.248137000	23.846442000
C	15.969953000	22.439257000	23.196549000
H	15.164524000	22.399516000	22.446565000
C	16.586438000	23.662088000	23.495958000
H	16.262855000	24.579429000	22.979072000
C	17.619592000	23.716813000	24.451950000
H	18.112274000	24.674215000	24.682186000
C	18.004797000	22.541868000	25.120710000
H	18.796579000	22.576453000	25.885874000
C	17.377167000	21.318915000	24.825732000
H	17.673207000	20.413088000	25.370864000
C	13.808206000	20.198112000	23.554716000
C	13.336187000	20.951276000	24.649814000
H	14.040386000	21.344743000	25.398485000
C	11.964209000	21.224814000	24.774474000
H	11.595333000	21.808020000	25.632746000
C	11.064025000	20.749571000	23.803107000
H	9.985931000	20.950465000	23.903476000
C	11.538985000	20.028372000	22.694188000
H	10.821303000	19.673474000	21.941176000
C	12.919011000	19.766131000	22.556874000
P	13.709703000	18.887672000	21.116593000
C	13.078489000	19.991568000	19.672973000
H	13.740511000	19.714453000	18.825976000
C	11.631597000	19.804118000	19.211150000
H	10.900825000	20.063258000	20.003198000
H	11.419575000	18.771682000	18.868905000
H	11.418893000	20.475909000	18.352912000
C	13.399441000	21.453614000	20.002798000
H	13.257870000	22.095009000	19.107217000
H	14.450828000	21.564683000	20.334476000
H	12.739709000	21.847562000	20.802465000
C	12.525401000	17.388134000	21.080247000
H	11.511626000	17.806665000	20.909550000
C	12.547063000	16.687288000	22.442872000
H	11.948540000	15.751906000	22.411300000
H	12.136755000	17.328080000	23.247330000
H	13.585912000	16.422984000	22.726913000
C	12.840278000	16.403003000	19.950783000
H	13.843591000	15.947374000	20.090883000

H	12.796119000	16.865320000	18.941389000
H	12.104098000	15.571321000	19.944500000
Fe	17.986115000	18.828748000	18.541985000
P	17.268978000	19.560682000	16.617308000
C	18.119612000	18.722231000	15.184693000
C	17.967072000	19.082470000	13.831869000
H	17.253053000	19.868162000	13.543055000
C	18.726942000	18.437116000	12.839761000
H	18.615194000	18.730090000	11.784049000
C	19.631739000	17.423562000	13.200216000
H	20.233556000	16.920180000	12.427228000
C	19.766244000	17.044185000	14.548745000
H	20.466785000	16.236267000	14.809926000
C	19.016032000	17.693039000	15.550742000
C	17.739924000	21.344649000	16.323919000
C	17.277683000	22.147538000	15.261388000
H	16.553010000	21.748423000	14.535565000
C	17.717059000	23.476972000	15.144039000
H	17.354752000	24.103967000	14.314201000
C	18.607659000	24.009172000	16.094834000
H	18.944508000	25.054420000	16.010822000
C	19.057560000	23.210985000	17.161349000
H	19.733655000	23.648401000	17.911686000
C	18.632705000	21.871211000	17.275580000
C	15.502079000	19.656234000	16.021414000
C	14.677625000	20.661717000	16.585356000
H	15.123403000	21.404068000	17.266825000
C	13.312971000	20.730112000	16.271610000
H	12.696812000	21.538818000	16.693083000
C	12.730505000	19.771197000	15.421751000
H	11.656173000	19.819291000	15.186888000
C	13.533659000	18.758052000	14.868851000
H	13.091028000	18.009923000	14.192429000
C	14.908632000	18.707778000	15.154551000
H	15.531225000	17.932883000	14.684643000
P	19.003977000	17.261924000	17.368132000
C	17.946790000	15.640834000	17.232736000
H	17.605789000	15.507697000	18.278209000
C	16.722118000	15.873008000	16.341463000
H	16.992211000	15.806543000	15.267586000
H	16.274850000	16.873751000	16.484404000
H	15.934557000	15.114086000	16.537246000
C	18.654826000	14.356351000	16.789823000
H	17.922952000	13.519495000	16.752784000
H	19.466656000	14.041822000	17.472527000
H	19.079090000	14.456037000	15.768655000
C	20.727024000	16.490901000	17.557950000
H	20.767847000	15.676469000	16.806380000
C	20.893609000	15.882763000	18.950197000
H	21.839307000	15.302989000	19.015989000
H	20.064038000	15.203579000	19.229048000
H	20.931796000	16.683398000	19.710110000
C	21.866957000	17.463445000	17.259770000
H	21.917266000	18.261992000	18.024094000
H	21.767949000	17.943013000	16.267235000
H	22.839501000	16.925389000	17.278004000
P	19.083812000	20.690298000	18.648403000
C	18.573307000	21.774626000	20.103015000
H	19.145495000	22.724235000	20.015441000
C	18.889311000	21.092383000	21.432080000
H	18.640253000	21.756696000	22.284903000
H	19.954712000	20.801061000	21.525078000
H	18.274346000	20.173434000	21.528245000
C	17.074482000	22.066192000	19.979570000
H	16.498532000	21.122445000	20.094173000
H	16.811794000	22.518250000	19.002301000
H	16.756120000	22.766675000	20.775574000
C	20.980295000	20.773889000	18.612136000
H	21.251404000	19.811633000	19.097459000
C	21.440512000	20.734747000	17.148492000
H	21.330873000	21.730012000	16.671847000

H	20.842885000	20.014641000	16.553556000
H	22.508795000	20.442224000	17.071600000
C	21.660496000	21.912921000	19.379104000
H	22.765354000	21.806901000	19.309136000
H	21.401552000	21.920580000	20.455458000
H	21.408241000	22.908338000	18.959785000
H	15.208490000	17.245013000	21.756120000
H	18.904939000	18.598720000	19.737446000
H	15.426084000	18.333312000	17.822143000
H	14.535337000	17.841827000	19.203992000
H	15.683955000	16.846537000	18.533080000

^{TS}DP222

N	16.690665000	18.474039000	19.734460000
N	15.520807000	17.845427000	18.751174000
Fe	16.001564000	18.621425000	21.476050000
P	15.696825000	19.753194000	23.340663000
C	15.971875000	18.606888000	24.788313000
C	15.534159000	18.817621000	26.110688000
H	15.030562000	19.754583000	26.388120000
C	15.740572000	17.827945000	27.089465000
H	15.374357000	17.986918000	28.115953000
C	16.421992000	16.644060000	26.758738000
H	16.592299000	15.871517000	27.525045000
C	16.898553000	16.449287000	25.448457000
H	17.469353000	15.537429000	25.213290000
C	16.649248000	17.413573000	24.452536000
P	17.308791000	17.341427000	22.713772000
C	19.149821000	17.477476000	23.266828000
H	19.285861000	16.602925000	23.942976000
C	19.398864000	18.749674000	24.072518000
H	18.887874000	18.718818000	25.053980000
H	20.485629000	18.879515000	24.266744000
H	19.046665000	19.646922000	23.531142000
C	20.104801000	17.337538000	22.086869000
H	20.073994000	16.316066000	21.664595000
H	19.845507000	18.043976000	21.267072000
H	21.153999000	17.530718000	22.401536000
C	17.319943000	15.497374000	22.267888000
H	18.198844000	15.067446000	22.796000000
C	17.529870000	15.378645000	20.753992000
H	18.315545000	16.056243000	20.372951000
H	17.778314000	14.334675000	20.467637000
H	16.596131000	15.668230000	20.232738000
C	16.065235000	14.724206000	22.679849000
H	15.862621000	14.758767000	23.767218000
H	15.174423000	15.124915000	22.156630000
H	16.166235000	13.656096000	22.389373000
C	16.499621000	21.328382000	23.929308000
C	16.112937000	22.530206000	23.293620000
H	15.311540000	22.505349000	22.538769000
C	16.729994000	23.747065000	23.615753000
H	16.412682000	24.672389000	23.109246000
C	17.755064000	23.786029000	24.581084000
H	18.248620000	24.738667000	24.828461000
C	18.129623000	22.600994000	25.237895000
H	18.912997000	22.622637000	26.012172000
C	17.500857000	21.384358000	24.920448000
H	17.786438000	20.471730000	25.459221000
C	13.940393000	20.295577000	23.612341000
C	13.476888000	21.065235000	24.699786000
H	14.188355000	21.480349000	25.429640000
C	12.103586000	21.323074000	24.842762000
H	11.741708000	21.919415000	25.695010000
C	11.193269000	20.811034000	23.900329000
H	10.113940000	20.995936000	24.017054000
C	11.659184000	20.070558000	22.799882000
H	10.932321000	19.682649000	22.072783000
C	13.040606000	19.828192000	22.640312000
P	13.820118000	18.917173000	21.214014000
C	13.136739000	19.954014000	19.751666000
H	13.714407000	19.574957000	18.884819000
C	11.653250000	19.837260000	19.393467000
H	10.999708000	20.286360000	20.167178000
H	11.324781000	18.791781000	19.229261000
H	11.450977000	20.387119000	18.449494000
C	13.561994000	21.409150000	19.973727000
H	13.366010000	22.022100000	19.069292000
H	14.642362000	21.475923000	20.207113000
H	13.000021000	21.868852000	20.812579000
C	12.660510000	17.403280000	21.253812000
H	11.636035000	17.812519000	21.129761000
C	12.754802000	16.723818000	22.624122000
H	12.165651000	15.782051000	22.636542000
H	12.374952000	17.374991000	23.435502000
H	13.808533000	16.477191000	22.865493000
C	12.917789000	16.412535000	20.115272000
H	13.924224000	15.948386000	20.201472000
H	12.821194000	16.873866000	19.109373000
H	12.183139000	15.580252000	20.150965000
Fe	18.084297000	18.906246000	18.655746000
P	17.375619000	19.644628000	16.722075000
C	18.250946000	18.823696000	15.293807000
C	18.126960000	19.203837000	13.943393000
H	17.436881000	20.010103000	13.653555000
C	18.885601000	18.551845000	12.954634000
H	18.797086000	18.860591000	11.901234000
C	19.756678000	17.508978000	13.314805000
H	20.353290000	16.996836000	12.543560000
C	19.864547000	17.112576000	14.660766000
H	20.537898000	16.281796000	14.922127000
C	19.121651000	17.773390000	15.660087000
C	17.835666000	21.433437000	16.443425000
C	17.362409000	22.246075000	15.393089000
H	16.629202000	21.854174000	14.671784000
C	17.802896000	23.575837000	15.282122000
H	17.431620000	24.210646000	14.462250000
C	18.706801000	24.098138000	16.225774000
H	19.045515000	25.143088000	16.145782000
C	19.167578000	23.290326000	17.280376000
H	19.854361000	23.719275000	18.025939000
C	18.739585000	21.951278000	17.389612000
C	15.616564000	19.730236000	16.106255000
C	14.770590000	20.697611000	16.702341000
H	15.196824000	21.413277000	17.423662000
C	13.408798000	20.760661000	16.371119000
H	12.773427000	21.539457000	16.819720000
C	12.854493000	19.835034000	15.468046000
H	11.783464000	19.879173000	15.217861000
C	13.681303000	18.860766000	14.880816000
H	13.260115000	18.139367000	14.163004000
C	15.051775000	18.815667000	15.186738000
H	15.692173000	18.072154000	14.690702000
P	19.094501000	17.335950000	17.474798000
C	18.014653000	15.728153000	17.333369000
H	17.601042000	15.649494000	18.358312000
C	16.853433000	15.940759000	16.354198000
H	17.190867000	15.844494000	15.302255000
H	16.396887000	16.946120000	16.434931000
H	16.051431000	15.190195000	16.521328000
C	18.720885000	14.411197000	16.996738000
H	17.975898000	13.586024000	16.961201000
H	19.486389000	14.120260000	17.740201000
H	19.203954000	14.449858000	15.997611000
C	20.815138000	16.555281000	17.667200000
H	20.836449000	15.714674000	16.943771000
C	20.999759000	15.997197000	19.077531000
H	21.947698000	15.422220000	19.152299000
H	20.175483000	15.327790000	19.391442000
H	21.044211000	16.825675000	19.806871000
C	21.963946000	17.502128000	17.320759000
H	22.048446000	18.314788000	18.066936000

H	21.852285000	17.963232000	16.321101000
H	22.926025000	16.945434000	17.328982000
P	19.200335000	20.760641000	18.749982000
C	18.737292000	21.846446000	20.217671000
H	19.326488000	22.785011000	20.124668000
C	19.058845000	21.152144000	21.539050000
H	18.821997000	21.812938000	22.398359000
H	20.122551000	20.850878000	21.621268000
H	18.435209000	20.238546000	21.632199000
C	17.242996000	22.165101000	20.118261000
H	16.656712000	21.228904000	20.241627000
H	16.971228000	22.621144000	19.145535000
H	16.948562000	22.868354000	20.920543000
C	21.096155000	20.812213000	18.671134000
H	21.362189000	19.844481000	19.148504000
C	21.520847000	20.768380000	17.197006000
H	21.406850000	21.764050000	16.722196000
H	20.903749000	20.051974000	16.617431000
H	22.584792000	20.468144000	17.094691000
C	21.811244000	21.939045000	19.423793000
H	22.912504000	21.816064000	19.329113000
H	21.576788000	21.949435000	20.505802000
H	21.564958000	22.938643000	19.010832000
H	15.358825000	17.281856000	21.760114000
H	19.012571000	18.677134000	19.842386000
H	15.234800000	18.556707000	18.055322000
H	14.716007000	17.506302000	19.301196000
H	15.933669000	17.056396000	18.234304000

EP11′

N	16.860455000	19.312131000	20.143069000
Fe	15.835633000	18.498546000	21.683614000
P	15.321342000	19.517247000	23.464072000
C	15.815461000	18.582907000	24.980671000
C	15.558310000	18.981390000	26.307990000
H	15.035574000	19.924333000	26.521757000
C	16.002472000	18.184969000	27.374125000
H	15.795962000	18.492470000	28.410610000
C	16.721483000	17.002329000	27.117159000
H	17.068898000	16.376164000	27.953207000
C	17.017554000	16.630160000	25.796898000
H	17.606428000	15.718400000	25.612992000
C	16.570216000	17.421712000	24.718611000
P	17.015624000	17.091232000	22.942926000
C	18.881332000	16.840983000	23.188861000
H	18.967670000	16.007877000	23.920654000
C	19.527601000	18.090173000	23.787507000
H	19.096426000	18.355527000	24.773244000
H	20.617442000	17.932882000	23.929896000
H	19.409470000	18.958125000	23.110127000
C	19.551102000	16.428115000	21.881134000
H	19.199250000	15.440909000	21.520295000
H	19.356610000	17.180248000	21.084510000
H	20.649694000	16.353820000	22.021651000
C	16.459540000	15.289202000	22.715756000
H	17.269069000	14.665481000	23.153885000
C	16.348061000	14.991401000	21.217876000
H	17.306544000	15.149119000	20.689648000
H	16.032632000	13.941069000	21.043251000
H	15.596378000	15.657207000	20.747417000
C	15.148377000	14.988724000	23.450168000
H	15.260570000	15.025845000	24.550275000
H	14.355814000	15.712201000	23.170347000
H	14.786088000	13.973951000	23.181276000
C	16.088490000	21.162567000	23.787459000
C	15.380109000	22.371561000	23.633216000
H	14.314669000	22.355912000	23.364751000
C	16.028916000	23.600582000	23.838575000
H	15.466817000	24.539186000	23.716556000
C	17.384574000	23.632194000	24.207931000
H	17.889177000	24.596457000	24.372803000
C	18.093127000	22.428005000	24.372680000
H	19.152064000	22.445168000	24.672180000
C	17.451018000	21.200197000	24.158220000
H	18.007935000	20.261784000	24.288686000
C	13.500871000	19.835167000	23.578038000
C	12.802758000	20.288480000	24.714041000
H	13.333125000	20.532543000	25.644574000
C	11.407198000	20.439124000	24.663002000
H	10.863256000	20.785915000	25.554711000
C	10.706632000	20.140422000	23.481051000
H	9.611274000	20.243520000	23.448326000
C	11.401752000	19.713455000	22.337407000
H	10.841116000	19.481409000	21.420283000
C	12.803312000	19.570467000	22.376242000
P	13.858594000	19.086421000	20.936477000
C	13.706300000	20.565784000	19.775802000
H	14.376531000	20.271705000	18.937297000
C	12.312877000	20.841685000	19.208567000
H	11.600524000	21.122307000	20.010704000
H	11.891496000	19.985343000	18.648654000
H	12.357413000	21.702132000	18.508276000
C	14.279513000	21.813738000	20.450889000
H	14.416559000	22.630558000	19.712108000
H	15.257493000	21.624219000	20.931185000
H	13.591761000	22.188706000	21.235662000
C	12.843441000	17.768399000	20.072780000
H	11.875580000	18.255055000	19.826860000
C	12.563605000	16.564936000	20.975337000
H	11.844984000	15.880638000	20.477507000
H	12.126412000	16.860893000	21.950252000
H	13.487837000	15.989871000	21.177206000
C	13.548175000	17.376022000	18.771369000
H	14.563732000	16.983855000	18.975742000
H	13.657553000	18.233380000	18.083194000
H	12.978401000	16.588448000	18.237196000
Fe	18.185238000	18.885463000	18.769621000
P	17.192780000	19.485406000	16.869509000
C	18.080237000	18.691902000	15.428615000
C	17.805888000	18.997458000	14.081648000
H	17.027005000	19.731180000	13.826028000
C	18.521435000	18.360349000	13.055610000
H	18.314132000	18.612631000	12.004359000
C	19.496081000	17.396064000	13.371410000
H	20.049686000	16.886922000	12.567623000
C	19.763532000	17.078144000	14.712623000
H	20.520371000	16.315386000	14.945850000
C	19.068351000	17.735594000	15.749509000
C	17.484960000	21.301397000	16.591269000
C	16.912388000	22.071866000	15.558905000
H	16.225182000	21.611894000	14.834152000
C	17.196360000	23.443562000	15.462332000
H	16.740843000	24.040214000	14.657183000
C	18.049202000	24.054965000	16.398813000
H	18.258231000	25.133815000	16.334463000
C	18.639825000	23.288261000	17.416572000
H	19.308616000	23.778107000	18.139718000
C	18.370196000	21.907595000	17.510237000
C	15.449226000	19.290110000	16.248559000
C	14.471916000	20.286252000	16.465618000
H	14.749225000	21.233841000	16.950590000
C	13.149917000	20.100938000	16.026375000
H	12.409707000	20.900301000	16.176434000
C	12.774813000	18.906214000	15.389271000
H	11.738114000	18.762383000	15.048742000
C	13.735559000	17.901800000	15.180876000
H	13.456395000	16.963531000	14.677238000
C	15.060770000	18.095506000	15.599492000
H	15.806055000	17.313502000	15.391557000
P	19.333621000	17.464068000	17.562655000
C	18.393748000	15.841900000	17.882775000
H	18.840037000	15.479836000	18.834236000

C	16.920436000	16.169948000	18.132738000
H	16.434837000	16.588550000	17.231887000
H	16.789589000	16.900983000	18.960991000
H	16.358215000	15.254801000	18.407483000
C	18.558218000	14.760566000	16.814189000
H	18.051260000	13.828563000	17.143919000
H	19.618295000	14.499941000	16.621341000
H	18.099098000	15.066011000	15.851700000
C	21.108758000	16.820873000	17.660822000
H	21.046876000	15.853146000	17.116764000
C	21.487059000	16.526830000	19.115011000
H	22.511812000	16.102284000	19.161316000
H	20.807054000	15.793476000	19.586504000
H	21.468927000	17.449117000	19.729933000
C	22.184121000	17.665921000	16.971923000
H	22.446128000	18.560800000	17.567807000
H	21.897947000	17.995788000	15.956225000
H	23.111000000	17.062456000	16.875594000
P	19.216590000	20.753888000	18.692816000
C	19.309387000	21.793407000	20.260023000
H	19.944448000	22.653111000	19.961115000
C	20.023741000	21.017999000	21.367154000
H	20.184418000	21.659786000	22.257240000
H	21.008039000	20.621199000	21.047095000
H	19.417709000	20.145454000	21.681233000
C	17.959376000	22.355581000	20.719026000
H	17.358657000	21.605758000	21.267376000
H	17.350400000	22.737983000	19.876952000
H	18.121135000	23.196707000	21.422647000
C	20.993755000	20.813215000	18.033675000
H	21.422537000	19.880319000	18.454653000
C	20.980863000	20.716931000	16.503143000
H	20.613905000	21.660078000	16.050733000
H	20.338059000	19.898083000	16.130610000
H	22.006599000	20.541060000	16.120938000
C	21.855547000	21.997589000	18.485300000
H	22.870042000	21.896627000	18.044113000
H	21.985002000	22.053406000	19.583042000
H	21.446989000	22.965703000	18.129129000
H	14.877804000	17.476724000	22.206082000
H	19.192213000	18.564782000	19.870254000
H	16.259560000	20.069737000	19.802416000
H	17.418597000	19.719216000	20.923985000

^{TS}EP11222(H)

N	17.013408000	18.019123000	19.401224000
N	15.607583000	17.531642000	18.805696000
Fe	18.336670000	18.959824000	18.327877000
P	17.589954000	19.440393000	16.368124000
C	18.592340000	18.621767000	15.033681000
C	18.472929000	18.884702000	13.656233000
H	17.710119000	19.586055000	13.286159000
C	19.339536000	18.257592000	12.743337000
H	19.252559000	18.473468000	11.667328000
C	20.320499000	17.363740000	13.206714000
H	21.004631000	16.878678000	12.493288000
C	20.432102000	17.084942000	14.581307000
H	21.204173000	16.380285000	14.926434000
C	19.572925000	17.711256000	15.505440000
C	17.663946000	21.260368000	16.010186000
C	17.048750000	21.900257000	14.917230000
H	16.500494000	21.313549000	14.163543000
C	17.105361000	23.300097000	14.806707000
H	16.620721000	23.804210000	13.956305000
C	17.761552000	24.056593000	15.794787000
H	17.788576000	25.154683000	15.719204000
C	18.373998000	23.416867000	16.887425000
H	18.865710000	24.025273000	17.661119000
C	18.335864000	22.012290000	16.999042000
C	15.824345000	19.095041000	15.927647000
C	14.847833000	19.794397000	16.683957000
H	15.163247000	20.615573000	17.349628000
C	13.491064000	19.444742000	16.597493000
H	12.744263000	19.997318000	17.188378000
C	13.088548000	18.386186000	15.758196000
H	12.026923000	18.102250000	15.698469000
C	14.046530000	17.705603000	14.988829000
H	13.735831000	16.888308000	14.319634000
C	15.406672000	18.059855000	15.067039000
H	16.146616000	17.517565000	14.460039000
P	19.566506000	17.434853000	17.348121000
C	18.742285000	15.696955000	17.428849000
H	18.475430000	15.607982000	18.506304000
C	17.450991000	15.697200000	16.602937000
H	17.666948000	15.557031000	15.524050000
H	16.911479000	16.663327000	16.668885000
H	16.767416000	14.878364000	16.912559000
C	19.606031000	14.490965000	17.046609000
H	19.003323000	13.558907000	17.109316000
H	20.481352000	14.354103000	17.709969000
H	19.971231000	14.569126000	16.001281000
C	21.348152000	16.978079000	17.764295000
H	21.582377000	16.068301000	17.173185000
C	21.428037000	16.634243000	19.256957000
H	22.451358000	16.295678000	19.524716000
H	20.723819000	15.829141000	19.552425000
H	21.185288000	17.525978000	19.871697000
C	22.359596000	18.060694000	17.388661000
H	22.206412000	18.977179000	17.989437000
H	22.304523000	18.335967000	16.317676000
H	23.391044000	17.700301000	17.589374000
P	19.115484000	20.996276000	18.356063000
C	18.643036000	21.985405000	19.890880000
H	18.988137000	23.028161000	19.721800000
C	19.335029000	21.422820000	21.136578000
H	19.089375000	22.036316000	22.029440000
H	20.439504000	21.403878000	21.037635000
H	19.004866000	20.381077000	21.330115000
C	17.118206000	21.986808000	20.038947000
H	16.733330000	20.954085000	20.181023000
H	16.614343000	22.411195000	19.147123000
H	16.807259000	22.583952000	20.922207000
C	20.947927000	21.380745000	18.082285000
H	21.440448000	20.591999000	18.691540000
C	21.270717000	21.141266000	16.601323000
H	20.899749000	21.979948000	15.977227000
H	20.804311000	20.210130000	16.221569000
H	22.366536000	21.064360000	16.444639000
C	21.451073000	22.749793000	18.549232000
H	22.543495000	22.830728000	18.360703000
H	21.295992000	22.921823000	19.632289000
H	20.967820000	23.579545000	17.993864000
H	19.251128000	18.829662000	19.571154000
H	15.193745000	18.338282000	18.289632000
H	14.943933000	17.172234000	19.517035000
H	15.824938000	16.790881000	18.115343000
H	17.337549000	17.151120000	19.857697000
H	16.709063000	18.632749000	20.176856000

DP1′

N	16.758573000	18.286823000	19.903978000
Fe	16.170375000	18.625153000	21.556699000
P	15.533245000	19.633171000	23.420790000
C	15.922247000	18.558843000	24.876099000
C	15.414236000	18.724652000	26.179944000
H	14.674956000	19.508260000	26.398091000
C	15.860148000	17.890357000	27.216770000
H	15.455958000	18.016810000	28.232677000
C	16.832621000	16.905775000	26.961434000
H	17.195002000	16.263671000	27.778693000
C	17.344555000	16.740512000	25.664895000
H	18.113470000	15.974421000	25.480873000

C	16.876166000	17.550884000	24.610187000
P	17.517502000	17.443382000	22.874508000
C	19.353439000	17.786429000	23.212044000
H	19.694740000	16.910747000	23.807827000
C	19.538390000	19.044765000	24.063356000
H	19.174084000	18.893547000	25.097956000
H	20.614340000	19.312585000	24.121563000
H	19.001812000	19.915398000	23.645762000
C	20.132135000	17.816743000	21.894475000
H	20.193774000	16.808652000	21.441435000
H	19.654106000	18.481366000	21.148038000
H	21.173254000	18.164129000	22.062212000
C	17.578314000	15.584710000	22.511594000
H	18.548250000	15.249139000	22.938446000
C	17.596236000	15.351834000	20.996670000
H	18.261732000	16.052213000	20.463841000
H	17.918129000	14.314374000	20.772437000
H	16.580226000	15.487191000	20.576883000
C	16.443700000	14.786920000	23.159828000
H	16.478438000	14.789809000	24.264608000
H	15.454683000	15.183005000	22.854148000
H	16.491078000	13.729641000	22.823819000
C	16.166208000	21.283040000	23.948619000
C	15.685482000	22.427160000	23.275341000
H	14.877834000	22.326889000	22.534370000
C	16.224581000	23.692680000	23.549460000
H	15.841087000	24.576512000	23.016822000
C	17.251636000	23.829969000	24.501462000
H	17.679214000	24.821915000	24.713384000
C	17.717090000	22.698435000	25.192836000
H	18.506414000	22.800909000	25.953376000
C	17.175099000	21.430399000	24.921834000
H	17.537702000	20.554226000	25.476201000
C	13.712278000	19.931681000	23.468102000
C	13.024825000	20.574047000	24.517466000
H	13.577634000	20.992169000	25.371824000
C	11.629119000	20.706863000	24.457514000
H	11.090055000	21.205262000	25.277536000
C	10.921056000	20.205741000	23.349038000
H	9.825368000	20.301766000	23.306335000
C	11.608631000	19.601144000	22.284466000
H	11.041594000	19.233881000	21.417011000
C	13.013082000	19.477492000	22.331354000
P	14.054624000	18.826335000	20.946705000
C	13.616840000	19.973536000	19.506932000
H	14.491303000	19.847992000	18.830358000
C	12.337608000	19.632129000	18.738655000
H	11.437891000	19.670824000	19.385543000
H	12.374903000	18.640115000	18.250006000
H	12.187593000	20.379361000	17.935145000
C	13.589453000	21.422292000	20.008050000
H	13.643282000	22.128727000	19.155252000
H	14.441283000	21.644025000	20.677435000
H	12.657170000	21.636253000	20.568566000
C	13.150429000	17.229475000	20.533261000
H	12.148652000	17.553599000	20.182518000
C	12.962681000	16.338730000	21.761320000
H	12.417801000	15.412973000	21.481758000
H	12.388938000	16.841422000	22.564256000
H	13.940347000	16.035131000	22.186158000
C	13.860725000	16.500028000	19.393152000
H	14.846620000	16.117805000	19.729816000
H	14.023343000	17.152112000	18.513194000
H	13.266797000	15.626232000	19.054897000
Fe	17.859303000	18.772872000	18.557217000
P	17.140891000	19.668096000	16.641650000
C	17.767942000	18.698908000	15.182617000
C	17.554493000	19.061187000	13.838057000
H	16.907631000	19.913154000	13.584863000
C	18.165842000	18.329932000	12.807071000
H	18.005967000	18.623871000	11.758482000
C	18.985819000	17.230206000	13.115948000
H	19.475068000	16.662363000	12.309762000
C	19.171318000	16.842272000	14.453468000
H	19.799782000	15.967039000	14.675180000
C	18.556554000	17.568652000	15.494308000
C	17.873857000	21.350138000	16.342861000
C	17.572036000	22.163966000	15.231251000
H	16.854073000	21.827779000	14.469858000
C	18.164098000	23.429630000	15.109073000
H	17.931375000	24.061169000	14.238089000
C	19.039093000	23.896825000	16.107792000
H	19.493032000	24.895873000	16.020544000
C	19.324519000	23.095380000	17.223840000
H	19.993329000	23.481285000	18.006841000
C	18.752349000	21.810749000	17.340162000
C	15.388903000	20.022754000	16.156286000
C	14.806134000	21.251327000	16.541625000
H	15.380330000	21.964211000	17.151986000
C	13.511231000	21.586674000	16.120941000
H	13.080882000	22.558765000	16.406340000
C	12.766485000	20.686934000	15.336380000
H	11.751557000	20.952566000	15.003634000
C	13.321800000	19.444614000	14.988811000
H	12.744975000	18.730328000	14.381320000
C	14.627154000	19.114857000	15.391644000
H	15.064352000	18.157626000	15.076141000
P	18.589449000	17.099019000	17.287577000
C	17.367114000	15.637538000	17.282201000
H	17.247739000	15.420528000	18.365197000
C	16.019817000	16.103930000	16.722412000
H	16.074744000	16.227669000	15.621499000
H	15.700114000	17.072023000	17.155857000
H	15.228738000	15.356849000	16.935921000
C	17.836590000	14.369551000	16.564196000
H	17.051974000	13.586979000	16.644894000
H	18.765412000	13.940373000	16.986462000
H	17.999642000	14.553098000	15.482179000
C	20.249389000	16.237583000	17.456646000
H	20.287882000	15.520706000	16.610031000
C	20.355971000	15.453799000	18.764797000
H	21.355758000	14.977137000	18.839398000
H	19.599488000	14.650603000	18.853333000
H	20.244545000	16.129344000	19.635439000
C	21.401118000	17.230365000	17.314428000
H	21.414056000	17.929123000	18.173212000
H	21.333123000	17.824968000	16.383051000
H	22.371225000	16.690734000	17.302340000
P	19.028771000	20.659500000	18.767768000
C	18.443120000	21.787076000	20.159477000
H	19.066669000	22.703871000	20.088413000
C	18.640880000	21.134896000	21.520116000
H	18.353599000	21.826370000	22.337138000
H	19.684086000	20.811942000	21.693362000
H	17.988316000	20.233247000	21.615232000
C	16.973774000	22.139799000	19.933408000
H	16.360391000	21.217736000	19.993800000
H	16.792686000	22.618346000	18.950729000
H	16.624646000	22.837613000	20.720281000
C	20.921546000	20.627604000	18.895667000
H	21.101262000	19.650927000	19.394183000
C	21.513138000	20.575359000	17.478103000
H	21.549039000	21.587635000	17.028675000
H	20.915215000	19.938467000	16.797499000
H	22.548715000	20.177663000	17.499925000
C	21.591604000	21.724112000	19.732389000
H	22.691116000	21.564265000	19.732281000
H	21.263754000	21.724490000	20.789347000
H	21.416094000	22.738160000	19.319657000
H	15.501288000	17.355420000	21.911376000
H	19.130239000	18.348208000	19.246291000
H	16.327398000	17.484376000	19.389129000

DP11′

N	16.467491000	18.421583000	19.550622000
Fe	15.937542000	18.787385000	21.483666000
P	15.459948000	19.594546000	23.455630000
C	16.109531000	18.477513000	24.782169000
C	15.773419000	18.546463000	26.148445000
H	14.996991000	19.236564000	26.504356000
C	16.446540000	17.736563000	27.075088000
H	16.177131000	17.787745000	28.140818000
C	17.470660000	16.870660000	26.647366000
H	18.003230000	16.243388000	27.378076000
C	17.811461000	16.801382000	25.288658000
H	18.604020000	16.109215000	24.964211000
C	17.129397000	17.604108000	24.350239000
P	17.388149000	17.490096000	22.525576000
C	19.253665000	17.645740000	22.269169000
H	19.682457000	16.625715000	22.369903000
C	19.921766000	18.583412000	23.281215000
H	19.872310000	18.193218000	24.314655000
H	20.992679000	18.719290000	23.023560000
H	19.450371000	19.587539000	23.273891000
C	19.512111000	18.174181000	20.854437000
H	18.990750000	17.551382000	20.071810000
H	19.160639000	19.219158000	20.808044000
H	20.588586000	18.153359000	20.605498000
C	17.142827000	15.624913000	22.341806000
H	17.984608000	15.183961000	22.918687000
C	17.286370000	15.210040000	20.881075000
H	18.229834000	15.583180000	20.442138000
H	17.294101000	14.105118000	20.782356000
H	16.435313000	15.578662000	20.270492000
C	15.821333000	15.148694000	22.946519000
H	15.766594000	15.330516000	24.036788000
H	14.957544000	15.659087000	22.476867000
H	15.701059000	14.058292000	22.778059000
C	16.130258000	21.198500000	24.073622000
C	15.409373000	22.401078000	23.924261000
H	14.383476000	22.384623000	23.532683000
C	15.993487000	23.624833000	24.291383000
H	15.421519000	24.557542000	24.171443000
C	17.296867000	23.657112000	24.816869000
H	17.753124000	24.617246000	25.102098000
C	18.008895000	22.457319000	24.994095000
H	19.022523000	22.472692000	25.422846000
C	17.428907000	21.233917000	24.626974000
H	17.989101000	20.299746000	24.776378000
C	13.638791000	19.765107000	23.671321000
C	12.993678000	20.139906000	24.866755000
H	13.574173000	20.434780000	25.752397000
C	11.591638000	20.160264000	24.927824000
H	11.089593000	20.445677000	25.864480000
C	10.832507000	19.810900000	23.797078000
H	9.733284000	19.807560000	23.850456000
C	11.471431000	19.484490000	22.589783000
H	10.859848000	19.229096000	21.712607000
C	12.879068000	19.479289000	22.512356000
P	13.839796000	19.255016000	20.943997000
C	13.375668000	20.815568000	19.977678000
H	14.098560000	20.793445000	19.132813000
C	11.957040000	20.878068000	19.407082000
H	11.191645000	20.852118000	20.208990000
H	11.735461000	20.067811000	18.687066000
H	11.818798000	21.839602000	18.869187000
C	13.675572000	22.036843000	20.849956000
H	13.674685000	22.963279000	20.240299000
H	14.661093000	21.958209000	21.346906000
H	12.906376000	22.155639000	21.640413000
C	12.855996000	17.905907000	20.079365000
H	11.818169000	18.300741000	20.076880000
C	12.872718000	16.594499000	20.866545000

H	12.136394000	15.883662000	20.437094000
H	12.619355000	16.737758000	21.936158000
H	13.868651000	16.111363000	20.812105000
C	13.286756000	17.698453000	18.624328000
H	14.247066000	17.151030000	18.559061000
H	13.392560000	18.644289000	18.061867000
H	12.536233000	17.082692000	18.088088000
Fe	18.196020000	18.723434000	18.597784000
P	17.403682000	19.370493000	16.686594000
C	18.112293000	18.342333000	15.326761000
C	17.938643000	18.634180000	13.959648000
H	17.278418000	19.450009000	13.635480000
C	18.617265000	17.875907000	12.993887000
H	18.486402000	18.110099000	11.926891000
C	19.470674000	16.830980000	13.389068000
H	20.019496000	16.250341000	12.632632000
C	19.616747000	16.512946000	14.748428000
H	20.277259000	15.682771000	15.036479000
C	18.926158000	17.259316000	15.724864000
C	17.964744000	21.081892000	16.272672000
C	17.580703000	21.777557000	15.108397000
H	16.881238000	21.329249000	14.389285000
C	18.071406000	23.071159000	14.879602000
H	17.773507000	23.612603000	13.969379000
C	18.927486000	23.681393000	15.815617000
H	19.305212000	24.698877000	15.635312000
C	19.278684000	23.007639000	16.994510000
H	19.909811000	23.512333000	17.739857000
C	18.797690000	21.702308000	17.222596000
C	15.610442000	19.468342000	16.293233000
C	14.884618000	20.625267000	16.656225000
H	15.385125000	21.448488000	17.184085000
C	13.534093000	20.751794000	16.297390000
H	12.983373000	21.664248000	16.567589000
C	12.894336000	19.724260000	15.582302000
H	11.835835000	19.827024000	15.299996000
C	13.611032000	18.570499000	15.222135000
H	13.119071000	17.766261000	14.655072000
C	14.963822000	18.442210000	15.573289000
H	15.522406000	17.551413000	15.257819000
P	18.885394000	16.833285000	17.514855000
C	17.788397000	15.291169000	17.519855000
H	17.806796000	14.985472000	18.582485000
C	16.352891000	15.653659000	17.139149000
H	16.263944000	15.814729000	16.046557000
H	15.997066000	16.569956000	17.647879000
H	15.662106000	14.828911000	17.407490000
C	18.306683000	14.126900000	16.670420000
H	17.635889000	13.254004000	16.814937000
H	19.327389000	13.797964000	16.944894000
H	18.294488000	14.371727000	15.589668000
C	20.597903000	16.138487000	17.845801000
H	20.749457000	15.440532000	16.995098000
C	20.690639000	15.311418000	19.133552000
H	21.694405000	14.842698000	19.190667000
H	19.950249000	14.489225000	19.173249000
H	20.577437000	15.929320000	20.044375000
C	21.671627000	17.224146000	17.760805000
H	21.603341000	17.939403000	18.604239000
H	21.605851000	17.797717000	16.815310000
H	22.678052000	16.758880000	17.798990000
P	19.005661000	20.809187000	18.808480000
C	18.091244000	21.974542000	19.966095000
H	18.734709000	22.879732000	20.000735000
C	17.956697000	21.423485000	21.377761000
H	17.492747000	22.182432000	22.034304000
H	18.921788000	21.134602000	21.834703000
H	17.284608000	20.523852000	21.418907000
C	16.726862000	22.350047000	19.392917000
H	16.081462000	21.453079000	19.309166000
H	16.796392000	22.835731000	18.401963000

H	16.227667000	23.064143000	20.074973000
C	20.842755000	20.934825000	19.221648000
H	21.025246000	20.020047000	19.820201000
C	21.670062000	20.825355000	17.932035000
H	21.703526000	21.788288000	17.386621000
H	21.270090000	20.061512000	17.235788000
H	22.712679000	20.540933000	18.180133000
C	21.251514000	22.143136000	20.071118000
H	22.341501000	22.087952000	20.272365000
H	20.742525000	22.170211000	21.053691000
H	21.065930000	23.107689000	19.557108000
H	15.108709000	17.677688000	21.994285000
H	19.583179000	18.862688000	17.952609000
H	15.690304000	18.631928000	18.912946000
H	16.427123000	17.391048000	19.653340000

FP11’

N	16.381985000	17.535701000	19.590674000
Fe	15.940365000	18.401435000	21.374048000
P	15.677044000	19.561457000	23.162727000
C	16.111880000	18.575371000	24.687186000
C	15.995734000	19.024072000	26.019163000
H	15.629460000	20.038844000	26.233891000
C	16.384211000	18.192571000	27.081362000
H	16.285990000	18.545625000	28.119773000
C	16.912793000	16.915064000	26.815846000
H	17.222410000	16.262023000	27.646734000
C	17.064367000	16.480419000	25.489402000
H	17.501019000	15.488532000	25.293674000
C	16.666586000	17.308117000	24.418322000
P	16.918869000	16.878906000	22.621208000
C	18.764641000	16.428868000	22.726937000
H	18.870271000	15.655864000	23.519377000
C	19.559400000	17.673268000	23.120648000
H	19.303353000	18.019020000	24.142555000
H	20.651806000	17.473599000	23.097068000
H	19.350001000	18.504605000	22.417680000
C	19.224410000	15.847137000	21.391524000
H	18.757993000	14.864969000	21.179289000
H	18.966133000	16.548071000	20.570354000
H	20.324569000	15.699289000	21.375822000
C	16.156125000	15.126142000	22.571709000
H	16.909788000	14.455598000	23.040638000
C	15.903556000	14.656468000	21.132556000
H	16.824436000	14.625007000	20.516879000
H	15.477809000	13.630444000	21.132272000
H	15.181843000	15.326417000	20.623876000
C	14.856241000	15.055510000	23.384127000
H	15.025688000	15.173486000	24.471133000
H	14.149998000	15.850491000	23.066502000
H	14.354158000	14.077127000	23.224109000
C	16.468454000	21.170021000	23.655911000
C	15.808013000	22.407689000	23.515255000
H	14.785523000	22.439437000	23.112884000
C	16.443226000	23.602265000	23.894241000
H	15.912514000	24.560999000	23.783674000
C	17.747702000	23.574367000	24.417111000
H	18.242466000	24.509574000	24.722004000
C	18.419705000	22.344477000	24.543957000
H	19.444215000	22.313842000	24.946791000
C	17.786520000	21.153016000	24.159887000
H	18.316550000	20.193552000	24.258115000
C	13.872975000	19.999255000	23.380460000
C	13.278277000	20.498950000	24.556268000
H	13.885450000	20.691191000	25.451968000
C	11.899826000	20.765971000	24.595581000
H	11.440911000	21.146386000	25.521412000
C	11.110023000	20.543781000	23.453906000
H	10.027554000	20.743827000	23.482190000
C	11.701434000	20.063693000	22.273128000
H	11.066408000	19.886471000	21.392627000
C	13.084970000	19.791587000	22.224447000
P	13.999249000	19.161760000	20.724658000
C	13.861639000	20.622442000	19.526783000
H	14.513520000	20.264787000	18.700843000
C	12.473394000	20.972844000	18.990425000
H	11.794990000	21.303438000	19.803876000
H	11.988489000	20.137065000	18.450658000
H	12.544791000	21.822112000	18.276564000
C	14.544262000	21.837788000	20.150312000
H	14.719894000	22.624231000	19.386099000
H	15.524483000	21.559102000	20.575126000
H	13.920942000	22.279446000	20.956187000
C	12.703565000	17.967028000	20.033932000
H	11.795429000	18.593636000	19.910116000
C	12.369388000	16.838358000	21.011470000
H	11.504309000	16.250123000	20.636484000
H	12.108813000	17.217089000	22.020025000
H	13.225515000	16.145465000	21.125808000
C	13.085105000	17.418548000	18.654901000
H	13.902583000	16.672194000	18.725509000
H	13.401401000	18.208563000	17.949308000
H	12.219641000	16.895377000	18.196301000
Fe	17.660092000	18.919112000	19.000876000
P	16.839504000	19.506785000	17.058233000
C	17.974763000	18.828526000	15.720957000
C	17.805444000	19.149280000	14.359556000
H	16.956749000	19.777241000	14.047940000
C	18.712453000	18.682663000	13.395782000
H	18.579929000	18.949594000	12.335527000
C	19.787521000	17.871173000	13.795381000
H	20.506098000	17.495438000	13.050139000
C	19.944001000	17.523389000	15.148197000
H	20.777893000	16.868891000	15.424799000
C	19.050755000	18.001449000	16.131664000
C	17.035684000	21.344634000	16.776736000
C	16.442211000	22.119805000	15.759495000
H	15.781408000	21.651809000	15.015019000
C	16.679673000	23.503414000	15.692543000
H	16.195737000	24.104380000	14.906487000
C	17.537303000	24.116423000	16.623552000
H	17.724679000	25.200608000	16.573545000
C	18.175525000	23.337640000	17.604554000
H	18.883945000	23.818520000	18.296529000
C	17.923624000	21.954738000	17.690845000
C	15.213027000	19.174411000	16.209449000
C	14.125838000	20.078416000	16.260076000
H	14.252184000	21.064252000	16.726676000
C	12.879941000	19.747130000	15.703233000
H	12.057053000	20.477510000	15.744222000
C	12.681387000	18.494973000	15.095649000
H	11.702357000	18.233824000	14.665224000
C	13.751173000	17.583902000	15.039258000
H	13.617162000	16.602045000	14.558150000
C	14.999677000	17.924155000	15.581434000
H	15.830992000	17.212432000	15.500823000
P	19.035966000	17.542433000	17.951233000
C	18.494357000	15.708534000	17.777612000
H	18.332351000	15.418653000	18.839433000
C	17.161366000	15.593083000	17.037935000
H	17.299808000	15.758643000	15.949442000
H	16.417423000	16.324540000	17.401985000
H	16.730487000	14.577116000	17.165802000
C	19.512936000	14.736904000	17.171915000
H	19.102502000	13.703707000	17.193025000
H	20.475839000	14.707357000	17.717966000
H	19.722602000	14.976533000	16.109384000
C	20.831922000	17.240241000	18.597609000
H	20.724340000	16.208382000	18.989817000
C	21.149175000	18.127565000	19.806410000
H	21.918705000	17.641618000	20.442461000
H	20.251773000	18.316715000	20.422940000

H	21.555952000	19.109059000	19.501540000
C	22.002179000	17.235417000	17.608871000
H	22.080774000	18.185875000	17.041237000
H	21.946778000	16.405528000	16.879232000
H	22.956143000	17.109712000	18.165762000
P	18.850192000	20.757776000	18.780902000
C	19.248479000	21.846396000	20.279533000
H	19.940074000	22.639762000	19.927138000
C	19.918140000	21.026482000	21.383034000
H	20.141904000	21.668348000	22.259730000
H	20.858700000	20.537701000	21.067991000
H	19.216080000	20.235371000	21.716178000
C	17.975883000	22.481294000	20.836001000
H	17.290835000	21.678839000	21.166302000
H	17.443740000	23.113276000	20.100679000
H	18.214703000	23.104889000	21.720695000
C	20.516832000	20.769917000	17.771840000
H	20.769166000	19.693082000	17.766555000
C	20.375521000	21.202069000	16.306676000
H	20.225375000	22.296607000	16.215124000
H	19.538159000	20.706107000	15.786777000
H	21.305835000	20.943531000	15.755477000
C	21.677029000	21.537908000	18.414076000
H	22.607327000	21.365191000	17.830164000
H	21.887784000	21.235238000	19.457600000
H	21.493385000	22.632553000	18.410426000
H	14.859122000	17.416178000	21.877872000
H	17.236603000	19.680581000	20.441981000
H	15.587005000	17.475062000	18.946755000
H	16.747161000	16.580091000	19.654572000

BP2 (Quartet)

N	16.640336000	18.790254000	20.553869000
N	17.018615000	19.481131000	19.571971000
Fe	15.538316000	18.085172000	21.742312000
P	15.069737000	19.276548000	23.483699000
C	15.755896000	18.495449000	25.025303000
C	15.655929000	19.033363000	26.324069000
H	15.154046000	20.000222000	26.485383000
C	16.225014000	18.353796000	27.414294000
H	16.141637000	18.774835000	28.428355000
C	16.911733000	17.143128000	27.207127000
H	17.361564000	16.611637000	28.060203000
C	17.047455000	16.623567000	25.908246000
H	17.616564000	15.692998000	25.753610000
C	16.475972000	17.296802000	24.809575000
P	16.757759000	16.798220000	23.037074000
C	18.641178000	16.933586000	23.000446000
H	19.015827000	16.331535000	23.858782000
C	19.033284000	18.402330000	23.182684000
H	18.660320000	18.824857000	24.137149000
H	20.138326000	18.512418000	23.171195000
H	18.619629000	18.996633000	22.342155000
C	19.217540000	16.391601000	21.692759000
H	19.033142000	15.307545000	21.559751000
H	18.798099000	16.949882000	20.828565000
H	20.316507000	16.541842000	21.680944000
C	16.436245000	14.938196000	23.012501000
H	17.325033000	14.445641000	23.466427000
C	16.285219000	14.489211000	21.550722000
H	17.162921000	14.746298000	20.927933000
H	16.132553000	13.390314000	21.492607000
H	15.408461000	14.991586000	21.092731000
C	15.181477000	14.579565000	23.816613000
H	15.294935000	14.782179000	24.899169000
H	14.307707000	15.160808000	23.451956000
H	14.941579000	13.501477000	23.697096000
C	15.529238000	21.049104000	23.749830000
C	14.632611000	22.050915000	24.178919000
H	13.587943000	21.795334000	24.409300000
C	15.063033000	23.383309000	24.307045000
H	14.351404000	24.155276000	24.640244000
C	16.394172000	23.729723000	24.017530000
H	16.731747000	24.772317000	24.123682000
C	17.290938000	22.736199000	23.581711000
H	18.332971000	22.998960000	23.344880000
C	16.858258000	21.410794000	23.433677000
H	17.546748000	20.642142000	23.054053000
C	13.223869000	19.283799000	23.713213000
C	12.521536000	19.493352000	24.915438000
H	13.063962000	19.683461000	25.853215000
C	11.115617000	19.450425000	24.929965000
H	10.572588000	19.603891000	25.875476000
C	10.407297000	19.209836000	23.740451000
H	9.306790000	19.175640000	23.750198000
C	11.102982000	18.994514000	22.536684000
H	10.534436000	18.784815000	21.618244000
C	12.511547000	19.014216000	22.516008000
P	13.560348000	18.627343000	21.025538000
C	13.417060000	20.177848000	19.962656000
H	13.944899000	19.879170000	19.030454000
C	11.987920000	20.596095000	19.610764000
H	11.422409000	20.897091000	20.516930000
H	11.417495000	19.796196000	19.098355000
H	12.002603000	21.472254000	18.929476000
C	14.198205000	21.326972000	20.605993000
H	14.295276000	22.177491000	19.898435000
H	15.213042000	21.012124000	20.916580000
H	13.684356000	21.704545000	21.514207000
C	12.515320000	17.383048000	20.078832000
H	11.540235000	17.872713000	19.866193000
C	12.267337000	16.118232000	20.904793000
H	11.576936000	15.435630000	20.365037000
H	11.819272000	16.340589000	21.894252000
H	13.216806000	15.574762000	21.083370000
C	13.218739000	17.067886000	18.754291000
H	14.227521000	16.649371000	18.944030000
H	13.349747000	17.963079000	18.116123000
H	12.640045000	16.323767000	18.166975000
Fe	18.559511000	19.178684000	18.472913000
P	17.653404000	19.694933000	16.579825000
C	18.186483000	18.551122000	15.215134000
C	17.873673000	18.713274000	13.851119000
H	17.227653000	19.540185000	13.521575000
C	18.386016000	17.815090000	12.897717000
H	18.149920000	17.953529000	11.831112000
C	19.203355000	16.747743000	13.307292000
H	19.611770000	16.047632000	12.561810000
C	19.497784000	16.567512000	14.671563000
H	20.132788000	15.722632000	14.977196000
C	18.991973000	17.464461000	15.634885000
C	18.124162000	21.416183000	16.020304000
C	17.616286000	22.068098000	14.878600000
H	16.914868000	21.550261000	14.207068000
C	17.965107000	23.403810000	14.617803000
H	17.565303000	23.911481000	13.726026000
C	18.801965000	24.099590000	15.510087000
H	19.057639000	25.153469000	15.317991000
C	19.305902000	23.453115000	16.652002000
H	19.946166000	24.011767000	17.351728000
C	18.982983000	22.104068000	16.904754000
C	15.824382000	19.825146000	16.308462000
C	15.156405000	20.978095000	16.786121000
H	15.738793000	21.809489000	17.213230000
C	13.762254000	21.095834000	16.668506000
H	13.261722000	22.010127000	17.022327000
C	13.008871000	20.055150000	16.097969000
H	11.915848000	20.148148000	16.007448000
C	13.661725000	18.893681000	15.645996000
H	13.080440000	18.072514000	15.198354000
C	15.057942000	18.779100000	15.753409000
H	15.559747000	17.872745000	15.382339000

P	19.289921000	17.361397000	17.472400000
C	18.210185000	15.877605000	17.989827000
H	18.492242000	15.756470000	19.057419000
C	16.731025000	16.264937000	17.939992000
H	16.404085000	16.479422000	16.901808000
H	16.521063000	17.156483000	18.560630000
H	16.106917000	15.427475000	18.316565000
C	18.476281000	14.564018000	17.249989000
H	17.861483000	13.749704000	17.691170000
H	19.533770000	14.237678000	17.309721000
H	18.199421000	14.637739000	16.177910000
C	21.009404000	16.580915000	17.540164000
H	20.947373000	15.657569000	16.925111000
C	21.369007000	16.185306000	18.973708000
H	22.394975000	15.760461000	19.010270000
H	20.681649000	15.422964000	19.389951000
H	21.338586000	17.071110000	19.640378000
C	22.074904000	17.494511000	16.935531000
H	22.211261000	18.400972000	17.556406000
H	21.820647000	17.816203000	15.906966000
H	23.051510000	16.966529000	16.891882000
P	19.639415000	21.070904000	18.309092000
C	19.508955000	22.260833000	19.764752000
H	20.109479000	23.160270000	19.505553000
C	20.087995000	21.607469000	21.024235000
H	20.069952000	22.317374000	21.878569000
H	21.138453000	21.276486000	20.889412000
H	19.497338000	20.710549000	21.298024000
C	18.049765000	22.681817000	19.972604000
H	17.433871000	21.819859000	20.295600000
H	17.599700000	23.096772000	19.047960000
H	17.976909000	23.460096000	20.760410000
C	21.489187000	21.054393000	17.872075000
H	21.847506000	20.170649000	18.444137000
C	21.630387000	20.759450000	16.372459000
H	21.407071000	21.662940000	15.769092000
H	20.935108000	19.959411000	16.046853000
H	22.663903000	20.436301000	16.127945000
C	22.316782000	22.278049000	18.276001000
H	23.381198000	22.125604000	17.992890000
H	22.295520000	22.473412000	19.365981000
H	21.974605000	23.196731000	17.756510000
H	14.560264000	17.000180000	22.258571000
H	19.581661000	18.877739000	19.602640000
H	16.277728000	20.135782000	19.203940000

CP12 (Quintet)

N	16.398566000	18.786106000	20.094304000
N	17.742593000	18.926498000	19.959342000
Fe	15.469828000	18.012604000	21.517546000
P	15.243358000	19.432820000	23.250807000
C	15.831241000	18.520538000	24.754772000
C	15.679750000	19.006719000	26.074115000
H	15.224803000	19.993098000	26.246571000
C	16.150309000	18.258886000	27.165281000
H	16.025257000	18.645581000	28.189013000
C	16.796897000	17.028474000	26.949598000
H	17.166550000	16.438904000	27.802935000
C	16.981485000	16.556331000	25.635995000
H	17.519242000	15.608406000	25.475514000
C	16.497507000	17.290452000	24.535623000
P	16.817002000	16.800403000	22.769548000
C	18.717341000	16.726886000	22.885656000
H	18.928765000	16.003894000	23.704332000
C	19.274947000	18.094338000	23.288436000
H	18.920320000	18.405680000	24.290981000
H	20.385182000	18.078046000	23.309809000
H	18.971832000	18.887479000	22.572196000
C	19.354461000	16.206863000	21.595738000
H	19.014743000	15.182541000	21.344759000
H	19.132447000	16.872531000	20.736176000
H	20.458512000	16.167184000	21.705613000
C	16.400218000	14.947480000	22.708303000
H	17.291767000	14.400835000	23.087102000
C	16.162965000	14.571316000	21.238105000
H	17.025870000	14.822255000	20.592656000
H	15.959938000	13.484037000	21.132736000
H	15.291032000	15.128739000	20.835833000
C	15.184677000	14.588964000	23.569518000
H	15.391836000	14.674122000	24.653281000
H	14.328477000	15.256831000	23.338546000
H	14.863717000	13.544584000	23.367550000
C	15.891715000	21.127610000	23.625481000
C	15.166895000	22.301021000	23.309233000
H	14.126145000	22.223776000	22.962942000
C	15.752807000	23.568493000	23.453396000
H	15.165667000	24.468279000	23.209316000
C	17.074960000	23.694714000	23.919430000
H	17.531784000	24.689426000	24.037314000
C	17.806574000	22.532655000	24.234731000
H	18.837536000	22.615094000	24.614481000
C	17.228082000	21.266126000	24.076007000
H	17.814676000	20.367644000	24.319571000
C	13.411491000	19.654965000	23.467760000
C	12.813578000	20.236842000	24.609123000
H	13.441203000	20.654423000	25.409951000
C	11.415402000	20.299001000	24.729207000
H	10.963917000	20.747886000	25.627993000
C	10.595567000	19.784938000	23.709550000
H	9.499488000	19.820480000	23.807035000
C	11.181569000	19.231464000	22.552960000
H	10.531873000	18.845585000	21.753158000
C	12.581577000	19.167853000	22.420273000
P	13.451012000	18.538851000	20.905537000
C	13.205498000	19.957042000	19.685393000
H	13.769598000	19.621018000	18.788454000
C	11.755510000	20.232529000	19.282102000
H	11.132962000	20.495893000	20.162029000
H	11.281852000	19.372875000	18.767981000
H	11.711812000	21.093710000	18.583607000
C	13.888787000	21.212320000	20.235335000
H	13.905605000	22.011815000	19.465870000
H	14.933989000	21.010610000	20.542931000
H	13.336074000	21.606303000	21.112021000
C	12.330221000	17.191338000	20.235135000
H	11.329674000	17.642935000	20.063049000
C	12.191919000	16.038321000	21.231976000
H	11.472832000	15.282488000	20.850091000
H	11.828659000	16.381501000	22.221837000
H	13.166581000	15.535456000	21.389688000
C	12.905066000	16.720903000	18.892769000
H	13.944147000	16.349140000	19.020654000
H	12.942220000	17.532102000	18.138459000
H	12.294248000	15.895440000	18.469746000
Fe	18.928686000	19.124663000	18.513335000
P	17.682387000	19.677192000	16.806597000
C	18.069285000	18.555377000	15.359889000
C	17.623700000	18.758892000	14.038222000
H	16.933355000	19.581804000	13.804964000
C	18.040783000	17.900753000	13.006482000
H	17.701254000	18.080112000	11.974518000
C	18.890500000	16.818506000	13.292770000
H	19.226290000	16.149628000	12.485174000
C	19.302117000	16.580466000	14.616177000
H	19.951913000	15.719030000	14.830360000
C	18.893868000	17.441936000	15.655230000
C	18.092926000	21.413181000	16.234767000
C	17.576767000	22.027128000	15.073488000
H	16.944644000	21.461476000	14.374921000
C	17.824986000	23.387398000	14.820383000
H	17.421538000	23.855728000	13.909028000
C	18.556508000	24.154741000	15.743094000

H	18.724835000	25.227543000	15.560762000
C	19.072804000	23.550557000	16.903710000
H	19.634374000	24.160164000	17.626933000
C	18.866574000	22.174923000	17.139394000
C	15.823789000	19.815524000	16.595155000
C	15.169173000	21.037154000	16.873238000
H	15.746200000	21.894360000	17.249807000
C	13.795227000	21.184582000	16.631166000
H	13.310619000	22.153396000	16.824815000
C	13.041183000	20.104157000	16.139091000
H	11.965039000	20.222261000	15.942317000
C	13.670406000	18.868518000	15.914315000
H	13.090148000	18.012355000	15.536810000
C	15.051321000	18.727315000	16.136665000
H	15.536769000	17.767153000	15.910991000
P	19.331780000	17.219456000	17.445327000
C	18.290856000	15.725337000	17.963783000
H	18.584164000	15.594781000	19.026546000
C	16.803979000	16.083343000	17.919918000
H	16.480977000	16.305071000	16.882560000
H	16.566106000	16.950756000	18.565146000
H	16.196666000	15.222768000	18.268962000
C	18.583163000	14.432048000	17.197938000
H	18.005744000	13.593886000	17.643834000
H	19.651978000	14.140785000	17.223400000
H	18.273014000	14.512220000	16.135687000
C	21.074767000	16.518563000	17.379104000
H	21.053050000	15.646763000	16.690300000
C	21.503135000	16.045342000	18.771239000
H	22.549050000	15.672517000	18.746228000
H	20.866889000	15.225403000	19.159980000
H	21.454322000	16.889101000	19.490151000
C	22.037782000	17.570622000	16.826072000
H	22.092548000	18.441169000	17.510073000
H	21.727068000	17.937416000	15.827082000
H	23.060241000	17.147931000	16.727661000
P	19.611270000	21.224374000	18.542243000
C	19.168004000	22.232816000	20.060114000
H	19.548974000	23.262168000	19.878997000
C	19.858117000	21.643312000	21.294439000
H	19.667913000	22.280631000	22.181397000
H	20.955993000	21.546633000	21.172349000
H	19.451067000	20.636331000	21.511957000
C	17.643937000	22.271604000	20.231940000
H	17.251238000	21.249422000	20.397541000
H	17.137026000	22.694864000	19.340867000
H	17.368463000	22.891818000	21.109745000
C	21.463899000	21.484879000	18.255394000
H	21.902842000	20.686948000	18.892581000
C	21.775852000	21.164032000	16.786598000
H	21.476787000	22.002472000	16.124674000
H	21.234481000	20.260477000	16.442301000
H	22.862817000	20.990521000	16.643138000
C	22.051524000	22.834980000	18.675534000
H	23.151529000	22.829672000	18.515542000
H	21.880451000	23.066735000	19.745112000
H	21.643698000	23.671051000	18.071822000
H	14.567723000	17.134177000	22.432036000
H	20.162994000	18.852036000	19.418009000
H	15.964631000	19.070692000	19.201934000
H	18.163736000	18.689872000	20.867188000

CP12 (Triplet)

N	16.361410000	18.758781000	20.071051000
N	17.662952000	18.725543000	19.832399000
Fe	15.439646000	18.236496000	21.614943000
P	15.188533000	19.458698000	23.344904000
C	15.783510000	18.542372000	24.859383000
C	15.651928000	18.983338000	26.191147000
H	15.191300000	19.958134000	26.410129000
C	16.136277000	18.196893000	27.250300000
H	16.022113000	18.543424000	28.289428000
C	16.775592000	16.973618000	26.979449000
H	17.156199000	16.353776000	27.806406000
C	16.945915000	16.550080000	25.650006000
H	17.474336000	15.604247000	25.450069000
C	16.451540000	17.327580000	24.582920000
P	16.722865000	16.916883000	22.780661000
C	18.630115000	16.768624000	22.877632000
H	18.823301000	16.091807000	23.738483000
C	19.227814000	18.142742000	23.185726000
H	18.932974000	18.501156000	24.192296000
H	20.337672000	18.118152000	23.148143000
H	18.884098000	18.905044000	22.454001000
C	19.261365000	16.140119000	21.631860000
H	18.862688000	15.128918000	21.417595000
H	19.120676000	16.764473000	20.725841000
H	20.356753000	16.034558000	21.779078000
C	16.274777000	15.073262000	22.655031000
H	17.152805000	14.498295000	23.023942000
C	16.043767000	14.761462000	21.169479000
H	16.911707000	15.037583000	20.540503000
H	15.831729000	13.681665000	21.013500000
H	15.183254000	15.353539000	20.794110000
C	15.046960000	14.708236000	23.493960000
H	15.242118000	14.772178000	24.581976000
H	14.200840000	15.388658000	23.263418000
H	14.717559000	13.670729000	23.269794000
C	15.897133000	21.137933000	23.690578000
C	15.162487000	22.327051000	23.488221000
H	14.097976000	22.270417000	23.216933000
C	15.771731000	23.583413000	23.644732000
H	15.180445000	24.498713000	23.482558000
C	17.123730000	23.675080000	24.020815000
H	17.599204000	24.659551000	24.151083000
C	17.862939000	22.495288000	24.232192000
H	18.919869000	22.553121000	24.536346000
C	17.258837000	21.241990000	24.057662000
H	17.847376000	20.327188000	24.217572000
C	13.362588000	19.752363000	23.634326000
C	12.773763000	20.271932000	24.803448000
H	13.400431000	20.615923000	25.639194000
C	11.374751000	20.366017000	24.913464000
H	10.922962000	20.761191000	25.836793000
C	10.556323000	19.953116000	23.847965000
H	9.460392000	20.015701000	23.936328000
C	11.137527000	19.465888000	22.663189000
H	10.487768000	19.154119000	21.831803000
C	12.538387000	19.365227000	22.547565000
P	13.443394000	18.790051000	21.028602000
C	13.193198000	20.233683000	19.832814000
H	13.651296000	19.827373000	18.906608000
C	11.748382000	20.627749000	19.520108000
H	11.228856000	21.010722000	20.422846000
H	11.151063000	19.790273000	19.107005000
H	11.725039000	21.444787000	18.766428000
C	14.023743000	21.436694000	20.282270000
H	14.078856000	22.199960000	19.476756000
H	15.054040000	21.141349000	20.560342000
H	13.564893000	21.928816000	21.163503000
C	12.309056000	17.487636000	20.275863000
H	11.320586000	17.964352000	20.099740000
C	12.122945000	16.298732000	21.220494000
H	11.402335000	15.570637000	20.789886000
H	11.738150000	16.608157000	22.213792000
H	13.085801000	15.775234000	21.383295000
C	12.902711000	17.054774000	18.927578000
H	13.947241000	16.698004000	19.056657000
H	12.926526000	17.876663000	18.184508000
H	12.308922000	16.227229000	18.484024000
Fe	18.881005000	19.036489000	18.474676000
P	17.717253000	19.557656000	16.717072000

C	18.391586000	18.592634000	15.267215000
C	18.200987000	18.924829000	13.912590000
H	17.612796000	19.810850000	13.634814000
C	18.765985000	18.128534000	12.901259000
H	18.629624000	18.406164000	11.844485000
C	19.503573000	16.981351000	13.240446000
H	19.945970000	16.354792000	12.450422000
C	19.682619000	16.633644000	14.591269000
H	20.268411000	15.736407000	14.840530000
C	19.137764000	17.438142000	15.613297000
C	17.892649000	21.373761000	16.299929000
C	17.267439000	22.021879000	15.215258000
H	16.661478000	21.448066000	14.497968000
C	17.367523000	23.415386000	15.069870000
H	16.877493000	23.914048000	14.219168000
C	18.063447000	24.174418000	16.027858000
H	18.118016000	25.269953000	15.931426000
C	18.682309000	23.535450000	17.115701000
H	19.209275000	24.140820000	17.868401000
C	18.617042000	22.132953000	17.246463000
C	15.888132000	19.415988000	16.350317000
C	15.017620000	20.430799000	16.804698000
H	15.423502000	21.289458000	17.361930000
C	13.644437000	20.376729000	16.519661000
H	12.986577000	21.194197000	16.850469000
C	13.108910000	19.285084000	15.813852000
H	12.030402000	19.240657000	15.598632000
C	13.962979000	18.256113000	15.380966000
H	13.556831000	17.398296000	14.822436000
C	15.342510000	18.329031000	15.635114000
H	16.004912000	17.542839000	15.244661000
P	19.393667000	17.160093000	17.437105000
C	18.367402000	15.627040000	17.847839000
H	18.632267000	15.451659000	18.913300000
C	16.876153000	15.967122000	17.781361000
H	16.564182000	16.191533000	16.742416000
H	16.626421000	16.835725000	18.418166000
H	16.269845000	15.102555000	18.123196000
C	18.707522000	14.375411000	17.034643000
H	18.121730000	13.509318000	17.411313000
H	19.778287000	14.095330000	17.096242000
H	18.445986000	14.504617000	15.963982000
C	21.162546000	16.518021000	17.488603000
H	21.225738000	15.668158000	16.775520000
C	21.508610000	16.019630000	18.893822000
H	22.564285000	15.676066000	18.929967000
H	20.873199000	15.171852000	19.219100000
H	21.389229000	16.842571000	19.628423000
C	22.125613000	17.620222000	17.039807000
H	22.067518000	18.485666000	17.731107000
H	21.899284000	17.983400000	16.017846000
H	23.171669000	17.246613000	17.042470000
P	19.489882000	21.139753000	18.546924000
C	19.219248000	22.118302000	20.121846000
H	19.567103000	23.156735000	19.926740000
C	20.054868000	21.516268000	21.255185000
H	19.912096000	22.096299000	22.189860000
H	21.139606000	21.505480000	21.024975000
H	19.743774000	20.471160000	21.452397000
C	17.721985000	22.135863000	20.458186000
H	17.341344000	21.110869000	20.634649000
H	17.124505000	22.584345000	19.638925000
H	17.537102000	22.726979000	21.378392000
C	21.297985000	21.420258000	18.058307000
H	21.821190000	20.644884000	18.658819000
C	21.456424000	21.075503000	16.570497000
H	21.052297000	21.886206000	15.929630000
H	20.916402000	20.143418000	16.307949000
H	22.526796000	20.938667000	16.310480000
C	21.889871000	22.794243000	18.384066000
H	22.962294000	22.821911000	18.093118000

H	21.838378000	23.041609000	19.463023000
H	21.381366000	23.602809000	17.820024000
H	14.524004000	17.253871000	22.354305000
H	20.094732000	18.794636000	19.421770000
H	15.897513000	19.118731000	19.218179000
H	18.112714000	18.413083000	20.708103000

B’

N	16.938229000	18.548677000	19.959551000
Fe	16.275108000	18.814135000	21.502377000
P	15.530162000	19.738257000	23.458966000
C	15.951320000	18.606795000	24.848057000
C	15.419877000	18.711479000	26.149795000
H	14.640214000	19.450395000	26.381110000
C	15.898923000	17.873052000	27.166087000
H	15.481156000	17.952520000	28.180843000
C	16.921128000	16.943980000	26.893834000
H	17.307017000	16.298143000	27.696759000
C	17.450256000	16.837176000	25.600069000
H	18.251848000	16.110191000	25.399369000
C	16.955311000	17.657718000	24.564324000
P	17.614316000	17.578722000	22.842713000
C	19.453340000	17.863339000	23.182680000
H	19.755451000	16.939751000	23.724466000
C	19.702457000	19.061613000	24.102069000
H	19.290262000	18.896927000	25.115923000
H	20.794771000	19.225171000	24.210272000
H	19.268365000	19.998355000	23.706871000
C	20.243062000	17.936924000	21.877256000
H	20.127123000	17.012842000	21.282011000
H	19.904605000	18.779756000	21.247203000
H	21.325053000	18.068975000	22.082785000
C	17.554990000	15.732826000	22.443903000
H	18.502592000	15.320767000	22.851860000
C	17.536629000	15.523714000	20.928110000
H	18.318626000	16.107985000	20.413461000
H	17.682574000	14.449810000	20.694245000
H	16.566780000	15.844914000	20.503043000
C	16.365997000	15.032954000	23.116620000
H	16.509835000	14.890871000	24.202938000
H	15.424010000	15.602474000	22.982915000
H	16.216405000	14.032144000	22.661244000
C	16.137636000	21.364987000	24.042759000
C	15.589770000	22.546551000	23.498531000
H	14.733091000	22.492759000	22.812481000
C	16.126509000	23.796256000	23.842935000
H	15.691511000	24.711627000	23.414196000
C	17.211336000	23.876492000	24.734478000
H	17.634226000	24.856834000	25.001358000
C	17.743705000	22.703428000	25.296718000
H	18.579704000	22.761017000	26.010028000
C	17.209773000	21.450295000	24.955698000
H	17.625889000	20.540366000	25.408425000
C	13.704569000	19.886001000	23.450078000
C	12.947385000	20.414894000	24.514049000
H	13.444580000	20.885826000	25.374803000
C	11.546760000	20.352004000	24.464649000
H	10.951317000	20.762534000	25.293758000
C	10.906905000	19.753727000	23.363757000
H	9.809214000	19.681742000	23.337935000
C	11.658912000	19.268038000	22.281939000
H	11.136977000	18.823734000	21.422758000
C	13.066081000	19.359242000	22.305067000
P	14.142752000	19.011428000	20.847822000
C	13.634646000	20.377436000	19.651173000
H	14.453541000	20.352083000	18.900431000
C	12.294899000	20.153256000	18.944835000
H	11.463925000	20.019436000	19.665732000
H	12.305849000	19.295127000	18.247508000
H	12.049105000	21.052175000	18.345963000
C	13.648632000	21.728356000	20.372137000

H	13.643661000	22.556312000	19.634261000
H	14.547181000	21.853370000	21.002272000
H	12.756237000	21.844001000	21.019602000
C	13.395945000	17.468821000	20.108622000
H	12.351992000	17.758217000	19.867506000
C	13.360894000	16.303000000	21.097160000
H	12.758785000	15.471723000	20.676069000
H	12.916230000	16.582833000	22.073061000
H	14.378375000	15.908792000	21.285136000
C	14.123988000	17.104216000	18.816451000
H	15.181663000	16.853112000	19.024187000
H	14.118216000	17.925492000	18.073438000
H	13.643013000	16.224411000	18.344560000
Fe	17.888703000	18.677595000	18.616985000
P	17.023159000	19.548129000	16.676713000
C	17.665004000	18.545615000	15.258477000
C	17.422566000	18.884106000	13.912251000
H	16.757056000	19.720850000	13.657620000
C	18.025849000	18.147105000	12.882619000
H	17.835321000	18.418458000	11.833530000
C	18.877056000	17.071394000	13.191796000
H	19.359595000	16.499067000	12.385376000
C	19.100042000	16.710103000	14.529432000
H	19.748907000	15.850451000	14.751205000
C	18.485083000	17.436074000	15.570880000
C	17.798106000	21.203689000	16.363636000
C	17.493292000	21.999445000	15.239233000
H	16.756220000	21.661919000	14.497565000
C	18.107376000	23.248673000	15.077313000
H	17.866508000	23.864856000	14.198172000
C	19.015281000	23.720585000	16.043761000
H	19.486696000	24.707501000	15.923876000
C	19.311525000	22.940024000	17.169730000
H	20.006750000	23.328846000	17.927691000
C	18.710123000	21.673014000	17.328453000
C	15.275758000	19.899660000	16.216728000
C	14.719263000	21.156698000	16.545495000
H	15.315435000	21.901681000	17.091717000
C	13.417666000	21.480953000	16.137014000
H	13.007163000	22.473692000	16.375073000
C	12.645915000	20.545699000	15.425541000
H	11.624288000	20.801981000	15.107094000
C	13.183872000	19.284780000	15.121290000
H	12.588773000	18.547892000	14.561160000
C	14.495701000	18.962630000	15.506998000
H	14.917001000	17.990054000	15.219490000
P	18.533318000	16.906875000	17.338377000
C	17.309731000	15.459261000	17.324651000
H	17.034609000	15.341490000	18.392323000
C	16.063236000	15.856420000	16.530877000
H	16.267255000	15.861658000	15.441656000
H	15.694419000	16.856602000	16.817955000
H	15.247957000	15.130165000	16.719742000
C	17.889685000	14.142461000	16.797945000
H	17.093712000	13.368890000	16.824568000
H	18.735191000	13.758755000	17.398921000
H	18.217380000	14.230850000	15.741841000
C	20.221894000	16.120165000	17.516935000
H	20.256061000	15.373426000	16.696445000
C	20.403643000	15.386036000	18.846084000
H	21.384473000	14.867914000	18.848385000
H	19.624807000	14.625262000	19.040292000
H	20.408300000	16.098383000	19.693660000
C	21.331239000	17.148125000	17.297458000
H	21.358728000	17.870545000	18.135619000
H	21.210060000	17.709234000	16.350684000
H	22.315650000	16.637955000	17.263926000
P	18.997614000	20.608443000	18.803563000
C	18.364035000	21.750954000	20.151120000
H	19.058215000	22.617412000	20.103601000
C	18.446467000	21.152584000	21.541321000

H	18.125756000	21.888061000	22.302991000
H	19.460006000	20.798210000	21.799868000
H	17.766212000	20.260003000	21.658700000
C	16.946982000	22.212483000	19.827422000
H	16.274494000	21.334709000	19.775742000
H	16.880918000	22.768317000	18.873050000
H	16.580639000	22.878118000	20.633693000
C	20.879810000	20.530304000	18.958880000
H	21.038506000	19.542417000	19.440995000
C	21.503762000	20.499934000	17.554514000
H	21.554670000	21.516600000	17.118859000
H	20.929714000	19.871424000	16.846816000
H	22.536614000	20.100196000	17.606376000
C	21.524741000	21.605037000	19.841242000
H	22.620380000	21.430185000	19.874291000
H	21.161963000	21.585858000	20.886768000
H	21.373209000	22.627973000	19.441548000
H	15.599610000	17.531123000	21.759574000
H	19.066030000	18.145670000	19.367477000

C′

N	16.857563000	18.339744000	19.911612000
Fe	16.174541000	18.513013000	21.442646000
P	15.532995000	19.641738000	23.293799000
C	15.862957000	18.578543000	24.770863000
C	15.322842000	18.792484000	26.054852000
H	14.608307000	19.608865000	26.231530000
C	15.705857000	17.966387000	27.122106000
H	15.277900000	18.130673000	28.122707000
C	16.646436000	16.940366000	26.915326000
H	16.960828000	16.302709000	27.755601000
C	17.189556000	16.728010000	25.638734000
H	17.935690000	15.931235000	25.498666000
C	16.784161000	17.530588000	24.551738000
P	17.494697000	17.377946000	22.846116000
C	19.308484000	17.737780000	23.280777000
H	19.602371000	16.884625000	23.932054000
C	19.453154000	19.028653000	24.087528000
H	19.034951000	18.921028000	25.107112000
H	20.525855000	19.295220000	24.193295000
H	18.945164000	19.881634000	23.602709000
C	20.167675000	17.704410000	22.017177000
H	20.202547000	16.684896000	21.588266000
H	19.768818000	18.380568000	21.234432000
H	21.212713000	18.004308000	22.242591000
C	17.598237000	15.515170000	22.523413000
H	18.570852000	15.202194000	22.960853000
C	17.627572000	15.285152000	21.009614000
H	18.294083000	15.993653000	20.488578000
H	17.951372000	14.249221000	20.779306000
H	16.615353000	15.434142000	20.586778000
C	16.474066000	14.698036000	23.164859000
H	16.495212000	14.708981000	24.269670000
H	15.479872000	15.067912000	22.843560000
H	16.550245000	13.639651000	22.837487000
C	16.164640000	21.290198000	23.819161000
C	15.716084000	22.436501000	23.128192000
H	14.946249000	22.343044000	22.348744000
C	16.240602000	23.701102000	23.434064000
H	15.879971000	24.586771000	22.888862000
C	17.222388000	23.835733000	24.432320000
H	17.638356000	24.827142000	24.668276000
C	17.657502000	22.701143000	25.138180000
H	18.411587000	22.799695000	25.934062000
C	17.128520000	21.434729000	24.838093000
H	17.464359000	20.557983000	25.407087000
C	13.709281000	19.910118000	23.320366000
C	13.015409000	20.614541000	24.324546000
H	13.567610000	21.131736000	25.123616000
C	11.613452000	20.669098000	24.293706000
H	11.068558000	21.216180000	25.078049000

C	10.906634000	20.018856000	23.264916000
H	9.806379000	20.046548000	23.250310000
C	11.599180000	19.351058000	22.241907000
H	11.031368000	18.864184000	21.436257000
C	13.009419000	19.313971000	22.250777000
P	14.044454000	18.654843000	20.864599000
C	13.627218000	19.845046000	19.464972000
H	14.457567000	19.662959000	18.749537000
C	12.289021000	19.609097000	18.764342000
H	11.431854000	19.713912000	19.459721000
H	12.226834000	18.618461000	18.275310000
H	12.154343000	20.370093000	17.971087000
C	13.739690000	21.277209000	19.996257000
H	13.776601000	22.003734000	19.161045000
H	14.657481000	21.411032000	20.599408000
H	12.871724000	21.538270000	20.635242000
C	13.144597000	17.073866000	20.422805000
H	12.129156000	17.392988000	20.106637000
C	13.005363000	16.143802000	21.627818000
H	12.469748000	15.216585000	21.335783000
H	12.444718000	16.611936000	22.460942000
H	13.999437000	15.847264000	22.018041000
C	13.847927000	16.408615000	19.241211000
H	14.884668000	16.126483000	19.511629000
H	13.907688000	17.082980000	18.366031000
H	13.306013000	15.492808000	18.926777000
Fe	17.879358000	18.851303000	18.704029000
P	17.037286000	19.746690000	16.810614000
C	17.622647000	18.760009000	15.344094000
C	17.346904000	19.104620000	14.005969000
H	16.689185000	19.953905000	13.772634000
C	17.905714000	18.357230000	12.957730000
H	17.693357000	18.634775000	11.914011000
C	18.738391000	17.260172000	13.242710000
H	19.183715000	16.676594000	12.422452000
C	18.994268000	16.897431000	14.574990000
H	19.633593000	16.025869000	14.778923000
C	18.433883000	17.642813000	15.633529000
C	17.819722000	21.395919000	16.426201000
C	17.466916000	22.213233000	15.331793000
H	16.664280000	21.913011000	14.643367000
C	18.123180000	23.436162000	15.129050000
H	17.847231000	24.068556000	14.271428000
C	19.119765000	23.860340000	16.028052000
H	19.623245000	24.827707000	15.878615000
C	19.465104000	23.054541000	17.123338000
H	20.229862000	23.404896000	17.831830000
C	18.825987000	21.811514000	17.316690000
C	15.290889000	20.129347000	16.323550000
C	14.745728000	21.393950000	16.638313000
H	15.341034000	22.121410000	17.209170000
C	13.463306000	21.747505000	16.193471000
H	13.062769000	22.745730000	16.427427000
C	12.695387000	20.833507000	15.449599000
H	11.692035000	21.114063000	15.094796000
C	13.212058000	19.556910000	15.172752000
H	12.616139000	18.830129000	14.599640000
C	14.502378000	19.206373000	15.604160000
H	14.909275000	18.220371000	15.341771000
P	18.552992000	17.192911000	17.426202000
C	17.340424000	15.726296000	17.478076000
H	17.143033000	15.621518000	18.562698000
C	16.037310000	16.127233000	16.785041000
H	16.166049000	16.180182000	15.684826000
H	15.677056000	17.109666000	17.143701000
H	15.245701000	15.379498000	16.992389000
C	17.861173000	14.396196000	16.925756000
H	17.063151000	13.627988000	17.012420000
H	18.741665000	14.009066000	17.473615000
H	18.120466000	14.467698000	15.848906000
C	20.232670000	16.348715000	17.497636000
H	20.154233000	15.546021000	16.735379000
C	20.502006000	15.701667000	18.854928000
H	21.479924000	15.176631000	18.838172000
H	19.731214000	14.962406000	19.140434000
H	20.547947000	16.471557000	19.648413000
C	21.374477000	17.281695000	17.101909000
H	21.551157000	18.038384000	17.888296000
H	21.185566000	17.808697000	16.146686000
H	22.312255000	16.698809000	16.985958000
P	19.152812000	20.674729000	18.737877000
C	18.652734000	21.830301000	20.145133000
H	19.368907000	22.676361000	20.076272000
C	18.784107000	21.150063000	21.500043000
H	18.527082000	21.851381000	22.319571000
H	19.803211000	20.761388000	21.685568000
H	18.079041000	20.289897000	21.561708000
C	17.231907000	22.346472000	19.918199000
H	16.518541000	21.498889000	19.913625000
H	17.125017000	22.900452000	18.965099000
H	16.944164000	23.030278000	20.741142000
C	21.044774000	20.581183000	18.793449000
H	21.206958000	19.575471000	19.236685000
C	21.605740000	20.589604000	17.362213000
H	21.658288000	21.621390000	16.962802000
H	20.985846000	20.000557000	16.659543000
H	22.632794000	20.170202000	17.346336000
C	21.765887000	21.612254000	19.670305000
H	22.858798000	21.415997000	19.638696000
H	21.458921000	21.569686000	20.732757000
H	21.616425000	22.650819000	19.310809000
H	15.565084000	17.191206000	21.750624000
H	19.097240000	18.375160000	19.445085000

BP12

N	16.319112000	18.631182000	20.065385000
N	17.618372000	18.538212000	19.786137000
Fe	15.574094000	18.591437000	21.791478000
P	14.934472000	19.438939000	23.649524000
C	15.619652000	18.541970000	25.107642000
C	15.370133000	18.857796000	26.458142000
H	14.755917000	19.731574000	26.723908000
C	15.930644000	18.067789000	27.474120000
H	15.731603000	18.308373000	28.529622000
C	16.752069000	16.974367000	27.141733000
H	17.185808000	16.349625000	27.937546000
C	17.043181000	16.691728000	25.797144000
H	17.716412000	15.853841000	25.557244000
C	16.487660000	17.480479000	24.769105000
P	16.941751000	17.302203000	22.967299000
C	18.833376000	17.386963000	23.220925000
H	19.013900000	16.707989000	24.081784000
C	19.204686000	18.813004000	23.629663000
H	18.703302000	19.115639000	24.570483000
H	20.299507000	18.911755000	23.783609000
H	18.912305000	19.536084000	22.841563000
C	19.680627000	16.871173000	22.053781000
H	19.437596000	15.825175000	21.783268000
H	19.612635000	17.487878000	21.131436000
H	20.751926000	16.887896000	22.346293000
C	16.696977000	15.461785000	22.612873000
H	17.583713000	14.942447000	23.038290000
C	16.672465000	15.259336000	21.092246000
H	17.552908000	15.708914000	20.593609000
H	16.656237000	14.178511000	20.839232000
H	15.774777000	15.737871000	20.653406000
C	15.430514000	14.907989000	23.274594000
H	15.496472000	14.908429000	24.379746000
H	14.536968000	15.503629000	22.992861000
H	15.254615000	13.861904000	22.945667000
C	15.359580000	21.202075000	23.999696000
C	14.400212000	22.185490000	24.317125000
H	13.338234000	21.915575000	24.410570000

C	14.795784000	23.521072000	24.508051000
H	14.039158000	24.282106000	24.754071000
C	16.147393000	23.884795000	24.385082000
H	16.453913000	24.931379000	24.535024000
C	17.107974000	22.906332000	24.069319000
H	18.169120000	23.183999000	23.974515000
C	16.714979000	21.574744000	23.871753000
H	17.462232000	20.813734000	23.606710000
C	13.090569000	19.415965000	23.790390000
C	12.325700000	19.508176000	24.968181000
H	12.811464000	19.580730000	25.951211000
C	10.922264000	19.500064000	24.892333000
H	10.325448000	19.561766000	25.815105000
C	10.282300000	19.409527000	23.643827000
H	9.183069000	19.400490000	23.587537000
C	11.042057000	19.315138000	22.465310000
H	10.528628000	19.219510000	21.497401000
C	12.449375000	19.312353000	22.532714000
P	13.578373000	19.088433000	21.080718000
C	13.389843000	20.652052000	20.055770000
H	13.895611000	20.358182000	19.111700000
C	11.957489000	21.078230000	19.730828000
H	11.418148000	21.405470000	20.643217000
H	11.366774000	20.273473000	19.249419000
H	11.965248000	21.942414000	19.032951000
C	14.200891000	21.781304000	20.698622000
H	14.229617000	22.669390000	20.032735000
H	15.244743000	21.468865000	20.899943000
H	13.761118000	22.099406000	21.665408000
C	12.687497000	17.775917000	20.068604000
H	11.712600000	18.228228000	19.783250000
C	12.427241000	16.510483000	20.891527000
H	11.820280000	15.793489000	20.299844000
H	11.882395000	16.714184000	21.834650000
H	13.379935000	16.007255000	21.152477000
C	13.482826000	17.455648000	18.799331000
H	14.454558000	16.990466000	19.055740000
H	13.679279000	18.350555000	18.180353000
H	12.923089000	16.738583000	18.164462000
Fe	18.781571000	18.823956000	18.441682000
P	17.587167000	19.422216000	16.685619000
C	18.247315000	18.480945000	15.216737000
C	18.012870000	18.839040000	13.875587000
H	17.390028000	19.711630000	13.633165000
C	18.577778000	18.085212000	12.833752000
H	18.402197000	18.380344000	11.787982000
C	19.367578000	16.959471000	13.126323000
H	19.811410000	16.368766000	12.310440000
C	19.593838000	16.585712000	14.461558000
H	20.215966000	15.704365000	14.676835000
C	19.040295000	17.344299000	15.513351000
C	17.857787000	21.224097000	16.280034000
C	17.192499000	21.923586000	15.252178000
H	16.480160000	21.405254000	14.593518000
C	17.398283000	23.302730000	15.090657000
H	16.875802000	23.842110000	14.285815000
C	18.246685000	23.997866000	15.971575000
H	18.387338000	25.083991000	15.861195000
C	18.909421000	23.308306000	16.999618000
H	19.557655000	23.863872000	17.693132000
C	18.730102000	21.917405000	17.147178000
C	15.756617000	19.364660000	16.339979000
C	14.936369000	20.399415000	16.844194000
H	15.380226000	21.211290000	17.441904000
C	13.564868000	20.433064000	16.543861000
H	12.943995000	21.261082000	16.916834000
C	12.985214000	19.415924000	15.765655000
H	11.909697000	19.442759000	15.534193000
C	13.787955000	18.367641000	15.285561000
H	13.344841000	17.565511000	14.675578000
C	15.164860000	18.346645000	15.563842000
H	15.787593000	17.544247000	15.142701000
P	19.302392000	16.988537000	17.309775000
C	18.204911000	15.500734000	17.681580000
H	18.445153000	15.295652000	18.747220000
C	16.728445000	15.887413000	17.589645000
H	16.434973000	16.104650000	16.543832000
H	16.499068000	16.775193000	18.205531000
H	16.091816000	15.049272000	17.939803000
C	18.523069000	14.255955000	16.848202000
H	17.876529000	13.414470000	17.175855000
H	19.573330000	13.920071000	16.954861000
H	18.319400000	14.424444000	15.770656000
C	21.030325000	16.269126000	17.398926000
H	21.052029000	15.415165000	16.688178000
C	21.304025000	15.750030000	18.814269000
H	22.334650000	15.342920000	18.877414000
H	20.610040000	14.941077000	19.117662000
H	21.215247000	16.575777000	19.550005000
C	22.074020000	17.299097000	16.963803000
H	22.087932000	18.161914000	17.659815000
H	21.885397000	17.680286000	15.941349000
H	23.085784000	16.842506000	16.971689000
P	19.626139000	20.865480000	18.372509000
C	19.514930000	21.828773000	19.969780000
H	19.901225000	22.849552000	19.757472000
C	20.390589000	21.172566000	21.040662000
H	20.285216000	21.706229000	22.008100000
H	21.465504000	21.181415000	20.770163000
H	20.098374000	20.114857000	21.192080000
C	18.040437000	21.924752000	20.384138000
H	17.610406000	20.922538000	20.580858000
H	17.422375000	22.400626000	19.596087000
H	17.934114000	22.532769000	21.305112000
C	21.407079000	20.983204000	17.767835000
H	21.906346000	20.166394000	18.332514000
C	21.431799000	20.644551000	16.270747000
H	21.020999000	21.480984000	15.669138000
H	20.831806000	19.742748000	16.035881000
H	22.472096000	20.461965000	15.931506000
C	22.125766000	22.303033000	18.061977000
H	23.182924000	22.229126000	17.728995000
H	22.141120000	22.557512000	19.140029000
H	21.673519000	23.152103000	17.510952000
H	14.746559000	17.424040000	22.188802000
H	19.988542000	18.515030000	19.349782000
H	15.835664000	18.921882000	19.199310000
H	18.077960000	18.205054000	20.653994000

BP122

N	16.988469000	18.887570000	20.486529000
N	16.701693000	19.706217000	19.353107000
Fe	15.805083000	18.558471000	21.948229000
P	14.959851000	19.292087000	23.765414000
C	15.645758000	18.465401000	25.255612000
C	15.294915000	18.764156000	26.586934000
H	14.576394000	19.567096000	26.812690000
C	15.889544000	18.044815000	27.635742000
H	15.616169000	18.270099000	28.677746000
C	16.840416000	17.044523000	27.356889000
H	17.302448000	16.481482000	28.182045000
C	17.222143000	16.777248000	26.031737000
H	17.991009000	16.014180000	25.833120000
C	16.630069000	17.492457000	24.971648000
P	17.159185000	17.349409000	23.199946000
C	19.007420000	17.657369000	23.417620000
H	19.331178000	16.984670000	24.240767000
C	19.231763000	19.106426000	23.853576000
H	18.676772000	19.357985000	24.778950000
H	20.308475000	19.293322000	24.045805000
H	18.913947000	19.816795000	23.063062000
C	19.793636000	17.294431000	22.155564000

H	19.785557000	16.205558000	21.962367000
H	19.388954000	17.793395000	21.251057000
H	20.855017000	17.600754000	22.260022000
C	17.064249000	15.510681000	22.800026000
H	17.974207000	15.048889000	23.241552000
C	17.091876000	15.330958000	21.274322000
H	17.910036000	15.901429000	20.797874000
H	17.219515000	14.260652000	21.010423000
H	16.143703000	15.685985000	20.824252000
C	15.818841000	14.863711000	23.415607000
H	15.844685000	14.862863000	24.522274000
H	14.894567000	15.392801000	23.099836000
H	15.732890000	13.809639000	23.077765000
C	15.193846000	21.093027000	24.062406000
C	14.142950000	21.948357000	24.450540000
H	13.137866000	21.544953000	24.639972000
C	14.374653000	23.328358000	24.582448000
H	13.549800000	23.990916000	24.885333000
C	15.649393000	23.861235000	24.325885000
H	15.825503000	24.943070000	24.426509000
C	16.701519000	23.009051000	23.943791000
H	17.703504000	23.421174000	23.749891000
C	16.473955000	21.632079000	23.810614000
H	17.291234000	20.962800000	23.504552000
C	13.130032000	19.092111000	23.765213000
C	12.286018000	18.997050000	24.885462000
H	12.698270000	19.001851000	25.903909000
C	10.897294000	18.883027000	24.699354000
H	10.234757000	18.798707000	25.573605000
C	10.355907000	18.871912000	23.402367000
H	9.268350000	18.779142000	23.260000000
C	11.198983000	18.958378000	22.281314000
H	10.759932000	18.913566000	21.274475000
C	12.592779000	19.061878000	22.455760000
P	13.835892000	19.035406000	21.080786000
C	13.569027000	20.646230000	20.129799000
H	14.063127000	20.426713000	19.157257000
C	12.113887000	21.015188000	19.834929000
H	11.558229000	21.233949000	20.768797000
H	11.572797000	20.220687000	19.283611000
H	12.072932000	21.933191000	19.211758000
C	14.319105000	21.792271000	20.816951000
H	14.381717000	22.677487000	20.151451000
H	15.347983000	21.506914000	21.118188000
H	13.802695000	22.105492000	21.746131000
C	13.124552000	17.738033000	19.922094000
H	12.147362000	18.132109000	19.568307000
C	12.897420000	16.405401000	20.642442000
H	12.400936000	15.685524000	19.959738000
H	12.260976000	16.513780000	21.541892000
H	13.860347000	15.958876000	20.962775000
C	14.057060000	17.564852000	18.722442000
H	15.057070000	17.218811000	19.056079000
H	14.170137000	18.493494000	18.128119000
H	13.656537000	16.802108000	18.026626000
Fe	18.219427000	18.902960000	18.385029000
P	17.515181000	19.556672000	16.400648000
C	18.215160000	18.510055000	15.066158000
C	18.147139000	18.820300000	13.696029000
H	17.652652000	19.740365000	13.351737000
C	18.726152000	17.946934000	12.761369000
H	18.686996000	18.190734000	11.689239000
C	19.358857000	16.767590000	13.193042000
H	19.814232000	16.087661000	12.457435000
C	19.420404000	16.451524000	14.560849000
H	19.928499000	15.529808000	14.879550000
C	18.849784000	17.323686000	15.508240000
C	17.951916000	21.330747000	16.142900000
C	17.431235000	22.117165000	15.097274000
H	16.776567000	21.672652000	14.333030000
C	17.716015000	23.490930000	15.055366000
H	17.310763000	24.107203000	14.239088000
C	18.492016000	24.083425000	16.067962000
H	18.691309000	25.165315000	16.045961000
C	19.013501000	23.302735000	17.112816000
H	19.613718000	23.784801000	17.897820000
C	18.754441000	21.917547000	17.148680000
C	15.705802000	19.625077000	16.064814000
C	14.951118000	20.653689000	16.676288000
H	15.453337000	21.442062000	17.259045000
C	13.558796000	20.702690000	16.507964000
H	12.980367000	21.512594000	16.975440000
C	12.906557000	19.723146000	15.738612000
H	11.813912000	19.758539000	15.614751000
C	13.655151000	18.711935000	15.113315000
H	13.152694000	17.954931000	14.492846000
C	15.050002000	18.663432000	15.268798000
H	15.625704000	17.878465000	14.759804000
P	18.869052000	17.030614000	17.334726000
C	17.603722000	15.642198000	17.600205000
H	17.381724000	15.712932000	18.686408000
C	16.331865000	15.961972000	16.813936000
H	16.491498000	15.826026000	15.725387000
H	15.987599000	16.997557000	16.982843000
H	15.512864000	15.281234000	17.120682000
C	18.106656000	14.231457000	17.279904000
H	17.281538000	13.510723000	17.459844000
H	18.958653000	13.911187000	17.908645000
H	18.396422000	14.130544000	16.213771000
C	20.508834000	16.184943000	17.642037000
H	20.438442000	15.243607000	17.058082000
C	20.641386000	15.838251000	19.127003000
H	21.531675000	15.197092000	19.289793000
H	19.763227000	15.292372000	19.524354000
H	20.776992000	16.757446000	19.729817000
C	21.714998000	16.977357000	17.140660000
H	21.834687000	17.922934000	17.703428000
H	21.652801000	17.215096000	16.061822000
H	22.637785000	16.381930000	17.299352000
P	19.522174000	20.723342000	18.326396000
C	19.770349000	21.674854000	19.917232000
H	20.325744000	22.588711000	19.620144000
C	20.640249000	20.857799000	20.877355000
H	20.801816000	21.419588000	21.819340000
H	21.636399000	20.619568000	20.455257000
H	20.156515000	19.893887000	21.134989000
C	18.449105000	22.109677000	20.560361000
H	17.926527000	21.257324000	21.042567000
H	17.765031000	22.602916000	19.839383000
H	18.646972000	22.841735000	21.368256000
C	21.259377000	20.588827000	17.606556000
H	21.711598000	19.758667000	18.191282000
C	21.180677000	20.181244000	16.131377000
H	20.796633000	21.011659000	15.505200000
H	20.526028000	19.307027000	15.967389000
H	22.191561000	19.919922000	15.758377000
C	22.121442000	21.843544000	17.779756000
H	23.118758000	21.654646000	17.331133000
H	22.290338000	22.120919000	18.837895000
H	21.691082000	22.717857000	17.250568000
H	14.990566000	17.352774000	22.237776000
H	19.642935000	18.591008000	18.887046000
H	16.679900000	20.720924000	19.557744000
H	15.780002000	19.432661000	18.977135000
H	17.920335000	19.196400000	20.820609000

CP22

N	16.526419000	18.564440000	20.557030000
N	16.875678000	18.525619000	19.327884000
Fe	15.627892000	18.408395000	21.974996000
P	15.068672000	19.337113000	23.835691000
C	15.629336000	18.347357000	25.301501000

C	15.437741000	18.684706000	26.655833000
H	14.919981000	19.615123000	26.934556000
C	15.935847000	17.844169000	27.665425000
H	15.779034000	18.106356000	28.723071000
C	16.644474000	16.676997000	27.325787000
H	17.037330000	16.021036000	28.117912000
C	16.872997000	16.357932000	25.976595000
H	17.456572000	15.458626000	25.723511000
C	16.367634000	17.189927000	24.957845000
P	16.739070000	16.960197000	23.151240000
C	18.619627000	17.074664000	23.200597000
H	18.937042000	16.341451000	23.975880000
C	19.025919000	18.485700000	23.634088000
H	18.576554000	18.780192000	24.603573000
H	20.129412000	18.555356000	23.735521000
H	18.709955000	19.221640000	22.867446000
C	19.256518000	16.709253000	21.857945000
H	19.072257000	15.654791000	21.575257000
H	18.900324000	17.370717000	21.038854000
H	20.356968000	16.836629000	21.927217000
C	16.406535000	15.139198000	22.808451000
H	17.275888000	14.576628000	23.215626000
C	16.317276000	14.914682000	21.290384000
H	17.181278000	15.332427000	20.738933000
H	16.257210000	13.829369000	21.063865000
H	15.411298000	15.406481000	20.882937000
C	15.120856000	14.672610000	23.501149000
H	15.202228000	14.682515000	24.605301000
H	14.267273000	15.326033000	23.220615000
H	14.868763000	13.637468000	23.187814000
C	15.643121000	21.048762000	24.252681000
C	14.932439000	21.937430000	25.085568000
H	13.972274000	21.637629000	25.529994000
C	15.433477000	23.226892000	25.335926000
H	14.867953000	23.914808000	25.983619000
C	16.645188000	23.640017000	24.755643000
H	17.034971000	24.651019000	24.950487000
C	17.351522000	22.761183000	23.914753000
H	18.294319000	23.083855000	23.447013000
C	16.849439000	21.476267000	23.658780000
H	17.373762000	20.797187000	22.968082000
C	13.218292000	19.466259000	23.949045000
C	12.441273000	19.587653000	25.116472000
H	12.915991000	19.597653000	26.108149000
C	11.040290000	19.673146000	25.024689000
H	10.437081000	19.754682000	25.941958000
C	10.412461000	19.645456000	23.767994000
H	9.315742000	19.708985000	23.697310000
C	11.181786000	19.514304000	22.597891000
H	10.676434000	19.462927000	21.622084000
C	12.584180000	19.412069000	22.681569000
P	13.712994000	19.116865000	21.239196000
C	13.671961000	20.731764000	20.265907000
H	14.321717000	20.501927000	19.391267000
C	12.293767000	21.173977000	19.766764000
H	11.618838000	21.423096000	20.611709000
H	11.792807000	20.412694000	19.136789000
H	12.393531000	22.094533000	19.152831000
C	14.347110000	21.825741000	21.096985000
H	14.440001000	22.759212000	20.503904000
H	15.358600000	21.521649000	21.420888000
H	13.757294000	22.064514000	22.007060000
C	12.766765000	17.918792000	20.139765000
H	11.895325000	18.482559000	19.744069000
C	12.262128000	16.696114000	20.909650000
H	11.682157000	16.034313000	20.232454000
H	11.607620000	16.965365000	21.761715000
H	13.112717000	16.107236000	21.309264000
C	13.671202000	17.500857000	18.971646000
H	14.527295000	16.909303000	19.357270000
H	14.063827000	18.364805000	18.401182000
H	13.113283000	16.863912000	18.255440000
Fe	18.578443000	19.061864000	18.159557000
P	17.707771000	19.481350000	16.275741000
C	18.413655000	18.400639000	14.936710000
C	18.259250000	18.582472000	13.549470000
H	17.647466000	19.410661000	13.160483000
C	18.894412000	17.705624000	12.648375000
H	18.787619000	17.860506000	11.563117000
C	19.664731000	16.636982000	13.135967000
H	20.164798000	15.952073000	12.432975000
C	19.800493000	16.437880000	14.524913000
H	20.406277000	15.594252000	14.889874000
C	19.184998000	17.319292000	15.435327000
C	17.976409000	21.267631000	15.787149000
C	17.438193000	21.892482000	14.645931000
H	16.850692000	21.309032000	13.919426000
C	17.611680000	23.274576000	14.450747000
H	17.189293000	23.762131000	13.557892000
C	18.299029000	24.036685000	15.412531000
H	18.413998000	25.123536000	15.274668000
C	18.833411000	23.414499000	16.556537000
H	19.352433000	24.026283000	17.310349000
C	18.690245000	22.025125000	16.745743000
C	15.874550000	19.393838000	15.951166000
C	15.054836000	20.360830000	16.583240000
H	15.526101000	21.188593000	17.138658000
C	13.656786000	20.282970000	16.499652000
H	13.036441000	21.053056000	16.981956000
C	13.045954000	19.221907000	15.804011000
H	11.948455000	19.155872000	15.747866000
C	13.849290000	18.248642000	15.185188000
H	13.382300000	17.415436000	14.636165000
C	15.251341000	18.337286000	15.252680000
H	15.870424000	17.578729000	14.750938000
P	19.295356000	17.228176000	17.304658000
C	18.232647000	15.666968000	17.666471000
H	18.136224000	15.724364000	18.774408000
C	16.839361000	15.845104000	17.056162000
H	16.863098000	15.675536000	15.959678000
H	16.441972000	16.869772000	17.198931000
H	16.111465000	15.128090000	17.491705000
C	18.811890000	14.300538000	17.289758000
H	18.105924000	13.489551000	17.576784000
H	19.773926000	14.082508000	17.793647000
H	18.973743000	14.213531000	16.194772000
C	21.036569000	16.534262000	17.558628000
H	21.106648000	15.595361000	16.968766000
C	21.245336000	16.220539000	19.041893000
H	22.276059000	15.847968000	19.227612000
H	20.539893000	15.454582000	19.421817000
H	21.088787000	17.142036000	19.639202000
C	22.089922000	17.520587000	17.055153000
H	22.034963000	18.465953000	17.629906000
H	21.955205000	17.766995000	15.983365000
H	23.111451000	17.100143000	17.180371000
P	19.412865000	21.018702000	18.151086000
C	19.128755000	22.142827000	19.646198000
H	19.563101000	23.141415000	19.423020000
C	19.817456000	21.538433000	20.874513000
H	19.658324000	22.174583000	21.771546000
H	20.912705000	21.422757000	20.739568000
H	19.400538000	20.529010000	21.076273000
C	17.623288000	22.282103000	19.886081000
H	17.181837000	21.290822000	20.122207000
H	17.093054000	22.688146000	19.000158000
H	17.417301000	22.954655000	20.745322000
C	21.271095000	21.226599000	17.781094000
H	21.714281000	20.438787000	18.428881000
C	21.516324000	20.848022000	16.314434000
H	21.212013000	21.672528000	15.636597000
H	20.933960000	19.947547000	16.032295000

H	22.591335000	20.639144000	16.128986000
C	21.915589000	22.573534000	18.121123000
H	23.003926000	22.544919000	17.892537000
H	21.818162000	22.843485000	19.191391000
H	21.483808000	23.398994000	17.518052000
H	14.661298000	17.268864000	22.167069000
H	19.635816000	18.893056000	19.337859000
H	16.959430000	19.574966000	18.799983000
H	16.324614000	17.864367000	18.748532000

CP122

N	16.369156000	18.980257000	20.400392000
N	17.764687000	18.641639000	20.147212000
Fe	15.460721000	18.616738000	21.934038000
P	14.736210000	19.532226000	23.702434000
C	15.353214000	18.711850000	25.238014000
C	15.038611000	19.082471000	26.560241000
H	14.408785000	19.963513000	26.758115000
C	15.549771000	18.336274000	27.634910000
H	15.297725000	18.618335000	28.668728000
C	16.388879000	17.233895000	27.388371000
H	16.783770000	16.643607000	28.229609000
C	16.746584000	16.897732000	26.071468000
H	17.432023000	16.053013000	25.897788000
C	16.238469000	17.639749000	24.986308000
P	16.764041000	17.387528000	23.209919000
C	18.644374000	17.541062000	23.537554000
H	18.800237000	16.980487000	24.484070000
C	18.951253000	19.017272000	23.796773000
H	18.377083000	19.405251000	24.662241000
H	20.029152000	19.176473000	24.007041000
H	18.681547000	19.642277000	22.920442000
C	19.566340000	16.902519000	22.493209000
H	19.400791000	15.811027000	22.400641000
H	19.485659000	17.340343000	21.476082000
H	20.625203000	17.039913000	22.799548000
C	16.587523000	15.522543000	22.945559000
H	17.443035000	15.042631000	23.469795000
C	16.683558000	15.242930000	21.439104000
H	17.632101000	15.610889000	20.998121000
H	16.630363000	14.153179000	21.232376000
H	15.847680000	15.741413000	20.906795000
C	15.279454000	14.978073000	23.527161000
H	15.238522000	15.068704000	24.630042000
H	14.405461000	15.523081000	23.113331000
H	15.161905000	13.903650000	23.271010000
C	15.095961000	21.325094000	23.998538000
C	14.143883000	22.251660000	24.470239000
H	13.123564000	21.924056000	24.717578000
C	14.487129000	23.608119000	24.612302000
H	13.734733000	24.324177000	24.978013000
C	15.780278000	24.049612000	24.285483000
H	16.046094000	25.112260000	24.395749000
C	16.732726000	23.128880000	23.810690000
H	17.746763000	23.470814000	23.551614000
C	16.388962000	21.777787000	23.661170000
H	17.116762000	21.056898000	23.262050000
C	12.881831000	19.481596000	23.758929000
C	12.052277000	19.567597000	24.892681000
H	12.483764000	19.654406000	25.899931000
C	10.654425000	19.527676000	24.743398000
H	10.008601000	19.582587000	25.633184000
C	10.083364000	19.414759000	23.464116000
H	8.988993000	19.382852000	23.349403000
C	10.907855000	19.322366000	22.328910000
H	10.448352000	19.205708000	21.336514000
C	12.309191000	19.343043000	22.470774000
P	13.526478000	19.095641000	21.093268000
C	13.374241000	20.629899000	20.016519000
H	13.873820000	20.295912000	19.082234000
C	11.949703000	21.069995000	19.673577000
H	11.418632000	21.447527000	20.571385000
H	11.340385000	20.256644000	19.230987000
H	11.972287000	21.901403000	18.936953000
C	14.201524000	21.769003000	20.622763000
H	14.223312000	22.641238000	19.935138000
H	15.243547000	21.447940000	20.817716000
H	13.775600000	22.113020000	21.587843000
C	12.754579000	17.740959000	20.036365000
H	11.866727000	18.189409000	19.538619000
C	12.307889000	16.543655000	20.880425000
H	11.885097000	15.752134000	20.226355000
H	11.535788000	16.811252000	21.627794000
H	13.169110000	16.107574000	21.427271000
C	13.767625000	17.315585000	18.966696000
H	14.659677000	16.853318000	19.435234000
H	14.123781000	18.165974000	18.358432000
H	13.311139000	16.579513000	18.273978000
Fe	18.665396000	18.462438000	18.380350000
P	17.448855000	19.278198000	16.716993000
C	18.093032000	18.572649000	15.104269000
C	17.597322000	18.951769000	13.842177000
H	16.782252000	19.686143000	13.763870000
C	18.134843000	18.387161000	12.674347000
H	17.751969000	18.698322000	11.690437000
C	19.153728000	17.421372000	12.761864000
H	19.565423000	16.968609000	11.847001000
C	19.645223000	17.028117000	14.017167000
H	20.433757000	16.263222000	14.072142000
C	19.128068000	17.611462000	15.192127000
C	17.757768000	21.099387000	16.549194000
C	17.105711000	21.956650000	15.640538000
H	16.348407000	21.566161000	14.945153000
C	17.401866000	23.329131000	15.636017000
H	16.885436000	23.996719000	14.929595000
C	18.343720000	23.851435000	16.540853000
H	18.561985000	24.930238000	16.549316000
C	19.012890000	22.997437000	17.432762000
H	19.754157000	23.419012000	18.127739000
C	18.729112000	21.616671000	17.432956000
C	15.629922000	19.172436000	16.368172000
C	14.749889000	20.196047000	16.780885000
H	15.143681000	21.086149000	17.294569000
C	13.374008000	20.104941000	16.510112000
H	12.704040000	20.923038000	16.813049000
C	12.853978000	18.976081000	15.855759000
H	11.775550000	18.905786000	15.648230000
C	13.719704000	17.940395000	15.463482000
H	13.322745000	17.051406000	14.949899000
C	15.097483000	18.040536000	15.710374000
H	15.765326000	17.237860000	15.363260000
P	19.677279000	17.228103000	16.911243000
C	18.757629000	15.633285000	17.343535000
H	19.369976000	15.213970000	18.169807000
C	17.391465000	16.028807000	17.918170000
H	16.681966000	16.359299000	17.137133000
H	17.447387000	16.844997000	18.683859000
H	16.921279000	15.179366000	18.454827000
C	18.636357000	14.602077000	16.224247000
H	18.126308000	13.690268000	16.601915000
H	19.624310000	14.284776000	15.833407000
H	18.040130000	14.994115000	15.375290000
C	21.441223000	16.582902000	16.789412000
H	21.309371000	15.614764000	16.256975000
C	21.983451000	16.293747000	18.195157000
H	22.971846000	15.794199000	18.121438000
H	21.319245000	15.631223000	18.784683000
H	22.114293000	17.231169000	18.773112000
C	22.424288000	17.433952000	15.977869000
H	22.768018000	18.316213000	16.550234000
H	22.005478000	17.786283000	15.016723000
H	23.325149000	16.828472000	15.746874000

P	19.672669000	20.354292000	18.405259000
C	19.938726000	21.214202000	20.053770000
H	20.539095000	22.109245000	19.787663000
C	20.794863000	20.338319000	20.974518000
H	21.040263000	20.880104000	21.910723000
H	21.749702000	20.044214000	20.494927000
H	20.286755000	19.394915000	21.248224000
C	18.641061000	21.730974000	20.693359000
H	17.900726000	20.947922000	20.954566000
H	18.113624000	22.437554000	20.023231000
H	18.885664000	22.279210000	21.625875000
C	21.365279000	20.453331000	17.556893000
H	21.815735000	19.478953000	17.839968000
C	21.181537000	20.503082000	16.034627000
H	20.760828000	21.477580000	15.716009000
H	20.509513000	19.711529000	15.653949000
H	22.160265000	20.382536000	15.528330000
C	22.291896000	21.584152000	18.017022000
H	23.249282000	21.516707000	17.458157000
H	22.543604000	21.538112000	19.093591000
H	21.863039000	22.583451000	17.798394000
H	14.595270000	17.468562000	22.388222000
H	19.818121000	17.949219000	19.260228000
H	16.027990000	19.346095000	19.500871000
H	17.900473000	17.703745000	20.559312000
H	18.337382000	19.235727000	20.766876000

CP1122

N	16.171561000	18.544781000	20.052328000
N	17.471270000	17.915805000	19.707437000
Fe	15.457759000	18.566375000	21.917894000
P	14.760068000	19.337076000	23.783527000
C	15.533229000	18.549001000	25.244757000
C	15.272643000	18.878242000	26.589979000
H	14.579302000	19.693746000	26.844518000
C	15.925491000	18.174031000	27.612935000
H	15.722753000	18.422824000	28.665503000
C	16.842727000	17.155787000	27.292165000
H	17.347243000	16.597521000	28.095233000
C	17.136248000	16.861357000	25.951098000
H	17.873748000	16.076484000	25.723057000
C	16.490501000	17.566103000	24.914891000
P	16.897170000	17.346096000	23.107273000
C	18.785479000	17.535879000	23.280266000
H	19.018364000	16.951655000	24.194728000
C	19.121399000	19.006039000	23.537820000
H	18.585993000	19.408643000	24.420573000
H	20.208212000	19.131718000	23.721994000
H	18.864256000	19.634559000	22.659901000
C	19.625692000	16.953189000	22.143188000
H	19.380722000	15.898413000	21.913114000
H	19.576405000	17.550650000	21.206418000
H	20.695914000	16.974167000	22.436517000
C	16.676383000	15.480374000	22.865331000
H	17.557265000	15.006014000	23.350585000
C	16.665979000	15.111282000	21.375701000
H	17.620043000	15.352336000	20.866851000
H	16.512724000	14.019721000	21.252500000
H	15.832410000	15.622278000	20.848819000
C	15.398418000	14.985135000	23.553694000
H	15.431629000	15.114545000	24.651925000
H	14.504264000	15.525753000	23.178760000
H	15.251477000	13.905302000	23.344062000
C	15.101084000	21.135408000	23.963955000
C	14.109047000	22.082448000	24.289465000
H	13.084625000	21.758133000	24.524110000
C	14.425253000	23.451843000	24.299246000
H	13.647062000	24.187001000	24.555021000
C	15.723781000	23.882967000	23.977697000
H	15.964154000	24.956962000	23.979164000
C	16.717355000	22.939956000	23.656539000

H	17.737386000	23.273703000	23.412091000
C	16.407052000	21.572609000	23.650197000
H	17.180252000	20.830357000	23.400969000
C	12.928126000	19.232997000	23.917661000
C	12.157196000	19.243876000	25.093516000
H	12.636149000	19.262411000	26.082160000
C	10.754790000	19.221531000	25.003668000
H	10.149928000	19.220185000	25.922892000
C	10.125777000	19.197182000	23.746793000
H	9.027496000	19.178205000	23.681143000
C	10.893178000	19.178469000	22.569731000
H	10.386113000	19.128594000	21.595443000
C	12.299183000	19.190174000	22.649659000
P	13.441991000	19.041012000	21.203086000
C	13.248208000	20.601325000	20.173507000
H	13.605222000	20.246523000	19.182938000
C	11.817073000	21.116238000	20.014957000
H	11.406317000	21.470974000	20.981722000
H	11.130308000	20.349736000	19.604107000
H	11.798886000	21.979266000	19.316314000
C	14.203818000	21.695532000	20.661320000
H	14.223463000	22.543145000	19.945797000
H	15.247377000	21.332162000	20.777421000
H	13.897537000	22.095589000	21.647581000
C	12.594300000	17.734429000	20.141430000
H	11.633665000	18.201464000	19.832604000
C	12.298658000	16.458782000	20.935708000
H	11.737083000	15.742147000	20.301523000
H	11.694200000	16.650126000	21.843246000
H	13.237122000	15.959315000	21.252145000
C	13.406673000	17.417166000	18.878699000
H	14.285728000	16.782336000	19.122404000
H	13.731126000	18.320323000	18.321464000
H	12.797130000	16.823830000	18.168259000
Fe	18.681803000	18.971663000	18.548129000
P	17.488182000	19.590294000	16.778704000
C	18.239878000	18.698667000	15.328737000
C	18.022074000	19.080995000	13.992797000
H	17.318985000	19.893071000	13.753889000
C	18.717441000	18.434910000	12.956854000
H	18.553320000	18.744287000	11.913633000
C	19.629219000	17.405696000	13.250694000
H	20.179867000	16.908266000	12.438159000
C	19.845184000	17.010691000	14.581984000
H	20.562737000	16.206276000	14.799978000
C	19.153689000	17.657432000	15.624776000
C	17.843484000	21.365622000	16.369190000
C	17.069421000	22.171686000	15.511699000
H	16.190937000	21.756439000	14.996230000
C	17.405132000	23.523075000	15.332122000
H	16.798848000	24.151682000	14.662444000
C	18.497321000	24.077168000	16.023180000
H	18.740033000	25.144370000	15.908040000
C	19.282057000	23.271790000	16.864721000
H	20.124854000	23.722425000	17.406996000
C	18.973444000	21.905118000	17.022138000
C	15.670384000	19.533769000	16.418250000
C	14.836943000	20.516783000	17.000034000
H	15.271405000	21.301674000	17.639976000
C	13.459460000	20.535031000	16.726280000
H	12.826743000	21.326513000	17.154361000
C	12.889280000	19.551613000	15.899188000
H	11.808751000	19.563993000	15.692261000
C	13.708153000	18.561875000	15.331115000
H	13.273231000	17.791817000	14.676198000
C	15.091039000	18.560122000	15.577893000
H	15.725251000	17.808453000	15.088712000
P	19.319235000	17.259465000	17.414277000
C	18.175327000	15.742713000	17.586234000
H	18.199300000	15.507218000	18.675387000
C	16.739936000	16.068890000	17.159582000

H	16.668925000	16.113937000	16.055226000
H	16.366748000	17.042797000	17.534524000
H	16.043470000	15.275293000	17.498097000
C	18.667187000	14.496972000	16.839771000
H	17.924467000	13.681562000	16.964552000
H	19.635796000	14.114319000	17.212191000
H	18.759836000	14.690145000	15.751486000
C	20.994598000	16.462096000	17.658162000
H	21.019874000	15.646800000	16.905237000
C	21.095362000	15.852041000	19.061432000
H	22.054642000	15.303865000	19.160277000
H	20.284430000	15.135283000	19.297799000
H	21.085105000	16.652436000	19.827134000
C	22.166566000	17.402393000	17.395412000
H	22.208946000	18.193168000	18.169259000
H	22.120299000	17.874592000	16.396700000
H	23.117562000	16.833923000	17.451608000
P	19.972776000	20.705622000	18.018536000
C	19.978439000	21.515509000	19.706760000
H	20.092123000	22.613368000	19.589064000
C	21.063762000	20.972923000	20.638568000
H	20.975056000	21.444539000	21.639252000
H	22.082613000	21.189116000	20.261053000
H	20.975247000	19.874394000	20.762702000
C	18.558479000	21.188594000	20.204617000
H	18.448135000	20.062814000	20.248194000
H	17.772497000	21.649457000	19.577451000
H	18.385327000	21.513281000	21.251296000
C	21.667526000	20.813348000	17.240467000
H	22.228772000	20.011001000	17.759001000
C	21.544181000	20.463096000	15.750414000
H	21.030498000	21.273475000	15.193934000
H	20.972029000	19.531295000	15.577243000
H	22.551322000	20.334160000	15.303960000
C	22.409885000	22.138071000	17.452608000
H	23.468497000	22.011476000	17.143496000
H	22.414863000	22.473671000	18.507964000
H	21.988475000	22.951555000	16.830991000
H	14.704417000	17.338947000	22.213508000
H	19.860468000	18.542695000	19.427635000
H	16.299786000	19.546336000	19.806045000
H	17.241211000	16.972046000	19.361464000
H	15.503544000	18.213514000	19.335979000
H	17.916081000	17.747643000	20.624884000

CP22(H)

N	17.239273000	19.817433000	20.956781000
N	17.877119000	20.790138000	20.394887000
Fe	16.550509000	18.505332000	21.808179000
P	16.352611000	19.181381000	23.869950000
C	17.652457000	18.451056000	24.969954000
C	17.910027000	18.837329000	26.300757000
H	17.322119000	19.641818000	26.769452000
C	18.940834000	18.210218000	27.021131000
H	19.144598000	18.509476000	28.061142000
C	19.719398000	17.208223000	26.410734000
H	20.530277000	16.721819000	26.975238000
C	19.474876000	16.838043000	25.076591000
H	20.103012000	16.067448000	24.602150000
C	18.442832000	17.462558000	24.348517000
P	18.011183000	17.102825000	22.570668000
C	19.713573000	17.229897000	21.746354000
H	20.328717000	16.385175000	22.131326000
C	20.366779000	18.566387000	22.123266000
H	20.531720000	18.669368000	23.213803000
H	21.352586000	18.669866000	21.621881000
H	19.726727000	19.408956000	21.789366000
C	19.579051000	17.121312000	20.222145000
H	19.120914000	16.168054000	19.896106000
H	18.939064000	17.944766000	19.842995000
H	20.575685000	17.199128000	19.737153000
C	17.719652000	15.239272000	22.739666000
H	18.663795000	14.816698000	23.149312000
C	17.428112000	14.572411000	21.392814000
H	18.336513000	14.493895000	20.763323000
H	17.040210000	13.542785000	21.542085000
H	16.670302000	15.145246000	20.817258000
C	16.593552000	15.039079000	23.764883000
H	16.930837000	15.274586000	24.793310000
H	15.734017000	15.703913000	23.542128000
H	16.229920000	13.989814000	23.757409000
C	16.539479000	20.971247000	24.276590000
C	15.543338000	21.776219000	24.864573000
H	14.603173000	21.322843000	25.212553000
C	15.741309000	23.161640000	25.003847000
H	14.956008000	23.779877000	25.466496000
C	16.934216000	23.756650000	24.556471000
H	17.085524000	24.841932000	24.664125000
C	17.935353000	22.956943000	23.970442000
H	18.874254000	23.414963000	23.619781000
C	17.737544000	21.576030000	23.826858000
H	18.513736000	20.949548000	23.359376000
C	14.720725000	18.698037000	24.603447000
C	14.393592000	18.718312000	25.972615000
H	15.123954000	19.069388000	26.716697000
C	13.129742000	18.270341000	26.399099000
H	12.882924000	18.267546000	27.472210000
C	12.189439000	17.820432000	25.457101000
H	11.202604000	17.463662000	25.790441000
C	12.505520000	17.820386000	24.085139000
H	11.756406000	17.466148000	23.361393000
C	13.774554000	18.249333000	23.648375000
P	14.376339000	18.269832000	21.881905000
C	13.463003000	19.787258000	21.173576000
H	13.732203000	19.734107000	20.094894000
C	11.937626000	19.775531000	21.315962000
H	11.633358000	19.830022000	22.382283000
H	11.460321000	18.880687000	20.868683000
H	11.501391000	20.662266000	20.806937000
C	14.062464000	21.072289000	21.749604000
H	13.629654000	21.963574000	21.246628000
H	15.162933000	21.089271000	21.621570000
H	13.844415000	21.165986000	22.833253000
C	13.400355000	16.869888000	21.079695000
H	12.328449000	17.107871000	21.248407000
C	13.702739000	15.512243000	21.716682000
H	13.036332000	14.732267000	21.290133000
H	13.551209000	15.517244000	22.814519000
H	14.747468000	15.205093000	21.521813000
C	13.660980000	16.853786000	19.569039000
H	14.743935000	16.724672000	19.359128000
H	13.331676000	17.786291000	19.067676000
H	13.116743000	16.011119000	19.091849000
H	16.442688000	17.372223000	20.772049000
H	17.850093000	21.749411000	20.781710000
H	18.551279000	20.630680000	19.625689000

DP222(H)

N	17.280897000	18.684790000	20.463402000
N	16.443353000	19.434696000	19.415546000
Fe	16.509015000	18.226424000	21.994567000
P	16.319222000	19.000515000	23.953457000
C	17.549352000	18.278849000	25.144259000
C	17.676263000	18.586072000	26.513453000
H	17.016470000	19.332295000	26.982944000
C	18.670585000	17.958395000	27.285932000
H	18.759459000	18.188944000	28.359119000
C	19.557103000	17.047565000	26.683862000
H	20.338835000	16.559057000	27.286509000
C	19.457821000	16.770946000	25.307874000
H	20.172149000	16.073022000	24.842609000
C	18.454220000	17.382007000	24.529927000

P	18.259509000	17.142538000	22.686736000
C	20.023631000	17.567199000	22.140236000
H	20.724095000	17.000737000	22.794409000
C	20.229337000	19.071789000	22.351578000
H	20.020772000	19.382296000	23.396087000
H	21.275607000	19.363482000	22.114395000
H	19.546530000	19.636949000	21.684902000
C	20.262869000	17.181294000	20.673733000
H	20.262445000	16.083521000	20.521506000
H	19.469322000	17.633683000	20.041392000
H	21.252579000	17.557405000	20.331936000
C	18.208071000	15.254960000	22.530906000
H	19.251665000	14.893464000	22.671281000
C	17.711676000	14.873496000	21.129590000
H	18.317867000	15.325255000	20.322109000
H	17.725693000	13.770139000	20.995428000
H	16.672155000	15.234769000	20.990052000
C	17.305766000	14.632709000	23.603076000
H	17.702889000	14.771952000	24.627403000
H	16.293925000	15.089570000	23.565935000
H	17.193013000	13.540942000	23.429077000
C	16.580537000	20.819750000	24.250164000
C	15.953716000	21.563325000	25.270253000
H	15.248361000	21.076388000	25.960170000
C	16.209561000	22.940887000	25.401033000
H	15.710174000	23.516376000	26.196541000
C	17.095201000	23.582864000	24.518127000
H	17.292606000	24.661357000	24.621640000
C	17.720699000	22.844455000	23.495456000
H	18.408525000	23.345159000	22.795530000
C	17.456612000	21.473182000	23.359034000
H	17.899875000	20.876707000	22.543397000
C	14.614853000	18.706610000	24.630670000
C	14.218432000	18.616351000	25.979132000
H	14.945585000	18.780694000	26.787835000
C	12.888316000	18.287843000	26.304277000
H	12.589226000	18.198627000	27.360420000
C	11.948809000	18.069949000	25.283828000
H	10.909342000	17.808084000	25.535852000
C	12.337862000	18.169128000	23.933073000
H	11.594114000	17.974689000	23.146223000
C	13.671147000	18.470474000	23.596498000
P	14.364409000	18.496095000	21.856004000
C	13.537115000	20.071706000	21.149827000
H	13.735363000	19.988996000	20.054971000
C	12.024495000	20.213812000	21.338644000
H	11.765155000	20.366453000	22.406244000
H	11.461732000	19.331326000	20.972298000
H	11.645554000	21.096919000	20.779594000
C	14.290135000	21.290616000	21.694052000
H	13.942244000	22.228417000	21.211046000
H	15.385683000	21.201136000	21.545502000
H	14.136612000	21.395722000	22.788397000
C	13.368384000	17.143795000	20.988951000
H	12.297224000	17.434196000	21.046523000
C	13.552208000	15.783549000	21.668322000
H	12.884193000	15.026536000	21.204080000
H	13.323545000	15.820186000	22.752181000
H	14.599058000	15.435804000	21.563065000
C	13.790205000	17.083550000	19.515138000
H	14.880621000	16.879214000	19.440765000
H	13.566378000	18.022403000	18.967345000
H	13.256587000	16.265505000	18.985758000
H	15.863901000	16.956087000	22.556423000
H	17.039918000	20.057861000	18.843197000
H	15.992933000	18.773987000	18.756902000
H	15.685744000	20.022616000	19.816747000

D’(H)

N	17.051006000	19.269165000	20.753572000
Fe	16.546502000	18.414328000	21.988655000
P	16.340645000	19.138260000	23.990602000
C	17.521562000	18.277773000	25.136185000
C	17.684721000	18.543641000	26.509732000
H	17.085955000	19.322958000	27.005027000
C	18.637206000	17.828910000	27.256014000
H	18.756321000	18.034063000	28.331015000
C	19.443664000	16.862961000	26.628012000
H	20.192848000	16.306160000	27.211687000
C	19.306625000	16.617954000	25.250987000
H	19.958168000	15.875520000	24.764169000
C	18.346460000	17.321413000	24.498139000
P	18.167974000	17.138636000	22.650892000
C	19.909583000	17.633409000	22.115773000
H	20.595376000	16.957032000	22.673547000
C	20.172247000	19.083432000	22.534508000
H	20.002580000	19.249679000	23.617115000
H	21.223019000	19.363083000	22.309302000
H	19.505240000	19.769821000	21.975825000
C	20.116047000	17.443556000	20.610126000
H	20.097491000	16.377634000	20.308948000
H	19.330319000	17.983779000	20.039978000
H	21.102043000	17.854763000	20.305617000
C	18.104790000	15.273376000	22.385984000
H	19.152177000	14.914968000	22.498528000
C	17.613776000	14.976080000	20.963328000
H	18.250698000	15.439203000	20.186098000
H	17.594880000	13.880582000	20.782255000
H	16.589206000	15.374006000	20.817288000
C	17.217077000	14.594234000	23.436269000
H	17.658476000	14.627266000	24.450699000
H	16.222427000	15.084554000	23.486544000
H	17.056446000	13.528156000	23.170665000
C	16.656436000	20.930099000	24.320303000
C	16.187640000	21.619924000	25.458077000
H	15.559788000	21.111873000	26.203979000
C	16.501410000	22.978181000	25.638152000
H	16.128405000	23.509710000	26.527432000
C	17.281909000	23.656650000	24.685727000
H	17.525192000	24.720840000	24.829630000
C	17.739248000	22.976148000	23.543197000
H	18.336426000	23.506713000	22.785324000
C	17.422600000	21.620982000	23.358420000
H	17.738087000	21.077886000	22.452397000
C	14.623704000	18.831855000	24.625208000
C	14.206373000	18.855326000	25.969213000
H	14.921123000	19.075006000	26.775075000
C	12.869131000	18.570375000	26.299407000
H	12.552836000	18.580979000	27.353781000
C	11.944569000	18.264904000	25.287498000
H	10.898386000	18.038235000	25.544156000
C	12.357827000	18.227063000	23.943239000
H	11.629090000	17.957447000	23.164420000
C	13.696929000	18.498013000	23.602612000
P	14.375718000	18.378711000	21.871356000
C	13.653656000	19.889982000	20.999643000
H	14.071806000	19.791707000	19.973561000
C	12.124639000	19.932095000	20.925088000
H	11.670469000	20.040927000	21.931413000
H	11.687567000	19.035134000	20.442720000
H	11.803283000	20.810787000	20.326205000
C	14.229041000	21.163333000	21.628528000
H	13.869156000	22.058696000	21.079085000
H	15.334528000	21.157099000	21.598916000
H	13.914714000	21.274487000	22.688124000
C	13.438649000	16.933652000	21.129877000
H	12.360015000	17.190663000	21.206228000
C	13.692665000	15.643909000	21.913237000
H	13.067081000	14.821660000	21.506185000
H	13.450487000	15.751103000	22.989749000
H	14.752852000	15.335146000	21.836127000
C	13.820642000	16.799793000	19.651344000

H	14.917536000	16.661952000	19.544784000
H	13.535848000	17.692225000	19.058108000
H	13.314610000	15.921874000	19.197902000
H	16.019536000	17.158457000	22.895456000

DP1’(H)

N	17.051588000	19.237293000	20.595817000
Fe	16.610009000	18.401417000	21.918879000
P	16.404920000	19.114894000	23.956955000
C	17.639046000	18.373451000	25.104118000
C	17.855220000	18.770079000	26.437407000
H	17.284081000	19.603964000	26.873263000
C	18.827283000	18.107218000	27.203602000
H	18.998806000	18.408664000	28.247919000
C	19.589310000	17.066628000	26.638293000
H	20.351106000	16.553433000	27.244322000
C	19.398042000	16.692619000	25.298147000
H	20.015734000	15.894050000	24.859240000
C	18.422244000	17.352969000	24.526527000
P	18.148494000	17.051572000	22.726666000
C	19.842909000	17.467006000	22.017273000
H	20.521896000	16.771877000	22.560269000
C	20.208322000	18.913622000	22.365899000
H	20.139496000	19.123436000	23.451625000
H	21.251119000	19.118524000	22.048072000
H	19.543574000	19.618851000	21.826647000
C	19.941312000	17.213258000	20.511685000
H	19.884751000	16.138742000	20.253909000
H	19.135413000	17.751076000	19.968378000
H	20.914277000	17.591745000	20.135532000
C	17.928893000	15.196889000	22.614367000
H	18.931202000	14.775493000	22.848975000
C	17.511748000	14.760014000	21.204254000
H	18.255770000	15.030919000	20.432936000
H	17.382227000	13.658686000	21.176409000
H	16.547199000	15.220716000	20.911411000
C	16.920601000	14.743460000	23.680343000
H	17.311159000	14.867280000	24.708352000
H	15.971337000	15.316133000	23.612352000
H	16.672561000	13.672119000	23.534963000
C	16.573549000	20.919317000	24.260763000
C	15.767717000	21.615912000	25.183961000
H	14.979593000	21.093796000	25.745859000
C	15.958427000	22.994620000	25.378569000
H	15.322880000	23.534510000	26.097018000
C	16.948091000	23.680889000	24.654857000
H	17.091503000	24.761650000	24.806304000
C	17.749947000	22.986720000	23.730628000
H	18.521008000	23.521877000	23.155666000
C	17.561625000	21.611527000	23.529695000
H	18.168254000	21.071776000	22.786816000
C	14.742417000	18.659748000	24.618814000
C	14.380841000	18.577987000	25.975782000
H	15.118391000	18.775474000	26.767010000
C	13.066559000	18.222126000	26.324376000
H	12.786032000	18.147701000	27.385767000
C	12.114695000	17.956090000	25.324488000
H	11.087224000	17.676953000	25.602227000
C	12.472681000	18.027047000	23.967214000
H	11.724266000	17.795521000	23.195317000
C	13.791918000	18.366260000	23.611379000
P	14.427826000	18.428087000	21.884169000
C	13.712581000	20.001684000	21.135523000
H	13.988985000	19.901288000	20.062848000
C	12.187644000	20.087713000	21.264290000
H	11.878924000	20.228586000	22.320040000
H	11.662288000	19.198975000	20.862676000
H	11.821013000	20.966331000	20.693978000
C	14.397107000	21.246246000	21.704045000
H	14.009105000	22.151827000	21.193967000
H	15.493466000	21.213166000	21.561647000
H	14.195630000	21.361997000	22.788341000
C	13.578108000	17.056825000	20.942248000
H	12.511012000	17.362081000	20.893409000
C	13.669807000	15.707638000	21.657716000
H	13.101531000	14.945062000	21.086451000
H	13.255108000	15.738560000	22.683817000
H	14.718032000	15.356619000	21.723512000
C	14.148077000	17.000957000	19.518237000
H	15.225373000	16.737790000	19.531561000
H	14.045160000	17.965126000	18.981593000
H	13.610918000	16.230793000	18.927764000
H	15.999587000	17.037199000	22.217499000
H	17.174388000	19.854843000	19.772055000

DP11’(H)

N	17.227810000	19.956587000	21.273323000
Fe	16.598061000	18.481208000	21.965254000
P	16.413151000	19.171838000	24.001179000
C	17.676698000	18.362542000	25.046265000
C	17.927086000	18.736250000	26.381288000
H	17.365445000	19.556287000	26.852242000
C	18.924274000	18.064485000	27.103846000
H	19.122310000	18.346888000	28.148348000
C	19.679017000	17.043505000	26.496155000
H	20.464253000	16.527536000	27.068208000
C	19.450316000	16.688503000	25.157950000
H	20.061050000	15.903640000	24.687471000
C	18.444825000	17.354186000	24.429299000
P	18.117325000	17.015260000	22.663150000
C	19.732357000	17.396895000	21.781997000
H	20.384047000	16.555556000	22.110083000
C	20.324730000	18.724033000	22.259071000
H	20.455813000	18.766260000	23.357443000
H	21.323686000	18.860431000	21.797396000
H	19.695515000	19.578047000	21.934494000
C	19.578848000	17.362528000	20.257330000
H	19.215804000	16.390931000	19.874730000
H	18.891322000	18.158094000	19.901979000
H	20.564793000	17.556087000	19.788466000
C	17.832438000	15.175577000	22.611286000
H	18.821997000	14.741898000	22.873548000
C	17.438316000	14.694916000	21.207139000
H	18.291060000	14.711210000	20.503343000
H	17.077774000	13.648093000	21.261333000
H	16.621032000	15.300407000	20.765093000
C	16.823000000	14.801196000	23.708376000
H	17.252671000	14.915609000	24.721486000
H	15.899736000	15.415985000	23.667693000
H	16.517911000	13.742581000	23.585414000
C	16.665866000	20.944402000	24.317965000
C	15.619017000	21.793572000	24.732792000
H	14.610837000	21.393140000	24.909406000
C	15.873211000	23.159491000	24.933145000
H	15.058821000	23.820047000	25.265826000
C	17.159533000	23.679552000	24.711076000
H	17.351900000	24.752044000	24.864523000
C	18.205152000	22.831581000	24.301003000
H	19.214977000	23.235833000	24.136589000
C	17.964801000	21.464620000	24.107701000
H	18.789781000	20.801142000	23.805866000
C	14.740588000	18.739045000	24.612834000
C	14.370833000	18.801386000	25.968677000
H	15.095453000	19.100015000	26.739450000
C	13.056086000	18.475825000	26.339339000
H	12.765011000	18.518428000	27.399155000
C	12.116832000	18.093005000	25.366045000
H	11.089703000	17.835843000	25.663891000
C	12.480559000	18.028658000	24.010846000
H	11.736549000	17.715423000	23.264232000
C	13.797151000	18.351096000	23.629495000
P	14.389840000	18.324384000	21.901767000

C	13.687366000	19.849833000	21.039951000
H	14.242026000	19.869260000	20.076949000
C	12.183509000	19.712089000	20.767056000
H	11.601753000	19.579602000	21.701767000
H	11.940222000	18.882772000	20.076482000
H	11.825535000	20.648056000	20.291178000
C	14.004416000	21.121948000	21.824172000
H	13.661435000	22.002796000	21.245089000
H	15.087844000	21.250989000	22.000881000
H	13.475367000	21.137215000	22.797517000
C	13.623208000	16.873498000	21.029937000
H	12.545518000	17.135479000	20.974821000
C	13.763818000	15.557701000	21.792312000
H	13.174119000	14.775789000	21.272258000
H	13.389165000	15.624040000	22.832315000
H	14.811520000	15.206897000	21.818928000
C	14.197784000	16.811316000	19.606664000
H	15.296610000	16.647255000	19.615679000
H	13.998415000	17.732971000	19.024988000
H	13.743587000	15.963281000	19.056148000
H	16.004605000	17.103093000	22.332228000
H	17.436312000	20.896282000	21.647553000
H	17.399088000	19.962495000	20.248569000

E’(H)

N	17.080024000	19.318689000	20.801378000
Fe	16.538649000	18.410188000	21.990351000
P	16.335640000	19.144882000	24.008466000
C	17.481724000	18.303480000	25.138507000
C	17.689279000	18.598741000	26.523161000
H	17.108704000	19.399319000	27.008569000
C	18.645688000	17.899574000	27.264110000
H	18.786127000	18.142026000	28.330657000
C	19.442030000	16.890419000	26.660047000
H	20.192607000	16.340873000	27.248419000
C	19.276733000	16.623559000	25.277292000
H	19.914836000	15.865260000	24.793268000
C	18.325877000	17.317416000	24.518555000
P	18.149538000	17.132043000	22.681484000
C	19.894153000	17.624948000	22.136187000
H	20.583610000	16.940791000	22.679785000
C	20.157412000	19.067878000	22.577621000
H	19.979372000	19.212664000	23.661824000
H	21.208404000	19.355286000	22.358972000
H	19.486443000	19.760002000	22.030531000
C	20.092415000	17.467019000	20.625538000
H	20.091573000	16.406530000	20.304185000
H	19.285724000	18.001240000	20.078880000
H	21.066386000	17.903758000	20.315085000
C	18.101838000	15.261682000	22.407922000
H	19.146556000	14.902567000	22.544557000
C	17.638666000	14.955642000	20.978086000
H	18.295776000	15.406619000	20.210341000
H	17.610205000	13.858715000	20.801475000
H	16.622287000	15.366810000	20.809452000
C	17.191347000	14.581075000	23.436779000
H	17.590020000	14.656625000	24.466591000
H	16.185108000	15.050201000	23.432768000
H	17.067985000	13.504047000	23.192975000
C	16.641896000	20.942541000	24.266114000
C	16.196601000	21.641280000	25.419203000
H	15.542678000	21.143687000	26.151221000
C	16.561824000	22.986181000	25.616171000
H	16.210426000	23.519175000	26.514934000
C	17.361045000	23.654729000	24.672765000
H	17.642763000	24.707627000	24.831689000
C	17.788756000	22.968332000	23.510412000
H	18.388149000	23.492862000	22.748791000
C	17.431663000	21.627462000	23.314408000
H	17.724248000	21.079992000	22.401789000
C	14.619785000	18.847277000	24.631671000
C	14.208313000	18.804478000	25.977663000
H	14.931276000	19.018636000	26.779701000
C	12.884050000	18.470907000	26.318320000
H	12.573459000	18.457953000	27.374779000
C	11.955723000	18.150727000	25.295148000
H	10.919581000	17.879666000	25.552608000
C	12.362325000	18.154903000	23.952849000
H	11.639659000	17.863870000	23.174215000
C	13.688679000	18.499949000	23.595438000
P	14.370754000	18.395330000	21.880802000
C	13.642326000	19.899415000	20.982361000
H	14.039419000	19.792783000	19.948729000
C	12.112719000	19.950076000	20.941054000
H	11.684609000	20.034071000	21.961662000
H	11.663000000	19.061619000	20.453403000
H	11.773035000	20.840836000	20.369174000
C	14.237691000	21.172563000	21.591218000
H	13.890961000	22.069643000	21.034092000
H	15.343267000	21.142672000	21.563204000
H	13.931689000	21.294693000	22.651968000
C	13.427247000	16.958804000	21.111331000
H	12.347994000	17.212488000	21.192480000
C	13.681034000	15.655628000	21.872794000
H	13.057985000	14.835871000	21.454553000
H	13.439705000	15.749116000	22.950683000
H	14.743208000	15.353421000	21.793718000
C	13.805594000	16.840082000	19.630923000
H	14.905003000	16.717063000	19.525911000
H	13.512304000	17.735663000	19.046198000
H	13.310123000	15.961309000	19.165671000
H	15.979861000	17.156009000	22.888323000

EP11’(H)

N	17.020922000	18.920529000	20.319116000
Fe	16.557569000	18.392036000	21.978501000
P	16.362920000	19.122766000	23.999790000
C	17.591309000	18.313572000	25.110206000
C	17.776942000	18.645679000	26.466683000
H	17.186405000	19.450511000	26.930232000
C	18.741720000	17.963551000	27.224306000
H	18.882893000	18.217893000	28.285594000
C	19.533840000	16.965620000	26.626804000
H	20.290061000	16.432767000	27.222806000
C	19.379221000	16.660731000	25.264988000
H	20.023248000	15.896739000	24.802835000
C	18.409091000	17.336690000	24.497965000
P	18.224899000	17.095874000	22.672204000
C	19.958941000	17.542112000	22.088099000
H	20.622377000	16.963313000	22.768477000
C	20.195501000	19.038771000	22.315554000
H	20.014182000	19.333439000	23.368734000
H	21.246947000	19.297636000	22.073340000
H	19.537982000	19.652052000	21.665457000
C	20.248997000	17.115401000	20.646257000
H	20.202836000	16.017583000	20.512735000
H	19.553522000	17.582373000	19.918789000
H	21.274924000	17.436123000	20.368808000
C	18.054808000	15.247804000	22.427950000
H	19.079750000	14.832534000	22.549490000
C	17.548667000	14.957920000	21.008122000
H	18.188271000	15.401357000	20.222461000
H	17.508147000	13.862469000	20.836788000
H	16.528822000	15.365972000	20.863187000
C	17.130642000	14.647800000	23.495038000
H	17.575871000	14.680901000	24.507576000
H	16.160661000	15.186055000	23.537212000
H	16.913386000	13.587098000	23.253030000
C	16.588489000	20.918860000	24.332954000
C	15.771500000	21.655799000	25.214023000
H	14.939899000	21.170881000	25.745293000
C	16.008755000	23.027875000	25.407000000

H	15.365662000	23.598346000	26.094516000
C	17.054510000	23.669402000	24.722462000
H	17.234445000	24.744695000	24.874063000
C	17.865451000	22.937582000	23.835946000
H	18.680015000	23.436662000	23.289216000
C	17.630292000	21.569397000	23.637305000
H	18.247489000	21.002135000	22.924243000
C	14.675517000	18.731660000	24.644852000
C	14.302517000	18.700550000	26.001349000
H	15.042912000	18.887914000	26.792046000
C	12.973745000	18.410432000	26.355260000
H	12.688166000	18.378162000	27.417439000
C	12.015919000	18.156169000	25.358941000
H	10.976282000	17.928100000	25.638187000
C	12.384230000	18.172925000	24.002968000
H	11.630664000	17.943226000	23.235447000
C	13.716024000	18.451781000	23.638991000
P	14.342853000	18.410520000	21.902504000
C	13.686096000	19.972199000	21.084043000
H	14.057243000	19.882884000	20.039242000
C	12.157463000	20.066183000	21.068343000
H	11.745978000	20.162947000	22.093668000
H	11.673717000	19.199373000	20.576469000
H	11.851443000	20.972827000	20.505340000
C	14.333660000	21.202837000	21.728318000
H	14.002302000	22.122455000	21.203483000
H	15.439436000	21.161993000	21.678174000
H	14.043400000	21.304630000	22.794232000
C	13.445615000	16.996720000	21.080021000
H	12.367796000	17.265078000	21.119639000
C	13.654009000	15.681979000	21.837497000
H	13.019544000	14.889033000	21.390619000
H	13.386304000	15.761536000	22.909679000
H	14.705637000	15.342286000	21.771809000
C	13.887907000	16.903934000	19.613970000
H	14.983114000	16.740163000	19.538365000
H	13.643430000	17.817289000	19.035435000
H	13.378143000	16.052423000	19.118184000
H	16.000239000	17.190237000	22.725480000
H	16.384091000	19.298561000	19.603816000
H	17.952020000	18.853933000	19.886783000

FP1’(H)

N	17.069493000	18.957874000	20.562139000
Fe	16.459460000	18.145012000	22.043677000
P	16.293227000	18.917002000	23.979552000
C	17.486812000	18.157171000	25.190463000
C	17.573135000	18.381172000	26.581183000
H	16.900744000	19.096436000	27.078199000
C	18.547726000	17.709657000	27.347051000
H	18.595867000	17.871033000	28.435936000
C	19.465352000	16.850247000	26.720816000
H	20.232503000	16.331633000	27.317713000
C	19.412872000	16.662715000	25.323307000
H	20.153322000	16.009390000	24.834390000
C	18.420593000	17.302283000	24.555067000
P	18.276740000	17.159747000	22.691551000
C	20.032490000	17.719664000	22.207611000
H	20.775613000	16.999624000	22.622442000
C	20.288872000	19.111490000	22.795374000
H	20.253375000	19.116784000	23.903510000
H	21.287255000	19.491679000	22.485337000
H	19.524750000	19.828630000	22.434062000
C	20.118863000	17.762182000	20.673475000
H	20.107536000	16.748823000	20.222198000
H	19.243219000	18.333301000	20.282000000
H	21.063287000	18.252885000	20.345906000
C	18.382326000	15.274244000	22.439760000
H	19.448410000	14.983139000	22.574897000
C	17.932890000	14.935687000	21.011940000
H	18.539750000	15.448666000	20.240763000
H	17.994818000	13.840736000	20.825250000
H	16.882872000	15.266597000	20.867250000
C	17.515008000	14.520104000	23.454411000
H	17.883891000	14.623913000	24.493390000
H	16.474254000	14.907452000	23.422579000
H	17.483860000	13.435129000	23.212611000
C	16.686695000	20.731337000	24.204905000
C	16.360197000	21.483698000	25.352699000
H	15.801127000	21.023119000	26.181292000
C	16.726856000	22.839752000	25.442075000
H	16.463671000	23.419911000	26.341152000
C	17.422724000	23.454337000	24.386001000
H	17.708489000	24.515936000	24.457042000
C	17.741067000	22.710057000	23.233378000
H	18.272886000	23.191121000	22.396464000
C	17.370262000	21.358733000	23.142585000
H	17.563311000	20.747894000	22.237019000
C	14.555978000	18.792291000	24.644864000
C	14.123287000	18.827344000	25.985832000
H	14.841430000	18.993172000	26.802017000
C	12.764202000	18.626035000	26.300072000
H	12.438854000	18.631209000	27.352609000
C	11.829677000	18.418402000	25.273661000
H	10.767095000	18.258311000	25.515962000
C	12.254243000	18.399005000	23.928643000
H	11.513439000	18.217871000	23.135380000
C	13.614242000	18.566571000	23.603822000
P	14.337251000	18.412542000	21.875194000
C	13.612571000	19.935780000	20.985113000
H	14.029485000	19.813656000	19.958774000
C	12.089985000	20.055080000	20.894443000
H	11.639195000	20.222630000	21.894870000
H	11.608518000	19.160749000	20.449283000
H	11.804429000	20.924526000	20.260985000
C	14.246844000	21.189504000	21.593569000
H	13.960163000	22.100313000	21.023547000
H	15.349718000	21.105826000	21.594309000
H	13.924879000	21.336661000	22.646899000
C	13.282835000	17.021571000	21.143398000
H	12.219020000	17.334687000	21.220959000
C	13.460207000	15.713631000	21.917638000
H	12.791063000	14.924303000	21.510582000
H	13.225888000	15.836036000	22.995220000
H	14.509063000	15.360396000	21.848806000
C	13.652893000	16.856507000	19.664593000
H	14.744410000	16.671948000	19.565255000
H	13.413782000	17.759467000	19.065741000
H	13.108462000	16.000773000	19.209326000
H	15.904294000	16.779909000	22.516504000
H	16.295065000	19.480288000	20.076410000

GP11’(H)

N	17.031285000	19.035163000	20.412233000
Fe	16.468868000	18.144980000	21.988284000
P	16.343766000	19.003902000	23.935646000
C	17.594813000	18.224084000	25.082912000
C	17.742010000	18.508203000	26.456600000
H	17.063634000	19.223808000	26.947085000
C	18.788014000	17.918692000	27.194704000
H	18.897011000	18.139672000	28.268806000
C	19.701021000	17.061565000	26.556013000
H	20.525548000	16.607691000	27.129278000
C	19.569746000	16.792470000	25.178906000
H	20.302165000	16.136232000	24.680806000
C	18.513469000	17.365121000	24.439379000
P	18.247888000	17.116897000	22.604912000
C	20.000013000	17.556186000	21.989510000
H	20.740629000	16.963188000	22.572344000
C	20.225327000	19.048147000	22.259505000
H	20.237352000	19.265898000	23.347169000
H	21.193118000	19.394193000	21.834580000

H	19.406766000	19.651746000	21.812220000
C	20.154273000	17.219568000	20.500155000
H	20.173568000	16.125891000	20.321302000
H	19.317171000	17.643512000	19.905475000
H	21.105111000	17.633929000	20.097415000
C	18.273665000	15.211008000	22.488998000
H	19.333551000	14.886033000	22.591939000
C	17.728544000	14.778897000	21.121946000
H	18.312179000	15.190343000	20.275880000
H	17.725110000	13.670448000	21.026761000
H	16.687702000	15.148797000	21.005963000
C	17.433088000	14.587296000	23.609100000
H	17.875902000	14.744786000	24.611753000
H	16.418932000	15.041755000	23.610987000
H	17.318490000	13.492062000	23.454831000
C	16.579344000	20.770973000	24.451996000
C	15.513520000	21.662274000	24.757715000
H	14.497837000	21.261251000	24.898492000
C	15.735837000	23.039552000	24.901474000
H	14.888946000	23.701237000	25.149740000
C	17.030352000	23.582733000	24.747836000
H	17.203044000	24.664325000	24.861441000
C	18.101185000	22.709079000	24.445101000
H	19.122128000	23.110025000	24.329206000
C	17.877805000	21.336215000	24.293262000
H	18.718690000	20.669009000	24.044939000
C	14.635909000	18.699661000	24.624250000
C	14.257768000	18.682036000	25.980375000
H	15.007102000	18.858524000	26.766189000
C	12.914512000	18.447515000	26.340859000
H	12.627148000	18.417609000	27.404256000
C	11.944552000	18.259871000	25.342787000
H	10.893092000	18.082057000	25.620333000
C	12.315229000	18.291363000	23.982446000
H	11.545108000	18.137552000	23.211582000
C	13.662204000	18.490452000	23.613987000
P	14.337872000	18.411337000	21.869828000
C	13.614633000	19.963120000	21.030648000
H	13.820051000	19.758548000	19.954878000
C	12.115057000	20.217513000	21.202293000
H	11.867769000	20.454429000	22.258140000
H	11.493490000	19.350961000	20.895893000
H	11.788185000	21.085390000	20.586761000
C	14.453213000	21.175071000	21.449043000
H	14.217853000	22.062183000	20.820487000
H	15.535688000	20.945443000	21.367992000
H	14.260098000	21.453446000	22.504882000
C	13.250951000	17.057139000	21.103299000
H	12.191154000	17.389075000	21.167094000
C	13.402637000	15.734225000	21.858697000
H	12.739613000	14.954086000	21.424833000
H	13.150282000	15.835724000	22.933830000
H	14.451212000	15.377236000	21.802099000
C	13.641160000	16.897847000	19.628651000
H	14.731179000	16.696578000	19.545875000
H	13.426526000	17.807920000	19.031699000
H	13.091643000	16.054077000	19.157305000
H	15.891085000	16.824227000	22.510299000
H	16.457104000	19.718596000	19.898638000
H	18.010702000	19.278598000	20.211916000

DP1122

N	16.245600000	18.539626000	20.046813000
N	17.479858000	17.795283000	19.684229000
Fe	15.509386000	18.607700000	21.892659000
P	14.812910000	19.415793000	23.742443000
C	15.563941000	18.619404000	25.216642000
C	15.301293000	18.960854000	26.558672000
H	14.614739000	19.785567000	26.802790000
C	15.942681000	18.260135000	27.591451000
H	15.737460000	18.519856000	28.641068000
C	16.852718000	17.231468000	27.283321000
H	17.350514000	16.676758000	28.093258000
C	17.146837000	16.922365000	25.945616000
H	17.879050000	16.129981000	25.726434000
C	16.511801000	17.622990000	24.899715000
P	16.925211000	17.381950000	23.092401000
C	18.814049000	17.566195000	23.274146000
H	19.057230000	16.926433000	24.148960000
C	19.133173000	19.022747000	23.619985000
H	18.612660000	19.355804000	24.539909000
H	20.222195000	19.155182000	23.786601000
H	18.842396000	19.699067000	22.790045000
C	19.649901000	17.068917000	22.091030000
H	19.421108000	16.022486000	21.809116000
H	19.574312000	17.712388000	21.184021000
H	20.724458000	17.091280000	22.370287000
C	16.700998000	15.507363000	22.899844000
H	17.571723000	15.046184000	23.415548000
C	16.710752000	15.082181000	21.425399000
H	17.680824000	15.272424000	20.925907000
H	16.522405000	13.991800000	21.342481000
H	15.905396000	15.599516000	20.862224000
C	15.408817000	15.039836000	23.582295000
H	15.424403000	15.200766000	24.676776000
H	14.525578000	15.576853000	23.177315000
H	15.256426000	13.955348000	23.401161000
C	15.139093000	21.212591000	23.993728000
C	14.117337000	22.161103000	24.206814000
H	13.069275000	21.839371000	24.292239000
C	14.434577000	23.527250000	24.300271000
H	13.631105000	24.261194000	24.466422000
C	15.767358000	23.955810000	24.179172000
H	16.011390000	25.026794000	24.249734000
C	16.789714000	23.012271000	23.968968000
H	17.836117000	23.342198000	23.879921000
C	16.476991000	21.648508000	23.872888000
H	17.274463000	20.911803000	23.700132000
C	12.975752000	19.310675000	23.862402000
C	12.195226000	19.339833000	25.031947000
H	12.668168000	19.380837000	26.023071000
C	10.793505000	19.310662000	24.933944000
H	10.182112000	19.322875000	25.848967000
C	10.173376000	19.265127000	23.673012000
H	9.075461000	19.242881000	23.599826000
C	10.949948000	19.231349000	22.502210000
H	10.450059000	19.168604000	21.524782000
C	12.355724000	19.245940000	22.590823000
P	13.512282000	19.077728000	21.153111000
C	13.322477000	20.626514000	20.105724000
H	13.708226000	20.267869000	19.127020000
C	11.891305000	21.127816000	19.908363000
H	11.455467000	21.490080000	20.861616000
H	11.219668000	20.351160000	19.491224000
H	11.881854000	21.982334000	19.198977000
C	14.256422000	21.731758000	20.609404000
H	14.293844000	22.570779000	19.883858000
H	15.296180000	21.372591000	20.759390000
H	13.917489000	22.142604000	21.580787000
C	12.660653000	17.762762000	20.102198000
H	11.698724000	18.226350000	19.791601000
C	12.367082000	16.492629000	20.906159000
H	11.801717000	15.771454000	20.280026000
H	11.768261000	16.689366000	21.816631000
H	13.308045000	15.996341000	21.220116000
C	13.469055000	17.435195000	18.839444000
H	14.353118000	16.808400000	19.082703000
H	13.792939000	18.333837000	18.275905000
H	12.855541000	16.837240000	18.136028000
Fe	18.665066000	18.803064000	18.454702000
P	17.550410000	19.458262000	16.717610000
C	18.213560000	18.587692000	15.192989000

C	17.935924000	18.972315000	13.868177000
H	17.231499000	19.792038000	13.662225000
C	18.564851000	18.316777000	12.794291000
H	18.358690000	18.636139000	11.760910000
C	19.459474000	17.261060000	13.041183000
H	19.956798000	16.750981000	12.201568000
C	19.719170000	16.852217000	14.361979000
H	20.415356000	16.019217000	14.542899000
C	19.105033000	17.514541000	15.443350000
C	17.869056000	21.268578000	16.368388000
C	17.194048000	22.045109000	15.406014000
H	16.436812000	21.586801000	14.752692000
C	17.449786000	23.423415000	15.305733000
H	16.915369000	24.026703000	14.555483000
C	18.365846000	24.034037000	16.180559000
H	18.545900000	25.118784000	16.122848000
C	19.053187000	23.259981000	17.132372000
H	19.761477000	23.751287000	17.816096000
C	18.823034000	21.871778000	17.217794000
C	15.714823000	19.451491000	16.349297000
C	14.902929000	20.465302000	16.909411000
H	15.364237000	21.257490000	17.521963000
C	13.521714000	20.502795000	16.656473000
H	12.911363000	21.321095000	17.067538000
C	12.917207000	19.502620000	15.873392000
H	11.833807000	19.529338000	15.681648000
C	13.709333000	18.474787000	15.335042000
H	13.249032000	17.687717000	14.717680000
C	15.096373000	18.456681000	15.561546000
H	15.708822000	17.672030000	15.095757000
P	19.357706000	17.131027000	17.241864000
C	18.257948000	15.569425000	17.438504000
H	18.327973000	15.360401000	18.531579000
C	16.800544000	15.887348000	17.083062000
H	16.673684000	15.926585000	15.982296000
H	16.458609000	16.870157000	17.467162000
H	16.116136000	15.100653000	17.463890000
C	18.713455000	14.314349000	16.688601000
H	17.994930000	13.484271000	16.862504000
H	19.709382000	13.951387000	17.007659000
H	18.745965000	14.492148000	15.593356000
C	21.062095000	16.336027000	17.343720000
H	21.061562000	15.475063000	16.642160000
C	21.275825000	15.816966000	18.771776000
H	22.284394000	15.363510000	18.874368000
H	20.535745000	15.044831000	19.064254000
H	21.194604000	16.651649000	19.499077000
C	22.176397000	17.295442000	16.928769000
H	22.216082000	18.171713000	17.605192000
H	22.047023000	17.664955000	15.893258000
H	23.161021000	16.784021000	16.982693000
P	19.737473000	20.680524000	18.314055000
C	19.818815000	21.606947000	19.954734000
H	20.133036000	22.649653000	19.733848000
C	20.832148000	20.956991000	20.901458000
H	20.820827000	21.458141000	21.892415000
H	21.869226000	21.012069000	20.514125000
H	20.590614000	19.885199000	21.050645000
C	18.409114000	21.632976000	20.557218000
H	18.084392000	20.596630000	20.800514000
H	17.668101000	22.072965000	19.859212000
H	18.380720000	22.221868000	21.496760000
C	21.482560000	20.822243000	17.600728000
H	22.012303000	19.993790000	18.119758000
C	21.415265000	20.508823000	16.100667000
H	20.984874000	21.364511000	15.540515000
H	20.781393000	19.625070000	15.892574000
H	22.428745000	20.314672000	15.692218000
C	22.234492000	22.131116000	17.860908000
H	23.277891000	22.041940000	17.488577000
H	22.294795000	22.396153000	18.934619000
H	21.774397000	22.983135000	17.321110000
H	14.744246000	17.382106000	22.187629000
H	19.833976000	18.528692000	19.424938000
H	16.512755000	19.524939000	19.816587000
H	17.161450000	16.872409000	19.351695000
H	15.581302000	18.317948000	19.286862000
H	17.931902000	17.603880000	20.591643000

EP1122

N	16.762967000	19.150434000	20.734022000
N	16.888012000	18.464358000	19.420427000
Fe	15.513361000	18.158512000	21.928118000
P	15.002764000	19.295705000	23.647922000
C	15.634163000	18.463737000	25.189870000
C	15.442342000	18.919129000	26.509584000
H	14.884276000	19.849770000	26.698290000
C	15.986497000	18.203596000	27.590463000
H	15.830012000	18.559468000	28.620897000
C	16.739734000	17.039604000	27.352310000
H	17.168337000	16.477368000	28.196821000
C	16.967328000	16.605293000	26.034241000
H	17.586640000	15.710546000	25.860829000
C	16.422258000	17.314258000	24.944844000
P	16.801245000	16.929966000	23.150042000
C	18.699081000	17.052083000	23.286180000
H	18.990276000	16.390551000	24.132200000
C	19.097960000	18.490552000	23.625918000
H	18.608663000	18.855854000	24.550344000
H	20.196934000	18.575721000	23.763705000
H	18.822074000	19.183911000	22.803575000
C	19.405187000	16.573049000	22.017414000
H	19.209974000	15.505200000	21.799101000
H	19.111419000	17.175724000	21.131448000
H	20.505329000	16.685817000	22.124972000
C	16.556893000	15.048745000	23.043243000
H	17.441849000	14.571054000	23.519514000
C	16.489171000	14.644480000	21.563193000
H	17.411252000	14.899780000	21.004302000
H	16.329806000	13.549469000	21.458864000
H	15.645393000	15.169584000	21.066646000
C	15.283292000	14.611078000	23.775290000
H	15.353435000	14.752029000	24.871289000
H	14.411860000	15.198192000	23.414206000
H	15.073436000	13.536587000	23.584834000
C	15.442166000	21.067555000	24.013778000
C	14.535690000	22.066660000	24.429236000
H	13.490360000	21.804909000	24.650737000
C	14.953548000	23.404358000	24.550660000
H	14.232129000	24.172605000	24.871877000
C	16.282401000	23.763748000	24.266762000
H	16.606655000	24.811609000	24.362372000
C	17.194964000	22.773982000	23.852893000
H	18.238319000	23.044278000	23.627474000
C	16.772705000	21.443857000	23.718449000
H	17.475071000	20.673731000	23.364503000
C	13.142715000	19.306532000	23.795192000
C	12.383239000	19.464638000	24.969970000
H	12.881065000	19.627784000	25.937199000
C	10.977836000	19.400314000	24.922306000
H	10.392198000	19.511320000	25.848341000
C	10.325990000	19.191445000	23.695853000
H	9.226722000	19.139284000	23.655782000
C	11.078252000	19.034085000	22.516206000
H	10.554281000	18.852607000	21.565698000
C	12.485453000	19.076891000	22.556890000
P	13.612171000	18.770342000	21.098593000
C	13.453820000	20.393323000	20.117728000
H	14.065852000	20.192630000	19.209168000
C	12.037983000	20.770300000	19.675264000
H	11.373323000	20.915872000	20.552038000
H	11.574830000	20.009371000	19.016608000

H	12.046549000	21.730242000	19.115419000
C	14.104878000	21.540741000	20.896218000
H	14.203389000	22.444175000	20.257525000
H	15.108861000	21.284933000	21.288919000
H	13.496561000	21.821567000	21.780570000
C	12.610445000	17.584271000	20.023930000
H	11.639903000	18.074343000	19.794264000
C	12.346062000	16.268006000	20.759036000
H	11.732249000	15.588166000	20.129873000
H	11.807038000	16.423075000	21.715405000
H	13.300888000	15.755820000	20.994956000
C	13.368476000	17.346460000	18.711864000
H	14.342739000	16.854770000	18.915135000
H	13.558391000	18.279710000	18.144401000
H	12.796036000	16.673580000	18.039010000
Fe	18.454000000	19.163307000	18.387246000
P	17.565116000	19.678648000	16.510405000
C	18.262884000	18.631912000	15.127849000
C	18.080519000	18.877486000	13.754063000
H	17.463745000	19.724398000	13.416667000
C	18.694524000	18.044964000	12.801026000
H	18.562706000	18.248875000	11.726867000
C	19.478673000	16.956443000	13.221756000
H	19.961739000	16.304743000	12.476862000
C	19.650867000	16.698688000	14.594236000
H	20.273063000	15.846402000	14.907880000
C	19.053139000	17.536753000	15.557331000
C	17.953014000	21.435225000	16.022863000
C	17.341006000	22.151060000	14.975976000
H	16.583864000	21.665870000	14.340680000
C	17.662715000	23.503987000	14.767384000
H	17.176533000	24.065689000	13.954392000
C	18.588845000	24.142595000	15.611189000
H	18.827773000	25.207311000	15.462083000
C	19.205227000	23.426722000	16.654110000
H	19.916187000	23.945171000	17.315100000
C	18.897301000	22.067283000	16.863804000
C	15.744590000	19.644074000	16.140393000
C	14.934697000	20.646804000	16.722053000
H	15.405827000	21.450920000	17.310918000
C	13.545074000	20.646979000	16.527427000
H	12.932189000	21.448541000	16.964870000
C	12.935320000	19.623960000	15.778116000
H	11.843973000	19.619764000	15.634541000
C	13.728315000	18.608676000	15.217178000
H	13.260405000	17.802845000	14.630334000
C	15.123062000	18.622005000	15.391207000
H	15.736072000	17.836681000	14.926022000
P	19.226329000	17.371984000	17.408956000
C	18.098422000	15.849968000	17.754148000
H	18.000333000	15.877968000	18.864539000
C	16.712279000	16.073191000	17.141220000
H	16.744151000	15.938379000	16.040547000
H	16.320470000	17.090200000	17.328763000
H	15.978942000	15.344069000	17.545783000
C	18.631423000	14.469098000	17.360746000
H	17.875868000	13.689199000	17.600611000
H	19.560376000	14.191093000	17.895328000
H	18.828115000	14.402661000	16.269988000
C	20.926745000	16.563615000	17.596471000
H	20.877819000	15.599837000	17.047576000
C	21.163344000	16.280173000	19.082534000
H	22.161975000	15.820345000	19.243492000
H	20.407184000	15.589933000	19.505497000
H	21.110454000	17.223489000	19.663343000
C	22.065243000	17.392641000	17.003740000
H	22.190745000	18.345333000	17.552200000
H	21.904971000	17.631616000	15.934892000
H	23.022779000	16.834224000	17.082939000
P	19.647869000	20.957892000	18.169823000
C	19.695930000	22.117145000	19.664031000
H	20.250112000	23.026574000	19.347054000
C	20.440043000	21.451043000	20.825031000
H	20.473736000	22.117213000	21.713286000
H	21.484852000	21.186225000	20.563223000
H	19.932977000	20.505846000	21.110352000
C	18.275579000	22.527891000	20.063938000
H	17.714665000	21.663034000	20.472860000
H	17.695826000	22.926055000	19.206837000
H	18.293933000	23.305015000	20.856550000
C	21.459317000	20.941633000	17.584314000
H	21.876864000	20.091283000	18.166253000
C	21.481006000	20.581067000	16.093530000
H	21.159318000	21.444671000	15.475622000
H	20.802854000	19.736407000	15.862335000
H	22.504678000	20.298248000	15.769472000
C	22.305614000	22.187729000	17.862652000
H	23.343020000	22.026497000	17.496130000
H	22.378775000	22.432512000	18.940608000
H	21.914150000	23.079931000	17.332748000
H	14.573202000	17.056898000	22.422658000
H	19.294350000	18.873995000	19.680706000
H	17.759790000	19.241153000	21.004298000
H	15.951753000	18.547967000	18.989229000
H	16.484348000	20.116142000	20.492895000
H	16.946871000	17.465470000	19.687894000

EP11222(H)

N	16.824214000	17.975391000	19.357868000
N	15.582676000	17.534471000	18.693011000
Fe	18.253156000	18.941134000	18.305131000
P	17.494081000	19.412776000	16.356976000
C	18.505468000	18.602181000	15.019909000
C	18.391830000	18.871498000	13.643265000
H	17.630608000	19.575226000	13.273860000
C	19.261284000	18.248051000	12.730159000
H	19.178757000	18.469586000	11.654878000
C	20.238734000	17.349828000	13.192352000
H	20.923920000	16.866122000	12.478963000
C	20.345796000	17.065754000	14.566357000
H	21.115374000	16.357892000	14.910742000
C	19.485921000	17.690860000	15.490552000
C	17.561191000	21.234417000	15.995947000
C	16.935265000	21.873801000	14.909009000
H	16.382933000	21.285456000	14.159463000
C	16.987215000	23.274152000	14.798846000
H	16.493653000	23.777845000	13.953257000
C	17.650663000	24.031186000	15.781449000
H	17.673882000	25.129510000	15.707145000
C	18.276697000	23.391423000	16.866767000
H	18.775473000	24.000746000	17.635310000
C	18.243298000	21.986746000	16.977709000
C	15.733287000	19.061731000	15.879708000
C	14.739645000	19.740168000	16.634916000
H	15.037943000	20.563598000	17.306363000
C	13.386934000	19.376068000	16.533337000
H	12.629010000	19.915794000	17.121952000
C	13.003582000	18.320800000	15.681416000
H	11.946042000	18.024954000	15.609473000
C	13.977295000	17.657852000	14.916251000
H	13.682624000	16.842032000	14.237977000
C	15.332640000	18.027756000	15.008524000
H	16.082952000	17.499172000	14.402121000
P	19.481453000	17.417819000	17.334577000
C	18.657320000	15.676518000	17.414662000
H	18.373469000	15.596510000	18.488461000
C	17.380577000	15.674849000	16.566145000
H	17.611226000	15.494563000	15.496472000
H	16.869329000	16.659329000	16.582140000
H	16.668712000	14.885949000	16.889619000
C	19.522588000	14.464349000	17.057046000
H	18.915980000	13.534551000	17.117423000

H	20.386306000	14.330659000	17.736222000
H	19.905679000	14.532441000	16.017356000
C	21.263761000	16.954925000	17.747843000
H	21.488890000	16.036643000	17.166529000
C	21.345450000	16.626421000	19.243757000
H	22.366605000	16.282101000	19.512779000
H	20.635062000	15.830484000	19.549496000
H	21.110525000	17.526540000	19.849045000
C	22.285312000	18.023093000	17.357826000
H	22.145919000	18.947010000	17.950544000
H	22.229077000	18.289533000	16.284780000
H	23.313458000	17.652737000	17.557571000
P	19.048889000	20.966235000	18.318630000
C	18.629467000	21.969220000	19.860485000
H	18.959871000	23.012740000	19.668491000
C	19.368735000	21.424191000	21.086244000
H	19.139039000	22.035920000	21.984670000
H	20.470044000	21.424671000	20.955177000
H	19.062452000	20.377288000	21.290963000
C	17.111102000	21.959627000	20.062411000
H	16.743965000	20.924284000	20.232317000
H	16.572369000	22.364391000	19.181657000
H	16.824023000	22.566750000	20.947050000
C	20.875842000	21.343376000	17.991860000
H	21.382881000	20.566209000	18.603961000
C	21.163648000	21.076743000	16.508696000
H	20.768135000	21.899109000	15.877808000
H	20.697644000	20.134144000	16.159548000
H	22.255983000	21.007945000	16.325579000
C	21.391798000	22.721030000	18.418002000
H	22.479780000	22.795743000	18.202648000
H	21.262438000	22.917995000	19.500173000
H	20.897134000	23.539109000	17.855469000
H	19.115933000	18.784101000	19.587296000
H	15.174802000	18.343856000	18.161262000
H	14.866247000	17.139032000	19.335593000
H	15.836875000	16.818994000	17.981203000
H	17.183624000	17.138221000	19.844319000
H	16.522574000	18.621366000	20.107490000

D’

N	16.923001000	18.527346000	19.982207000
Fe	16.151401000	18.513368000	21.506919000
P	15.543669000	19.616348000	23.322564000
C	15.870685000	18.570113000	24.827340000
C	15.351862000	18.789512000	26.119255000
H	14.694046000	19.647744000	26.318232000
C	15.683927000	17.916195000	27.169896000
H	15.262244000	18.080606000	28.173796000
C	16.565300000	16.844877000	26.941671000
H	16.838824000	16.169181000	27.767036000
C	17.105770000	16.638233000	25.659543000
H	17.818131000	15.813475000	25.502727000
C	16.739606000	17.480734000	24.590555000
P	17.424977000	17.340702000	22.866254000
C	19.252200000	17.658060000	23.316215000
H	19.524521000	16.821032000	23.997871000
C	19.404712000	18.971915000	24.083433000
H	18.981287000	18.896558000	25.104180000
H	20.477361000	19.242738000	24.185507000
H	18.894334000	19.806376000	23.568381000
C	20.130702000	17.578660000	22.065325000
H	20.195093000	16.540814000	21.684812000
H	19.734979000	18.209314000	21.242713000
H	21.167899000	17.908829000	22.290092000
C	17.532075000	15.479794000	22.528787000
H	18.480620000	15.144859000	23.002577000
C	17.628416000	15.292600000	21.010876000
H	18.310417000	16.020896000	20.537957000
H	17.960782000	14.263298000	20.759523000
H	16.636372000	15.463209000	20.549667000
C	16.372072000	14.659081000	23.096195000
H	16.330334000	14.663196000	24.201584000
H	15.401818000	15.041255000	22.720333000
H	16.458776000	13.601857000	22.765439000
C	16.161809000	21.275444000	23.877565000
C	15.687006000	22.428832000	23.214011000
H	14.898131000	22.331268000	22.453235000
C	16.206774000	23.696074000	23.515814000
H	15.820912000	24.583061000	22.989121000
C	17.217399000	23.833887000	24.486084000
H	17.630646000	24.827354000	24.719678000
C	17.684648000	22.694237000	25.163932000
H	18.463019000	22.791486000	25.937141000
C	17.157497000	21.426209000	24.866511000
H	17.518255000	20.547231000	25.417333000
C	13.715692000	19.931375000	23.361705000
C	13.026504000	20.615802000	24.383148000
H	13.583662000	21.085432000	25.208037000
C	11.625900000	20.719761000	24.336405000
H	11.086733000	21.246728000	25.139033000
C	10.914878000	20.148483000	23.265483000
H	9.816492000	20.219620000	23.232499000
C	11.604398000	19.500049000	22.225209000
H	11.035238000	19.075334000	21.385343000
C	13.010814000	19.400256000	22.261638000
P	14.061447000	18.687226000	20.904838000
C	13.674393000	19.841549000	19.459311000
H	14.530088000	19.644279000	18.777835000
C	12.359638000	19.590747000	18.720363000
H	11.480780000	19.700643000	19.388231000
H	12.315108000	18.590531000	18.248425000
H	12.243092000	20.334525000	17.907792000
C	13.774756000	21.287905000	19.953904000
H	13.871778000	21.988417000	19.100772000
H	14.660178000	21.424157000	20.603614000
H	12.874885000	21.577874000	20.534526000
C	13.110831000	17.118115000	20.489916000
H	12.115763000	17.447229000	20.123008000
C	12.908457000	16.249062000	21.732073000
H	12.457244000	15.271623000	21.458940000
H	12.246260000	16.735400000	22.475361000
H	13.879537000	16.053217000	22.231495000
C	13.841779000	16.390818000	19.361746000
H	14.874034000	16.128701000	19.669490000
H	13.930546000	17.026024000	18.459834000
H	13.307922000	15.460843000	19.072796000
Fe	17.920567000	18.888467000	18.666856000
P	17.081368000	19.736443000	16.789933000
C	17.656637000	18.751759000	15.305122000
C	17.406168000	19.106352000	13.964761000
H	16.758929000	19.962595000	13.726592000
C	17.974814000	18.359991000	12.917889000
H	17.789905000	18.652799000	11.872574000
C	18.781863000	17.246275000	13.208194000
H	19.234690000	16.663375000	12.390851000
C	19.002733000	16.866869000	14.544458000
H	19.619391000	15.980026000	14.755413000
C	18.442979000	17.617212000	15.599257000
C	17.805326000	21.420085000	16.400080000
C	17.457526000	22.221339000	15.291927000
H	16.690762000	21.887693000	14.578273000
C	18.062988000	23.475318000	15.109774000
H	17.792232000	24.092372000	14.238807000
C	18.996737000	23.950141000	16.049033000
H	19.458063000	24.941431000	15.917995000
C	19.334254000	23.160041000	17.160625000
H	20.051737000	23.546385000	17.899857000
C	18.756846000	21.884036000	17.326878000
C	15.325473000	20.093184000	16.269623000
C	14.748744000	21.349887000	16.558726000
H	15.327121000	22.098954000	17.119472000

C	13.462328000	21.672442000	16.101040000
H	13.041458000	22.666623000	16.317878000
C	12.715446000	20.732877000	15.367248000
H	11.708106000	20.987147000	15.003299000
C	13.260843000	19.462473000	15.115544000
H	12.682476000	18.713558000	14.552080000
C	14.554741000	19.145633000	15.562326000
H	14.984418000	18.164511000	15.317411000
P	18.575468000	17.211616000	17.408355000
C	17.390835000	15.713316000	17.483403000
H	17.229886000	15.607012000	18.574010000
C	16.056425000	16.105578000	16.847747000
H	16.142820000	16.177088000	15.743515000
H	15.708571000	17.081387000	17.237292000
H	15.278086000	15.347805000	17.073179000
C	17.899333000	14.387068000	16.912385000
H	17.113593000	13.607609000	17.021509000
H	18.802183000	14.006148000	17.428860000
H	18.124943000	14.464518000	15.827900000
C	20.262777000	16.366978000	17.470833000
H	20.201365000	15.542753000	16.729488000
C	20.533294000	15.767704000	18.850302000
H	21.518031000	15.253684000	18.865786000
H	19.765300000	15.032484000	19.154989000
H	20.553791000	16.567298000	19.615673000
C	21.391343000	17.309414000	17.058335000
H	21.520169000	18.113700000	17.806670000
H	21.209759000	17.782425000	16.073528000
H	22.350848000	16.752650000	16.994510000
P	19.139665000	20.712345000	18.714692000
C	18.708215000	21.828801000	20.172103000
H	19.356097000	22.728478000	20.085674000
C	18.964771000	21.109114000	21.492904000
H	18.736382000	21.773423000	22.352559000
H	20.009715000	20.757905000	21.600866000
H	18.294177000	20.225669000	21.564759000
C	17.237049000	22.234002000	20.064185000
H	16.606829000	21.327001000	20.156216000
H	16.996286000	22.730740000	19.103009000
H	16.967886000	22.924741000	20.888345000
C	21.041544000	20.665419000	18.676323000
H	21.242795000	19.667636000	19.122477000
C	21.525540000	20.662181000	17.217935000
H	21.541174000	21.690863000	16.805202000
H	20.869856000	20.057573000	16.561419000
H	22.555578000	20.254565000	17.143331000
C	21.794195000	21.720155000	19.496283000
H	22.888553000	21.539209000	19.420395000
H	21.534766000	21.698806000	20.572051000
H	21.614043000	22.748714000	19.121880000
H	15.523871000	17.168440000	21.798150000
H	19.127306000	18.423118000	19.447239000

EP1’

N	16.394504000	18.200594000	19.553095000
Fe	16.087654000	18.505685000	21.320744000
P	15.578665000	19.690288000	23.112227000
C	15.917656000	18.655722000	24.627882000
C	15.397477000	18.892479000	25.916414000
H	14.723509000	19.741842000	26.096590000
C	15.742551000	18.049145000	26.984607000
H	15.323814000	18.232330000	27.986469000
C	16.632324000	16.979847000	26.774316000
H	16.915873000	16.323374000	27.611760000
C	17.165583000	16.752244000	25.495315000
H	17.882096000	15.929025000	25.350801000
C	16.793981000	17.571689000	24.408536000
P	17.500328000	17.406408000	22.692480000
C	19.324920000	17.670092000	23.247547000
H	19.517556000	16.807186000	23.924136000
C	19.489903000	18.955379000	24.060944000
H	19.010078000	18.873208000	25.055967000
H	20.567235000	19.172622000	24.227387000
H	19.050548000	19.826174000	23.542600000
C	20.287789000	17.576963000	22.066521000
H	20.299730000	16.551939000	21.648029000
H	19.993519000	18.262242000	21.242931000
H	21.326662000	17.817804000	22.380387000
C	17.573669000	15.527922000	22.412510000
H	18.531416000	15.190604000	22.865722000
C	17.617738000	15.314543000	20.899319000
H	18.346419000	15.986758000	20.412723000
H	17.859270000	14.260189000	20.647340000
H	16.632015000	15.576666000	20.467034000
C	16.419461000	14.737479000	23.031348000
H	16.421406000	14.743469000	24.136927000
H	15.445176000	15.145894000	22.696326000
H	16.464517000	13.677656000	22.699581000
C	16.176099000	21.351861000	23.686438000
C	15.662312000	22.508787000	23.061204000
H	14.877416000	22.410372000	22.296657000
C	16.139183000	23.782547000	23.406098000
H	15.724056000	24.673138000	22.908709000
C	17.145572000	23.921238000	24.379707000
H	17.524516000	24.919803000	24.647249000
C	17.654096000	22.776238000	25.017341000
H	18.431745000	22.873599000	25.791202000
C	17.168303000	21.502245000	24.677966000
H	17.561452000	20.617071000	25.195702000
C	13.745305000	19.965039000	23.261856000
C	13.106103000	20.697788000	24.282029000
H	13.701976000	21.262157000	25.015347000
C	11.703554000	20.721755000	24.355608000
H	11.205369000	21.289707000	25.156695000
C	10.938132000	20.011589000	23.412561000
H	9.839204000	20.010755000	23.481598000
C	11.572579000	19.319944000	22.366182000
H	10.959818000	18.787520000	21.624255000
C	12.980017000	19.315848000	22.271584000
P	13.964775000	18.661383000	20.845860000
C	13.434087000	19.867183000	19.481055000
H	14.149536000	19.644093000	18.659922000
C	12.002335000	19.726442000	18.961234000
H	11.255400000	19.892167000	19.764748000
H	11.802926000	18.742428000	18.493944000
H	11.816704000	20.493110000	18.182218000
C	13.709925000	21.293573000	19.963903000
H	13.575781000	22.015388000	19.133391000
H	14.748126000	21.393558000	20.333910000
H	13.017290000	21.588446000	20.779441000
C	13.000937000	17.101912000	20.412433000
H	11.970106000	17.452246000	20.195281000
C	12.934717000	16.131276000	21.591789000
H	12.270803000	15.273925000	21.351361000
H	12.548235000	16.613052000	22.512306000
H	13.938493000	15.725834000	21.825460000
C	13.562055000	16.431394000	19.155610000
H	14.592982000	16.066619000	19.335040000
H	13.590139000	17.118041000	18.285114000
H	12.940296000	15.557833000	18.865952000
Fe	17.862011000	18.847747000	18.752089000
P	17.062008000	19.710710000	16.880951000
C	17.702776000	18.772459000	15.395624000
C	17.432109000	19.114883000	14.056126000
H	16.734106000	19.932686000	13.823822000
C	18.045978000	18.409257000	13.007720000
H	17.838743000	18.687433000	11.962522000
C	18.927957000	17.352893000	13.297142000
H	19.418130000	16.801901000	12.479212000
C	19.175221000	16.988216000	14.632394000
H	19.849893000	16.143778000	14.838674000
C	18.563201000	17.693029000	15.690169000

C	17.800600000	21.388946000	16.503042000
C	17.439335000	22.211667000	15.415132000
H	16.641915000	21.903528000	14.723102000
C	18.077567000	23.446996000	15.221811000
H	17.792363000	24.084182000	14.370071000
C	19.070887000	23.874349000	16.122905000
H	19.563847000	24.848929000	15.981495000
C	19.427591000	23.058479000	17.208687000
H	20.192010000	23.409944000	17.917820000
C	18.806039000	21.805526000	17.394320000
C	15.311571000	20.043335000	16.339069000
C	14.700005000	21.275539000	16.664306000
H	15.274427000	22.038480000	17.211043000
C	13.380422000	21.548454000	16.275836000
H	12.933193000	22.526233000	16.513761000
C	12.628436000	20.576571000	15.590397000
H	11.590917000	20.788487000	15.289517000
C	13.209645000	19.330486000	15.302705000
H	12.629970000	18.559151000	14.771499000
C	14.542129000	19.069702000	15.665259000
H	14.996956000	18.108182000	15.390685000
P	18.654844000	17.249396000	17.497815000
C	17.480623000	15.726927000	17.465144000
H	17.282906000	15.574268000	18.542162000
C	16.157844000	16.094463000	16.790783000
H	16.276503000	16.191084000	15.691813000
H	15.750698000	17.048248000	17.174009000
H	15.395891000	15.308923000	16.976795000
C	18.031328000	14.428940000	16.866129000
H	17.252769000	13.636607000	16.920173000
H	18.919603000	14.042206000	17.402095000
H	18.292750000	14.545284000	15.793108000
C	20.335869000	16.377443000	17.552292000
H	20.262363000	15.600504000	16.764060000
C	20.580083000	15.684623000	18.891113000
H	21.559380000	15.159757000	18.882093000
H	19.802080000	14.937889000	19.137072000
H	20.601251000	16.430897000	19.706734000
C	21.501739000	17.297902000	17.201475000
H	21.690354000	18.021622000	18.016340000
H	21.330585000	17.866021000	16.267383000
H	22.428965000	16.700009000	17.068811000
P	19.151013000	20.634706000	18.799672000
C	18.752319000	21.833657000	20.207452000
H	19.459985000	22.682920000	20.097385000
C	18.942374000	21.157554000	21.558520000
H	18.760575000	21.868320000	22.390627000
H	19.956759000	20.731108000	21.682423000
H	18.214924000	20.321881000	21.655782000
C	17.317536000	22.336002000	20.047041000
H	16.622646000	21.470400000	20.051617000
H	17.166008000	22.897772000	19.104107000
H	17.044224000	22.999375000	20.891677000
C	21.054530000	20.550397000	18.776345000
H	21.231206000	19.540120000	19.203615000
C	21.549973000	20.563740000	17.322118000
H	21.578241000	21.597563000	16.924263000
H	20.894486000	19.974045000	16.651467000
H	22.577125000	20.148810000	17.250046000
C	21.830866000	21.569153000	19.619690000
H	22.920279000	21.359155000	19.545792000
H	21.566214000	21.536014000	20.693858000
H	21.682323000	22.609704000	19.264502000
H	15.439760000	17.294508000	21.930290000
H	18.831402000	18.341284000	19.812589000
H	15.614939000	17.960454000	18.919601000

EP11’

N	16.502272000	18.140785000	19.619071000
Fe	15.962932000	18.514317000	21.542053000
P	15.426483000	19.639627000	23.293985000
C	15.867544000	18.692640000	24.827035000
C	15.432718000	18.999502000	26.132199000
H	14.771531000	19.857147000	26.317291000
C	15.851503000	18.210933000	27.214242000
H	15.502906000	18.447498000	28.231101000
C	16.722478000	17.126341000	26.999766000
H	17.055090000	16.509235000	27.848431000
C	17.177970000	16.836114000	25.705030000
H	17.876784000	15.998885000	25.554929000
C	16.746588000	17.611753000	24.608482000
P	17.371149000	17.369864000	22.874781000
C	19.233148000	17.456347000	23.296665000
H	19.379998000	16.588735000	23.977551000
C	19.608300000	18.722409000	24.070415000
H	19.129019000	18.748682000	25.068550000
H	20.706848000	18.753533000	24.231288000
H	19.328019000	19.644547000	23.531775000
C	20.084802000	17.236743000	22.049464000
H	19.946669000	16.218190000	21.638160000
H	19.822026000	17.953812000	21.243401000
H	21.163789000	17.352236000	22.286740000
C	17.212470000	15.487695000	22.627966000
H	18.115864000	15.053076000	23.107611000
C	17.253771000	15.153080000	21.132333000
H	18.083693000	15.671120000	20.610779000
H	17.393439000	14.063529000	20.975421000
H	16.299259000	15.431479000	20.638894000
C	15.963698000	14.898991000	23.293785000
H	16.033509000	14.891619000	24.397215000
H	15.053263000	15.471257000	23.024401000
H	15.811833000	13.851002000	22.958776000
C	16.091047000	21.317123000	23.679286000
C	15.466973000	22.472109000	23.161257000
H	14.536472000	22.383320000	22.584466000
C	16.023960000	23.741031000	23.386700000
H	15.526815000	24.633893000	22.977555000
C	17.208498000	23.870722000	24.133161000
H	17.644825000	24.865996000	24.308475000
C	17.825669000	22.725356000	24.665930000
H	18.745793000	22.818831000	25.262999000
C	17.269204000	21.455901000	24.442273000
H	17.753833000	20.566706000	24.867364000
C	13.596673000	19.886714000	23.409401000
C	12.928456000	20.536415000	24.465535000
H	13.498267000	21.042089000	25.258562000
C	11.525096000	20.551342000	24.500736000
H	11.003363000	21.053498000	25.329602000
C	10.789121000	19.915770000	23.484388000
H	9.689229000	19.904571000	23.524994000
C	11.451945000	19.313714000	22.401826000
H	10.859932000	18.839619000	21.605922000
C	12.860803000	19.321067000	22.344149000
P	13.877246000	18.784805000	20.894518000
C	13.412994000	20.090617000	19.604494000
H	14.103881000	19.874508000	18.760045000
C	11.969503000	20.042945000	19.097282000
H	11.245209000	20.187032000	19.924427000
H	11.720610000	19.102974000	18.568597000
H	11.804323000	20.869205000	18.377261000
C	13.751465000	21.475193000	20.160624000
H	13.723117000	22.238782000	19.356447000
H	14.760182000	21.498150000	20.611053000
H	13.019316000	21.780042000	20.937101000
C	12.945983000	17.271455000	20.276727000
H	11.910380000	17.643350000	20.127507000
C	12.907401000	16.147313000	21.313165000
H	12.176278000	15.371060000	21.004175000
H	12.609464000	16.509665000	22.317661000
H	13.894776000	15.654823000	21.405310000
C	13.474368000	16.777959000	18.925202000
H	14.475147000	16.314533000	19.030027000

H	13.540802000	17.581785000	18.163520000
H	12.801713000	15.997075000	18.513094000
Fe	17.989242000	18.851078000	18.615449000
P	17.098491000	19.704028000	16.777401000
C	17.768518000	18.782714000	15.299692000
C	17.457053000	19.105172000	13.965186000
H	16.722147000	19.892772000	13.741373000
C	18.080629000	18.416375000	12.911716000
H	17.838797000	18.675310000	11.869480000
C	19.015264000	17.402154000	13.188038000
H	19.508290000	16.866110000	12.362593000
C	19.316851000	17.064447000	14.518414000
H	20.038937000	16.259392000	14.720962000
C	18.694422000	17.753197000	15.579552000
C	17.782038000	21.402579000	16.429463000
C	17.352085000	22.253434000	15.390897000
H	16.546410000	21.942489000	14.709458000
C	17.937820000	23.519164000	15.233501000
H	17.599025000	24.181491000	14.422095000
C	18.945562000	23.944966000	16.118619000
H	19.392330000	24.944861000	16.007158000
C	19.381811000	23.095226000	17.147584000
H	20.165566000	23.442085000	17.836616000
C	18.814489000	21.812817000	17.295700000
C	15.325884000	19.947626000	16.292883000
C	14.662952000	21.151075000	16.626366000
H	15.208955000	21.953310000	17.144652000
C	13.318403000	21.346598000	16.278385000
H	12.828138000	22.302549000	16.518062000
C	12.599600000	20.326617000	15.629727000
H	11.542199000	20.477865000	15.364429000
C	13.238118000	19.112959000	15.327141000
H	12.684493000	18.306807000	14.821412000
C	14.593922000	18.927357000	15.647339000
H	15.090885000	17.990425000	15.360160000
P	18.875599000	17.345232000	17.375505000
C	17.756790000	15.788609000	17.483196000
H	17.620336000	15.665907000	18.576208000
C	16.392029000	16.065501000	16.848058000
H	16.468981000	16.111186000	15.742609000
H	15.953294000	17.017982000	17.197152000
H	15.679305000	15.253620000	17.100695000
C	18.349988000	14.492793000	16.918878000
H	17.596989000	13.680421000	17.006089000
H	19.253399000	14.153593000	17.460742000
H	18.598274000	14.584894000	15.841051000
C	20.578612000	16.559254000	17.484625000
H	20.495710000	15.699887000	16.787841000
C	20.825981000	16.025803000	18.896188000
H	21.755499000	15.419700000	18.924237000
H	19.999096000	15.386107000	19.262949000
H	20.939982000	16.865094000	19.607197000
C	21.736054000	17.434324000	17.008720000
H	21.977600000	18.213102000	17.753646000
H	21.532969000	17.925709000	16.038091000
H	22.644587000	16.808557000	16.882980000
P	19.279924000	20.589406000	18.613049000
C	18.767593000	21.581945000	20.134820000
H	19.358934000	22.521144000	20.077533000
C	19.087389000	20.837033000	21.427663000
H	18.896447000	21.489440000	22.303614000
H	20.136279000	20.487679000	21.482355000
H	18.430367000	19.945674000	21.521837000
C	17.275879000	21.919489000	20.063956000
H	16.667439000	20.993226000	20.145205000
H	16.991593000	22.432332000	19.124749000
H	16.991844000	22.573953000	20.912597000
C	21.168210000	20.620017000	18.582825000
H	21.412837000	19.615537000	18.989871000
C	21.651308000	20.703376000	17.125403000
H	21.614086000	21.747206000	16.757006000
H	21.034604000	20.091755000	16.437406000
H	22.701827000	20.356538000	17.041900000
C	21.863123000	21.671866000	19.456811000
H	22.963835000	21.550868000	19.367287000
H	21.611439000	21.575547000	20.529945000
H	21.627419000	22.708419000	19.140860000
H	15.195603000	17.459884000	22.241805000
H	18.919693000	18.276577000	19.674783000
H	15.666752000	18.043109000	19.033135000
H	16.766920000	17.185565000	19.917539000

EP111’(H)

N	16.929269000	18.389254000	20.028414000
Fe	16.569159000	18.512195000	22.004838000
P	16.329602000	19.007424000	24.124852000
C	17.628363000	18.276658000	25.182175000
C	17.797287000	18.560818000	26.551421000
H	17.139698000	19.279552000	27.062698000
C	18.836705000	17.936571000	27.257519000
H	18.973823000	18.150785000	28.327820000
C	19.708792000	17.047733000	26.600489000
H	20.521775000	16.562272000	27.160623000
C	19.561915000	16.789975000	25.228357000
H	20.262933000	16.107444000	24.723847000
C	18.520954000	17.413090000	24.512377000
P	18.279524000	17.213353000	22.698196000
C	19.968471000	17.694034000	22.031173000
H	20.672274000	17.172722000	22.716184000
C	20.137184000	19.207852000	22.205192000
H	20.005053000	19.516430000	23.261499000
H	21.156783000	19.516363000	21.897313000
H	19.419232000	19.780180000	21.578098000
C	20.254018000	17.220591000	20.604273000
H	20.224387000	16.118363000	20.510203000
H	19.563818000	17.657166000	19.854705000
H	21.276335000	17.543697000	20.318771000
C	18.052142000	15.371535000	22.461861000
H	19.061576000	14.933376000	22.623592000
C	17.572667000	15.040613000	21.043072000
H	18.242098000	15.426614000	20.251155000
H	17.514903000	13.940800000	20.915825000
H	16.553025000	15.439607000	20.862072000
C	17.085085000	14.836131000	23.527247000
H	17.495412000	14.920710000	24.551248000
H	16.113928000	15.376021000	23.510743000
H	16.871046000	13.765579000	23.333157000
C	16.426147000	20.808653000	24.430405000
C	15.325401000	21.551098000	24.907631000
H	14.385327000	21.049370000	25.178577000
C	15.433955000	22.944841000	25.041605000
H	14.577467000	23.521382000	25.421905000
C	16.626976000	23.599832000	24.690185000
H	16.703946000	24.692897000	24.791160000
C	17.723918000	22.859894000	24.212711000
H	18.660636000	23.369062000	23.940904000
C	17.627027000	21.467443000	24.082955000
H	18.489694000	20.892719000	23.714950000
C	14.669397000	18.529448000	24.733764000
C	14.298321000	18.403745000	26.084034000
H	15.045458000	18.493510000	26.885011000
C	12.954142000	18.158656000	26.409223000
H	12.661220000	18.051208000	27.464085000
C	11.984865000	18.055969000	25.395391000
H	10.932004000	17.874323000	25.657257000
C	12.353085000	18.169209000	24.044942000
H	11.585938000	18.065851000	23.263987000
C	13.702272000	18.395183000	23.708587000
P	14.338019000	18.448161000	21.987804000
C	13.832782000	20.050317000	21.154697000
H	13.796080000	19.757194000	20.082647000
C	12.455248000	20.556762000	21.582030000

H	12.436963000	20.831903000	22.655802000
H	11.656424000	19.811425000	21.396138000
H	12.198979000	21.465402000	20.998050000
C	14.943706000	21.093772000	21.338135000
H	14.803257000	21.939313000	20.635329000
H	15.961147000	20.681140000	21.122969000
H	14.963856000	21.502321000	22.364802000
C	13.414668000	17.092807000	21.098237000
H	12.356316000	17.436836000	21.109773000
C	13.525462000	15.757492000	21.841063000
H	12.920291000	14.990956000	21.316071000
H	13.165904000	15.812094000	22.886378000
H	14.572034000	15.390039000	21.858327000
C	13.877357000	16.973352000	19.641165000
H	14.901280000	16.553612000	19.574304000
H	13.849515000	17.937088000	19.094469000
H	13.207740000	16.276606000	19.098171000
H	16.058439000	17.135420000	22.069457000
H	16.187688000	18.780131000	19.426262000
H	17.079697000	17.430366000	19.680747000
H	17.779921000	18.914928000	19.764038000

DP111’(H)

N	16.871812000	18.247347000	19.948882000
Fe	16.643543000	18.600405000	21.954141000
P	16.424063000	19.241291000	24.118031000
C	17.659232000	18.384912000	25.145053000
C	17.913541000	18.701407000	26.492799000
H	17.407143000	19.547676000	26.979434000
C	18.840496000	17.929584000	27.210190000
H	19.043307000	18.168108000	28.264460000
C	19.514537000	16.861349000	26.589726000
H	20.242623000	16.265973000	27.159816000
C	19.281416000	16.555884000	25.239387000
H	19.837226000	15.736665000	24.759457000
C	18.346814000	17.324636000	24.519354000
P	18.107258000	17.118611000	22.726531000
C	19.626232000	17.916073000	21.983242000
H	20.413763000	17.687862000	22.734035000
C	19.346980000	19.432171000	22.018280000
H	19.290872000	19.826678000	23.047301000
H	20.144806000	19.993028000	21.489807000
H	18.423463000	19.751070000	21.455225000
C	20.083648000	17.380971000	20.629128000
H	20.308581000	16.298420000	20.667662000
H	19.368609000	17.569246000	19.806100000
H	21.024968000	17.901361000	20.357906000
C	17.939639000	15.312651000	22.377488000
H	18.971491000	14.926981000	22.544571000
C	17.530498000	15.034921000	20.926570000
H	18.220866000	15.477874000	20.184799000
H	17.527773000	13.940865000	20.754616000
H	16.498904000	15.386375000	20.716595000
C	16.978441000	14.691978000	23.401196000
H	17.386776000	14.705128000	24.428254000
H	15.998989000	15.212747000	23.424158000
H	16.782852000	13.637229000	23.123946000
C	16.549353000	21.007897000	24.525236000
C	15.377084000	21.789482000	24.652055000
H	14.381281000	21.330121000	24.566716000
C	15.483954000	23.162441000	24.915000000
H	14.571291000	23.766769000	25.022225000
C	16.750272000	23.758938000	25.047974000
H	16.829451000	24.836992000	25.252952000
C	17.915721000	22.980377000	24.932087000
H	18.905967000	23.443212000	25.054501000
C	17.821451000	21.606782000	24.669147000
H	18.740211000	21.004957000	24.609270000
C	14.770349000	18.730392000	24.705857000
C	14.377464000	18.802932000	26.053493000
H	15.077204000	19.147145000	26.828190000
C	13.070274000	18.433803000	26.408104000
H	12.760439000	18.483659000	27.462081000
C	12.158311000	18.005501000	25.426601000
H	11.134072000	17.724469000	25.711541000
C	12.543024000	17.929485000	24.079539000
H	11.816187000	17.591024000	23.327770000
C	13.858230000	18.289507000	23.720652000
P	14.462574000	18.268449000	22.010029000
C	14.073875000	19.876426000	21.128634000
H	13.996778000	19.558087000	20.067202000
C	12.735980000	20.455616000	21.585411000
H	12.755981000	20.753153000	22.652244000
H	11.896071000	19.751823000	21.425368000
H	12.525323000	21.364694000	20.983696000
C	15.234372000	20.878059000	21.265052000
H	15.123460000	21.680665000	20.507713000
H	16.256618000	20.472097000	21.012222000
H	15.285399000	21.356526000	22.257075000
C	13.560910000	16.915761000	21.122629000
H	12.519203000	17.310584000	21.196025000
C	13.640940000	15.569627000	21.847574000
H	12.881029000	14.887716000	21.417185000
H	13.445031000	15.641984000	22.933960000
H	14.626048000	15.087306000	21.697984000
C	13.925747000	16.807858000	19.639269000
H	14.926675000	16.356860000	19.491665000
H	13.875910000	17.774646000	19.101231000
H	13.199025000	16.132866000	19.145892000
H	16.138187000	17.214351000	22.101273000
H	16.067556000	18.546754000	19.373816000
H	17.035662000	17.264010000	19.684761000
H	17.668094000	18.779957000	19.560094000

N_2-Fe-$(PR_3)_3^{+1}$ half-complex

Fe	16.530403000	17.991595000	21.974642000
P	16.400188000	18.927331000	23.886196000
C	17.632533000	18.152547000	25.028739000
C	17.768023000	18.500330000	26.386321000
H	17.143693000	19.289430000	26.830882000
C	18.728955000	17.850689000	27.178346000
H	18.825973000	18.115981000	28.241808000
C	19.572137000	16.874916000	26.616523000
H	20.322931000	16.370122000	27.242818000
C	19.479560000	16.563262000	25.250285000
H	20.172168000	15.830985000	24.807890000
C	18.511776000	17.201691000	24.451630000
P	18.407055000	17.049642000	22.627339000
C	20.132075000	17.480505000	22.046820000
H	20.792203000	16.750992000	22.567826000
C	20.504904000	18.900849000	22.481017000
H	20.359984000	19.063683000	23.567529000
H	21.572355000	19.098638000	22.252844000
H	19.907469000	19.651547000	21.924791000
C	20.274208000	17.292317000	20.531561000
H	20.124547000	16.241879000	20.214143000
H	19.562944000	17.934149000	19.971837000
H	21.298028000	17.583329000	20.217268000
C	18.100234000	15.297343000	22.048475000
H	19.062503000	14.803715000	21.798248000
C	17.276752000	15.538808000	20.764215000
H	17.867389000	15.979470000	19.941070000
H	16.801915000	14.607869000	20.389259000
H	16.391553000	16.219132000	20.976051000
C	17.353377000	14.462093000	23.091470000
H	17.976299000	14.268859000	23.986697000
H	16.423844000	14.969927000	23.424579000
H	17.067518000	13.481583000	22.656965000
C	16.676881000	20.730862000	24.091222000
C	15.737203000	21.580496000	24.711046000
H	14.803485000	21.173753000	25.124311000
C	15.989372000	22.959811000	24.795700000

H	15.251963000	23.616765000	25.281552000
C	17.170580000	23.498823000	24.259074000
H	17.361320000	24.580972000	24.322487000
C	18.108179000	22.653783000	23.637943000
H	19.034511000	23.069390000	23.213284000
C	17.861919000	21.276429000	23.552627000
H	18.593385000	20.620385000	23.062411000
C	14.699802000	18.658627000	24.550583000
C	14.336264000	18.728247000	25.907604000
H	15.091064000	18.909207000	26.684999000
C	12.992023000	18.556903000	26.278729000
H	12.711339000	18.601582000	27.341678000
C	12.012234000	18.325653000	25.298715000
H	10.960336000	18.189928000	25.591810000
C	12.371434000	18.251662000	23.942260000
H	11.596730000	18.044368000	23.189817000
C	13.716920000	18.412746000	23.558609000
P	14.329413000	18.259037000	21.821364000
C	13.689082000	19.779228000	20.922715000
H	14.167369000	19.687985000	19.921883000
C	12.167039000	19.827278000	20.753462000
H	11.652374000	19.899181000	21.733325000
H	11.761151000	18.952201000	20.209340000
H	11.888245000	20.730845000	20.171982000
C	14.218156000	21.048260000	21.600498000
H	13.949352000	21.937515000	20.993825000
H	15.317841000	21.046327000	21.715976000
H	13.769439000	21.184597000	22.605404000
C	13.348551000	16.825599000	21.119774000
H	12.282675000	17.114470000	21.243623000
C	13.593740000	15.535238000	21.908699000
H	12.897887000	14.745320000	21.557886000
H	13.427666000	15.669664000	22.996231000
H	14.623823000	15.156537000	21.765832000
C	13.647101000	16.671756000	19.622473000
H	14.715422000	16.432866000	19.439904000
H	13.404349000	17.585562000	19.044389000
H	13.045570000	15.840777000	19.199982000
H	16.045695000	16.918010000	22.936536000
N	17.096063000	19.277451000	20.854627000
N	17.453811000	20.105313000	20.160677000

N_2-Fe-$(PR_3)_3^{+2}$ half-complex

Fe	16.553901000	18.298202000	21.865969000
P	16.421117000	19.052984000	23.907532000
C	17.673985000	18.223103000	24.948839000
C	17.910739000	18.596790000	26.286399000
H	17.361166000	19.431749000	26.744914000
C	18.880742000	17.906223000	27.028624000
H	19.065519000	18.189078000	28.075388000
C	19.624511000	16.868256000	26.437478000
H	20.386955000	16.335896000	27.024963000
C	19.419378000	16.522582000	25.092643000
H	20.031998000	15.734133000	24.630795000
C	18.439580000	17.202514000	24.343613000
P	18.224459000	16.951322000	22.548313000
C	19.894621000	17.434062000	21.827010000
H	20.562879000	16.661506000	22.270903000
C	20.341926000	18.813886000	22.313074000
H	20.313324000	18.909479000	23.415856000
H	21.390928000	18.982178000	21.995641000
H	19.740624000	19.627904000	21.862921000
C	19.920120000	17.311984000	20.300290000
H	19.776680000	16.271192000	19.955624000
H	19.153707000	17.953015000	19.816131000
H	20.909329000	17.646689000	19.927051000
C	18.001796000	15.126311000	22.247027000
H	19.030696000	14.705001000	22.271134000
C	17.393107000	14.892970000	20.857148000
H	17.974742000	15.357451000	20.038968000
H	17.338112000	13.804921000	20.652592000
H	16.358903000	15.285027000	20.802693000
C	17.163757000	14.499569000	23.368308000
H	17.702049000	14.478530000	24.334146000
H	16.209506000	15.042461000	23.531495000
H	16.907891000	13.455362000	23.097321000
C	16.689125000	20.829049000	24.166979000
C	15.647700000	21.687079000	24.582186000
H	14.644230000	21.291006000	24.791539000
C	15.903284000	23.057679000	24.737569000
H	15.094513000	23.725727000	25.068682000
C	17.183484000	23.573456000	24.471595000
H	17.375794000	24.650576000	24.588126000
C	18.222720000	22.716569000	24.062159000
H	19.227334000	23.117429000	23.862427000
C	17.980238000	21.346094000	23.911268000
H	18.796749000	20.677731000	23.602554000
C	14.740386000	18.688007000	24.541700000
C	14.403898000	18.736526000	25.906674000
H	15.165964000	18.938048000	26.672187000
C	13.071255000	18.522777000	26.294634000
H	12.806897000	18.554405000	27.361795000
C	12.080867000	18.266803000	25.330614000
H	11.039433000	18.099711000	25.642477000
C	12.412795000	18.211055000	23.967195000
H	11.631618000	17.988337000	23.225980000
C	13.746782000	18.420661000	23.568059000
P	14.309204000	18.334639000	21.834164000
C	13.667402000	19.853733000	20.925422000
H	14.228753000	19.832129000	19.965708000
C	12.163580000	19.757076000	20.638198000
H	11.568352000	19.692616000	21.571250000
H	11.898741000	18.903557000	19.986061000
H	11.848830000	20.681689000	20.111616000
C	14.018013000	21.131034000	21.691404000
H	13.708463000	22.011106000	21.092798000
H	15.099362000	21.242024000	21.891686000
H	13.479329000	21.179125000	22.658199000
C	13.503133000	16.857357000	21.046035000
H	12.423887000	17.117771000	21.010453000
C	13.666649000	15.587280000	21.882418000
H	13.094442000	14.764211000	21.408557000
H	13.288592000	15.707248000	22.916300000
H	14.721911000	15.258554000	21.939666000
C	14.038270000	16.726588000	19.612535000
H	15.136398000	16.560894000	19.596090000
H	13.824535000	17.622076000	18.996094000
H	13.567491000	15.856335000	19.112898000
H	15.978125000	17.050248000	22.490661000
N	17.192533000	19.845381000	21.024288000
N	17.544445000	20.772894000	20.493251000

^{TS}CP222

N	16.284667000	18.149572000	19.491786000
N	15.162801000	17.326575000	18.547625000
Fe	15.953901000	18.492819000	21.216750000
P	15.932967000	19.567353000	23.187295000
C	15.921269000	18.275345000	24.522445000
C	15.550285000	18.500183000	25.863153000
H	15.149045000	19.474389000	26.174725000
C	15.703851000	17.482938000	26.817139000
H	15.400724000	17.659746000	27.860501000
C	16.262926000	16.247143000	26.442539000
H	16.403071000	15.453601000	27.192557000
C	16.639623000	16.022744000	25.109498000
H	17.073724000	15.050170000	24.830282000
C	16.450734000	17.025710000	24.134750000
P	16.871826000	16.784949000	22.337416000
C	18.696633000	16.270876000	22.557903000
H	18.677714000	15.471616000	23.330806000
C	19.515326000	17.447242000	23.085599000
H	19.265475000	17.663692000	24.140590000

H	20.600868000	17.218692000	23.048304000
H	19.340940000	18.366969000	22.489836000
C	19.270241000	15.690637000	21.267635000
H	18.787426000	14.735481000	20.983425000
H	19.141171000	16.410192000	20.440065000
H	20.355723000	15.488916000	21.381805000
C	16.124814000	15.059426000	22.016181000
H	16.865342000	14.353584000	22.451008000
C	15.994570000	14.778728000	20.514575000
H	15.840613000	13.695295000	20.332036000
H	15.108835000	15.306422000	20.112820000
H	16.877428000	15.097311000	19.930679000
C	14.767160000	14.824080000	22.684412000
H	14.791725000	14.932288000	23.784361000
H	14.009458000	15.529858000	22.291487000
H	14.410632000	13.797724000	22.454194000
C	17.144702000	20.750159000	23.976389000
C	16.965906000	22.147301000	23.899266000
H	16.089216000	22.562579000	23.386244000
C	17.891808000	23.023681000	24.488307000
H	17.729525000	24.110460000	24.418092000
C	19.016961000	22.518199000	25.160954000
H	19.744857000	23.205250000	25.619134000
C	19.195983000	21.127148000	25.257336000
H	20.061371000	20.715635000	25.799677000
C	18.261786000	20.254536000	24.678494000
H	18.395384000	19.172334000	24.793327000
C	14.391478000	20.580408000	23.403680000
C	14.068722000	21.319579000	24.561572000
H	14.742803000	21.321573000	25.430046000
C	12.901004000	22.095699000	24.603244000
H	12.658324000	22.671291000	25.509486000
C	12.051677000	22.144793000	23.483684000
H	11.138643000	22.759258000	23.508028000
C	12.361031000	21.403125000	22.333146000
H	11.668999000	21.429155000	21.481526000
C	13.534690000	20.621342000	22.283384000
P	13.943557000	19.409694000	20.940426000
C	13.515736000	20.231982000	19.295252000
H	13.843587000	19.454310000	18.574116000
C	12.054439000	20.564625000	18.977842000
H	11.972791000	20.863610000	17.911285000
H	11.684785000	21.426571000	19.566838000
H	11.359843000	19.716408000	19.131728000
C	14.437201000	21.436343000	19.111976000
H	14.244010000	21.949746000	18.146358000
H	15.494192000	21.114116000	19.128430000
H	14.284417000	22.186597000	19.913885000
C	12.433885000	18.293070000	21.240079000
H	11.561674000	18.961508000	21.081239000
C	12.403173000	17.804727000	22.692774000
H	11.617715000	17.031623000	22.824536000
H	12.187411000	18.628135000	23.399959000
H	13.373115000	17.358434000	22.991635000
C	12.338862000	17.142203000	20.236537000
H	13.172033000	16.418686000	20.361982000
H	12.310616000	17.489658000	19.181657000
H	11.404470000	16.564787000	20.395594000
Fe	17.633177000	18.967058000	18.794985000
P	17.058867000	19.462413000	16.732186000
C	18.322876000	18.810899000	15.538901000
C	18.288180000	19.080758000	14.157848000
H	17.511819000	19.741167000	13.742408000
C	19.238754000	18.498713000	13.304840000
H	19.218823000	18.716671000	12.226022000
C	20.200897000	17.619788000	13.832639000
H	20.930962000	17.135433000	13.166033000
C	20.234042000	17.349910000	15.211350000
H	20.981367000	16.640763000	15.591650000
C	19.319124000	17.968314000	16.088190000
C	17.025297000	21.298798000	16.466843000
C	16.421663000	21.960391000	15.380944000
H	15.934011000	21.385342000	14.578948000
C	16.412559000	23.365537000	15.335917000
H	15.923727000	23.883171000	14.496229000
C	17.017283000	24.106866000	16.366377000
H	16.998468000	25.207279000	16.337126000
C	17.651504000	23.447165000	17.434751000
H	18.134000000	24.041231000	18.225691000
C	17.655239000	22.040455000	17.493193000
C	15.494440000	18.930640000	15.874169000
C	14.290809000	19.635663000	16.113845000
H	14.321236000	20.584979000	16.665577000
C	13.063261000	19.145181000	15.640346000
H	12.139256000	19.710998000	15.833122000
C	13.013705000	17.939991000	14.918209000
H	12.050081000	17.551382000	14.555452000
C	14.205529000	17.247294000	14.643449000
H	14.182154000	16.316923000	14.055616000
C	15.434848000	17.739867000	15.109890000
H	16.356244000	17.192008000	14.872585000
P	19.210408000	17.695745000	17.935657000
C	18.666384000	15.856269000	17.919466000
H	18.178976000	15.774609000	18.908728000
C	17.593584000	15.629527000	16.852353000
H	18.037438000	15.518900000	15.842468000
H	16.885728000	16.474906000	16.796268000
H	17.015791000	14.704846000	17.064804000
C	19.759185000	14.791205000	17.817090000
H	19.293846000	13.782439000	17.792886000
H	20.460688000	14.798129000	18.673509000
H	20.353596000	14.895343000	16.885929000
C	21.016359000	17.619515000	18.591633000
H	21.189721000	16.538565000	18.751713000
C	21.123077000	18.298032000	19.966361000
H	20.208026000	18.169906000	20.568052000
H	21.290628000	19.388278000	19.876259000
H	21.985133000	17.880343000	20.526258000
C	22.115045000	18.129786000	17.646256000
H	21.874510000	19.114978000	17.201656000
H	22.328506000	17.433021000	16.816258000
H	23.060205000	18.249529000	18.217618000
P	18.548491000	21.006034000	18.750876000
C	18.526047000	22.046628000	20.313206000
H	19.081384000	22.969817000	20.051765000
C	19.276849000	21.311612000	21.426294000
H	19.337802000	21.939121000	22.337454000
H	20.310841000	21.043197000	21.130596000
H	18.743742000	20.374591000	21.682809000
C	17.116507000	22.453170000	20.727010000
H	16.531776000	21.576232000	21.072481000
H	16.567686000	22.936247000	19.898292000
H	17.170492000	23.183064000	21.556553000
C	20.340781000	21.330750000	18.127732000
H	20.886282000	20.455613000	18.526780000
C	20.427143000	21.287609000	16.598591000
H	19.969779000	22.187864000	16.140122000
H	19.930445000	20.402685000	16.166222000
H	21.491264000	21.260557000	16.281622000
C	21.022872000	22.600600000	18.646714000
H	22.044759000	22.665505000	18.214721000
H	21.138142000	22.622658000	19.747096000
H	20.484097000	23.518162000	18.330761000
H	14.964509000	17.453820000	21.623642000
H	18.269803000	18.914336000	20.205859000
H	15.563184000	16.393656000	18.380452000
H	15.033690000	17.804846000	17.637913000
H	14.264407000	17.218239000	19.043376000

CP11’

N	16.062993000	19.145121000	19.314589000
Fe	16.021795000	19.172097000	21.282094000

P	15.695716000	19.817353000	23.419815000
C	15.549877000	18.314234000	24.455849000
C	14.945633000	18.273126000	25.726159000
H	14.463358000	19.165171000	26.149853000
C	14.961014000	17.075394000	26.456530000
H	14.481564000	17.034424000	27.445740000
C	15.595179000	15.934226000	25.931883000
H	15.617696000	15.000706000	26.513496000
C	16.200605000	15.975927000	24.666213000
H	16.701336000	15.076097000	24.279280000
C	16.157482000	17.164186000	23.909901000
P	16.969308000	17.364226000	22.266184000
C	18.743112000	16.964832000	22.808549000
H	18.576284000	15.951411000	23.238135000
C	19.253906000	17.881610000	23.915610000
H	18.632989000	17.807345000	24.826657000
H	20.283913000	17.579949000	24.195975000
H	19.296262000	18.942183000	23.601100000
C	19.731530000	16.839676000	21.664101000
H	19.387075000	16.155933000	20.864154000
H	19.937853000	17.826416000	21.209353000
H	20.691399000	16.440710000	22.050031000
C	16.550156000	15.803882000	21.286234000
H	17.363390000	15.092455000	21.537839000
C	16.593952000	16.089817000	19.787382000
H	17.532962000	16.649739000	19.516749000
H	16.628716000	15.143284000	19.220868000
H	15.702555000	16.640283000	19.444492000
C	15.204125000	15.166860000	21.643598000
H	15.174244000	14.771130000	22.673750000
H	14.368282000	15.883765000	21.534254000
H	15.004902000	14.322651000	20.952516000
C	16.860054000	20.922233000	24.306818000
C	16.863984000	22.295769000	23.974148000
H	16.151685000	22.691130000	23.236096000
C	17.748137000	23.173345000	24.616642000
H	17.743503000	24.241303000	24.352072000
C	18.625619000	22.690079000	25.604462000
H	19.320827000	23.378834000	26.107664000
C	18.593428000	21.333395000	25.967805000
H	19.251031000	20.956755000	26.765479000
C	17.711127000	20.450090000	25.325153000
H	17.670396000	19.399691000	25.640438000
C	14.112344000	20.711022000	23.651852000
C	13.751780000	21.362085000	24.849022000
H	14.468660000	21.450025000	25.678526000
C	12.467784000	21.913772000	24.968095000
H	12.178997000	22.424827000	25.898465000
C	11.550504000	21.815206000	23.904575000
H	10.541869000	22.242048000	24.007663000
C	11.918882000	21.191590000	22.702771000
H	11.194222000	21.137488000	21.877624000
C	13.215167000	20.653096000	22.571452000
P	13.862332000	19.899826000	21.033804000
C	13.590961000	21.267676000	19.743047000
H	14.386703000	21.092978000	18.986819000
C	12.243265000	21.198863000	19.017720000
H	11.396426000	21.391445000	19.705387000
H	12.066502000	20.230788000	18.514380000
H	12.215184000	21.989316000	18.239330000
C	13.823544000	22.650974000	20.368904000
H	14.007317000	23.395644000	19.568399000
H	14.683867000	22.681193000	21.062643000
H	12.935901000	22.982339000	20.940962000
C	12.604361000	18.553984000	20.696330000
H	11.672437000	19.109462000	20.460392000
C	12.367328000	17.727674000	21.966162000
H	11.702471000	16.871121000	21.734340000
H	11.889842000	18.320877000	22.768243000
H	13.309769000	17.312650000	22.376983000
C	12.981590000	17.694540000	19.488568000
H	13.858903000	17.054307000	19.705413000
H	13.193625000	18.294585000	18.582768000
H	12.147262000	17.011784000	19.233063000
Fe	17.840399000	18.524338000	18.779722000
P	17.015295000	18.916704000	16.712498000
C	18.018394000	18.166359000	15.389118000
C	17.658473000	18.232526000	14.029473000
H	16.719922000	18.715527000	13.720406000
C	18.506479000	17.667379000	13.066140000
H	18.234758000	17.718738000	12.001585000
C	19.699487000	17.036286000	13.461262000
H	20.363164000	16.591378000	12.705195000
C	20.050358000	16.955685000	14.818600000
H	20.980561000	16.442120000	15.096949000
C	19.207107000	17.521669000	15.797198000
C	17.019577000	20.743018000	16.480521000
C	16.448926000	21.382636000	15.362093000
H	15.995165000	20.797380000	14.549494000
C	16.441453000	22.783988000	15.294772000
H	15.992158000	23.283367000	14.423879000
C	16.992274000	23.547848000	16.339891000
H	16.969455000	24.646455000	16.290055000
C	17.588223000	22.916201000	17.442756000
H	18.041316000	23.528201000	18.236127000
C	17.609581000	21.508174000	17.512911000
C	15.279511000	18.452391000	16.336617000
C	14.232063000	19.399406000	16.409762000
H	14.439278000	20.450463000	16.654987000
C	12.914404000	19.013643000	16.114853000
H	12.111410000	19.764508000	16.138121000
C	12.623582000	17.680187000	15.783038000
H	11.589046000	17.382190000	15.557171000
C	13.658143000	16.730202000	15.733313000
H	13.440295000	15.685402000	15.466996000
C	14.980822000	17.113407000	15.999051000
H	15.783020000	16.368734000	15.909133000
P	19.497615000	17.340793000	17.611127000
C	19.163997000	15.468626000	17.858689000
H	18.951800000	15.382102000	18.943041000
C	17.925759000	15.069129000	17.050813000
H	18.153201000	15.027932000	15.967409000
H	17.082091000	15.768533000	17.190885000
H	17.579366000	14.062127000	17.358800000
C	20.330082000	14.526540000	17.543517000
H	19.968629000	13.485647000	17.677755000
H	21.194854000	14.652844000	18.221594000
H	20.674552000	14.611334000	16.493025000
C	21.389111000	17.383043000	17.751666000
H	21.587601000	16.390756000	17.303175000
C	21.854869000	17.327148000	19.204228000
H	22.953241000	17.176625000	19.229727000
H	21.395367000	16.491707000	19.762473000
H	21.641424000	18.271755000	19.745324000
C	22.209289000	18.374094000	16.918797000
H	22.389731000	19.329948000	17.441199000
H	21.778294000	18.585957000	15.924563000
H	23.205032000	17.916441000	16.747846000
P	18.624493000	20.556012000	18.716310000
C	18.536910000	21.513875000	20.313775000
H	19.107436000	22.438489000	20.089045000
C	19.233236000	20.776408000	21.455770000
H	19.324743000	21.438770000	22.339152000
H	20.250576000	20.430608000	21.187052000
H	18.636405000	19.887650000	21.751637000
C	17.118530000	21.942265000	20.651516000
H	16.508154000	21.141054000	21.152585000
H	16.540538000	22.287916000	19.776618000
H	17.135244000	22.764070000	21.389867000
C	20.356470000	20.866621000	18.053063000
H	20.913851000	19.976188000	18.400694000
C	20.342715000	20.917805000	16.519519000

H	19.811917000	21.819460000	16.158536000
H	19.868481000	20.035966000	16.049267000
H	21.381777000	20.977318000	16.144061000
C	21.034973000	22.116448000	18.625117000
H	22.042787000	22.204165000	18.169385000
H	21.178575000	22.075269000	19.721094000
H	20.483686000	23.043346000	18.366733000
H	15.133245000	18.005327000	21.393829000
H	19.082859000	18.366578000	19.650495000
H	15.333228000	18.528285000	18.921038000
H	15.832231000	20.075028000	18.929098000

EP11222(H)

N	16.824214000	17.975391000	19.357868000
N	15.582676000	17.534471000	18.693011000
Fe	18.253156000	18.941134000	18.305131000
P	17.494081000	19.412776000	16.356976000
C	18.505468000	18.602181000	15.019909000
C	18.391830000	18.871498000	13.643265000
H	17.630608000	19.575226000	13.273860000
C	19.261284000	18.248051000	12.730159000
H	19.178757000	18.469586000	11.654878000
C	20.238734000	17.349828000	13.192352000
H	20.923920000	16.866122000	12.478963000
C	20.345796000	17.065754000	14.566357000
H	21.115374000	16.357892000	14.910742000
C	19.485921000	17.690860000	15.490552000
C	17.561191000	21.234417000	15.995947000
C	16.935265000	21.873801000	14.909009000
H	16.382933000	21.285456000	14.159463000
C	16.987215000	23.274152000	14.798846000
H	16.493653000	23.777845000	13.953257000
C	17.650663000	24.031186000	15.781449000
H	17.673882000	25.129510000	15.707145000
C	18.276697000	23.391423000	16.866767000
H	18.775473000	24.000746000	17.635310000
C	18.243298000	21.986746000	16.977709000
C	15.733287000	19.061731000	15.879708000
C	14.739645000	19.740168000	16.634916000
H	15.037943000	20.563598000	17.306363000
C	13.386934000	19.376068000	16.533337000
H	12.629010000	19.915794000	17.121952000
C	13.003582000	18.320800000	15.681416000
H	11.946042000	18.024954000	15.609473000
C	13.977295000	17.657852000	14.916251000
H	13.682624000	16.842032000	14.237977000
C	15.332640000	18.027756000	15.008524000
H	16.082952000	17.499172000	14.402121000
P	19.481453000	17.417819000	17.334577000
C	18.657320000	15.676518000	17.414662000
H	18.373469000	15.596510000	18.488461000
C	17.380577000	15.674849000	16.566145000
H	17.611226000	15.494563000	15.496472000
H	16.869329000	16.659329000	16.582140000
H	16.668712000	14.885949000	16.889619000
C	19.522588000	14.464349000	17.057046000
H	18.915980000	13.534551000	17.117423000
H	20.386306000	14.330659000	17.736222000
H	19.905679000	14.532441000	16.017356000
C	21.263761000	16.954925000	17.747843000
H	21.488890000	16.036643000	17.166529000
C	21.345450000	16.626421000	19.243757000
H	22.366605000	16.282101000	19.512779000
H	20.635062000	15.830484000	19.549496000
H	21.110525000	17.526540000	19.849045000
C	22.285312000	18.023093000	17.357826000
H	22.145919000	18.947010000	17.950544000
H	22.229077000	18.289533000	16.284780000
H	23.313458000	17.652737000	17.557571000
P	19.048889000	20.966235000	18.318630000
C	18.629467000	21.969220000	19.860485000
H	18.959871000	23.012740000	19.668491000
C	19.368735000	21.424191000	21.086244000
H	19.139039000	22.035920000	21.984670000
H	20.470044000	21.424671000	20.955177000
H	19.062452000	20.377288000	21.290963000
C	17.111102000	21.959627000	20.062411000
H	16.743965000	20.924284000	20.232317000
H	16.572369000	22.364391000	19.181657000
H	16.824023000	22.566750000	20.947050000
C	20.875842000	21.343376000	17.991860000
H	21.382881000	20.566209000	18.603961000
C	21.163648000	21.076743000	16.508696000
H	20.768135000	21.899109000	15.877808000
H	20.697644000	20.134144000	16.159548000
H	22.255983000	21.007945000	16.325579000
C	21.391798000	22.721030000	18.418002000
H	22.479780000	22.795743000	18.202648000
H	21.262438000	22.917995000	19.500173000
H	20.897134000	23.539109000	17.855469000
H	19.115933000	18.784101000	19.587296000
H	15.174802000	18.343856000	18.161262000
H	14.866247000	17.139032000	19.335593000
H	15.836875000	16.818994000	17.981203000
H	17.183624000	17.138221000	19.844319000
H	16.522574000	18.621366000	20.107490000

CP1’

N	16.780311000	18.504329000	19.945866000
Fe	16.173442000	18.828207000	21.573699000
P	15.476744000	19.714532000	23.523628000
C	15.861218000	18.561243000	24.906534000
C	15.328903000	18.650604000	26.208521000
H	14.565799000	19.401995000	26.453779000
C	15.778588000	17.773682000	27.206333000
H	15.357786000	17.840311000	28.220740000
C	16.772655000	16.820662000	26.915537000
H	17.134521000	16.143721000	27.703867000
C	17.303068000	16.727338000	25.620539000
H	18.080319000	15.978558000	25.404661000
C	16.836182000	17.585937000	24.603614000
P	17.493091000	17.535929000	22.878852000
C	19.335335000	17.801044000	23.222201000
H	19.612172000	16.894417000	23.804098000
C	19.594567000	19.021650000	24.108358000
H	19.182311000	18.877224000	25.125462000
H	20.688145000	19.179761000	24.213500000
H	19.164548000	19.953920000	23.699313000
C	20.141448000	17.790032000	21.921471000
H	20.119483000	16.793670000	21.443037000
H	19.754239000	18.517865000	21.183491000
H	21.205171000	18.033628000	22.121966000
C	17.483456000	15.695446000	22.446295000
H	18.421592000	15.308972000	22.899307000
C	17.554837000	15.502843000	20.928679000
H	18.293616000	16.167951000	20.446928000
H	17.818230000	14.452044000	20.692290000
H	16.568127000	15.710576000	20.468246000
C	16.293849000	14.931155000	23.032799000
H	16.301380000	14.889491000	24.136974000
H	15.332206000	15.384455000	22.720930000
H	16.302566000	13.887532000	22.655099000
C	16.153834000	21.322163000	24.089532000
C	15.684163000	22.506889000	23.481524000
H	14.851271000	22.464710000	22.765055000
C	16.259004000	23.743898000	23.807644000
H	15.883439000	24.661651000	23.330405000
C	17.306237000	23.808419000	24.744799000
H	17.758947000	24.778631000	24.999303000
C	17.758711000	22.634533000	25.371037000
H	18.560648000	22.682059000	26.123211000
C	17.185292000	21.393405000	25.047647000

H	17.531665000	20.483821000	25.556795000
C	13.663613000	19.983486000	23.542592000
C	12.954267000	20.541112000	24.624986000
H	13.489814000	20.928228000	25.503996000
C	11.554905000	20.616726000	24.570051000
H	10.996373000	21.048284000	25.414048000
C	10.867606000	20.135323000	23.440450000
H	9.768574000	20.177195000	23.406028000
C	11.575934000	19.619318000	22.343802000
H	11.018028000	19.264803000	21.466014000
C	12.985212000	19.563174000	22.379109000
P	14.040687000	19.097207000	20.937994000
C	13.604968000	20.415940000	19.655641000
H	14.453360000	20.350089000	18.939048000
C	12.295103000	20.181050000	18.899269000
H	11.428299000	20.087908000	19.583133000
H	12.325924000	19.293507000	18.240426000
H	12.091375000	21.056357000	18.251699000
C	13.612020000	21.797066000	20.319601000
H	13.639866000	22.593952000	19.548941000
H	14.490815000	21.938314000	20.974409000
H	12.701486000	21.948946000	20.933253000
C	13.190782000	17.555777000	20.290462000
H	12.192671000	17.914146000	19.965946000
C	12.989278000	16.501960000	21.378298000
H	12.452456000	15.625305000	20.961619000
H	12.401130000	16.887577000	22.233457000
H	13.958676000	16.140145000	21.773732000
C	13.926937000	17.002812000	19.068906000
H	14.872382000	16.508969000	19.375823000
H	14.161433000	17.784130000	18.317541000
H	13.314575000	16.229608000	18.563233000
Fe	17.814139000	18.635038000	18.542658000
P	17.047314000	19.501312000	16.592889000
C	17.674066000	18.534349000	15.145398000
C	17.306848000	18.799867000	13.811938000
H	16.531193000	19.545184000	13.582513000
C	17.929823000	18.099492000	12.767060000
H	17.647590000	18.310824000	11.724762000
C	18.914688000	17.135801000	13.049693000
H	19.406864000	16.594018000	12.228098000
C	19.259750000	16.844025000	14.379498000
H	20.012102000	16.069178000	14.586344000
C	18.632696000	17.537595000	15.434411000
C	17.790865000	21.165020000	16.305238000
C	17.506881000	21.974557000	15.185982000
H	16.815145000	21.629502000	14.404476000
C	18.084259000	23.248041000	15.088657000
H	17.862770000	23.882717000	14.217620000
C	18.931749000	23.720813000	16.109216000
H	19.370188000	24.727561000	16.038873000
C	19.217524000	22.914564000	17.220344000
H	19.871840000	23.302961000	18.014452000
C	18.656208000	21.623560000	17.312533000
C	15.282204000	19.807405000	16.174113000
C	14.716942000	21.078685000	16.420926000
H	15.315175000	21.868198000	16.898040000
C	13.406185000	21.358740000	16.008512000
H	12.989383000	22.363264000	16.175303000
C	12.633981000	20.365187000	15.381677000
H	11.606838000	20.588392000	15.056090000
C	13.177501000	19.087024000	15.172276000
H	12.580481000	18.303935000	14.681024000
C	14.497886000	18.807200000	15.560251000
H	14.925783000	17.820329000	15.337135000
P	18.815756000	17.126190000	17.220258000
C	17.685686000	15.594165000	17.354296000
H	17.584029000	15.448874000	18.448182000
C	16.302872000	15.901085000	16.773339000
H	16.339463000	15.982224000	15.668278000
H	15.879123000	16.842842000	17.170593000
H	15.596065000	15.086112000	17.028412000
C	18.260454000	14.315537000	16.736565000
H	17.508877000	13.504075000	16.834151000
H	19.182117000	13.963389000	17.237491000
H	18.466963000	14.436195000	15.653032000
C	20.521591000	16.377171000	17.376924000
H	20.483577000	15.529069000	16.661907000
C	20.749310000	15.817160000	18.782446000
H	21.713501000	15.270084000	18.817775000
H	19.957773000	15.114033000	19.105549000
H	20.804958000	16.634550000	19.526811000
C	21.657097000	17.305868000	16.950926000
H	21.843258000	18.081128000	17.714901000
H	21.466248000	17.803418000	15.980380000
H	22.591244000	16.716590000	16.846362000
P	18.949830000	20.475435000	18.728350000
C	18.353401000	21.587447000	20.124549000
H	19.063956000	22.441244000	20.091091000
C	18.442533000	20.960960000	21.501005000
H	18.137299000	21.680347000	22.284533000
H	19.451779000	20.586473000	21.743160000
H	17.764238000	20.066540000	21.609315000
C	16.941028000	22.088326000	19.839283000
H	16.241512000	21.231032000	19.805625000
H	16.861907000	22.648014000	18.888572000
H	16.620139000	22.763263000	20.657168000
C	20.839073000	20.396355000	18.830724000
H	21.015010000	19.356402000	19.178887000
C	21.444215000	20.563251000	17.427588000
H	21.452481000	21.627095000	17.121649000
H	20.891093000	20.001783000	16.649666000
H	22.493919000	20.206832000	17.424961000
C	21.492769000	21.358997000	19.829609000
H	22.592514000	21.211909000	19.800345000
H	21.172107000	21.189038000	20.874813000
H	21.302156000	22.421874000	19.577890000
H	15.492794000	17.546110000	21.823318000
H	18.972402000	18.084084000	19.330255000
H	16.372824000	17.653037000	19.423193000

A (Triplet, def2-tzvp on all atoms)

N	16.700690000	18.631542000	20.642781000
N	17.433561000	18.961738000	19.815160000
Fe	15.498818000	17.843717000	21.750255000
P	15.058718000	19.177877000	23.396342000
C	15.783370000	18.574055000	24.979035000
C	15.681787000	19.224305000	26.217493000
H	15.148384000	20.172912000	26.298821000
C	16.293302000	18.674761000	27.346310000
H	16.210718000	19.179083000	28.310641000
C	17.022028000	17.484089000	27.236658000
H	17.504174000	17.056739000	28.117729000
C	17.153653000	16.854118000	25.996426000
H	17.747439000	15.941137000	25.919190000
C	16.541274000	17.397881000	24.858209000
P	16.723022000	16.712818000	23.143237000
C	18.591250000	16.699974000	23.016079000
H	18.962298000	16.060184000	23.835665000
C	19.122619000	18.126254000	23.198575000
H	18.778322000	18.589903000	24.132521000
H	20.223780000	18.117082000	23.211303000
H	18.807793000	18.753481000	22.351772000
C	19.073728000	16.154386000	21.673005000
H	18.734023000	15.131277000	21.475246000
H	18.728424000	16.801365000	20.856006000
H	20.173558000	16.153113000	21.652245000
C	16.216066000	14.937499000	23.471110000
H	16.858867000	14.565881000	24.286656000
C	16.373916000	14.029412000	22.249387000
H	17.421343000	13.762935000	22.059518000
H	15.818487000	13.090823000	22.404654000

H	15.977002000	14.521530000	21.347353000
C	14.758755000	14.959635000	23.948887000
H	14.630482000	15.565878000	24.856503000
H	14.106312000	15.383504000	23.171394000
H	14.412878000	13.937346000	24.168426000
C	15.506284000	20.957979000	23.523696000
C	14.599074000	21.972429000	23.859163000
H	13.549545000	21.731845000	24.032083000
C	15.031550000	23.297392000	23.971990000
H	14.315484000	24.079540000	24.231343000
C	16.375698000	23.617780000	23.763206000
H	16.715476000	24.650261000	23.859974000
C	17.283514000	22.609514000	23.419541000
H	18.332336000	22.855127000	23.249202000
C	16.849287000	21.291501000	23.285124000
H	17.548887000	20.509123000	22.986471000
C	13.228116000	19.176008000	23.614316000
C	12.541221000	19.435690000	24.807128000
H	13.089861000	19.668962000	25.719791000
C	11.144902000	19.380181000	24.843102000
H	10.617218000	19.566762000	25.779939000
C	10.429191000	19.080481000	23.680730000
H	9.338981000	19.035446000	23.705304000
C	11.108703000	18.822328000	22.486242000
H	10.535065000	18.571037000	21.593489000
C	12.509844000	18.853694000	22.440530000
P	13.528967000	18.406328000	20.955730000
C	13.382911000	19.893034000	19.827798000
H	13.855054000	19.544791000	18.894709000
C	11.946823000	20.328627000	19.538928000
H	11.445070000	20.670530000	20.456358000
H	11.337734000	19.531889000	19.090348000
H	11.950522000	21.172175000	18.833255000
C	14.214247000	21.056814000	20.363177000
H	14.218059000	21.880477000	19.633480000
H	15.253076000	20.759750000	20.547464000
H	13.800536000	21.446451000	21.304288000
C	12.453089000	17.143120000	20.101883000
H	11.458160000	17.592506000	19.955096000
C	12.318705000	15.893037000	20.971402000
H	11.649035000	15.162825000	20.490363000
H	11.912759000	16.118443000	21.968037000
H	13.303850000	15.422839000	21.102799000
C	13.054727000	16.796994000	18.739087000
H	14.081593000	16.424020000	18.864330000
H	13.095008000	17.657903000	18.058022000
H	12.459759000	16.010273000	18.248797000
Fe	18.638106000	19.315148000	18.515248000
P	17.607301000	19.869072000	16.715540000
C	18.090070000	18.763355000	15.303437000
C	17.741881000	18.964043000	13.961271000
H	17.086816000	19.788305000	13.677136000
C	18.233285000	18.108653000	12.970422000
H	17.972681000	18.280630000	11.924397000
C	19.064246000	17.039371000	13.320553000
H	19.456240000	16.375439000	12.547621000
C	19.389745000	16.816248000	14.662398000
H	20.033863000	15.974563000	14.921792000
C	18.906744000	17.673523000	15.661944000
C	18.107175000	21.558787000	16.129106000
C	17.532575000	22.247844000	15.051203000
H	16.732776000	21.789813000	14.468465000
C	17.950205000	23.544549000	14.743094000
H	17.499036000	24.077463000	13.903940000
C	18.930153000	24.166704000	15.525496000
H	19.243478000	25.187837000	15.299778000
C	19.501282000	23.484214000	16.603045000
H	20.250236000	23.987931000	17.215317000
C	19.103861000	22.172406000	16.903230000
C	15.784095000	20.020973000	16.460654000
C	15.131654000	21.183279000	16.908997000

H	15.712886000	21.982560000	17.371534000
C	13.756013000	21.338070000	16.746021000
H	13.272508000	22.259159000	17.076369000
C	12.995626000	20.315915000	16.165839000
H	11.917875000	20.433658000	16.043766000
C	13.627178000	19.140363000	15.751945000
H	13.044380000	18.333753000	15.302890000
C	15.010770000	18.997403000	15.894010000
H	15.494866000	18.086433000	15.543644000
P	19.229615000	17.519774000	17.476957000
C	18.108158000	16.061645000	17.940369000
H	18.248999000	15.993927000	19.030814000
C	16.643077000	16.412308000	17.686615000
H	16.424925000	16.417775000	16.607774000
H	16.375044000	17.389666000	18.104158000
H	15.994654000	15.655469000	18.153318000
C	18.442329000	14.710677000	17.306085000
H	17.738486000	13.944805000	17.672345000
H	19.455030000	14.357076000	17.540750000
H	18.337257000	14.748759000	16.210905000
C	20.904527000	16.681274000	17.515200000
H	20.815397000	15.773915000	16.896773000
C	21.244426000	16.266863000	18.945825000
H	22.249714000	15.817323000	18.986479000
H	20.533756000	15.532232000	19.346744000
H	21.229216000	17.145236000	19.608824000
C	22.002132000	17.563834000	16.932151000
H	22.137946000	18.459652000	17.550892000
H	21.776835000	17.887250000	15.907456000
H	22.958470000	17.016586000	16.912220000
P	19.781532000	21.116046000	18.265402000
C	19.622751000	22.281946000	19.726079000
H	20.180167000	23.201413000	19.480899000
C	20.213446000	21.645855000	20.985580000
H	20.175094000	22.353425000	21.828665000
H	21.262408000	21.341255000	20.854040000
H	19.644560000	20.745563000	21.258966000
C	18.145243000	22.627297000	19.925584000
H	17.560991000	21.714319000	20.111186000
H	17.720533000	23.123885000	19.041511000
H	18.014943000	23.299161000	20.786857000
C	21.617774000	21.116461000	17.853207000
H	21.979369000	20.241215000	18.416908000
C	21.775305000	20.839002000	16.353314000
H	21.592033000	21.753497000	15.770525000
H	21.065072000	20.075023000	16.006148000
H	22.793603000	20.489963000	16.123783000
C	22.443433000	22.334972000	18.271316000
H	23.502411000	22.171620000	18.011194000
H	22.398888000	22.528535000	19.351134000
H	22.121329000	23.246804000	17.748909000
H	15.408506000	16.533328000	20.928254000
H	19.801246000	19.017054000	19.483941000

A (Singlet, def2-tzvp on all atoms)

N	16.693584000	18.574024000	20.649174000
N	17.419877000	18.967068000	19.847725000
Fe	15.498909000	17.790604000	21.757600000
P	15.066530000	19.134034000	23.383354000
C	15.756864000	18.525473000	24.981645000
C	15.645786000	19.179826000	26.217176000
H	15.128002000	20.137788000	26.288241000
C	16.227197000	18.621713000	27.357822000
H	16.135724000	19.129185000	28.319736000
C	16.936479000	17.418078000	27.263622000
H	17.394932000	16.983858000	28.153967000
C	17.079647000	16.784091000	26.026724000
H	17.659672000	15.861316000	25.960802000
C	16.496621000	17.336519000	24.877251000
P	16.701202000	16.651311000	23.166132000
C	18.573429000	16.627151000	23.072894000

H	18.921076000	15.961191000	23.881812000
C	19.121832000	18.039386000	23.303261000
H	18.775364000	18.481041000	24.246878000
H	20.222661000	18.013798000	23.325692000
H	18.825436000	18.697738000	22.473154000
C	19.075005000	16.112396000	21.724870000
H	18.727341000	15.099059000	21.493091000
H	18.749025000	16.785173000	20.921008000
H	20.175091000	16.100334000	21.722423000
C	16.193879000	14.873899000	23.485670000
H	16.828426000	14.502316000	24.307731000
C	16.370379000	13.970396000	22.263389000
H	17.422260000	13.716685000	22.079844000
H	15.824689000	13.025022000	22.412379000
H	15.973477000	14.460973000	21.360335000
C	14.731366000	14.889221000	23.946076000
H	14.591280000	15.488806000	24.856363000
H	14.087617000	15.317724000	23.164029000
H	14.385152000	13.864628000	24.154303000
C	15.564362000	20.903287000	23.506737000
C	14.688040000	21.950913000	23.821811000
H	13.630101000	21.746487000	23.989980000
C	15.161818000	23.262984000	23.920720000
H	14.468971000	24.070737000	24.164446000
C	16.516896000	23.538533000	23.718320000
H	16.887966000	24.561258000	23.803734000
C	17.394741000	22.497361000	23.395456000
H	18.451921000	22.705791000	23.228542000
C	16.918804000	21.192634000	23.275789000
H	17.594899000	20.383877000	22.994522000
C	13.234881000	19.189665000	23.590432000
C	12.544604000	19.491200000	24.771428000
H	13.090069000	19.736600000	25.682630000
C	11.146966000	19.460700000	24.798739000
H	10.616502000	19.678048000	25.727383000
C	10.433964000	19.145685000	23.639003000
H	9.343005000	19.118492000	23.657109000
C	11.117268000	18.850751000	22.454825000
H	10.545468000	18.590708000	21.563552000
C	12.518869000	18.857254000	22.418998000
P	13.545878000	18.369237000	20.952456000
C	13.421758000	19.848726000	19.808119000
H	13.908038000	19.497447000	18.883102000
C	11.991100000	20.287806000	19.498092000
H	11.474288000	20.624185000	20.409082000
H	11.388710000	19.495336000	19.033216000
H	12.007538000	21.136482000	18.799310000
C	14.245129000	21.013879000	20.353675000
H	14.248834000	21.841041000	19.627910000
H	15.284581000	20.722391000	20.542407000
H	13.823660000	21.396958000	21.294044000
C	12.451621000	17.115955000	20.106934000
H	11.463247000	17.579446000	19.959732000
C	12.299393000	15.873400000	20.983643000
H	11.614347000	15.152498000	20.510107000
H	11.902503000	16.112040000	21.980973000
H	13.276672000	15.386779000	21.113583000
C	13.044402000	16.754676000	18.744490000
H	14.069899000	16.377621000	18.868518000
H	13.085001000	17.610652000	18.057225000
H	12.443134000	15.967116000	18.263233000
Fe	18.621874000	19.385058000	18.539140000
P	17.576321000	19.910606000	16.734401000
C	18.072256000	18.793633000	15.334873000
C	17.722355000	18.978498000	13.990883000
H	17.057869000	19.792622000	13.699732000
C	18.222900000	18.119844000	13.007748000
H	17.960016000	18.279563000	11.960375000
C	19.065859000	17.062953000	13.367088000
H	19.464999000	16.396606000	12.599959000
C	19.393890000	16.855538000	14.710564000
H	20.047384000	16.023754000	14.978244000
C	18.900863000	17.716162000	15.702369000
C	18.071679000	21.592634000	16.122193000
C	17.487006000	22.268999000	15.041654000
H	16.678820000	21.806428000	14.474248000
C	17.904925000	23.560043000	14.711317000
H	17.444926000	24.083153000	13.870840000
C	18.896767000	24.189416000	15.472657000
H	19.210022000	25.206606000	15.229883000
C	19.480246000	23.518873000	16.550882000
H	20.239059000	24.027472000	17.146757000
C	19.081385000	22.212876000	16.873640000
C	15.753793000	20.047651000	16.467227000
C	15.091339000	21.212382000	16.894219000
H	15.663460000	22.021478000	17.351026000
C	13.717073000	21.358674000	16.713808000
H	13.226316000	22.282302000	17.025698000
C	12.967608000	20.325465000	16.139175000
H	11.890830000	20.437057000	16.003582000
C	13.608336000	19.147359000	15.748484000
H	13.034238000	18.332319000	15.303544000
C	14.991201000	19.013335000	15.906013000
H	15.482759000	18.100841000	15.570928000
P	19.221773000	17.578351000	17.517503000
C	18.099688000	16.123262000	17.989202000
H	18.220514000	16.073970000	19.082765000
C	16.637429000	16.459737000	17.701837000
H	16.442932000	16.453469000	16.618671000
H	16.349229000	17.436710000	18.106587000
H	15.988141000	15.699713000	18.161455000
C	18.450637000	14.761464000	17.387745000
H	17.733843000	14.004332000	17.746968000
H	19.454346000	14.409002000	17.660153000
H	18.378798000	14.778011000	16.289273000
C	20.895498000	16.738591000	17.568412000
H	20.799318000	15.821052000	16.966484000
C	21.231029000	16.345452000	19.005905000
H	22.232453000	15.888345000	19.054940000
H	20.513491000	15.623214000	19.416061000
H	21.221504000	17.233309000	19.656420000
C	22.001789000	17.597607000	16.967322000
H	22.147579000	18.507030000	17.563303000
H	21.783022000	17.896493000	15.933982000
H	22.952303000	17.039987000	16.963331000
P	19.778087000	21.172697000	18.236994000
C	19.657012000	22.357401000	19.683681000
H	20.219318000	23.266594000	19.413079000
C	20.270034000	21.733553000	20.939070000
H	20.305262000	22.470506000	21.756709000
H	21.295630000	21.371569000	20.773701000
H	19.668729000	20.873116000	21.266620000
C	18.187782000	22.723576000	19.906614000
H	17.591701000	21.820406000	20.104983000
H	17.756503000	23.223796000	19.027774000
H	18.080906000	23.399568000	20.768011000
C	21.604507000	21.149400000	17.789282000
H	21.971200000	20.282839000	18.362826000
C	21.728984000	20.841714000	16.292441000
H	21.530383000	21.743608000	15.695341000
H	21.013364000	20.069272000	15.976796000
H	22.742788000	20.490101000	16.047952000
C	22.446043000	22.369396000	18.168852000
H	23.499086000	22.191101000	17.895268000
H	22.420125000	22.584721000	19.245156000
H	22.123106000	23.272826000	17.632663000
H	15.392544000	16.472522000	20.943193000
H	19.788552000	19.107242000	19.509809000

A – PBE0

N	16.676535000	18.504847000	20.617494000
N	17.332981000	18.989864000	19.837892000

Fe	15.417705000	17.671917000	21.693501000
P	15.300051000	18.954997000	23.422043000
C	15.950011000	18.139019000	24.944634000
C	16.059174000	18.744230000	26.202708000
H	15.737398000	19.778840000	26.347719000
C	16.609391000	18.039700000	27.271374000
H	16.692324000	18.513789000	28.252723000
C	17.065883000	16.732663000	27.083898000
H	17.502218000	16.180807000	27.920410000
C	16.983618000	16.137504000	25.826358000
H	17.360371000	15.119867000	25.691538000
C	16.434072000	16.838296000	24.745938000
P	16.306781000	16.165223000	23.020153000
C	18.051350000	15.529853000	22.801551000
H	18.299619000	14.943321000	23.702576000
C	18.977657000	16.738700000	22.730901000
H	18.873405000	17.387303000	23.614680000
H	20.029551000	16.414505000	22.678524000
H	18.763135000	17.349366000	21.840552000
C	18.193224000	14.636331000	21.575720000
H	17.808682000	13.622643000	21.752191000
H	17.654243000	15.056744000	20.713386000
H	19.254810000	14.533293000	21.298063000
C	15.258342000	14.651898000	23.389249000
H	15.830649000	14.043977000	24.113015000
C	14.960101000	13.815404000	22.152377000
H	15.844668000	13.290306000	21.770126000
H	14.200019000	13.052120000	22.387937000
H	14.576025000	14.452826000	21.340988000
C	13.959457000	15.112184000	24.045086000
H	14.131867000	15.666447000	24.979247000
H	13.394950000	15.771466000	23.367520000
H	13.322435000	14.243990000	24.280736000
C	16.137367000	20.586499000	23.600480000
C	15.531997000	21.746082000	24.095064000
H	14.476601000	21.739218000	24.376899000
C	16.269518000	22.923827000	24.229033000
H	15.785057000	23.824218000	24.616075000
C	17.617972000	22.951045000	23.875971000
H	18.196111000	23.872109000	23.987124000
C	18.224674000	21.798998000	23.372427000
H	19.277018000	21.815306000	23.083843000
C	17.487069000	20.627940000	23.223532000
H	17.956442000	19.734315000	22.802030000
C	13.517654000	19.329271000	23.729780000
C	12.945297000	19.665027000	24.960550000
H	13.564418000	19.736800000	25.857409000
C	11.571770000	19.889370000	25.059508000
H	11.128944000	20.136347000	26.027639000
C	10.765992000	19.790061000	23.925705000
H	9.688962000	19.960456000	24.001703000
C	11.330240000	19.455984000	22.694052000
H	10.682758000	19.361507000	21.819017000
C	12.703835000	19.212095000	22.583243000
P	13.559313000	18.653414000	21.034697000
C	13.656981000	20.204798000	19.995105000
H	14.152034000	19.841671000	19.078038000
C	12.317228000	20.826018000	19.629902000
H	11.783495000	21.181001000	20.526285000
H	11.654280000	20.136580000	19.087659000
H	12.474631000	21.705387000	18.983236000
C	14.573975000	21.229801000	20.643770000
H	14.731586000	22.080520000	19.960627000
H	15.556570000	20.809470000	20.889287000
H	14.137241000	21.629300000	21.572985000
C	12.254787000	17.653007000	20.158804000
H	11.350005000	18.276794000	20.067030000
C	11.915995000	16.407300000	20.963034000
H	11.142639000	15.815134000	20.446334000
H	11.534937000	16.650053000	21.967050000
H	12.809601000	15.775641000	21.080170000
C	12.757069000	17.299472000	18.764522000
H	13.718947000	16.764503000	18.828982000
H	12.913641000	18.185941000	18.133261000
H	12.032466000	16.649103000	18.247210000
Fe	18.515918000	19.752484000	18.621181000
P	17.547505000	19.832558000	16.667126000
C	18.480704000	18.748052000	15.477051000
C	18.365285000	18.824723000	14.085639000
H	17.653472000	19.515516000	13.628021000
C	19.166741000	18.029801000	13.266410000
H	19.082065000	18.107768000	12.179277000
C	20.081403000	17.143205000	13.835514000
H	20.715992000	16.523982000	13.195976000
C	20.189642000	17.048351000	15.223077000
H	20.911577000	16.351429000	15.656305000
C	19.398648000	17.850481000	16.053844000
C	17.716862000	21.524087000	15.919266000
C	17.031122000	21.967778000	14.782045000
H	16.363779000	21.290638000	14.242922000
C	17.163450000	23.286682000	14.352309000
H	16.622188000	23.627929000	13.466114000
C	17.966617000	24.175463000	15.069236000
H	18.052056000	25.217363000	14.749752000
C	18.655107000	23.737537000	16.199670000
H	19.266446000	24.449977000	16.758157000
C	18.548655000	22.406712000	16.624063000
C	15.788355000	19.512252000	16.187709000
C	14.822786000	20.440391000	16.605913000
H	15.130096000	21.317141000	17.184113000
C	13.481540000	20.273301000	16.270900000
H	12.745927000	21.015699000	16.588473000
C	13.076488000	19.157908000	15.534326000
H	12.023041000	19.023563000	15.276045000
C	14.023364000	18.216550000	15.135850000
H	13.716819000	17.338472000	14.561145000
C	15.370412000	18.396191000	15.455013000
H	16.103557000	17.664276000	15.112407000
P	19.461060000	17.882792000	17.899407000
C	18.457955000	16.349960000	18.339590000
H	18.388270000	16.441467000	19.435769000
C	17.043309000	16.437430000	17.785828000
H	17.037578000	16.270609000	16.697124000
H	16.569006000	17.404944000	17.992714000
H	16.410740000	15.662236000	18.247165000
C	19.076916000	15.005949000	17.984659000
H	18.401301000	14.189383000	18.291438000
H	20.042828000	14.823097000	18.476656000
H	19.225746000	14.906215000	16.896740000
C	21.189929000	17.299899000	18.308317000
H	21.344770000	16.336221000	17.795469000
C	21.295830000	17.071991000	19.810997000
H	22.319806000	16.764200000	20.081100000
H	20.611871000	16.289945000	20.170456000
H	21.061173000	17.997733000	20.359912000
C	22.255661000	18.271896000	17.832918000
H	22.142780000	19.247104000	18.327653000
H	22.220476000	18.433865000	16.746856000
H	23.258619000	17.885256000	18.079901000
P	19.462544000	21.648658000	18.042909000
C	19.332883000	22.973752000	19.348763000
H	19.594986000	23.933400000	18.871901000
C	20.301115000	22.705930000	20.491742000
H	20.185159000	23.468928000	21.279024000
H	21.353199000	22.723932000	20.168700000
H	20.104298000	21.716680000	20.932457000
C	17.890327000	23.035842000	19.833621000
H	17.588788000	22.075817000	20.281656000
H	17.191734000	23.254172000	19.010761000
H	17.767853000	23.818458000	20.600463000
C	21.229293000	21.779776000	17.427157000
H	21.785119000	21.200748000	18.183681000

C	21.344890000	21.069061000	16.084240000
H	20.889145000	21.671858000	15.282427000
H	20.841796000	20.092992000	16.088342000
H	22.402922000	20.910022000	15.820035000
C	21.833885000	23.174933000	17.345812000
H	22.899642000	23.098145000	17.071424000
H	21.782636000	23.727722000	18.294414000
H	21.347879000	23.783238000	16.568197000
H	15.179534000	16.529491000	20.666754000
H	19.528309000	19.845654000	19.786588000

AP2 – PBE0

N	16.818591000	18.729848000	20.528295000
N	16.945760000	19.421690000	19.511886000
Fe	15.649217000	17.798685000	21.532291000
P	15.329162000	19.133613000	23.278983000
C	16.107226000	18.447416000	24.802869000
C	16.261534000	19.164973000	25.995785000
H	15.935498000	20.206218000	26.060698000
C	16.856464000	18.559898000	27.099969000
H	16.977613000	19.123008000	28.028578000
C	17.306476000	17.240031000	27.018097000
H	17.775561000	16.767609000	27.884529000
C	17.175163000	16.530193000	25.827032000
H	17.547421000	15.503802000	25.771153000
C	16.585182000	17.132999000	24.709760000
P	16.424086000	16.301627000	23.075135000
C	18.163829000	15.700377000	22.807971000
H	18.455675000	15.174814000	23.733430000
C	19.057170000	16.924407000	22.625959000
H	18.995361000	17.609241000	23.486105000
H	20.108364000	16.609788000	22.530594000
H	18.773707000	17.495825000	21.726495000
C	18.273671000	14.733543000	21.635735000
H	17.885196000	13.735361000	21.877313000
H	17.724182000	15.106414000	20.758892000
H	19.330722000	14.606617000	21.353330000
C	15.362468000	14.839390000	23.527935000
H	15.925267000	14.272762000	24.290654000
C	15.084337000	13.933436000	22.333618000
H	15.949019000	13.309905000	22.074980000
H	14.247593000	13.254990000	22.563780000
H	14.820181000	14.516998000	21.438911000
C	14.076210000	15.366531000	24.159754000
H	14.264573000	15.899900000	25.102880000
H	13.551694000	16.062067000	23.487073000
H	13.391925000	14.530944000	24.376197000
C	15.886556000	20.874670000	23.402876000
C	15.058993000	21.944944000	23.756064000
H	13.993849000	21.784375000	23.935947000
C	15.589611000	23.229440000	23.885236000
H	14.934102000	24.059525000	24.159913000
C	16.949891000	23.449406000	23.676989000
H	17.367176000	24.453257000	23.789148000
C	17.777965000	22.384270000	23.318175000
H	18.842346000	22.552825000	23.147710000
C	17.248861000	21.106076000	23.164854000
H	17.895748000	20.278096000	22.859243000
C	13.524159000	19.180794000	23.662651000
C	13.004043000	19.518272000	24.916430000
H	13.671724000	19.787774000	25.737143000
C	11.628104000	19.492892000	25.137596000
H	11.231951000	19.744949000	26.124133000
C	10.761885000	19.136637000	24.104170000
H	9.683203000	19.110486000	24.276970000
C	11.270312000	18.800924000	22.850472000
H	10.579771000	18.509480000	22.055923000
C	12.651226000	18.811921000	22.618794000
P	13.435693000	18.308826000	21.032176000
C	13.255482000	19.816633000	19.938819000
H	13.784004000	19.521131000	19.016275000
C	11.816499000	20.166601000	19.588328000
H	11.213589000	20.352709000	20.491216000
H	11.315726000	19.388656000	18.994748000
H	11.794164000	21.091318000	18.990252000
C	13.980127000	21.009924000	20.543990000
H	13.977765000	21.854983000	19.836964000
H	15.025349000	20.784247000	20.797734000
H	13.478792000	21.353948000	21.461657000
C	12.258625000	17.102041000	20.252360000
H	11.296307000	17.624275000	20.125305000
C	12.032852000	15.876075000	21.122402000
H	11.311456000	15.200985000	20.634660000
H	11.628933000	16.130923000	22.113049000
H	12.965034000	15.314172000	21.268148000
C	12.801208000	16.723065000	18.878129000
H	13.813022000	16.295569000	18.965196000
H	12.863686000	17.580537000	18.190807000
H	12.148698000	15.970267000	18.407815000
Fe	18.710138000	19.682783000	18.569202000
P	17.785744000	19.994886000	16.554372000
C	18.518418000	18.828721000	15.328732000
C	18.395576000	18.968668000	13.942459000
H	17.830174000	19.799769000	13.516568000
C	19.006235000	18.052167000	13.088824000
H	18.917818000	18.175667000	12.006764000
C	19.733535000	16.983856000	13.615225000
H	20.217103000	16.267770000	12.946449000
C	19.849874000	16.829346000	14.995393000
H	20.427901000	15.991715000	15.393004000
C	19.247637000	17.748462000	15.862921000
C	18.094471000	21.699291000	15.919435000
C	17.484715000	22.229672000	14.775996000
H	16.804670000	21.619100000	14.176828000
C	17.710118000	23.555827000	14.416745000
H	17.232273000	23.965683000	13.523750000
C	18.524144000	24.366740000	15.210014000
H	18.684950000	25.412916000	14.938641000
C	19.122705000	23.849264000	16.356497000
H	19.739792000	24.502804000	16.977390000
C	18.919380000	22.510360000	16.713992000
C	15.977176000	19.884735000	16.259509000
C	15.173727000	20.882566000	16.832610000
H	15.635375000	21.708864000	17.382071000
C	13.789896000	20.846508000	16.681796000
H	13.178073000	21.640478000	17.115517000
C	13.187320000	19.798803000	15.983214000
H	12.100268000	19.765125000	15.876257000
C	13.979047000	18.799937000	15.418339000
H	13.516505000	17.979749000	14.863641000
C	15.367528000	18.845540000	15.549074000
H	15.976984000	18.067280000	15.086636000
P	19.357326000	17.665081000	17.696111000
C	18.172926000	16.303780000	18.183697000
H	18.080947000	16.455621000	19.272334000
C	16.800291000	16.542786000	17.576643000
H	16.814403000	16.392661000	16.486096000
H	16.421579000	17.550305000	17.789496000
H	16.079992000	15.829394000	18.004364000
C	18.654927000	14.889636000	17.893624000
H	17.895048000	14.165759000	18.230810000
H	19.593178000	14.634347000	18.405851000
H	18.798741000	14.725774000	16.813535000
C	21.035786000	16.947727000	18.040695000
H	21.072047000	15.975393000	17.522266000
C	21.184448000	16.715875000	19.538089000
H	22.178383000	16.294947000	19.759655000
H	20.434012000	16.016738000	19.932021000
H	21.078257000	17.664114000	20.087242000
C	22.151869000	17.830162000	17.505864000
H	22.175651000	18.794971000	18.033398000
H	22.056364000	18.026417000	16.428581000

H	23.124609000	17.339215000	17.669612000
P	19.705272000	21.684514000	18.154653000
C	19.575483000	22.922751000	19.524095000
H	19.997071000	23.868195000	19.142651000
C	20.390561000	22.467870000	20.728249000
H	20.369896000	23.242635000	21.510958000
H	21.446591000	22.285779000	20.478089000
H	19.985653000	21.533564000	21.145583000
C	18.104850000	23.136325000	19.862842000
H	17.639068000	22.202413000	20.216343000
H	17.533674000	23.494408000	18.992168000
H	17.998507000	23.887163000	20.661250000
C	21.500768000	21.618994000	17.651466000
H	21.934177000	20.909319000	18.376505000
C	21.601693000	21.018164000	16.253230000
H	21.272978000	21.741415000	15.490643000
H	20.988319000	20.112084000	16.141106000
H	22.645930000	20.749573000	16.030040000
C	22.260552000	22.934687000	17.736413000
H	23.312895000	22.765690000	17.454996000
H	22.261390000	23.368223000	18.746350000
H	21.858058000	23.685066000	17.038901000
H	15.700802000	16.674035000	20.489186000
H	19.654230000	19.531431000	19.788413000
H	16.048398000	19.741738000	19.090470000

B – PBE0

N	16.522544000	18.647129000	20.380146000
N	17.349857000	19.084724000	19.708483000
Fe	15.251005000	17.965243000	21.447536000
P	15.441498000	19.092563000	23.250139000
C	16.017071000	18.069091000	24.683666000
C	16.384003000	18.552940000	25.945489000
H	16.364330000	19.626640000	26.151307000
C	16.804730000	17.669476000	26.939165000
H	17.088213000	18.050158000	27.924192000
C	16.871402000	16.300174000	26.670601000
H	17.201627000	15.605335000	27.447610000
C	16.533982000	15.820983000	25.404446000
H	16.611815000	14.748540000	25.201658000
C	16.109749000	16.700555000	24.401050000
P	15.728755000	16.222608000	22.641641000
C	17.377323000	15.369161000	22.281678000
H	17.563450000	14.667486000	23.114636000
C	18.488865000	16.409772000	22.255144000
H	18.552841000	16.958504000	23.208017000
H	19.465880000	15.924860000	22.088369000
H	18.324548000	17.147640000	21.454746000
C	17.326039000	14.584592000	20.980649000
H	16.699125000	13.684616000	21.061514000
H	16.927293000	15.215082000	20.172113000
H	18.335347000	14.250043000	20.685816000
C	14.607922000	14.728882000	22.935212000
H	15.230171000	13.906948000	23.333401000
C	13.977327000	14.283047000	21.620667000
H	14.704299000	13.817864000	20.941109000
H	13.172584000	13.549754000	21.800559000
H	13.556234000	15.149665000	21.088521000
C	13.544241000	15.093808000	23.967139000
H	13.953182000	15.127972000	24.986738000
H	13.118575000	16.086672000	23.751396000
H	12.718275000	14.362548000	23.960911000
C	16.541260000	20.561177000	23.483689000
C	16.117518000	21.849273000	23.826262000
H	15.055930000	22.051787000	23.984272000
C	17.042635000	22.884505000	23.980654000
H	16.694183000	23.885442000	24.250976000
C	18.403035000	22.641407000	23.802789000
H	19.128654000	23.447949000	23.937653000
C	18.832431000	21.363979000	23.435380000
H	19.895548000	21.168902000	23.274298000
C	17.907980000	20.340119000	23.258175000
H	18.240362000	19.351034000	22.930849000
C	13.794104000	19.738357000	23.824251000
C	13.518998000	20.264215000	25.091255000
H	14.311673000	20.350546000	25.837746000
C	12.225959000	20.674788000	25.420513000
H	12.015971000	21.068804000	26.418429000
C	11.205892000	20.579264000	24.474435000
H	10.191035000	20.898783000	24.727094000
C	11.477609000	20.065043000	23.204747000
H	10.663301000	19.986224000	22.480052000
C	12.765623000	19.628292000	22.871882000
P	13.276722000	18.818397000	21.275973000
C	13.235939000	20.290255000	20.084635000
H	13.584903000	19.814136000	19.153761000
C	11.897364000	20.967016000	19.827206000
H	11.490796000	21.425384000	20.743671000
H	11.134438000	20.285395000	19.422352000
H	12.020457000	21.781047000	19.091100000
C	14.291546000	21.306182000	20.495673000
H	14.365394000	22.111426000	19.745159000
H	15.277720000	20.833393000	20.592644000
H	14.040856000	21.779852000	21.459809000
C	11.684905000	17.955079000	20.780249000
H	10.905053000	18.733461000	20.744642000
C	11.249399000	16.906928000	21.791693000
H	10.263798000	16.494986000	21.513804000
H	11.161587000	17.318328000	22.808412000
H	11.960579000	16.073236000	21.830864000
C	11.832015000	17.353684000	19.389673000
H	12.663997000	16.632136000	19.368950000
H	12.048478000	18.113721000	18.625071000
H	10.908156000	16.830937000	19.088393000
Fe	18.697317000	19.593991000	18.599705000
P	17.843827000	19.503455000	16.636213000
C	18.998936000	18.635627000	15.467946000
C	18.958333000	18.722049000	14.074025000
H	18.160367000	19.281920000	13.579819000
C	19.950734000	18.114935000	13.300530000
H	19.923673000	18.202771000	12.210957000
C	20.979069000	17.407154000	13.921476000
H	21.761407000	16.936298000	13.319801000
C	21.013369000	17.299670000	15.314595000
H	21.824259000	16.737901000	15.785899000
C	20.034576000	17.917841000	16.099508000
C	17.646048000	21.205092000	15.912754000
C	16.891202000	21.520459000	14.777609000
H	16.398962000	20.727971000	14.207605000
C	16.725755000	22.850790000	14.391038000
H	16.129194000	23.093397000	13.507425000
C	17.300646000	23.870628000	15.150530000
H	17.151733000	24.916076000	14.866440000
C	18.058838000	23.558566000	16.280806000
H	18.488563000	24.370519000	16.872152000
C	18.249868000	22.225781000	16.665224000
C	16.181016000	18.844391000	16.156685000
C	15.086355000	19.438003000	16.803481000
H	15.269657000	20.221671000	17.545774000
C	13.785488000	19.039965000	16.509757000
H	12.941185000	19.531913000	16.999644000
C	13.555001000	18.007805000	15.596442000
H	12.533410000	17.687424000	15.375852000
C	14.637090000	17.386801000	14.975318000
H	14.467956000	16.573028000	14.264383000
C	15.940932000	17.809314000	15.246549000
H	16.776132000	17.326130000	14.734366000
P	19.962595000	17.948309000	17.956330000
C	19.244356000	16.232069000	18.291276000
H	19.213571000	16.204317000	19.393411000
C	17.812878000	16.161809000	17.784684000
H	17.772339000	16.280888000	16.690669000

H	17.186558000	16.940371000	18.242515000
H	17.372082000	15.178114000	18.016446000
C	20.057589000	15.051306000	17.780171000
H	19.585402000	14.101605000	18.086520000
H	21.088927000	15.034957000	18.162185000
H	20.107038000	15.045563000	16.679022000
C	21.738760000	17.670439000	18.488501000
H	22.150274000	16.820169000	17.919608000
C	21.750569000	17.326580000	19.974051000
H	22.780716000	17.332872000	20.367949000
H	21.326966000	16.332168000	20.178160000
H	21.156153000	18.064532000	20.538615000
C	22.583390000	18.901272000	18.208556000
H	22.179310000	19.768906000	18.751030000
H	22.604615000	19.152416000	17.138521000
H	23.624574000	18.742996000	18.537806000
P	19.274705000	21.615096000	18.091720000
C	18.979145000	22.935277000	19.386930000
H	19.065749000	23.920330000	18.896554000
C	20.025418000	22.829991000	20.486470000
H	19.799091000	23.528250000	21.308464000
H	21.042708000	23.053600000	20.129994000
H	20.023691000	21.809327000	20.896708000
C	17.573517000	22.768236000	19.944304000
H	17.473948000	21.790596000	20.438548000
H	16.815027000	22.812127000	19.147022000
H	17.345158000	23.555072000	20.682850000
C	20.989850000	22.093625000	17.446937000
H	21.652803000	21.639179000	18.202637000
C	21.240652000	21.400450000	16.115483000
H	20.652968000	21.869955000	15.309535000
H	20.958370000	20.339909000	16.151811000
H	22.304701000	21.467905000	15.831736000
C	21.319651000	23.576536000	17.340160000
H	22.376611000	23.705595000	17.047617000
H	21.179233000	24.124497000	18.282717000
H	20.712658000	24.071433000	16.566213000
H	14.804686000	17.033745000	20.300960000
H	19.613191000	19.843622000	19.822158000

BP2 – PBE0

N	16.505707000	18.469373000	20.387608000
N	17.192127000	19.140866000	19.671111000
Fe	15.361952000	17.737811000	21.448570000
P	15.617115000	18.875539000	23.273625000
C	16.118282000	17.817953000	24.696271000
C	16.572969000	18.289260000	25.933523000
H	16.687519000	19.362814000	26.107349000
C	16.904178000	17.386337000	26.941461000
H	17.259462000	17.754156000	27.907469000
C	16.790334000	16.012309000	26.712765000
H	17.052367000	15.303836000	27.502735000
C	16.364812000	15.542103000	25.471403000
H	16.306706000	14.463579000	25.298618000
C	16.032793000	16.441758000	24.451307000
P	15.598942000	15.958743000	22.714259000
C	17.179469000	15.019128000	22.313796000
H	17.275676000	14.235919000	23.086357000
C	18.371060000	15.964408000	22.412592000
H	18.477723000	16.395716000	23.419097000
H	19.304287000	15.425551000	22.180589000
H	18.259379000	16.794765000	21.697667000
C	17.119699000	14.368821000	20.939807000
H	16.415931000	13.525604000	20.899104000
H	16.818360000	15.107643000	20.182225000
H	18.111541000	13.976929000	20.661326000
C	14.353751000	14.591281000	23.009295000
H	14.905074000	13.740307000	23.447083000
C	13.725908000	14.142131000	21.695042000
H	14.442176000	13.624685000	21.042889000
H	12.888648000	13.450710000	21.885571000
H	13.344113000	15.003527000	21.128328000
C	13.304621000	15.069789000	24.009723000
H	13.708533000	15.136489000	25.029476000
H	12.924557000	16.067580000	23.742794000
H	12.446144000	14.378640000	24.034205000
C	16.809326000	20.266643000	23.417175000
C	16.486773000	21.570267000	23.808452000
H	15.457751000	21.828400000	24.066994000
C	17.476605000	22.552752000	23.870441000
H	17.211860000	23.567877000	24.178248000
C	18.795842000	22.240316000	23.549591000
H	19.571590000	23.007680000	23.607308000
C	19.120196000	20.947336000	23.135279000
H	20.147632000	20.700203000	22.857466000
C	18.131938000	19.972149000	23.055132000
H	18.381221000	18.971064000	22.694519000
C	14.002411000	19.590032000	23.828848000
C	13.770119000	20.162588000	25.084097000
H	14.570659000	20.216964000	25.825248000
C	12.507716000	20.659459000	25.406134000
H	12.330223000	21.096796000	26.391713000
C	11.475005000	20.596230000	24.469940000
H	10.485210000	20.987270000	24.719187000
C	11.699694000	20.023667000	23.217996000
H	10.876356000	19.968043000	22.501560000
C	12.958553000	19.505714000	22.890748000
P	13.405502000	18.670519000	21.300811000
C	13.538166000	20.141137000	20.123252000
H	14.005021000	19.674849000	19.238794000
C	12.240432000	20.817288000	19.703015000
H	11.697828000	21.229991000	20.569564000
H	11.556916000	20.150663000	19.158605000
H	12.464280000	21.665722000	19.034107000
C	14.506444000	21.173706000	20.683121000
H	14.749031000	21.923817000	19.914300000
H	15.445572000	20.720201000	21.027834000
H	14.063535000	21.710833000	21.537385000
C	11.787588000	17.921706000	20.745059000
H	11.098552000	18.775129000	20.639951000
C	11.173841000	16.953565000	21.743447000
H	10.165876000	16.660224000	21.405774000
H	11.070000000	17.391662000	22.746938000
H	11.768886000	16.037231000	21.834581000
C	11.964794000	17.274583000	19.376878000
H	12.637311000	16.405813000	19.439409000
H	12.399592000	17.959913000	18.634395000
H	10.993477000	16.929172000	18.985778000
Fe	18.527580000	19.877653000	18.579003000
P	17.765121000	19.668186000	16.563850000
C	18.965061000	18.699137000	15.537041000
C	18.962266000	18.650911000	14.140697000
H	18.183771000	19.164549000	13.571349000
C	19.967218000	17.962499000	13.459165000
H	19.966870000	17.940116000	12.366266000
C	20.975511000	17.314740000	14.172657000
H	21.767315000	16.781696000	13.639703000
C	20.978528000	17.348625000	15.568656000
H	21.775706000	16.838918000	16.116052000
C	19.981058000	18.041992000	16.261769000
C	17.639308000	21.324036000	15.743432000
C	16.944340000	21.574612000	14.555438000
H	16.475316000	20.752920000	14.007838000
C	16.806433000	22.880767000	14.087421000
H	16.255383000	23.074174000	13.163586000
C	17.349127000	23.940940000	14.815012000
H	17.219417000	24.968303000	14.464539000
C	18.050615000	23.694137000	15.995773000
H	18.456412000	24.537503000	16.559143000
C	18.212845000	22.384741000	16.464413000
C	16.107273000	19.001299000	16.098877000
C	15.002691000	19.697788000	16.613499000

H	15.167329000	20.609351000	17.197023000
C	13.704812000	19.252302000	16.382381000
H	12.857255000	19.816901000	16.778937000
C	13.488220000	18.084904000	15.646206000
H	12.470448000	17.727695000	15.469673000
C	14.577967000	17.380145000	15.138267000
H	14.416893000	16.466754000	14.559665000
C	15.879704000	17.837188000	15.358092000
H	16.719990000	17.275253000	14.945436000
P	19.849189000	18.203338000	18.101047000
C	19.089201000	16.536604000	18.545749000
H	18.832458000	16.665089000	19.610469000
C	17.790748000	16.327665000	17.782115000
H	17.975728000	16.221870000	16.701878000
H	17.088815000	17.157042000	17.939046000
H	17.296560000	15.402242000	18.118805000
C	20.006257000	15.331596000	18.390294000
H	19.455600000	14.408114000	18.635178000
H	20.882862000	15.368215000	19.052905000
H	20.365075000	15.223938000	17.353554000
C	21.585222000	17.990268000	18.753104000
H	21.982461000	17.041712000	18.355654000
C	21.511261000	17.903242000	20.274461000
H	22.522758000	17.827803000	20.706086000
H	20.935461000	17.033457000	20.624862000
H	21.022435000	18.804530000	20.679800000
C	22.504512000	19.120979000	18.325814000
H	22.131993000	20.083352000	18.705064000
H	22.598334000	19.195807000	17.233209000
H	23.515428000	18.966449000	18.738113000
P	19.180041000	21.866640000	17.959718000
C	18.861340000	23.251865000	19.172084000
H	18.958677000	24.202110000	18.620631000
C	19.884337000	23.219216000	20.298402000
H	19.638518000	23.976736000	21.060012000
H	20.908833000	23.420605000	19.951438000
H	19.874069000	22.233283000	20.785124000
C	17.445660000	23.134507000	19.714006000
H	17.341416000	22.213746000	20.307992000
H	16.697399000	23.109850000	18.905994000
H	17.207194000	23.985978000	20.372527000
C	20.920145000	22.259604000	17.357271000
H	21.548492000	21.870893000	18.175942000
C	21.218883000	21.462097000	16.096160000
H	20.660034000	21.864242000	15.235311000
H	20.943839000	20.404839000	16.199366000
H	22.291958000	21.515525000	15.849194000
C	21.259269000	23.729152000	17.143981000
H	22.332535000	23.829039000	16.908428000
H	21.063279000	24.357692000	18.023857000
H	20.704352000	24.152063000	16.292407000
H	14.867560000	16.814248000	20.333844000
H	19.378991000	20.205627000	19.821940000
H	16.774396000	20.103361000	19.373413000

Ferrocene – PBE0

Fe	18.470240000	0.033740000	7.198373000
C	17.703250000	-1.698292000	6.424650000
C	19.866426000	-0.339238000	8.647808000
C	18.721849000	-1.186968000	5.575949000
C	16.685485000	-0.713027000	6.531657000
C	18.607814000	-0.046398000	9.238455000
C	20.254875000	0.780882000	7.864908000
C	18.332835000	0.114035000	5.158358000
C	17.074234000	0.407112000	5.748949000
C	18.218514000	1.254451000	8.820695000
C	19.236909000	1.765955000	7.971862000
H	17.716475000	-2.653421000	6.928657000
H	20.415063000	-1.264066000	8.748638000
H	19.645301000	-1.685681000	5.321009000
H	15.790195000	-0.789260000	7.130863000
H	18.031798000	-0.710014000	9.866261000
H	21.150134000	0.857207000	7.265667000
H	18.908959000	0.777564000	4.530540000
H	16.525987000	1.332183000	5.648367000
H	17.295088000	1.753129000	9.075843000
H	19.223266000	2.720920000	7.467567000

Ferrocene (+) – PBE0

Fe	18.470311000	0.033789000	7.198468000
C	17.696075000	-1.705101000	6.412845000
C	19.874166000	-0.333746000	8.660308000
C	18.715275000	-1.193465000	5.563474000
C	16.677602000	-0.719161000	6.519859000
C	18.614681000	-0.040734000	9.251353000
C	20.262862000	0.787117000	7.876860000
C	18.326058000	0.108423000	5.145549000
C	17.066623000	0.401692000	5.736553000
C	18.225141000	1.261047000	8.833391000
C	19.244160000	1.772836000	7.983887000
H	17.718950000	-2.651059000	6.932515000
H	20.412417000	-1.265755000	8.744393000
H	19.647054000	-1.683715000	5.324916000
H	15.792894000	-0.787050000	7.134715000
H	18.029553000	-0.711906000	9.861672000
H	21.147340000	0.855208000	7.261691000
H	18.911430000	0.779372000	4.535234000
H	16.528480000	1.333766000	5.652379000
H	17.292937000	1.750771000	9.071383000
H	19.220689000	2.718484000	7.463632000

Et_2O – PBE0

H	-1.245005000	-0.004001000	-3.682468000
O	-1.432432000	-0.203746000	-1.047463000
C	-0.945515000	-0.297065000	0.261651000
C	-1.520248000	-1.525199000	0.921954000
H	-1.215577000	0.611962000	0.839674000
H	0.164004000	-0.344858000	0.258574000
H	-1.229617000	-2.434839000	0.373571000
H	-1.154610000	-1.613582000	1.956066000
H	-2.619903000	-1.476427000	0.947423000
C	-0.942939000	0.905608000	-1.747387000
C	-1.522270000	0.913141000	-3.139751000
H	0.166205000	0.875653000	-1.792856000
H	-1.206893000	1.844448000	-1.216053000
H	-2.621156000	0.972071000	-3.104555000
H	-1.146761000	1.776833000	-3.709009000

$(Et_2O)_2H^+$ - PBE0

H	-4.586150000	6.313311000	-2.970262000
O	-5.498965000	3.863469000	-2.476913000
C	-6.099772000	2.800272000	-1.705342000
C	-5.101983000	1.696307000	-1.493058000
H	-6.999869000	2.449866000	-2.237235000
H	-6.413990000	3.247402000	-0.750733000
H	-4.212797000	2.058828000	-0.956897000
H	-5.571532000	0.911016000	-0.882916000
H	-4.787131000	1.238163000	-2.441816000
C	-6.305058000	5.052389000	-2.612794000
C	-5.563891000	6.082052000	-3.418678000
H	-6.506713000	5.410755000	-1.592426000
H	-7.265057000	4.775423000	-3.078629000
H	-5.420898000	5.773709000	-4.465179000
H	-6.155709000	7.008747000	-3.431905000
H	-4.909338000	3.501907000	-3.462770000
O	-4.326204000	3.107328000	-4.417072000
C	-5.141455000	2.968954000	-5.603695000
C	-2.988377000	3.621439000	-4.605883000
C	-4.924438000	1.634636000	-6.262802000
C	-2.202800000	3.469985000	-3.334055000
H	-3.065643000	4.673563000	-4.925454000
H	-2.538720000	3.043543000	-5.425140000

H	-1.186847000	3.855999000	-3.501260000
H	-2.122003000	2.414176000	-3.037149000
H	-2.644727000	4.043350000	-2.505784000
H	-6.180066000	3.079041000	-5.260297000
H	-4.911138000	3.810171000	-6.275401000
H	-3.886673000	1.508184000	-6.605127000
H	-5.578736000	1.561231000	-7.144220000
H	-5.175761000	0.810554000	-5.579051000
